# Indirect effects of the COVID-19 pandemic: A cause-of-death analysis of life expectancy changes in 24 countries, 2015 to 2022

**DOI:** 10.1093/pnasnexus/pgae508

**Published:** 2024-12-19

**Authors:** Antonino Polizzi, Luyin Zhang, Sergey Timonin, Aashish Gupta, Jennifer Beam Dowd, David A Leon, José Manuel Aburto

**Affiliations:** Department of Sociology, University of Oxford, Oxford OX1 1JD, United Kingdom; Leverhulme Centre for Demographic Science, University of Oxford, Oxford OX1 1JD, United Kingdom; Nuffield College, University of Oxford, Oxford OX1 1NF, United Kingdom; Nuffield Department of Population Health, University of Oxford, Oxford OX3 7LF, United Kingdom; Leverhulme Centre for Demographic Science, University of Oxford, Oxford OX1 1JD, United Kingdom; Office of Population Research, Princeton University, Princeton, NJ 08544, USA; School of Demography, College of Arts and Social Sciences, Australian National University, Canberra, ACT 2601, Australia; Department of Sociology, University of Oxford, Oxford OX1 1JD, United Kingdom; Leverhulme Centre for Demographic Science, University of Oxford, Oxford OX1 1JD, United Kingdom; Nuffield College, University of Oxford, Oxford OX1 1NF, United Kingdom; Leverhulme Centre for Demographic Science, University of Oxford, Oxford OX1 1JD, United Kingdom; Nuffield College, University of Oxford, Oxford OX1 1NF, United Kingdom; Nuffield Department of Population Health, University of Oxford, Oxford OX3 7LF, United Kingdom; London School of Hygiene and Tropical Medicine, London WC1E 7HT, United Kingdom; Department of Sociology, University of Oxford, Oxford OX1 1JD, United Kingdom; Leverhulme Centre for Demographic Science, University of Oxford, Oxford OX1 1JD, United Kingdom; Nuffield College, University of Oxford, Oxford OX1 1NF, United Kingdom; London School of Hygiene and Tropical Medicine, London WC1E 7HT, United Kingdom

**Keywords:** demography, cardiovascular disease, COVID-19, mortality, Social Sciences: Demography

## Abstract

Worldwide, mortality was strongly affected by the COVID-19 pandemic, both directly through COVID-19 deaths and indirectly through changes in other causes of death. Here, we examine the impact of the pandemic on COVID-19 and non-COVID-19 mortality in 24 countries: Australia, Austria, Brazil, Bulgaria, Canada, Chile, Croatia, Czechia, Denmark, England and Wales, Hungary, Japan, Latvia, Lithuania, The Netherlands, Northern Ireland, Poland, Russia, Scotland, South Korea, Spain, Sweden, Switzerland, and the United States. Using demographic decomposition methods, we compare age- and cause-specific contributions to changes in female and male life expectancy at birth in 2019–2020, 2020–2021, and 2021–2022 with those before the COVID-19 pandemic (2015–2019). We observe large life expectancy losses due to COVID-19 in most countries, usually followed by partial recoveries. Life expectancy losses due to cardiovascular disease (CVD) mortality were widespread during the pandemic, including in countries with substantial (Russia, Central and Eastern Europe, and the Baltic countries) and more modest (United States) improvements in CVD mortality before the pandemic. Many Anglo-Saxon countries, including Canada, Scotland, and the United States, continued their prepandemic trajectories of rising drug-related mortality. Most countries saw small changes in suicide mortality during the pandemic, while alcohol mortality increased and cancer mortality continued to decline. Patterns for other causes were more variable. By 2022, life expectancy had still not returned to prepandemic levels in several countries. Our results suggest important indirect effects of the pandemic on non-COVID-19 mortality through the consequences of COVID-19 infection, nonpharmaceutical interventions, and underreporting of COVID-19-related deaths.

**Significance Statement**

The COVID-19 pandemic affected mortality from different causes of death, including COVID-19 infection, cardiovascular diseases, cancer, and external and substance-related mortality. However, most existing studies have not considered the heterogeneous effects of the pandemic on different causes of death across countries. Using state-of-the-art decomposition methods, we highlight the wide variation in the direct and indirect impacts of the pandemic on COVID-19 mortality and 11 non-COVID-19 causes of death across 24 countries. Most countries saw continued declines in cancer mortality but increases in cardiovascular mortality after 2019. Some countries also saw a noticeable rise in accident, alcohol, and drug mortality. Future life expectancy changes will have to be interpreted cautiously, keeping in mind the pandemic's indirect effects on non-COVID-19 mortality.

## Introduction

Life expectancy trajectories have become more diverse in recent years. Japan continues to lead global life expectancy trends with steady linear increases (1), while South Korea saw a remarkable 16-year improvement from 1970 to 2005 (2). Most European countries have also experienced steady, and parallel, increases in life expectancy at birth, with Central and Eastern Europe and the Baltics catching up since the mid-1990s after periods of stagnation and decline (3, 4). However, since 2010, many high-income countries, including Western European countries, Australia, and Canada, have seen a slowdown in mortality improvements (5). In England and Wales, life expectancy has stagnated since 2011, due to high working-age mortality and the lagged effects of smoking behaviors (6). In the United States, life expectancy even declined before the COVID-19 pandemic, due to a slowdown in improvements in cardiovascular mortality and increases in alcohol-related deaths, fatal drug overdoses, and suicides—often collectively referred to as “deaths of despair.” (7, 8) Similarly, life expectancy improvements in many Latin American countries, such as Brazil, have been slowed down by high levels of violence (9).

The COVID-19 pandemic further diversified life expectancy trajectories, with many countries experiencing substantial losses in life expectancy at birth in 2020, albeit with large heterogeneity (10–14). In 2021, the pandemic's impact on life expectancy varied further (15), with most of Western Europe seeing improvements compared with 2020, while Central and Eastern Europe, the Baltic states, and the United States experienced additional life expectancy declines (16, 17). Moreover, the age profile of mortality impacts in 2021 was often younger, with working-age groups in many countries contributing more to life expectancy losses than in 2020. Latin American countries, including Brazil and Chile, saw significant excess mortality in 2020 (18), with existing studies highlighting large variation in life expectancy declines at the subnational level (19–22). Notably, East Asian countries and Australia saw almost no drops in life expectancy in 2020 or 2021 (23, 24). Finally, in 2022 and 2023, many countries experienced (further) recoveries in life expectancy, usually driven by improvements in mortality at older ages (25). Nevertheless, life expectancy had rarely returned to pre-2020 levels (25), illustrating the long-lasting disruption of life expectancy trends caused by the COVID-19 pandemic. Some countries, such as Australia (24), even experienced (further) life expectancy losses in 2022 and/or 2023 (25).

Life expectancy is an important measure of the pandemic mortality burden because it is comparable over time and across countries. However, most studies rely on all-cause mortality, and few quantify how COVID-19 vs. other causes of death contributed to observed life expectancy changes during the pandemic years (10, 22, 24, 26). Examining the contributions of other causes of death (e.g. cardiovascular diseases [CVDs], cancers, suicide) to life expectancy changes can illuminate the indirect pathways through which the pandemic affected mortality and population health. A comparison across countries can help highlight differences in pandemic experiences that might inform future policy and pandemic preparedness.

For example, the United States recorded >350,000 deaths from COVID-19 in 2020 (https://wonder.cdc.gov). Excess mortality in the same year was estimated even higher, over 500,000, contributing to a large drop in life expectancy at birth of around 25.5 months (17, 27, 28). While there is evidence that deaths attributed to COVID-19 may be undercounted or misclassified (29, 30), it is likely that the pandemic did affect mortality from other causes of death, both positively and negatively. For example, lockdowns may have led to lower mortality from accidents and other external causes of death (31), whereas the overload of healthcare systems in many countries could have led to increased mortality from diseases that require treatments or prompt interventions (e.g. cancer, heart attacks). Similarly, fear of infection and avoidance of hospital care may have increased mortality from certain conditions, including acute cardiovascular events. This is consistent with the large increase in deaths at home seen in some countries (32). Notably, the impact of these changes in cause-of-death profiles is likely to be different across countries due to differences in the overall quality of healthcare systems, socioeconomic resources and inequalities, prepandemic trends in mortality, and the pharmaceutical and nonpharmaceutical interventions employed during the pandemic.

In this article, we compare changes in female and male life expectancy at birth in 2019–2020, 2020–2021, and 2021–2022 with those before the COVID-19 pandemic (2015–2019) in 24 countries. Importantly, we go beyond the analysis of all-cause mortality by estimating the contributions of COVID-19 and 11 non-COVID-19 causes of death to changes in life expectancy, including mortality from CVD, cancer, acute respiratory disease, chronic obstructive pulmonary disease (COPD), infectious diseases, as well as alcohol, drug, suicide, and accident mortality. Our analysis draws attention to the broad disruptions in mortality caused by SARS-CoV-2, highlights variation in how different countries adapted to the initial pandemic shocks, and allows us to hypothesize how this may affect their life expectancy trajectories in the future.

## Results

### Life expectancy differences and trends

Among the 24 countries included in our study (see Materials and Methods), life expectancy ranged from a low of 65.6 years for Russian males in 2021 to a high of 87.9 years for Japanese females in 2020 (Tables 1 and 2). From 2015 to 2019, Russia had the largest gains in female and male life expectancy, at 1.5 and 2.4 years, respectively. The smallest gains were observed in the United States, improving only 0.3 and 0.2 years among females and males, respectively.

**Table 1.** Female life expectancy at birth and changes in female life expectancy at birth by country, 2015–2022.

Sex	Country	Life expectancy at birth					Changes in life expectancy			
		2015	2019	2020	2021	2022	2015–2019	2019–2020	2020–2021	2021–2022
Female	Australia	84.5	85.1	85.8	85.5	84.8	+0.6	+0.7	−0.3	−0.7
	Austria	83.5	84.1	83.7	83.8	—	+0.6	−0.4^a^	+0.1	—
	Brazil	78.3	78.9	77.9	76.0	—	+0.6	−1.0	−2.0	—
	Bulgaria	78.0	78.6	77.3	75.0	—	+0.6	−1.3	−2.4	—
	Canada	84.4	84.8	84.2	84.4	83.8	+0.4	−0.6	+0.3	−0.7
	Chile	83.0	83.9	83.0	82.1	—	+0.9	−0.9	−0.8	—
	Croatia	80.6	81.5	80.8	79.9	—	+1.0	−0.7	−0.9	—
	Czechia	81.4	82.0	81.2	80.4	—	+0.6	−0.8	−0.8	—
	Denmark	82.7	83.4	83.5	84.0	—	+0.7	+0.1	+0.6^b^	—
	England and Wales	82.9	83.7	82.5	82.9	83.3	+0.8	−1.1	+0.4	+0.4
	Hungary	78.8	79.6	78.9	77.7	79.3	+0.8	−0.6	−1.3	+1.6
	Japan	87.1	87.6	87.9	87.7	—	+0.5	+0.3	−0.2	—
	Latvia	79.4	80.1	80.0	78.1	—	+0.6	−0.1	−1.9	—
	Lithuania	79.5	80.9	79.9	78.7	79.9	+1.4	−1.0	−1.2	+1.2
	The Netherlands	83.1	83.5	83.0	83.0	83.1	+0.5	−0.5	−0.1	+0.1
	Northern Ireland	82.2	82.8	81.9	82.0	—	+0.6	−0.8	+0.1	—
	Poland	81.2	81.5	80.4	79.4	—	+0.3	−1.1	−1.0	—
	Russia	76.6	78.0	76.2	74.4	—	+1.5	−1.8	−1.9	—
	Scotland	81.0	81.2	80.6	80.5	—	+0.3	−0.6	−0.2	—
	South Korea	85.6	87.0	87.2	87.2	—	+1.4	+0.2	+0.0	—
	Spain	85.2	86.0	84.9	85.7	—	+0.8	−1.1	+0.8	—
	Sweden	83.9	84.6	84.1	84.7	84.6	+0.7	−0.4	+0.6	−0.1
	Switzerland	84.8	85.5	85.0	85.6	—	+0.7	−0.5	+0.6	—
	USA	80.9	81.3	79.7	79.3	80.2	+0.3	−1.5	−0.4	+0.9

^a^Life expectancy decreases in authors’ dataset, but increases in UNWPP.

^b^Life expectancy increases in authors’ dataset, but decreases in UNWPP.

**Table 2.** Male life expectancy at birth and changes in male life expectancy at birth by country, 2015–2022.

Sex	Country	Life expectancy at birth					Changes in life expectancy			
		2015	2019	2020	2021	2022	2015–2019	2019–2020	2020–2021	2021–2022
Male	Australia	80.5	80.9	81.7	81.6	80.8	+0.4	+0.8	−0.1^a^	−0.7
	Austria	78.6	79.5	78.9	78.8	—	+0.9	−0.6	−0.1	—
	Brazil	71.7	72.8	71.2	69.4	—	+1.0	−1.6	−1.8	—
	Bulgaria	71.1	71.5	69.8	67.9	—	+0.3	−1.6	−1.9	—
	Canada	80.2	80.5	79.6	79.6	79.2	+0.3	−0.9	−0.1^a^	−0.3
	Chile	78.2	79.5	78.0	77.1	—	+1.2	−1.5	−0.8	—
	Croatia	74.1	75.0	74.3	73.4	—	+0.9	−0.7	−0.9	—
	Czechia	75.5	76.2	75.1	74.0	—	+0.7	−1.1	−1.1	—
	Denmark	78.8	79.5	79.6	80.3	—	+0.7	+0.1	+0.7^b^	—
	England and Wales	79.4	80.0	78.6	78.9	79.5	+0.7	−1.4	+0.3	+0.6
	Hungary	72.1	73.0	72.2	70.7	72.6	+0.8	−0.7	−1.5	+1.9
	Japan	81.0	81.7	81.9	81.8	—	+0.7	+0.2	−0.1	—
	Latvia	69.9	71.1	70.8	68.5	—	+1.2	−0.3	−2.3	—
	Lithuania	69.1	71.4	69.9	69.3	70.9	+2.3	−1.5	−0.6	+1.6
	The Netherlands	79.7	80.4	79.6	79.6	80.0	+0.7	−0.8	+0.0	+0.4
	Northern Ireland	78.1	79.0	78.1	78.1	—	+0.8	−0.9	+0.0	—
	Poland	73.3	73.9	72.3	71.5	—	+0.6	−1.5	−0.8	—
	Russia	65.9	68.3	66.6	65.6	—	+2.4	−1.7	−0.9	—
	Scotland	76.9	77.2	76.1	76.3	—	+0.3	−1.1	+0.2	—
	South Korea	79.1	80.5	80.7	80.7	—	+1.4	+0.2^b^	+0.0	—
	Spain	79.7	80.6	79.4	80.1	—	+0.9	−1.2	+0.7	—
	Sweden	80.2	81.2	80.4	81.1	81.2	+0.9	−0.8	+0.7	+0.1
	Switzerland	80.6	81.9	80.9	81.6	—	+1.2	−0.9	+0.7	—
	USA	76.5	76.7	74.6	74.0	75.2	+0.2	−2.1	−0.7	+1.3

^a^Life expectancy decreases in authors’ dataset, but increases in UNWPP.

^b^Life expectancy increases in authors’ dataset, but decreases in UNWPP.

In 2020, life expectancy declined in most countries, except for females and males in Australia, Denmark, Japan, and South Korea. The largest decline was seen for males in the United States (−2.1 years). In 2021, most countries experienced further declines in life expectancy, with the largest losses, of >2 years, occurring in Bulgaria (females) and Latvia (males). Spanish females experienced the largest gains in life expectancy in 2021, at 0.8 years. Although often substantial, life expectancy gains in 2021 rarely compensated for the losses experienced in 2020, so that life expectancy in 2021 was still lower than observed before the pandemic, except for females and males in Australia, Denmark, Japan, and South Korea, as well as females in Sweden and Switzerland. Finally, in 2022, most countries with available cause-of-death information experienced gains in life expectancy, except for females and males in Australia and Canada, as well as females in Sweden, where life expectancy declined (further). The 2022 gains in life expectancy were greatest among males in Hungary, at 1.9 years, while losses were greatest among females and males in Australia, as well as females in Canada, at −0.7 years. By 2022, only Sweden had returned to prepandemic life expectancy levels.

Overall, the life expectancy trends based on our dataset agree well with the trends published in the United Nations World Population Prospects (UNWPP) 2024 revision (33). The last four columns in Tables 1 and 2 highlight differences in time trends between our dataset and the UNWPP where they occur (see also Fig. S4). Superscript **a** indicates a decrease in life expectancy according to our dataset, while UNWPP reports an increase in life expectancy. Conversely, superscript **b** indicates an increase in life expectancy according to our dataset, while UNWPP reports a decrease in life expectancy. For example, in Denmark, life expectancy in 2021 increased by 0.6 years (females) and 0.7 years (males) according to our estimates, whereas UNWPP reports a life expectancy decline for that year.

### Cause-of-death contributions

**Fig. 1.**
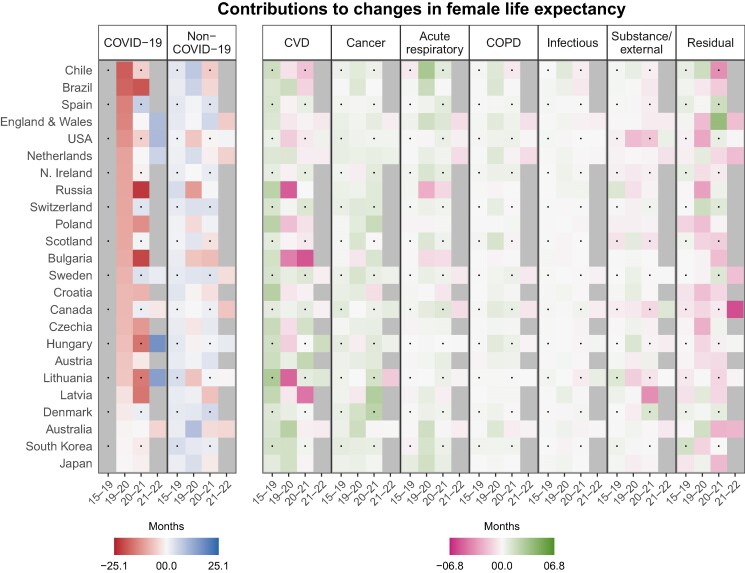
Cause-of-death-specific contributions to changes in female life expectancy at birth by country and period. Contributions in months. Tiles for period 2015–2019 show average annual contributions. Cause-of-death data for period 2021–2022 only available for: Australia, Canada, England and Wales, Hungary, Lithuania, the Netherlands, Sweden, and the United States.

Figure 1 shows cause-of-death-specific contributions to changes in female life expectancy in months over the periods 2015–2019, 2019–2020, 2020–2021, and 2021–2022 for each of the 24 countries. For the period 2015–2019, Fig. 1 shows average annual contributions over the 4-year observation window. Contributions for each country–period–cause combination are represented by square tiles, with darker tiles indicating larger negative or positive contributions and lighter tiles representing smaller negative or positive contributions. Negative values indicate that a given cause of death contributed to losses in life expectancy over a given period, whereas positive values indicate life expectancy gains. Gray tiles for a given period indicate a lack of available cause-of-death information. The countries in Fig. 1 are ordered according to the contribution from deaths coded as COVID-19 to life expectancy changes over the period 2019–2020, the first year of the pandemic. The black dots in Fig. 1 serve as visual guide. The Materials and Methods section describes our coding and decomposition procedures in more detail. Additionally, Fig. S7 breaks down the cause-specific contributions by 10-year age groups (0–9, 10–19, …, 70–79, 80+).

The first two panels in Fig. 1 respectively show the contributions from COVID-19 and all non-COVID-19 mortality to changes in female life expectancy, on a scale ranging from negative contributions (red) over no contributions (white) to positive contributions (blue). In 2019–2020, COVID-19 contributed to sizeable losses in female life expectancy in almost all countries (leftmost panel in Fig. 1). The largest losses from COVID-19 were seen in Spain (−14.4 months), Brazil (−17.8), and Chile (−18.7), while Australia (−0.5 months), South Korea (−0.4), and Japan (−0.2) experienced the smallest losses. In 2020–2021, COVID-19 contributed to additional losses in female life expectancy, particularly in Brazil, Russia, and among Central and Eastern European and Baltic countries. Although COVID-19 mortality was mostly concentrated in older age groups, many of the latter countries also had substantial negative contributions at middle adult ages (see Fig. S7). Finally, most of the countries with available cause-of-death data in 2021–2022 experienced improvements in COVID-19 mortality, except for Australia and Canada, where COVID-19 mortality worsened (further).

Moving to mortality from all non-COVID-19 causes (second panel in Fig. 1), all countries experienced gains in female life expectancy over the period 2015–2019. These contributions ranged from an average of 0.8 months per year in Scotland to 4.4 months per year in Russia. In contrast, we observed large heterogeneity in life expectancy contributions for the periods 2019–2020, 2020–2021, and 2021–2022, with sizeable life expectancy losses due to non-COVID-19 mortality in Russia (−11.2 months), Lithuania (−6.3 months), the United States (−5.9 months), and all Central and Eastern European countries (−0.6 months in Croatia to −6.1 months in Bulgaria) in 2020. In 2021, losses in female life expectancy due to non-COVID-19 mortality were geographically more dispersed. Many of these losses persisted even in 2022, with only Hungary and the United States experiencing recovery.

The remaining panels in Fig. 1 break the contributions from non-COVID-19 mortality down into more detailed causes of death. We differentiate between mortality from (i) CVDs, (ii) cancer, (iii) acute respiratory diseases, (iv) COPD, (v) certain infectious diseases, and (vi) substance and external mortality, with all remaining causes of death grouped into a residual category. Contributions from these seven causes of death are shown on a scale ranging from negative contributions (pink) over no contributions (white) to positive contributions (green). Figure S5 further disaggregates the category “CVD” into “acute CVD” and “other CVD,” and further disaggregates the category “substance/external” into “alcohol,” “drug,” “suicide,” and “other external.”

Improvements in CVD mortality constituted one of the largest sources of gains in female life expectancy over the period 2015–2019. On average, CVD mortality contributed to life expectancy gains of 0.5 (United States) to 3.0 (Lithuania) months per year. Improvements were seen in mortality from both acute and nonacute CVD (see Fig. S5) and were concentrated above age 60 (see Fig. S7). In the first 2 years of the pandemic, earlier improvements in CVD mortality were reversed in several countries, with the largest losses seen in Russia in 2019–2020 (−5.3 months) and Bulgaria in 2020–2021 (−5.5 months). These losses were disproportionately driven by rising mortality from nonacute CVD. In Russia as well as many Central and Eastern European and Baltic countries, the age profile of CVD- and COVID-19-related life expectancy losses strongly overlapped. Among many countries with available cause-of-death data, life expectancy losses from CVD mortality persisted even in 2021–2022, such as in England and Wales (−0.5 months).

Next, reductions in cancer mortality contributed substantially to gains in female life expectancy in most countries, including during pandemic years. The bulk of these improvements in cancer mortality happened above age 50 (see Fig. S7). During the pandemic years, individual countries experienced increases in cancer mortality, most notably Lithuania in 2021–2022, where rising cancer mortality led to life expectancy losses of 1.6 months among females.

The following three panels show contributions from acute respiratory diseases, COPD, and infectious diseases to female life expectancy changes. These contributions were generally small and mixed. Particularly noteworthy were the large gains from acute respiratory diseases in Chile (3.2 months) in 2019–2020 as well as the large losses in Russia in 2019–2020 and 2020–2021 (−2.6 and −1.1 months, respectively). In Bulgaria and Russia, the negative contributions from COVID-19 and acute respiratory mortality had similar age profiles (see Fig. S7).

Contributions from substance and external mortality to female life expectancy changes were generally small. In 2019–2020, negative contributions were particularly pronounced for the United States (−2.1 months) and Lithuania (−1.4 months). While increasing drug-related mortality in middle adulthood was mostly responsible for the negative contributions in the United States, alcohol mortality was driving the patterns in Lithuania (Fig. S5). In 2020–2021, negative contributions from substance and external mortality also stood out in Latvia (−3.7 months), which were driven by alcohol and accident mortality. While generally small, alcohol-related losses in female life expectancy were visible across a broad range of countries during the pandemic, whereas drug-related losses were mostly visible in Canada, Northern Ireland, Scotland, and the United States. Suicide mortality declined in many countries during the pandemic years, with comparatively large increases in Japan (−0.7 months) and Northern Ireland (−0.7 months) in 2019–2020.

Finally, while more difficult to interpret, we note that a large number of countries experienced losses in female life expectancy due to mortality increases in the residual category (up to −5.7 months in Canada in 2021–2022). Nonetheless, some relatively large improvements were also visible, such as for England and Wales in 2020–2021 (4.0 months).

**Fig. 2.**
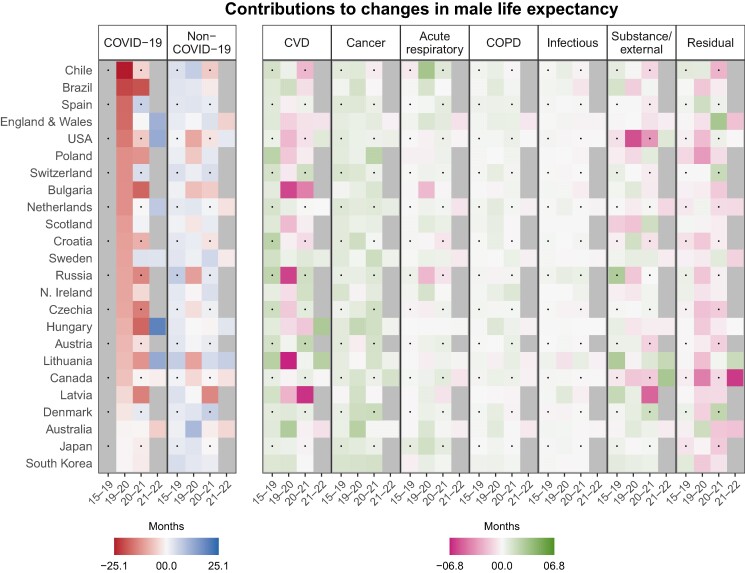
Cause-of-death-specific contributions to changes in male life expectancy at birth by country and period. Contributions in months. Tiles for period 2015–2019 show average annual contributions. Cause-of-death data for period 2021–2022 only available for: Australia, Canada, England and Wales, Hungary, Lithuania, The Netherlands, Sweden, and the United States.

Figure 2 shows the decomposition results for changes in male life expectancy. To facilitate comparison, the decomposition results for females and males are shown on the same scale. Like for females, the countries on the vertical axis are ordered according to the contributions from COVID-19 mortality in 2019–2020. Although the order of countries in Figs. 1 and 2 is different, the countries with the smallest (Australia, Japan, South Korea) and largest (Brazil, Chile, Spain) contributions from deaths coded as COVID-19 in 2019–2020 were the same for females and males. In 2020–2021, a few countries (Denmark, England and Wales, Scotland, Spain, Sweden, and Switzerland) experienced gains in male life expectancy due to reductions in COVID-19 mortality, while most countries experienced further losses. Brazil, Russia, the Baltics, and several Central and Eastern European countries experienced the largest COVID-19-related declines during this period. In contrast, in 2021–2022, we observed life expectancy gains due to COVID-19 in most countries with available cause-of-death data, except for Australia and Canada.

In 2019–2020, Russia, Lithuania, and many Central and Eastern European and Anglo-Saxon countries (Canada, Northern Ireland, Scotland, and the United States) saw male life expectancy losses due to rising non-COVID-19 mortality. Similar losses were seen on a geographically wider scale in 2020–2021 and continued in 2021–2022, except for Hungary, Lithuania, and the United States. CVD mortality, particularly nonacute CVD, appeared to be the main driver of changes in non-COVID-19 mortality during the pandemic years (see Fig. S6).

Before and during the pandemic, we observed widespread positive contributions to changes in male life expectancy from reductions in cancer mortality, with few exceptions. Contributions from acute respiratory diseases, COPD, and infectious diseases were small and mixed, with some exceptionally large positive or negative contributions from acute respiratory diseases in Bulgaria, Chile, and Russia during the pandemic.

Contributions from substance and external mortality were generally larger among males vs. females. Before the pandemic, life expectancy losses due to substances and external causes were particularly visible for Canada, Scotland, and the United States. These negative contributions were predominantly driven by rising drug-related mortality, which continued to worsen during the pandemic, except for Scotland in 2020–2021 and Canada and the United States in 2021–2022 (see Fig. S6). In the United States, mortality from other external causes, including accidents, also worsened noticeably during the pandemic. Several other countries experienced losses in male life expectancy due to rising substance and external mortality during the pandemic years. Our more detailed decomposition results in Fig. S6 suggest that these negative contributions were to a large extent driven by alcohol and/or drug mortality, which increased in many countries during the pandemic, most notably in Latvia in 2020–2021. Like for females, suicide and accident mortality often declined during the pandemic years, with many of the positive contributions appearing larger for males, especially for accident mortality.

Finally, similar to the decomposition results for females, we observed widespread losses in male life expectancy due to rising mortality in the residual category, with some exceptions, such as England and Wales in 2020–2021.

## Discussion

The first waves of the COVID-19 pandemic in 2020 induced sizable life expectancy losses in many countries around the world, with very few exceptions (11, 13, 15, 17). In many countries, life expectancy continued to deteriorate in 2021 and had still not returned to prepandemic levels by 2022 (25). In this study, we comprehensively analyzed life expectancy changes due to 12 major causes of death in 24 countries during the period 2015–2021, and in eight countries through 2022. Comparing changes before the COVID-19 pandemic with those in 2020–2022 puts into perspective the magnitude and diversity of the sharp mortality changes observed since the first SARS-CoV-2 outbreaks. While most countries in our study experienced life expectancy declines at some point during the pandemic, life expectancy trajectories showed considerable variability after 2019. In particular, our results highlight how different causes of death were driving life expectancy trends over this period.

We find that prior to the pandemic, declines in cardiovascular and cancer mortality were the main drivers of increases in life expectancy. Russia as well as many Central and Eastern European and Baltic countries experienced particularly large gains due to improvements in CVD mortality, consistent with their late onset of the cardiovascular mortality transition (4, 34). These improvements were concentrated at middle and older ages. In contrast, changes in CVD mortality were smaller in Canada, England and Wales, Northern Ireland, Scotland, and the United States. Stagnation in CVD mortality improvements in these countries has been linked to changes in behavioral risk factors, such as rising levels of obesity and alcohol consumption (35). Limited gains from further reductions in smoking-related mortality have also been discussed as a potential cause for stagnating CVD mortality in Anglo-Saxon countries (36).

Our results also show that drug-related mortality contributed to life expectancy losses in most English-speaking countries prior to the pandemic. This is consistent with previous evidence showing worsening mortality due to fatal drug overdoses in these countries, especially among males (37). While synthetic opioids have played a dominant role for drug mortality trends in Canada and the United States, the United Kingdom countries have also seen a fast rise in mortality involving benzodiazepines and cocaine (38).

Consistent with previous studies, we find that most countries suffered substantial drops in life expectancy due to COVID-19 mortality in 2019–2020. These losses were sizable in England and Wales, Spain, and the United States. However, life expectancy losses skewed much younger in the United States, potentially due to more risk factors for severe COVID-19 at younger ages or social factors that increased exposure for working-age populations (10, 39). Brazil and Chile also saw large life expectancy declines from COVID-19 in 2019–2020. Previous studies have documented considerable subnational variation in COVID-19 mortality in these two countries (19, 20). In addition, other Latin American countries have experienced similar or larger COVID-19-related losses in life expectancy (21). In line with previous studies, we find that COVID-19-related life expectancy losses in 2019–2020 were smallest in Australia, Japan, and South Korea (23, 24).

In 2021, many Central and Eastern European and Baltic countries saw larger losses due to COVID-19 than in the first year of the pandemic. Like the United States, these countries generally exhibited a younger mortality profile of COVID-19 deaths than many Western European countries (17). Finally, among the eight countries with available cause-of-death information for 2021–2022, females and males in Australia and Canada experienced (further) losses in life expectancy due to rising COVID-19 mortality. Evidence from Australia suggests that COVID-19-related life expectancy losses in 2022 may be due to the comparatively late relaxation of nonpharmaceutical interventions and restrictions, despite high vaccination coverage (24).

Our cause-of-death-specific decomposition results support the hypothesis that the pandemic affected a wide range of causes of death unrelated to SARS-CoV-2. This may have occurred through the indirect impacts of nonpharmaceutical interventions, such as lockdowns, the increased strain on healthcare systems, preexisting social inequalities, as well as the misclassification of COVID-19 deaths. In particular, in 15 out of 24 countries (females) and 16 out of 24 countries (males), rising non-COVID-19 mortality contributed to life expectancy losses in 2020 and/or 2021, albeit with large variation in magnitude. In 2022, (further) life expectancy losses from non-COVID-19 mortality were seen in six (female) and five (male) out of eight countries with available cause-of-death data.

Rising CVD mortality was a major driver of increasing non-COVID-19 mortality during the pandemic, especially in 2019–2020 and 2020–2021. In 2020 and 2021 combined, we observed the greatest life expectancy losses from CVD among both females and males in Bulgaria, ∼10 months. Only females in Canada and Switzerland, males in the Netherlands, and females and males in Austria, Denmark, South Korea, and Sweden did not experience losses from CVD in 2020 and 2021. However, females in Canada and Sweden did experience subsequent losses in 2022. Miscoding of COVID-19 deaths as CVD may account for a large proportion of the changes observed (29, 30). In addition, evidence from multiple countries shows a substantial decrease in admission rates for acute CVD during the pandemic, which may have led to an increase in untreated strokes and heart attacks (40–42), explaining some of the observed life expectancy losses, including from nonacute CVD. More research to aid our understanding of the impacts of the pandemic on cardiovascular mortality in the medium and long term is needed. Recent evidence suggests that survivors of COVID-19 are at a higher risk of incident CVD (43), which may be reflected in higher CVD mortality in future years.

In contrast to the negative pandemic trends in CVD mortality, cancer mortality usually continued to contribute to life expectancy gains, more so among males. Cumulatively in 2020 and 2021, only females in Croatia saw stagnating improvements in cancer mortality. The generally positive contributions for cancer mortality during the pandemic may be suggestive of limited disruptions to cancer care. Alternatively, our observed patterns may be the result of mortality displacement (44), with cancer patients dying of COVID-19, thereby lowering cancer-related mortality. In fact, improvements in cancer mortality in most countries during the pandemic years exceeded average annual improvements seen in the prepandemic period. Still, it is possible that the positive contributions for cancer mortality documented in our study were smaller than would have been seen in the absence of the pandemic, which would be suggestive of disruptions in cancer care. Related to this point, we observed worsening cancer mortality in several countries during the pandemic period, possibly due to delayed diagnoses or treatment (45). We suggest that changes in cancer mortality after 2019 be further explored using counterfactual estimation approaches, such as cause-of-death-specific estimation of excess mortality (46), to determine whether cancer mortality was higher or lower than expected in the absence of the COVID-19 pandemic. In addition, use of individual-level diagnostic and treatment data and a closer examination of specific slow- or fast-growing cancer types will be helpful to determine the degree to which cancer care was disrupted. It may also be possible that pandemic disruptions in cancer care will only be visible in the near or more distant future, underlining the importance of prospective data to monitor changes in cancer incidence and mortality in the coming years.

We find that COPD and acute respiratory mortality often improved during the pandemic, particularly at older ages. On the one hand, this might suggest that older age groups were particularly protected from COVID-19 and lived longer than they would have in the absence of nonpharmaceutical interventions. On the other hand, this pattern might also be suggestive of potential mortality displacement. Many older individuals that would have died from respiratory conditions may have died earlier than in the absence of the pandemic. Further research using more fine-grained mortality data is required to disentangle these two pathways.

Alcohol- and drug-related deaths, suicides, and accidents contributed substantially to life expectancy losses in several countries. Females and males in Latvia and the United States, as well as males in Canada, experienced losses of >3 months in 2020 and 2021 combined. Substance- and accident-related mortality accounted for most of these losses. Latvia has historically had high levels of alcohol- and accident-related mortality, often contributing to stagnating or declining life expectancy (4). Improvements in recent decades have led to rapid increases in life expectancy prior to 2020, which were, however, reversed during the pandemic. As discussed above, Canada, Scotland, and the United States have had high and rising levels of drug-related mortality before the pandemic. However, these countries experienced small improvements in mortality from lethal drug overdoses in some pandemic years, consistent with recent evidence of a levelling off of drug mortality (37, 47). So far, the reasons for these trend changes in lethal drug overdoses remain underexplored and may likely reflect country- and substance-specific factors (37).

The widespread rise in alcohol-related mortality shown in our study should be of concern, as longer term impacts of high alcohol consumption may be anticipated in addition to the observed pandemic increases in alcohol deaths. Despite early concerns, we find no evidence that suicide mortality strongly and systematically increased during the pandemic, with many countries even showing improvements. However, females in Japan and Northern Ireland saw comparatively large losses from suicide mortality in 2019–2020. In Northern Ireland, suicide mortality appears to have followed the upward trajectory already seen before 2020 (48), while in Japan, suicide mortality declined at the beginning of the pandemic only to increase in later waves (49, 50). Pandemic changes in accident mortality were more mixed, with many countries seeing small life expectancy gains from fewer accidents, especially among males. This is likely explained by lockdown measures and work-from-home schemes, including their effects on car traffic (31).

Finally, the residual category accounted for a large share of changes due to non-COVID-19 mortality, and most of its contributions were negative. Although we classified deaths according to broad cause-of-death categories that feature prominently in the current discourse on pandemic mortality dynamics, our findings underline that the pandemic has noticeably affected other causes of death that are less regularly studied. For example, a recent study from Australia showed rising mortality due to Parkinson's disease, disorders of gallbladder, biliary tract and pancreas, diseases of the musculoskeletal system and connective tissue, and other disorders of the urinary system in 2021 (24). The Australian case suggests that the indirect effects of the COVID-19 outbreak may have created unmet needs across a wide range of causes of death. The recent rise in the complexity of cause-of-death profiles in high-income countries means that it may become increasingly difficult to predict which causes of death will be most influential for life expectancy changes (51). Future studies and policies should increase efforts to account for this growing diversity of causes of death.

Our study has several limitations. First, our analysis is restricted to 24 countries. Understanding the experience of more countries, including low- and middle-income countries, in this comparative perspective would be valuable. Second, while our estimated life expectancy trends tend to agree well with those of more established sources such as the UNWPP, there are a few cases where the directions of life expectancy changes differ. One example is Denmark, where life expectancy increased in 2020–2021 according to our estimates, but decreased according to UNWPP estimates. Further examination of the mortality age schedules from both sources suggests that this deviation may be due to an underestimation of young adult mortality in our dataset. We note that we have used the most recent available data on population and deaths by cause to estimate mortality dynamics during the COVID-19 pandemic as accurately as possible, and we have highlighted deviations between data sources where they occurred. Finally, the process of cause-of-death coding varies across countries, and pandemic-associated impacts, such as misclassification between underlying and contributory causes of deaths, delays in registration, and the location of deaths (at home vs. hospital or nursing home), could affect estimates (52). Importantly, differences in testing capacities and willingness to assign COVID-19 as an underlying cause of death mean that an increase in CVD and respiratory mortality in some countries may reflect undercounted COVID-19 deaths (29, 30). For example, we observed a strong overlap in the age patterns of COVID-19, CVD, and acute respiratory mortality in Bulgaria and Russia, pointing toward potential large under-detection of COVID-19 deaths in vital statistics, particularly during the first year of the pandemic (53, 54). Detailed analysis of the temporal and geographic patterns of non-COVID-19 excess mortality in the United States suggests that a high proportion were likely undercounted COVID-19 deaths (30). Moreover, it is estimated that causes of death other than COVID-19 represented 19.6% of the total mortality burden associated with COVID-19 during the first year of the pandemic in the United States, with circulatory diseases making up the highest percentage of these (29). While difficult to test directly, we suspect that undercounting also accounts for much of the increased CVD mortality in Central and Eastern European and Baltic countries as well as Russia, which had even higher ratios of excess deaths to official COVID-19 deaths than the United States (27, 55). Furthermore, the fact that these countries saw large gains in life expectancy due to CVD before the pandemic as well as noticeable CVD-related losses during the pandemic may be related to the poorer public health and medical infrastructure in these countries, which may have contributed to both a later “cardiovascular revolution” and COVID-19 underreporting. Our findings highlight the need for high-quality data on causes of death, which are often lacking in many countries, and the need to develop methods to mitigate existing data limitations, such as miscoding of causes of death and a lack of data on multiple causes of death.

To summarize, we find that the indirect effects of the COVID-19 pandemic on mortality varied across countries, even those with a comparable burden of COVID-19 mortality. This variation may suggest that some explanations for changes in non-COVID-19 mortality, such as mortality displacement, do not provide a comprehensive explanation for the cross-country variation observed in our study. Rather, our results highlight variability in the impact of the pandemic on overall population health through the (non-)implementation of pharmaceutical and nonpharmaceutical interventions, as well as preexisting vulnerabilities in the population. The variable impact of the COVID-19 pandemic across countries has therefore contributed to a further diversification of life expectancy trajectories, a trend already observed prior to the outbreak of SARS-CoV-2. Importantly, the continued life expectancy losses due to rising non-COVID-19 mortality, even in 2022, represent a major puzzle and an important point of intervention for public health efforts and research in the postpandemic period.

## Materials and methods

We used data for the 2015–2022 period for 24 countries: Australia, Austria, Brazil, Bulgaria, Canada, Chile, Croatia, Czechia, Denmark, England and Wales (treated as one country), Hungary, Japan, Latvia, Lithuania, The Netherlands, Northern Ireland, Poland, Russia, Scotland, South Korea, Spain, Sweden, Switzerland, and the United States. We describe our country selection in more detail in the Supplementary Materials.

Annual death counts by sex, age group (0, 1–4, 5–9, 10–14, …, 85+/90+/95+/100+), and cause of death were taken from: (i) for England and Wales in 2021 and 2022, the United Kingdom Office for National Statistics (ONS), (ii) for Russia, the depersonalized death records provided by the Russian Federal State Statistics Service, (iii) for the United States in 2022, the Underlying Cause of Death Database compiled by the Centers for Disease Control and Prevention, and (iv) for all remaining country–year combinations, the World Health Organization Mortality Database. Cause-of-death information was available for all 24 countries for the period 2015–2021, and available for 8 countries for the year 2022: Australia, Canada, England and Wales, Hungary, Lithuania, The Netherlands, Sweden, and the United States. Recognizing the variation in death certification practices between countries, we categorized deaths by assigning ICD-10 codes to create 12 cause-of-death groups of public health and clinical relevance, in line with previous analyses (see Table S1): (i) acute CVDs (acute ischemic heart disease and strokes), (ii) other CVD, (iii) acute respiratory diseases, (iv) COPD, (v) certain infectious diseases, (vi) suicide, (vii) drug-related deaths, (viii) alcohol-related deaths, (ix) cancers, (x) other external causes of death, (xi) COVID-19, and (xii) remaining causes of death. For each country, annual cause-of-death counts were harmonized into consistent age groups. We used 85+ as open-ended age group for Canada and England and Wales; 90+ as open-ended age group for Northern Ireland and Scotland; 100+ as open-ended age group for Russia; and 95+ as open-ended age group for all remaining countries.

We used annual sex- and age-group-specific population exposures for the period 2015–2022 provided in the UNWPP 2024 revision. Population exposures in UNWPP are provided by single years of age (0, 1, …, 99, 100+). We aggregated the population exposures for each country to match the age groups used in the cause-of-death data. Since UNWPP does not provide separate data for England and Wales, Northern Ireland, and Scotland, we used population exposures for these three countries as provided by ONS. For Russia, we used population exposures from the Russian Fertility and Mortality Database.

We estimated annual sex-, age-group-, and cause-specific mortality rates by dividing the available cause-specific death counts by the corresponding population exposures for each country. Life expectancy at birth for each available country–sex–calendar year combination was derived from piecewise-exponential life tables constructed from these cause-specific mortality rates following standard procedures (56). In Figs. S1–S4, we compare all-cause mortality rates and life expectancies at birth based on our dataset with external sources and find generally high agreement.

To disentangle changes in life expectancy over time into age-group- and cause-specific contributions, we applied the linear integral decomposition method (57). Decomposition was performed for adjacent years (i.e. 2015–2016, 2016–2017, …, 2019–2020, 2020–2021, and, where available, 2021–2022). We compare contributions over 2019–2020, 2020–2021, and, where available, 2021–2022 with average annual contributions before the pandemic (2015–2019). In the main manuscript, we focus on total cause-of-death-specific contributions across all ages. For this purpose, we combine the categories “acute CVD” and “other CVD” into “CVD,” and combine the categories “alcohol,” “drug,” “suicide,” and “other external” into “substance and external.” We present the full decomposition results in Figs. S5 and S6. Moreover, we further explore variations in cause-of-death contributions by 10-year age groups (0–9, 10–19, …, 70–79, 80+) in Figs. S7 and S8.

## Supplementary Material

pgae508_Supplementary_Data

## Data Availability

This study uses data from the World Health Organization Mortality Database (WHO MDB), downloaded on 13 May 2024; the UNWPP, downloaded on 14 July 2024; the Russian Fertility and Mortality Database (RusFMD), downloaded on 07 July 2024; the Russian Federal State Statistics Service (Rosstat), downloaded on 07 July 2024; the United Kingdom ONS, downloaded on 23 May 2024; and the United States Centers for Disease Control and Prevention (CDC), downloaded on 14 May 2024. Data from WHO MDB are publicly available, for noncommercial purposes, from: https://www.who.int/data/data-collection-tools/who-mortality-database. Data from UNWPP are publicly available, under a Creative Commons license CC BY 3.0 IGO, from: https://population.un.org/wpp. Data from RusFMD are publicly available, for scholarly, educational, and research purposes, from: http://demogr.nes.ru. Data from Rosstat are available from: https://rosstat.gov.ru. Data from ONS are available from: https://www.ons.gov.uk. Data from CDC are publicly available, for the purpose of health statistical reporting and analysis, from: https://wonder.cdc.gov. To facilitate replication, we provide source code through our Open Science Framework (OSF) repositories: https://doi.org/10.17605/OSF.IO/86PMY and https://doi.org/10.17605/OSF.IO/89NZK.

## Previous Presentation

An earlier version of this manuscript was presented at the Population Association of America annual meeting 2023.
