# Supplementary material for: Indirect effects of the COVID-19 pandemic: A cause-of-death analysis of life expectancy changes in 24 countries, 2015 to 2022
Source: PNAS Nexus. 2024 Dec 19;4(1):pgae508. doi: 10.1093/pnasnexus/pgae508 (PMC11664260; doi:10.1093/pnasnexus/pgae508)

INDIRECT EFFECTS OF THE COVID-19 PANDEMIC: A CAUSE-OF-DEATH ANALYSIS OF LIFE EXPECTANCY CHANGES IN 24 COUNTRIES, 2015 TO 2022

This file contains:

- **Supplementary Text**
- **Table S1.** Cause-of-death coding.
- **Figure S1.** Comparison of female and male age-group-specific logged mortality rates based on author data set vs. United Nations World Population Prospects 2024 revision. *Note*: Comparisons only shown for country–years with available data from both sources. Age groups may differ across countries and sources.
- **Figure S2.** Comparison of female and male age-group-specific logged mortality rates based on author data set vs. Human Lifetable Database. *Note*: Comparisons only shown for country–years with available data from both sources. Age groups may differ across countries and sources. Comparisons for Australia, Bulgaria, Canada, and Denmark are based on pooled data from two or three calendar years.
- **Figure S3.** Comparison of female and male age-group-specific logged mortality rates based on author data set vs. Human Mortality Database. *Note*: Comparisons only shown for country–years with available data from both sources. Age groups may differ across countries and sources.
- **Figure S4.** Comparison of female and male life expectancy at birth based on author data set vs. United Nations World Population Prospects 2024 revision. *Note*: Comparisons only shown for country–years with available data from both sources.
- **Figure S5.** Cause-of-death-specific contributions to changes in female life expectancy at birth by country and period. *Note:* Contributions in months. Tiles for period 2015–2019 show average annual contributions. Cause-of-death data for period 2021–2022 only available for: Australia, Canada, England and Wales, Hungary, Lithuania, the Netherlands, Sweden, and the United States.
- **Figure S6.** Cause-of-death-specific contributions to changes in male life expectancy at birth by country and period. *Note:* Contributions in months. Tiles for period 2015–2019 show average annual contributions. Cause-of-death data for period 2021–2022 only available for: Australia, Canada, England and Wales, Hungary, Lithuania, the Netherlands, Sweden, and the United States.
- **Figure S7.** Age-group- and cause-of-death-specific contributions to changes in female life expectancy at birth by country and period. *Note:* Contributions in months. Bars for period 2015–2019 show average annual contributions. Cause-of-death data for period 2021–2022 only available for: Australia, Canada, England and Wales, Hungary, Lithuania, the Netherlands, Sweden, and the United States.
- **Figure S8.** Age-group- and cause-of-death-specific contributions to changes in male life expectancy at birth by country and period. *Note*: Contributions in months. Bars for period 2015–2019 show average annual contributions. Cause-of-death data for period 2021–2022 only available for: Australia, Canada, England and Wales, Hungary, Lithuania, the Netherlands, Sweden, and the United States.

Supplementary Text

Following the data gathering process described in the main manuscript, cause-of-death information with 4-character ICD-10 codes was available through at least 2021 for a total of 53 countries or regions, including the 24 countries listed in the main manuscript (Australia, Austria, Brazil, Bulgaria, Canada, Chile, Croatia, Czechia, Denmark, England and Wales, Hungary, Japan, Latvia, Lithuania, the Netherlands, Northern Ireland, Poland, Russia, Scotland, South Korea, Spain, Sweden, Switzerland, the United States), as well as Antigua and Barbuda, Argentina, Colombia, Cuba, Cyprus, Ecuador, Georgia, Grenada, Guatemala, Hong Kong SAR, Israel, Kazakhstan, Lebanon, Luxembourg, Mauritius, Mexico, Micronesia (Federated States of), Mongolia, Montserrat, Nicaragua, Oman, Panama, Paraguay, Puerto Rico, Saint Vincent and Grenadines, Saudi Arabia, Singapore, Tunisia, Virgin Islands (USA).

Our focus was on European countries, and we included countries where data quality was not a concern, excluding Luxembourg, due to its small population, and Cyprus, due to its comparatively low level of death registration (1). Additionally, we included Canada and the United States of America (North America), Brazil and Chile (South America), Japan, Russia, and South Korea (Asia), as well as Australia (Oceania) as comparison countries. Most of these countries have received much attention during the COVID-19 pandemic due to their successful (Australia, Japan, South Korea) or less successful (Brazil, Russia, USA) pandemic responses. In addition, death registration in these countries is very high (1).

In a second step, we explored the plausibility of our cause-of-death data set. To this end, we visually compared all-cause mortality rates constructed from our data set with: (a) abridged life tables provided in the United Nations World Population Prospects (UNWPP) 2024 revision (2), as published in the database; (b) abridged life tables provided in the Human Lifetable Database (3), as published in the database; (c) abridged life tables provided in the Human Mortality Database (4), as published in the database. Data for England and Wales, Northern Ireland, and Scotland were not available in UNWPP. Data for Chile, Croatia, England and Wales, Lithuania, Northern Ireland, and Scotland were not available in the HLD or not available for the period 2015–2022. For the remaining country–years, we used HLD data for comparison where available. For Australia, Bulgaria, Canada, and Denmark, HLD life tables were only available for periods spanning two or three calendar years. To enable comparison for these countries, we aggregated our cause-of-death data to match the HLD data. Data for

Brazil and Russia were not available in the HMD or not available for the period 2015–2022. For the remaining country–years, we used HMD data for comparison where available.

We noticed few large deviations of our all-cause mortality rates from those in UNWPP, the HMD, or the HLD, including: (a) Australia in 2022 (UNWPP only); (b) Brazil in 2021 (HLD only); and (c) South Korea (UNWPP only). Based on our comparisons, we concluded that: (a) the mortality age profile for Australia in 2022 in UNWPP is inconsistent with previous years and potentially affected by an estimation error; (b) HLD life tables for Brazil in 2021 potentially underestimate mortality; (c) UNWPP consistently underestimates young-age mortality in South Korea in the pre-pandemic period. We therefore proceeded with our analysis without further adjustment of our cause-of-death-specific mortality rates.

References

1. A. Karlinsky, International completeness of death registration. *Demographic Research* **50**, 1151–1170 (2024).

2. United Nations, Department of Economic and Social Affairs, Population Division, World Population Prospects 2024. *https://population.un.org/wpp/* (2024).

3. HLD, Human Lifetable Database. Max Planck Institute for Demographic Research (Germany), University of California, Berkeley (USA), and French Institute for Demographic Studies (France). Available at *www.lifetable.de* (Downloaded 22 July 2024). Deposited 2024.

4. HMD, Human Mortality Database. Max Planck Institute for Demographic Research (Germany), University of California, Berkeley (USA), and French Institute for Demographic Studies (France). Available at *www.mortality.org* (Downloaded 23 July 2024). Deposited 2024.

Table S1. Cause-of-death coding.

	Cause of death	ICD-10 codes
**1**	Acute CVD (acute IHD and strokes)	I20-I24, I60-I64
**2**	Other CVD	The rest of I, excluding I42.6
**3**	Acute respiratory diseases	J00-J22, U04
**4**	Chronic obstructive pulmonary disease (COPD)	J40-J47
**5**	Certain infectious diseases	A00-B99
**6**	Suicides	X60–X84, Y87.0
**7**	Drug-related deaths	F11–F19, X40–X44, X85, Y10–Y14
**8**	Alcohol-related deaths	F10, I42.6, K70, K74, X45, Y15
**9**	Cancer (or Neoplasms)	C00-D48
**10**	Other external causes of death	V, W, and the rest of X and Y
**11**	COVID-19	U07.1, U07.2
**12**	Residual	All else

Figure S1a

Comparison of age-group-specific logged mortality rates (Australia), author data set (solid line) vs. UNWPP (dashed line)

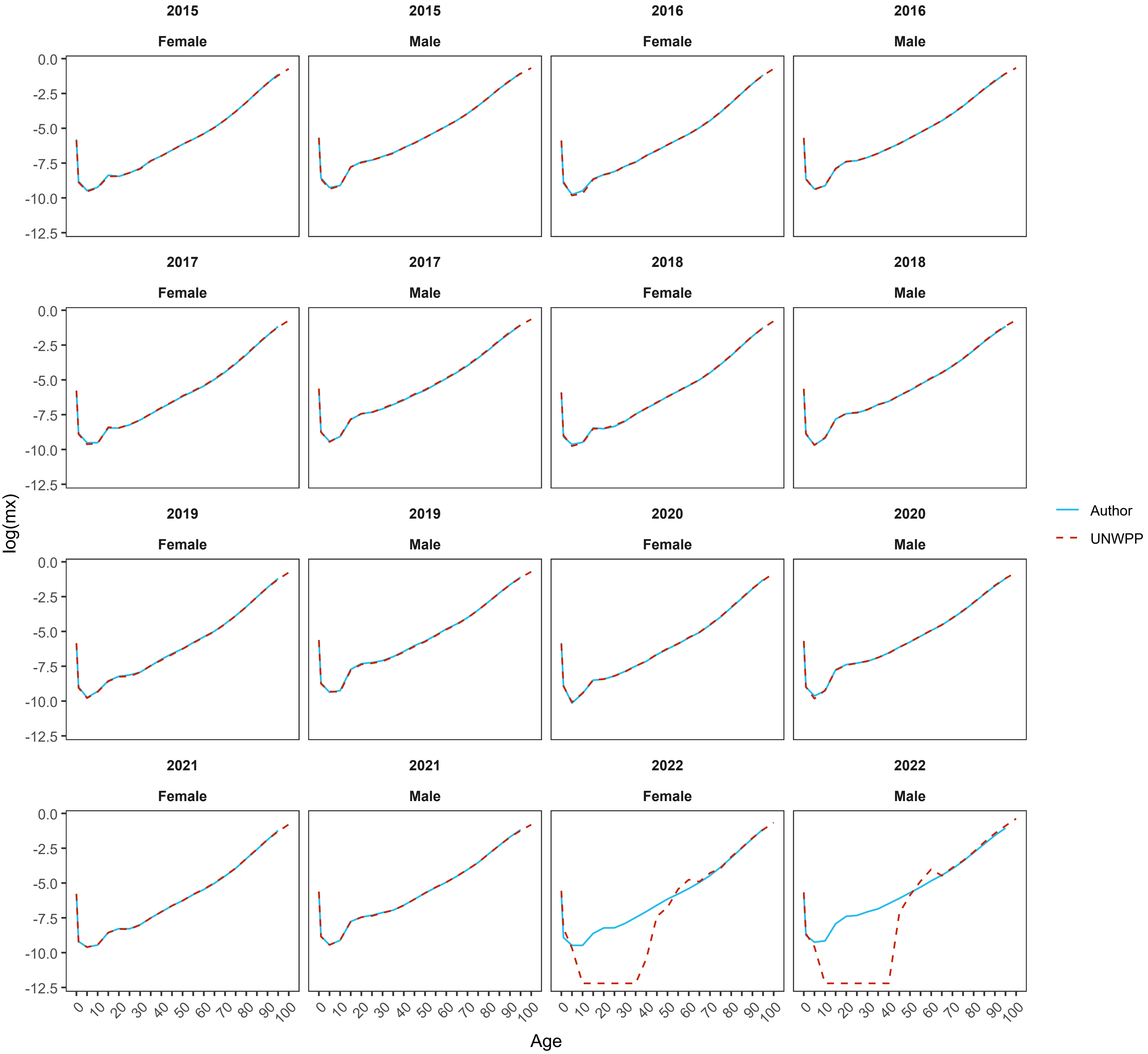

Figure S1b

Comparison of age-group-specific logged mortality rates (Austria), author data set (solid line) vs. UNWPP (dashed line)

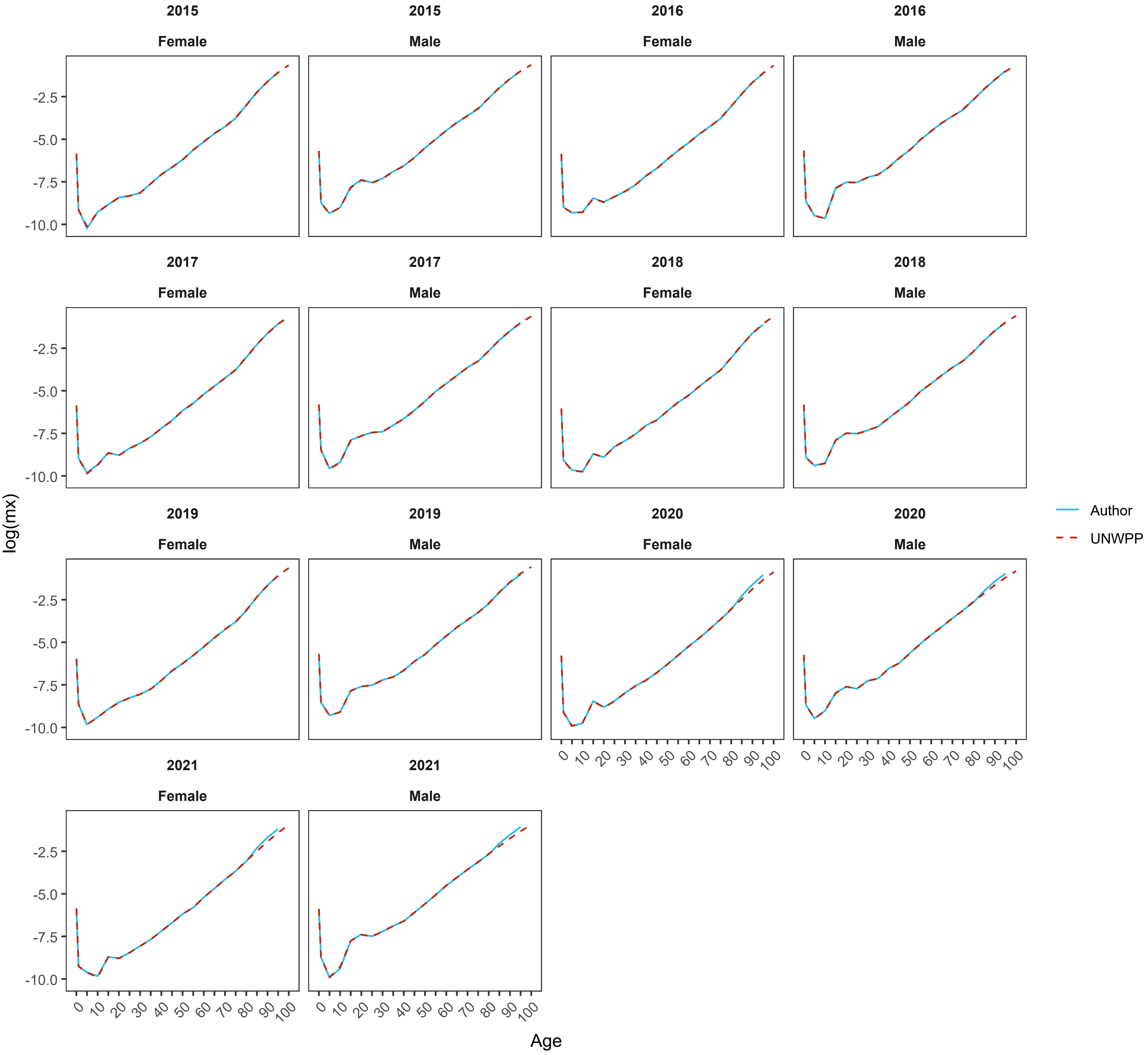

Figure S1c

Comparison of age-group-specific logged mortality rates (Brazil), author data set (solid line) vs. UNWPP (dashed line)

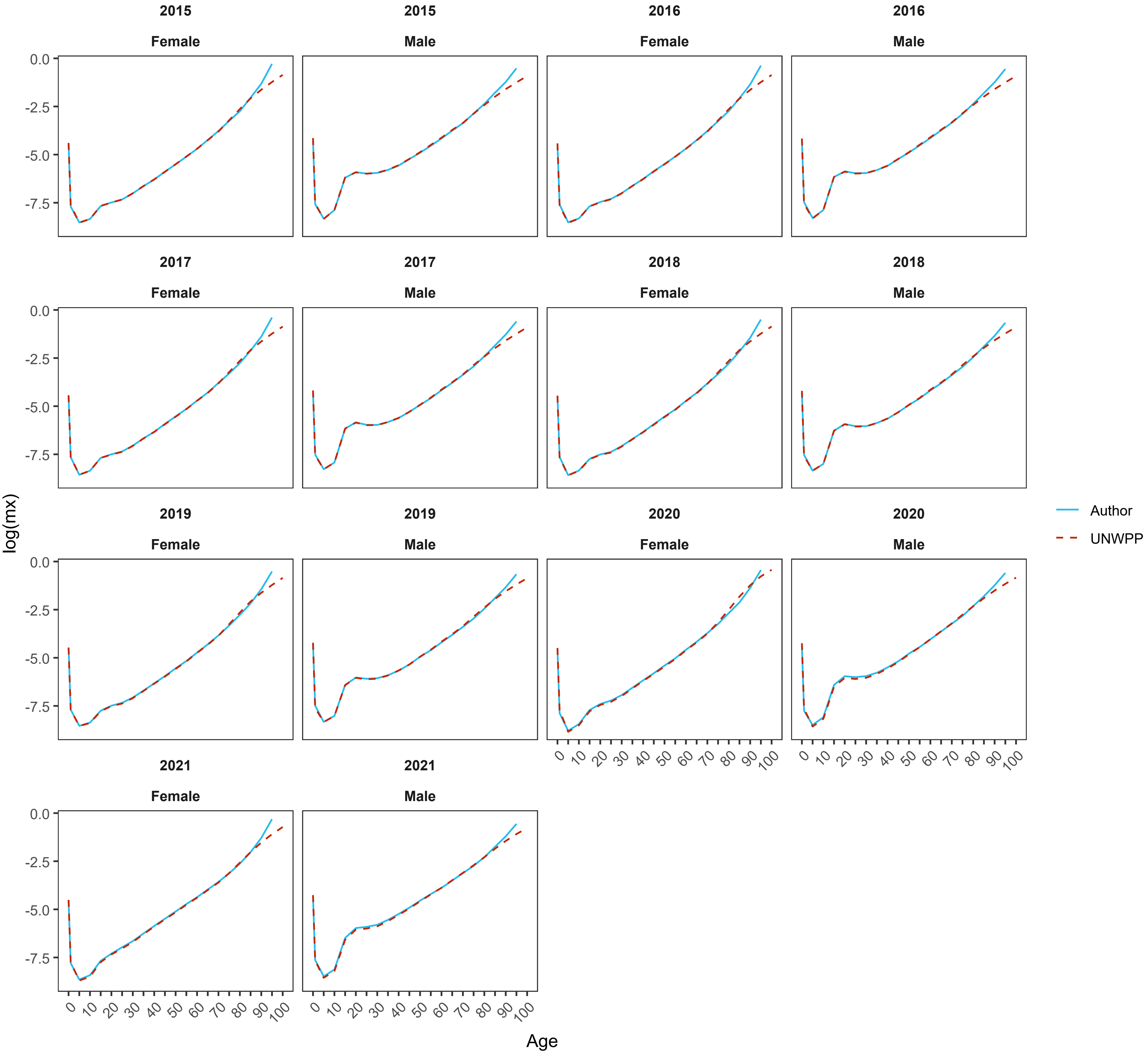

Figure S1d

Comparison of age-group-specific logged mortality rates (Bulgaria), author data set (solid line) vs. UNWPP (dashed line)

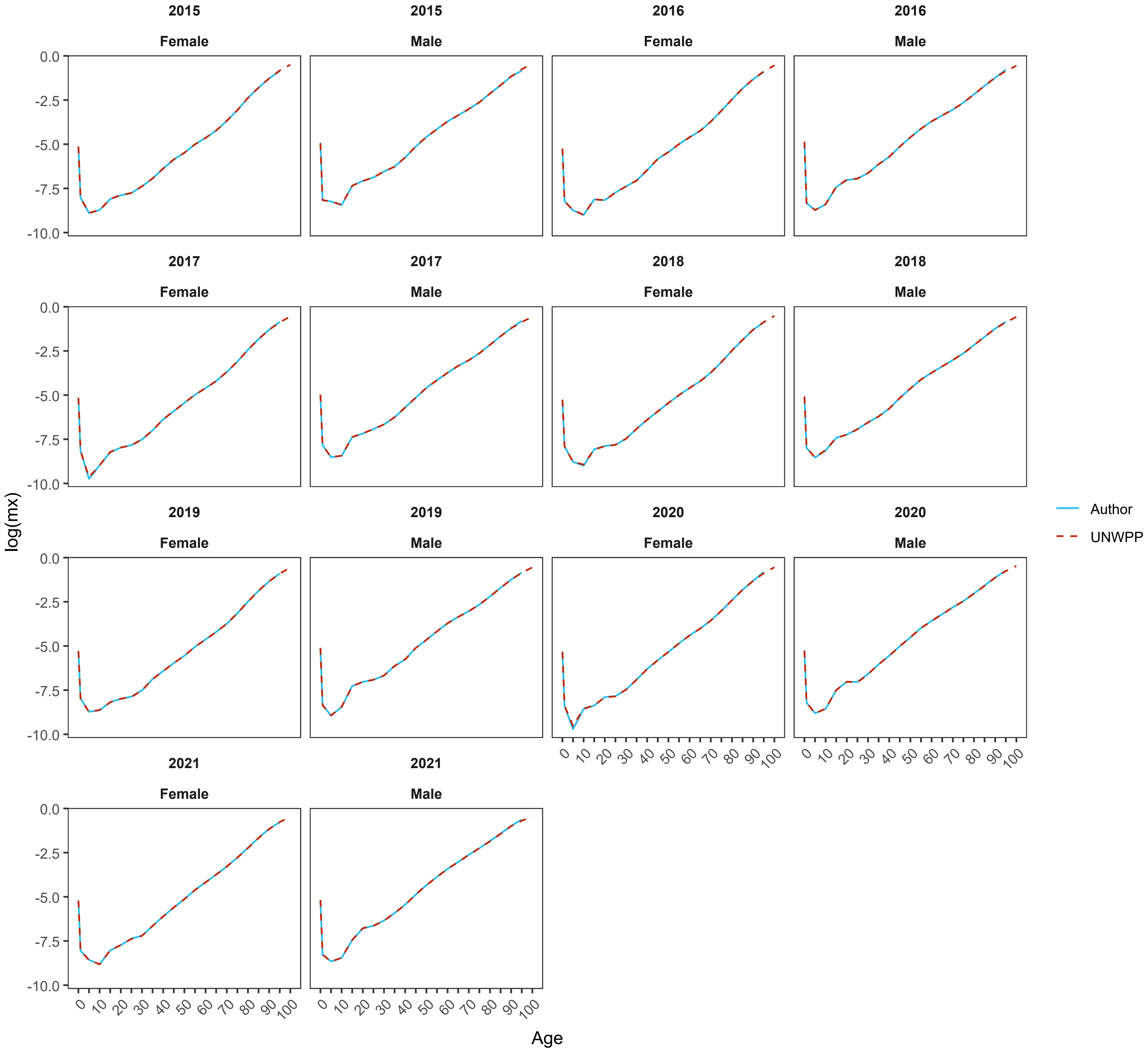

Figure S1e

Comparison of age-group-specific logged mortality rates (Canada),
author data set (solid line) vs. UNWPP (dashed line)

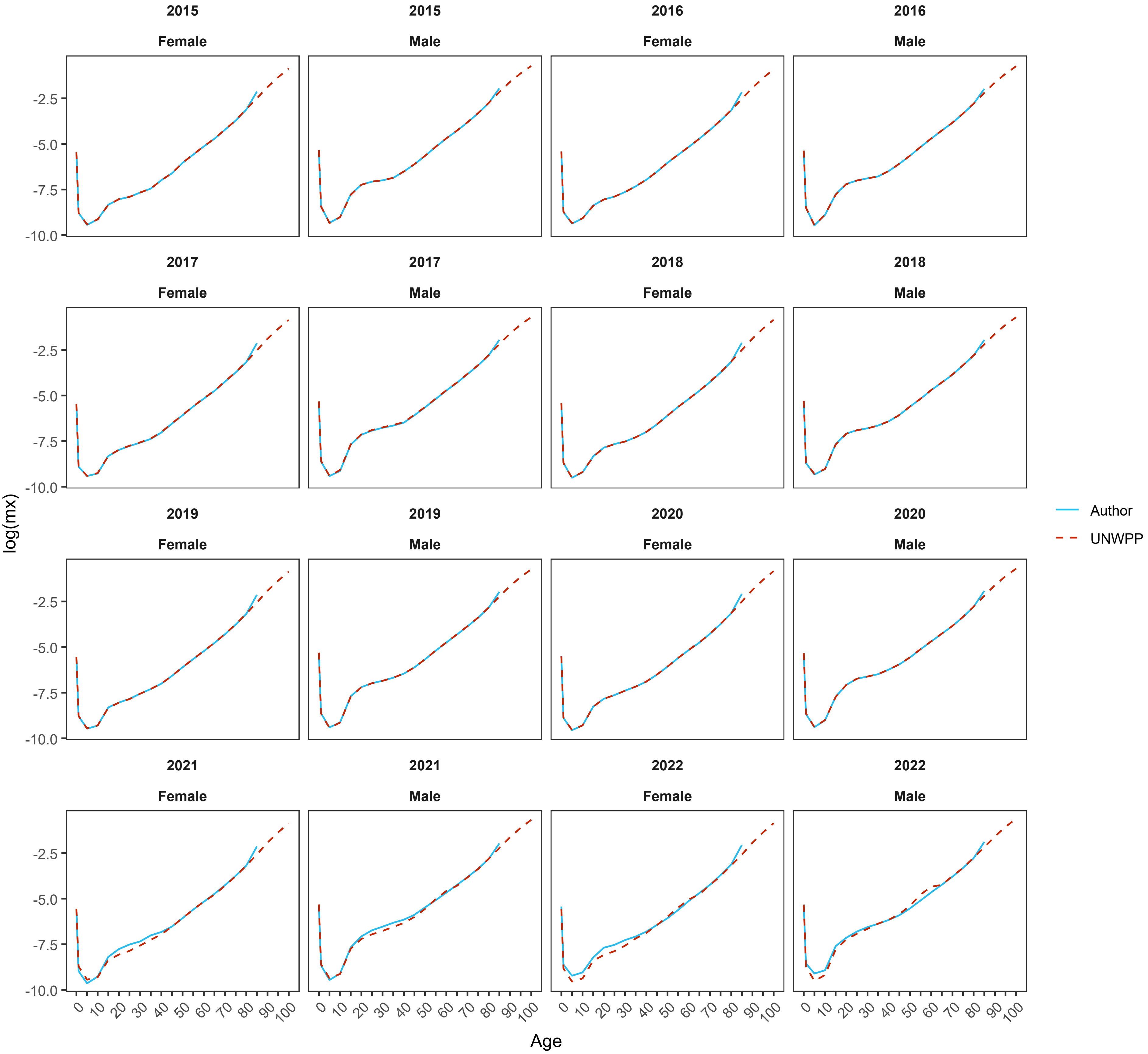

Figure S1f

Comparison of age-group-specific logged mortality rates (Chile), author data set (solid line) vs. UNWPP (dashed line)

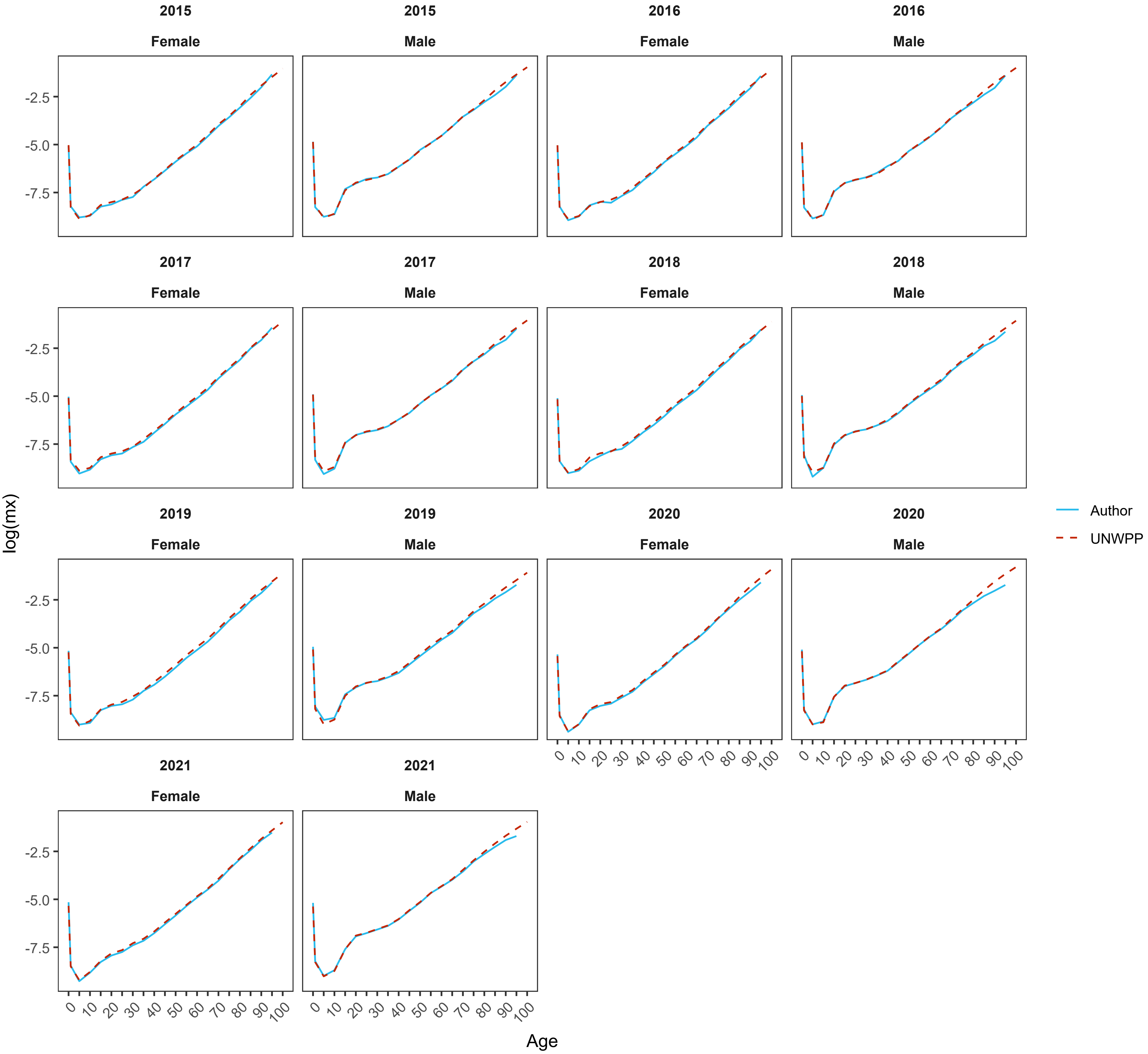

Figure S1g

Comparison of age-group-specific logged mortality rates (Croatia), author data set (solid line) vs. UNWPP (dashed line)

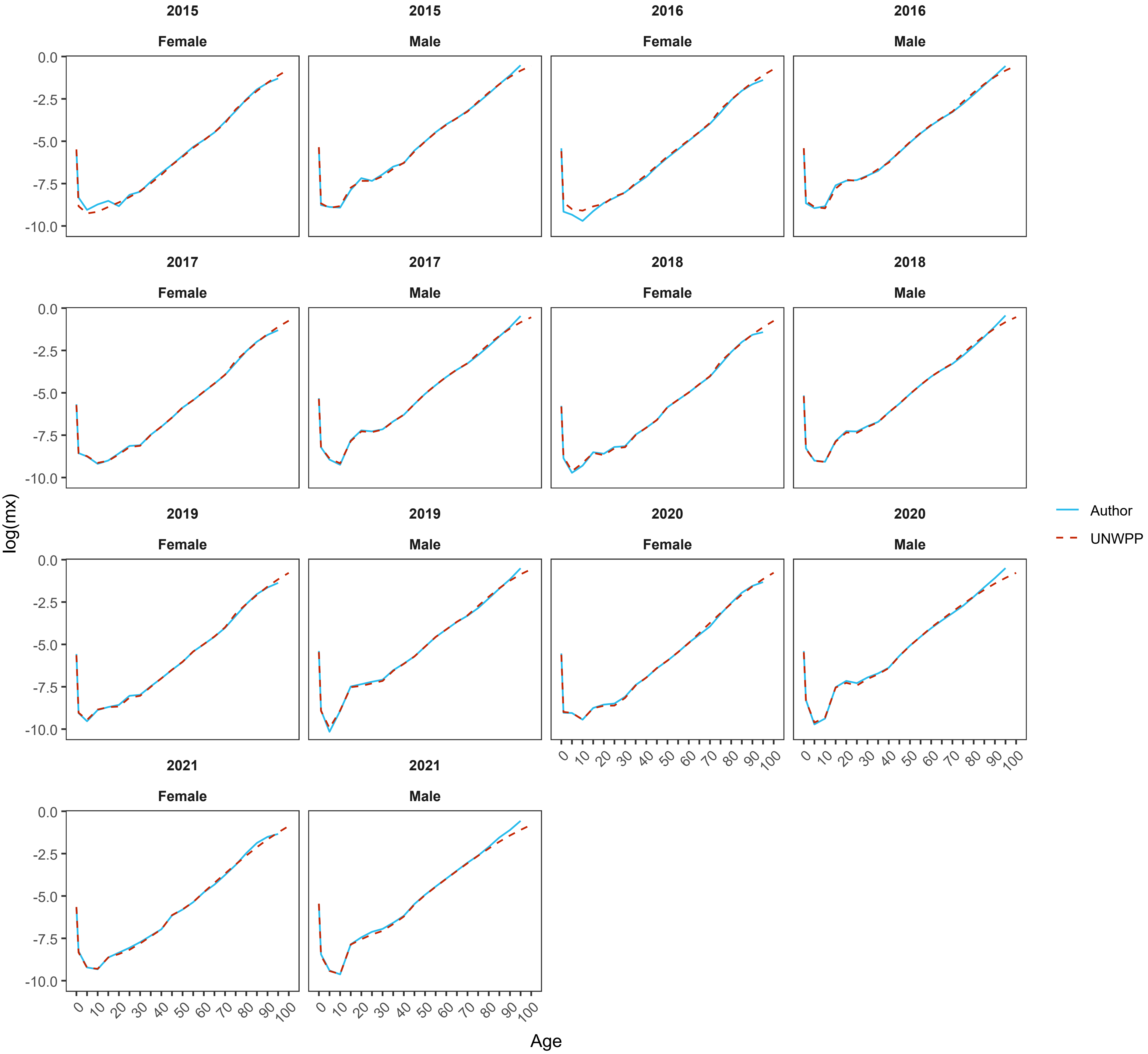

Figure S1h

Comparison of age-group-specific logged mortality rates (Czechia), author data set (solid line) vs. UNWPP (dashed line)

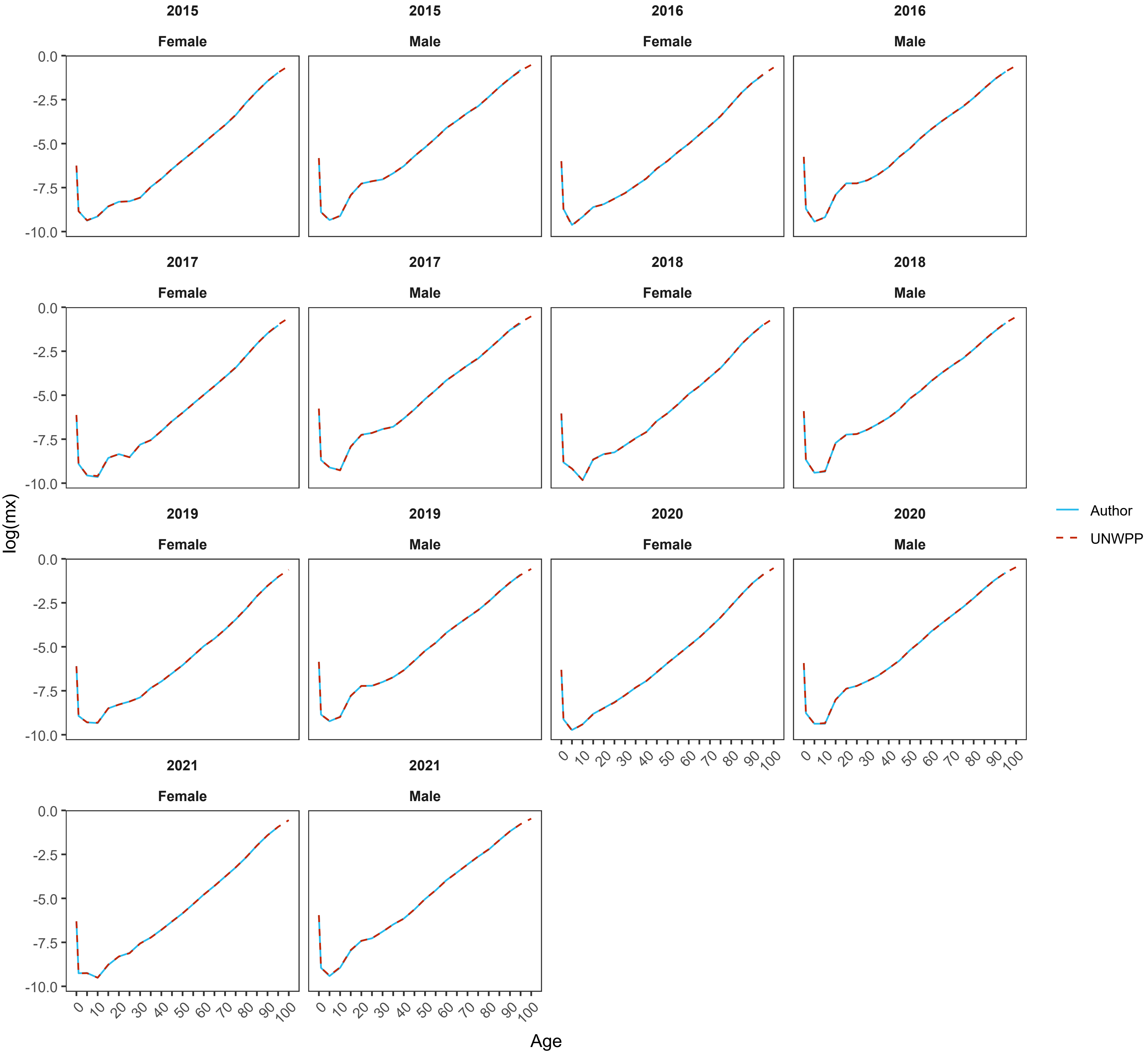

Figure S1i

Comparison of age-group-specific logged mortality rates (Denmark), author data set (solid line) vs. UNWPP (dashed line)

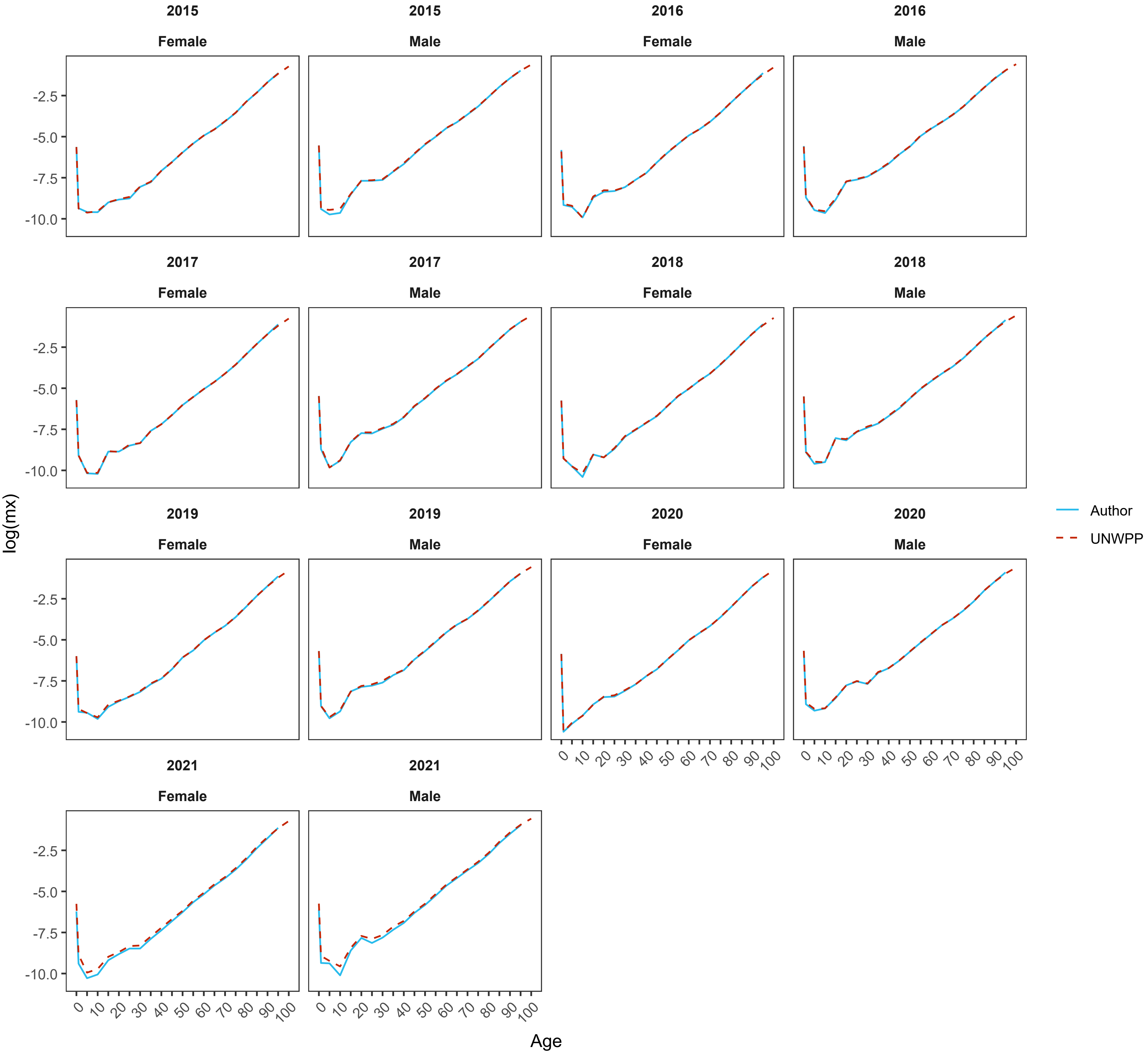

Figure S1j

Comparison of age-group-specific logged mortality rates (Hungary), author data set (solid line) vs. UNWPP (dashed line)

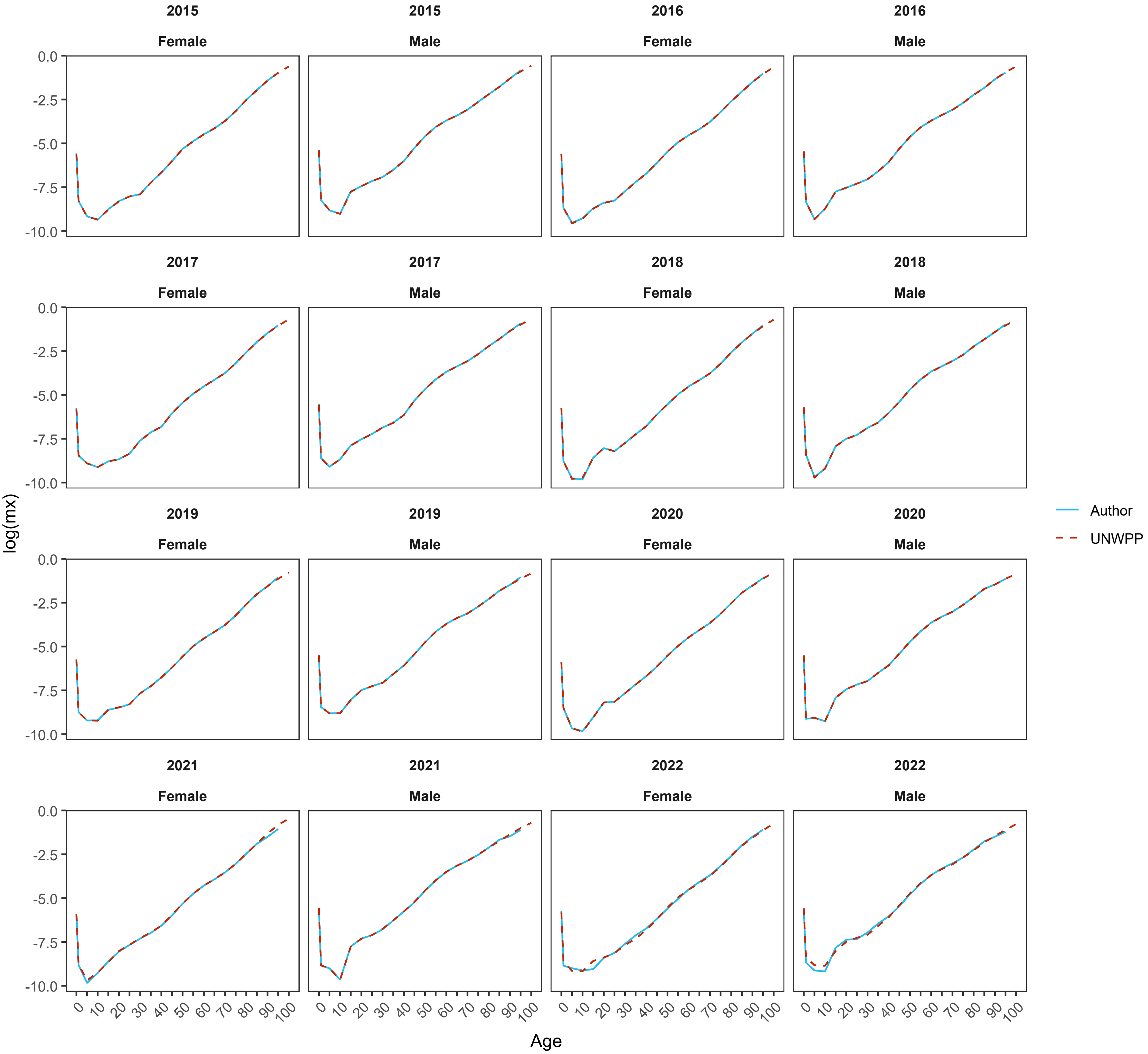

Figure S1k

Comparison of age-group-specific logged mortality rates (Japan),
author data set (solid line) vs. UNWPP (dashed line)

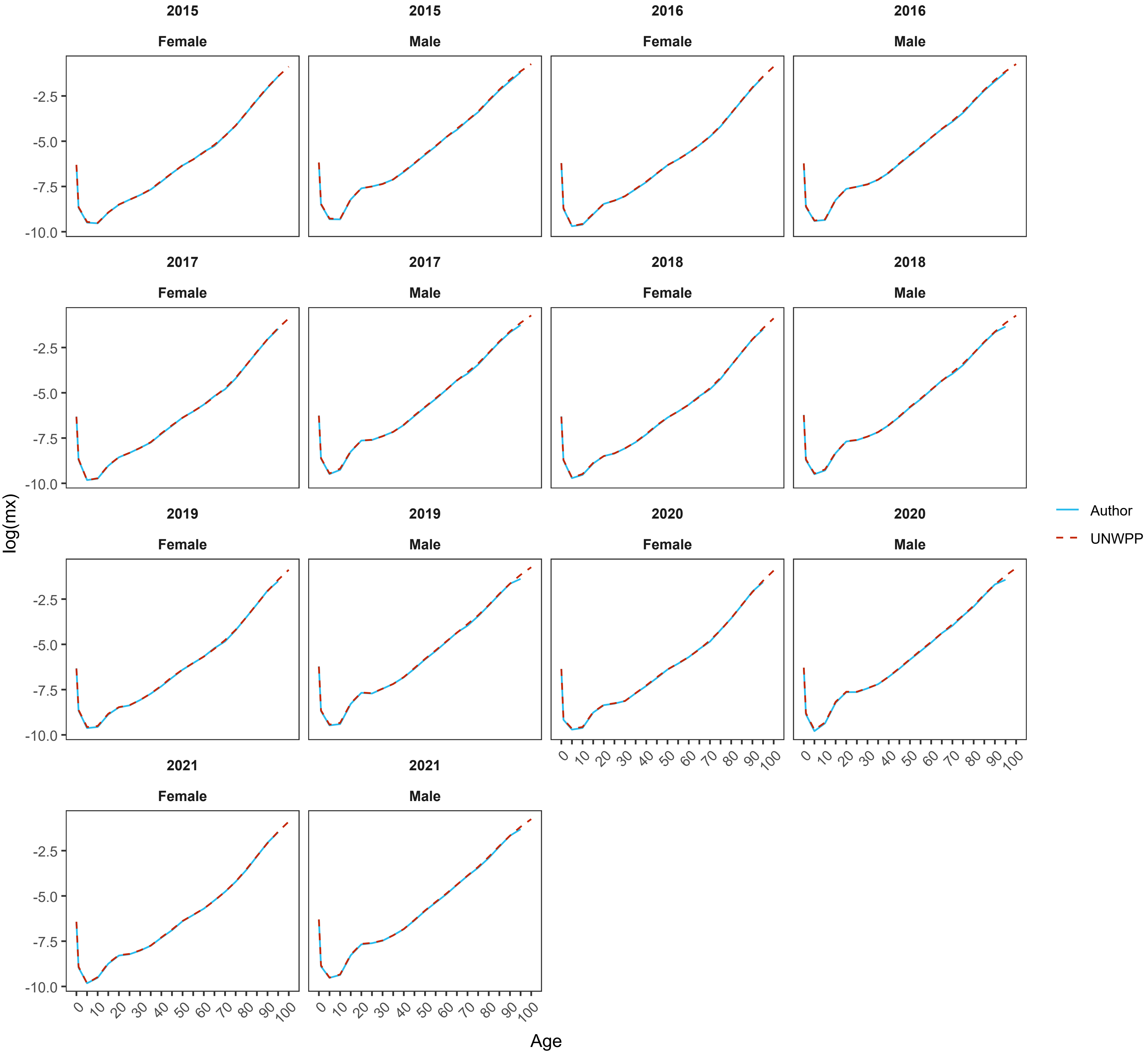

Figure S1l

Comparison of age-group-specific logged mortality rates (Latvia), author data set (solid line) vs. UNWPP (dashed line)

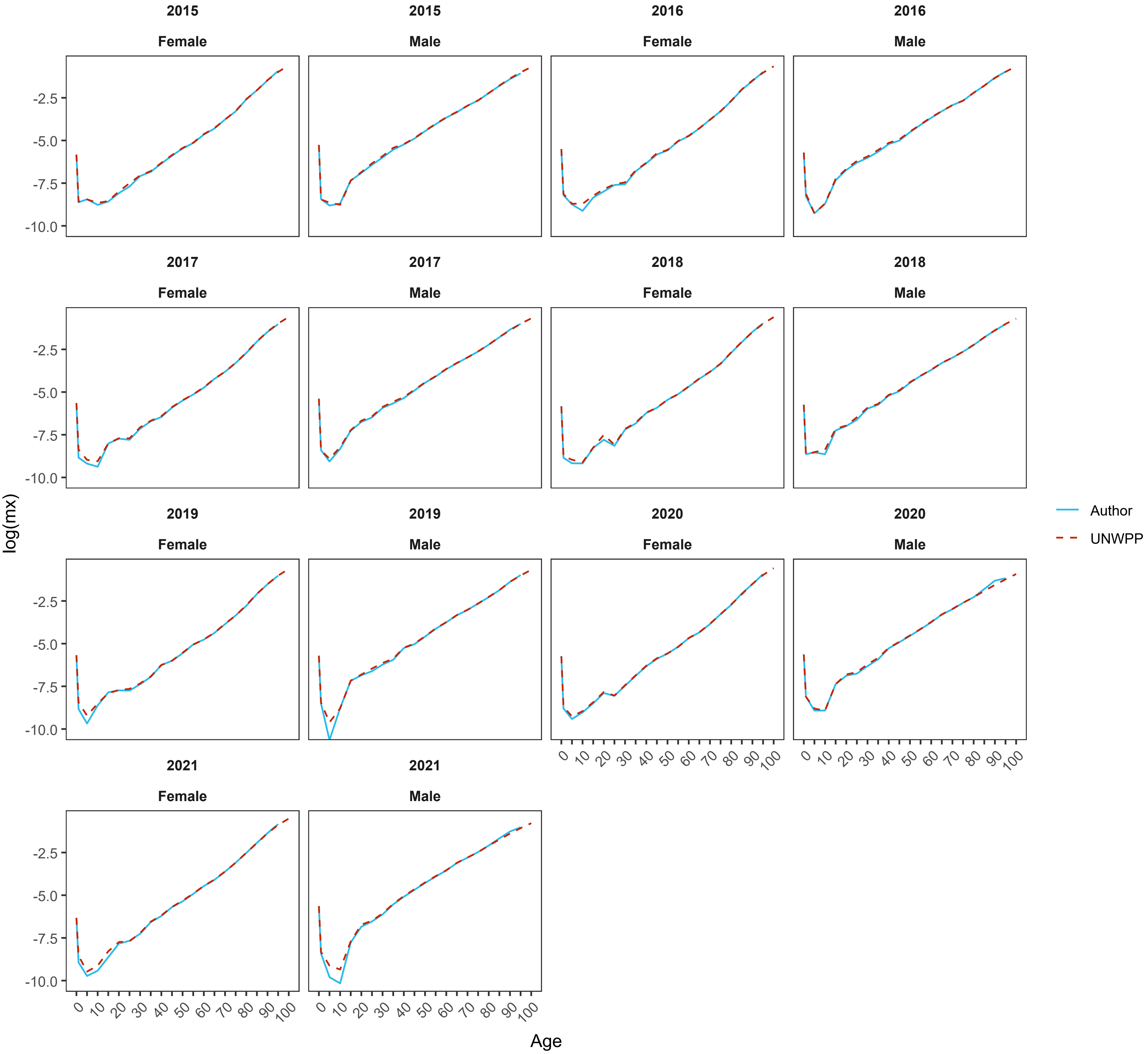

Figure S1m

Comparison of age-group-specific logged mortality rates (Lithuania), author data set (solid line) vs. UNWPP (dashed line)

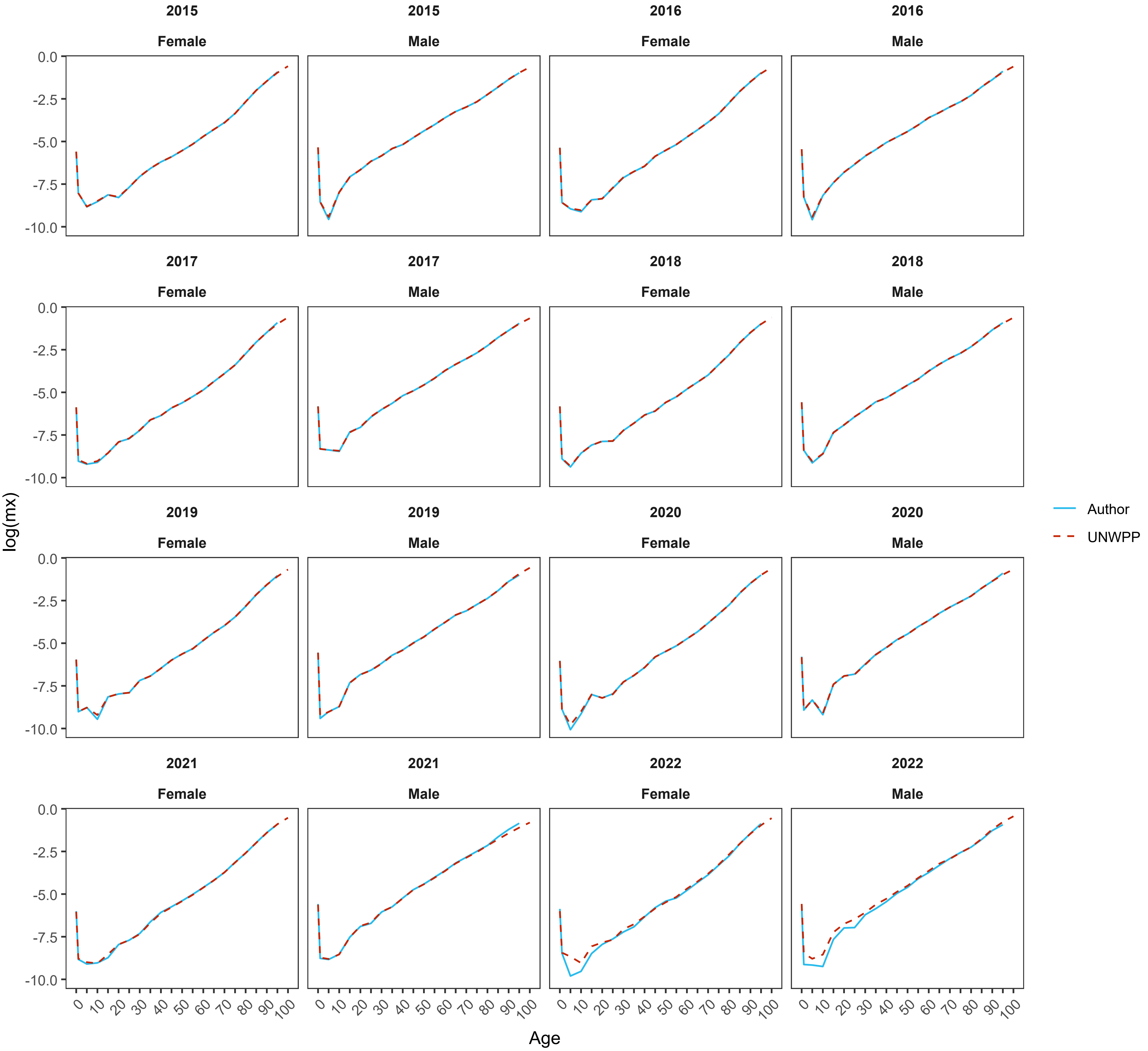

Figure S1n

Comparison of age-group-specific logged mortality rates (Netherlands), author data set (solid line) vs. UNWPP (dashed line)

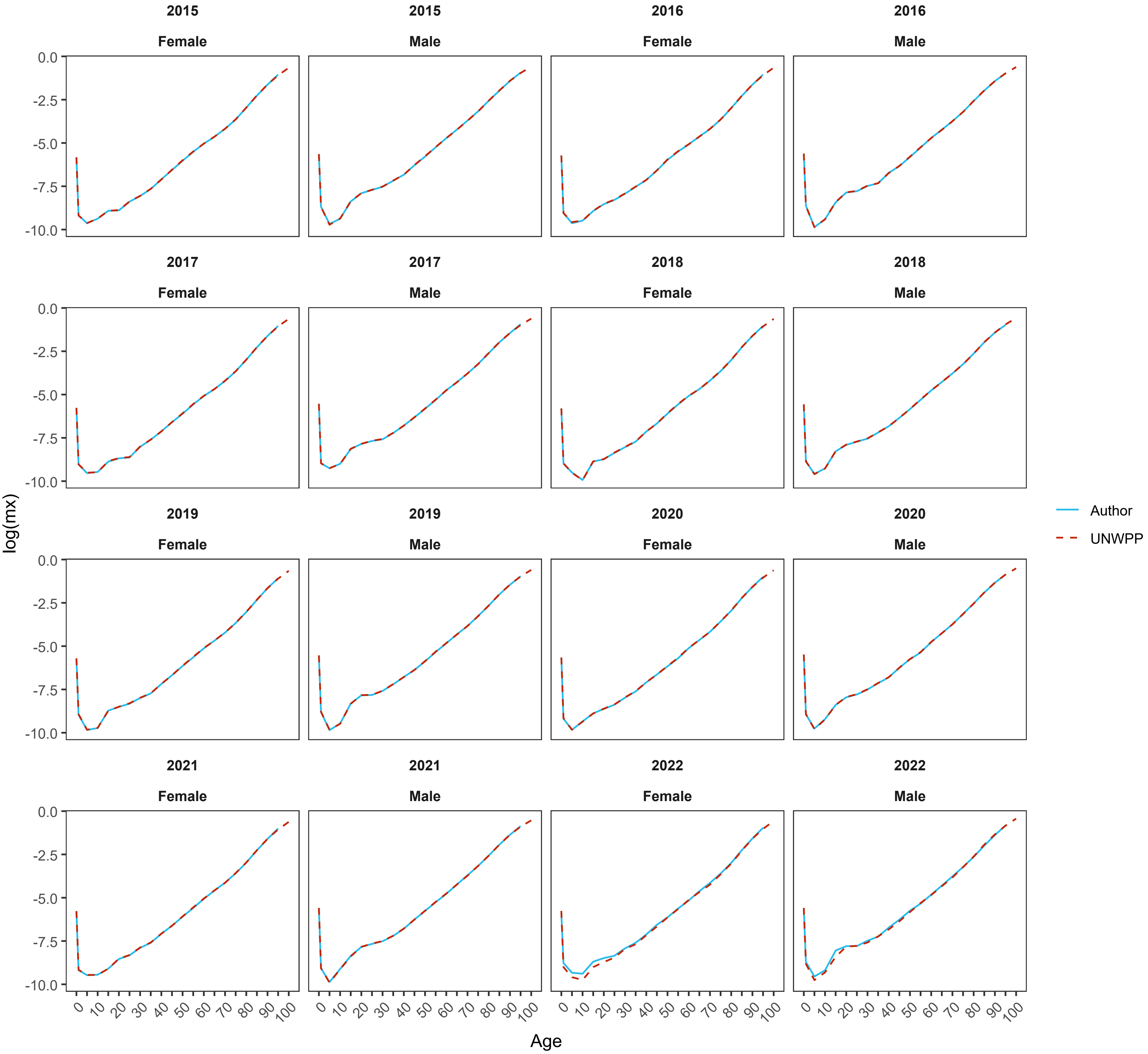

Figure S1o

Comparison of age-group-specific logged mortality rates (Poland), author data set (solid line) vs. UNWPP (dashed line)

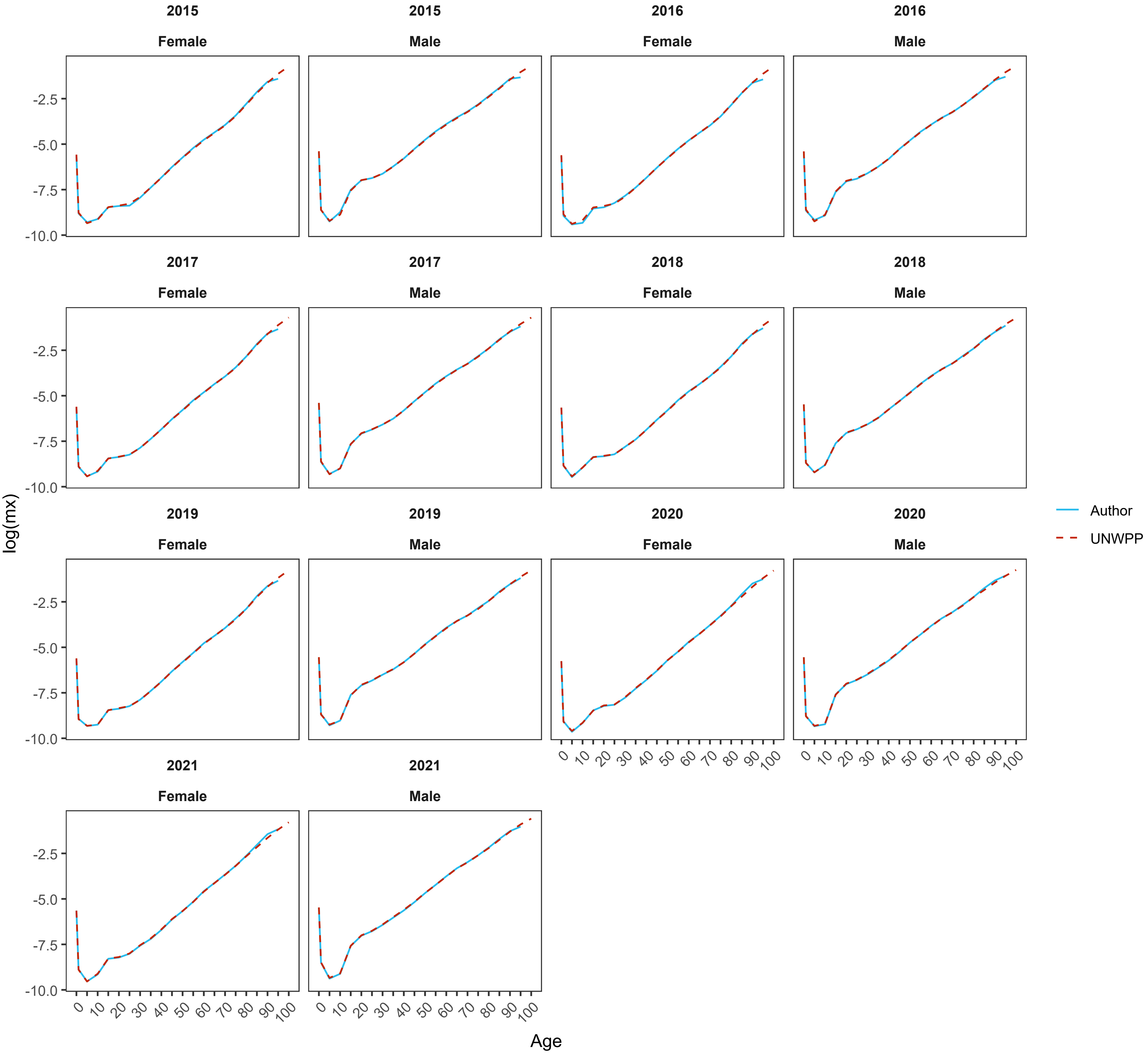

Figure S1p

Comparison of age-group-specific logged mortality rates (Russia), author data set (solid line) vs. UNWPP (dashed line)

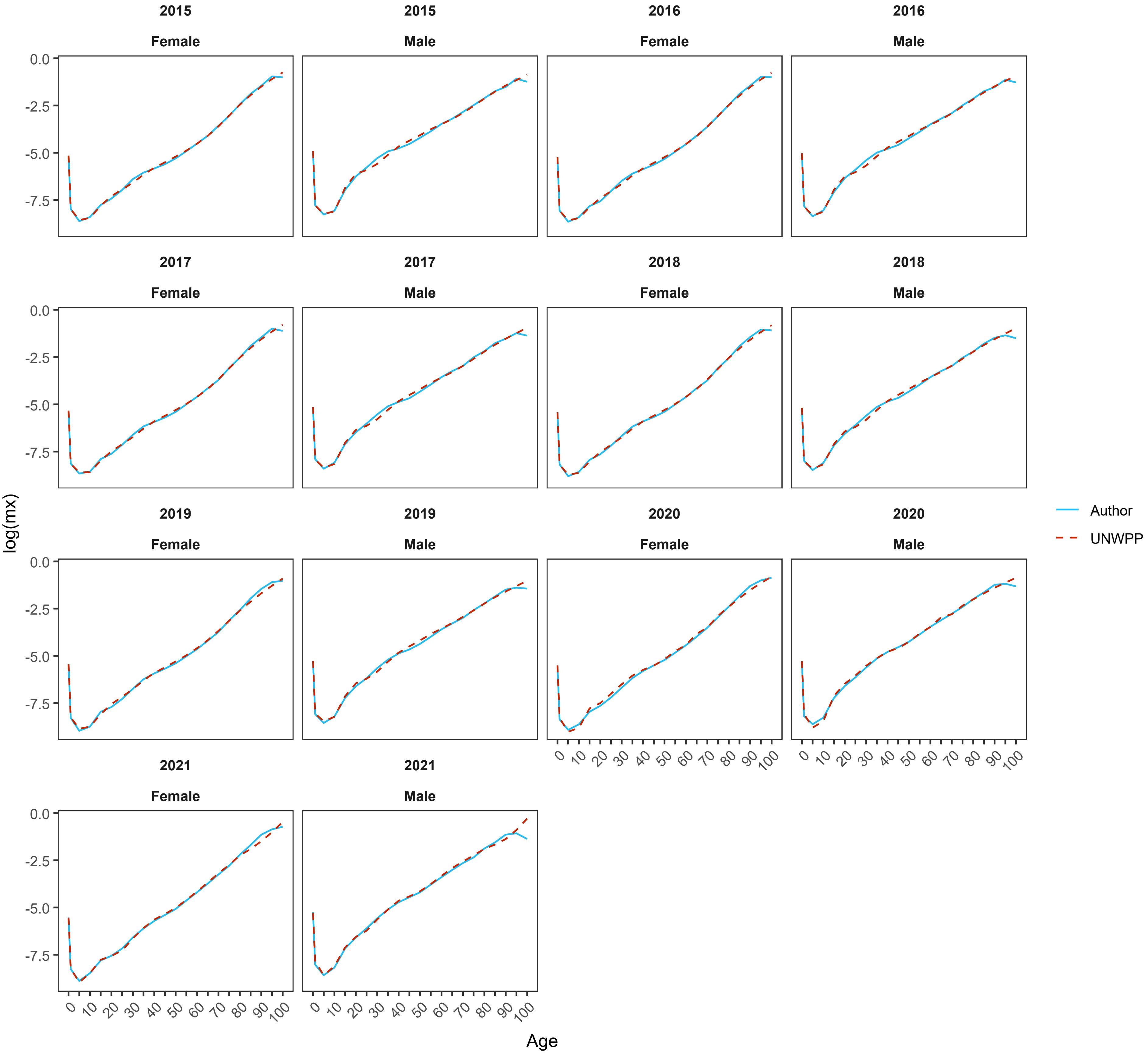

Figure S1q

Comparison of age-group-specific logged mortality rates (South Korea), author data set (solid line) vs. UNWPP (dashed line)

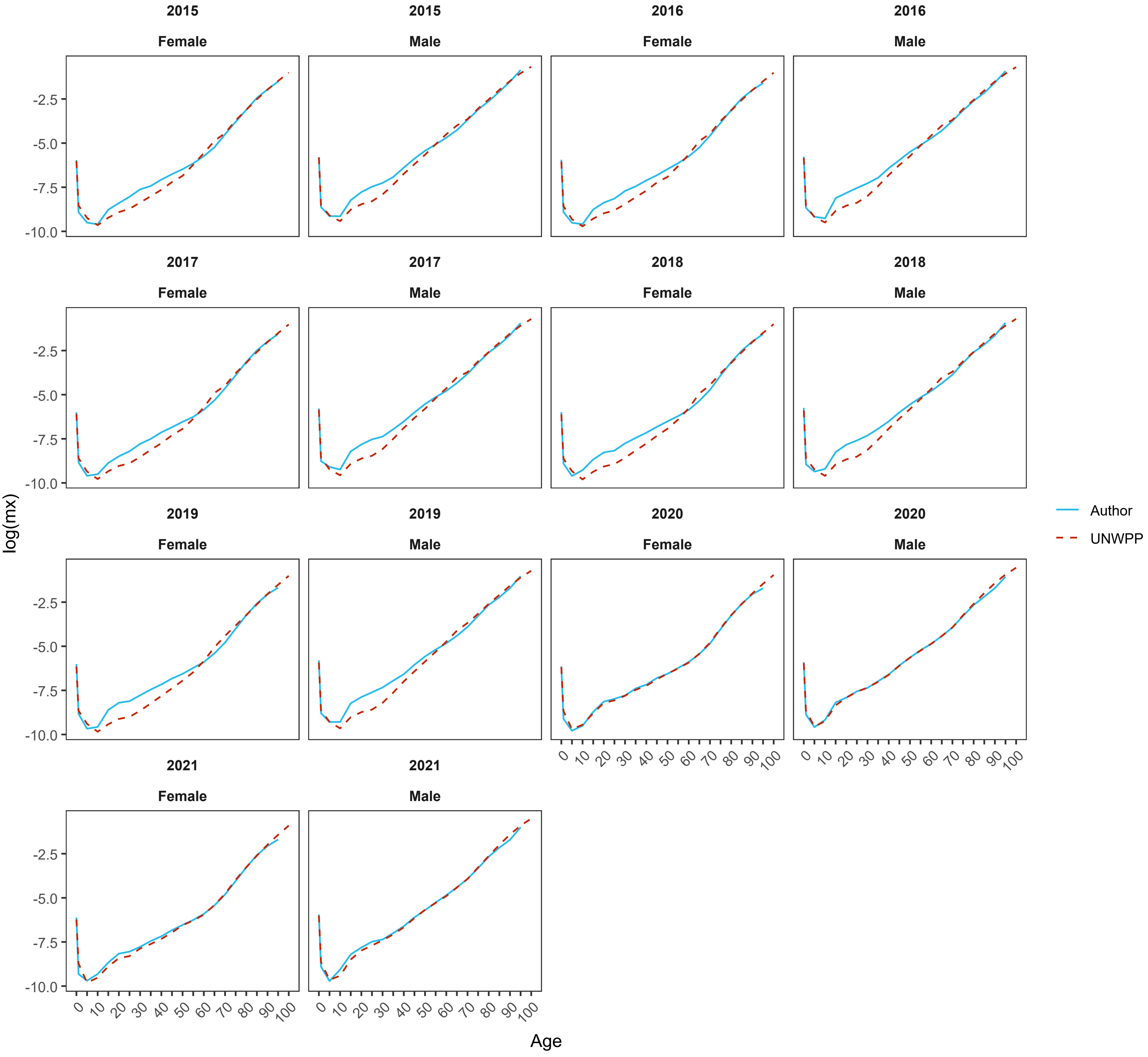

Figure S1r

Comparison of age-group-specific logged mortality rates (Spain),
author data set (solid line) vs. UNWPP (dashed line)

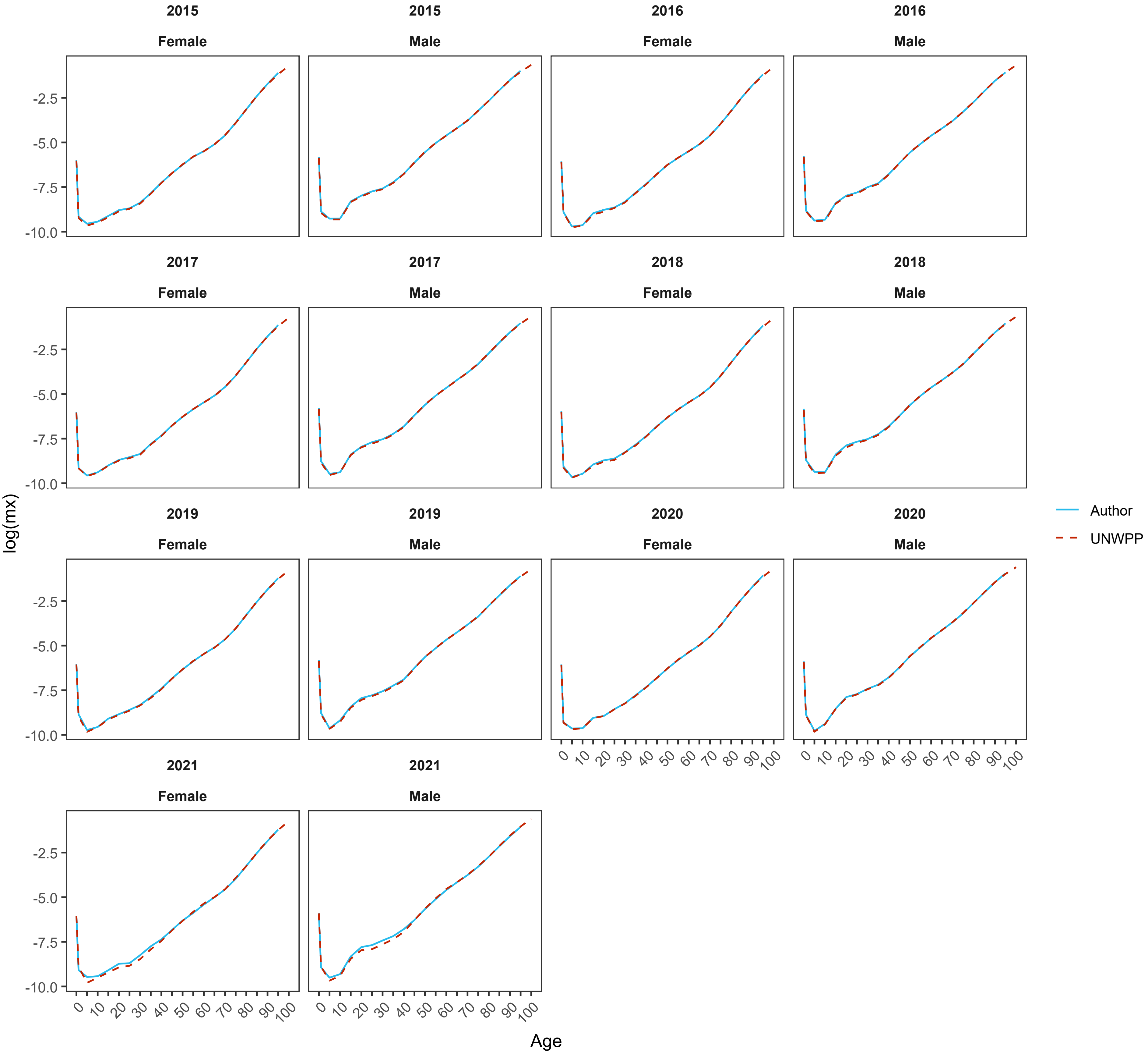

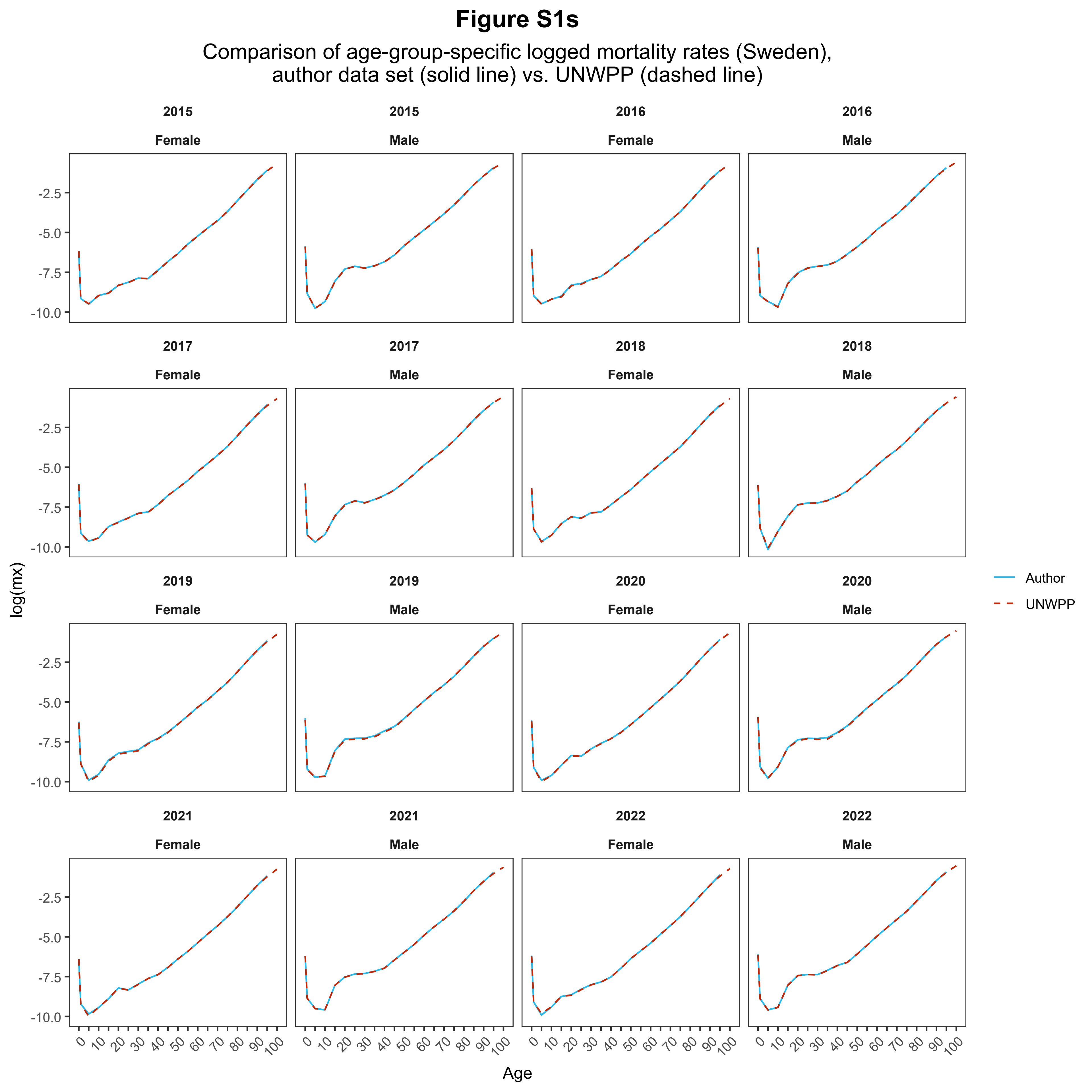
Figure S1s
Comparison of age-group-specific logged mortality rates (Sweden),
author data set (solid line) vs. UNWPP (dashed line)
2015 Female
2015 Male
2016 Female
2016 Male
2017 Female
2017 Male
2018 Female
2018 Male
2019 Female
2019 Male
2020 Female
2020 Male
2021 Female
2021 Male
2022 Female
2022 Male
-2.5
-5.0
-7.5
-10.0
log(mx)
0 10 20 30 40 50 60 70 80 90 100
Age
Author
UNWPP

Figure S1t

Comparison of age-group-specific logged mortality rates (Switzerland), author data set (solid line) vs. UNWPP (dashed line)

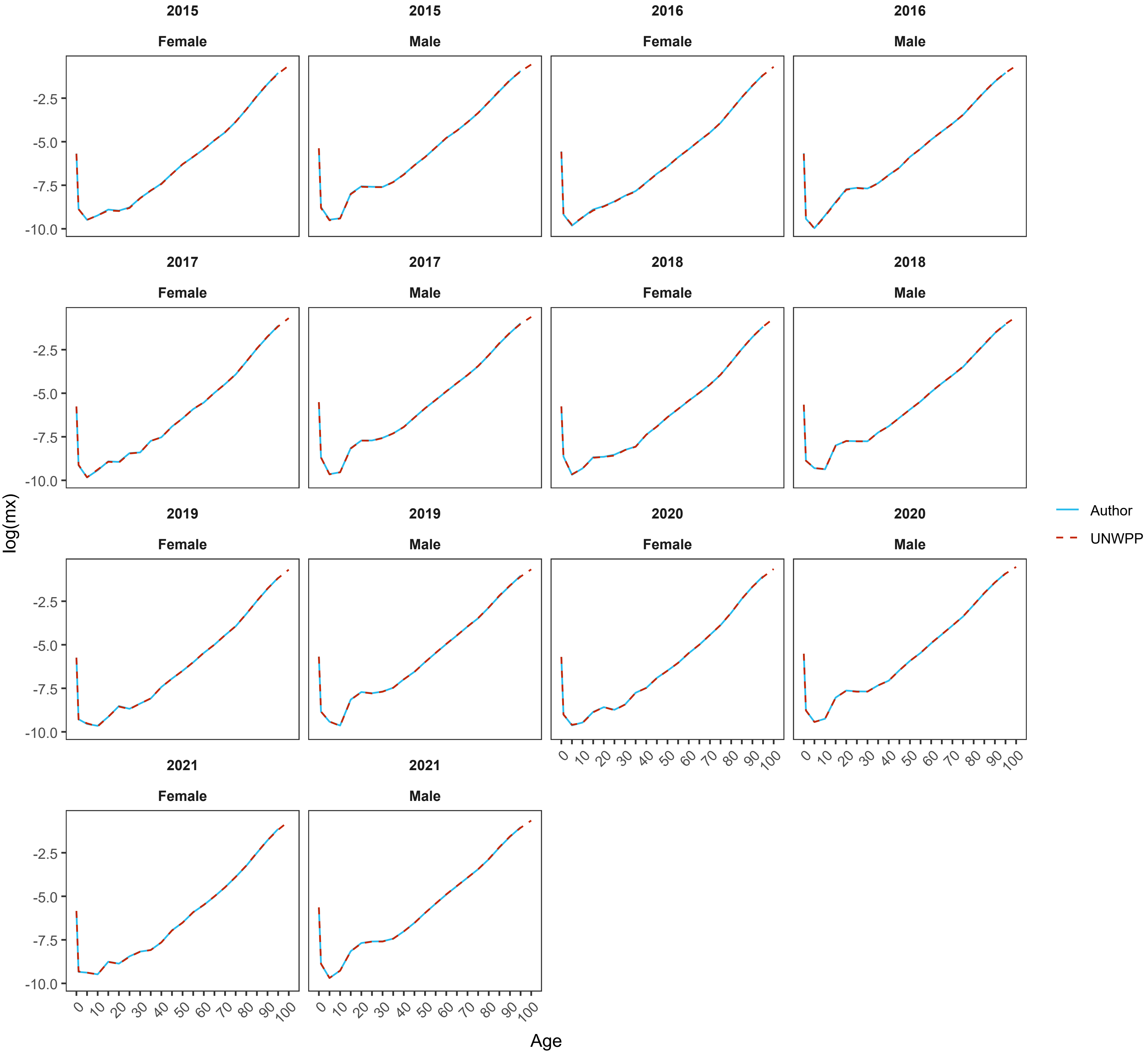

Figure S1u

Comparison of age-group-specific logged mortality rates (USA), author data set (solid line) vs. UNWPP (dashed line)

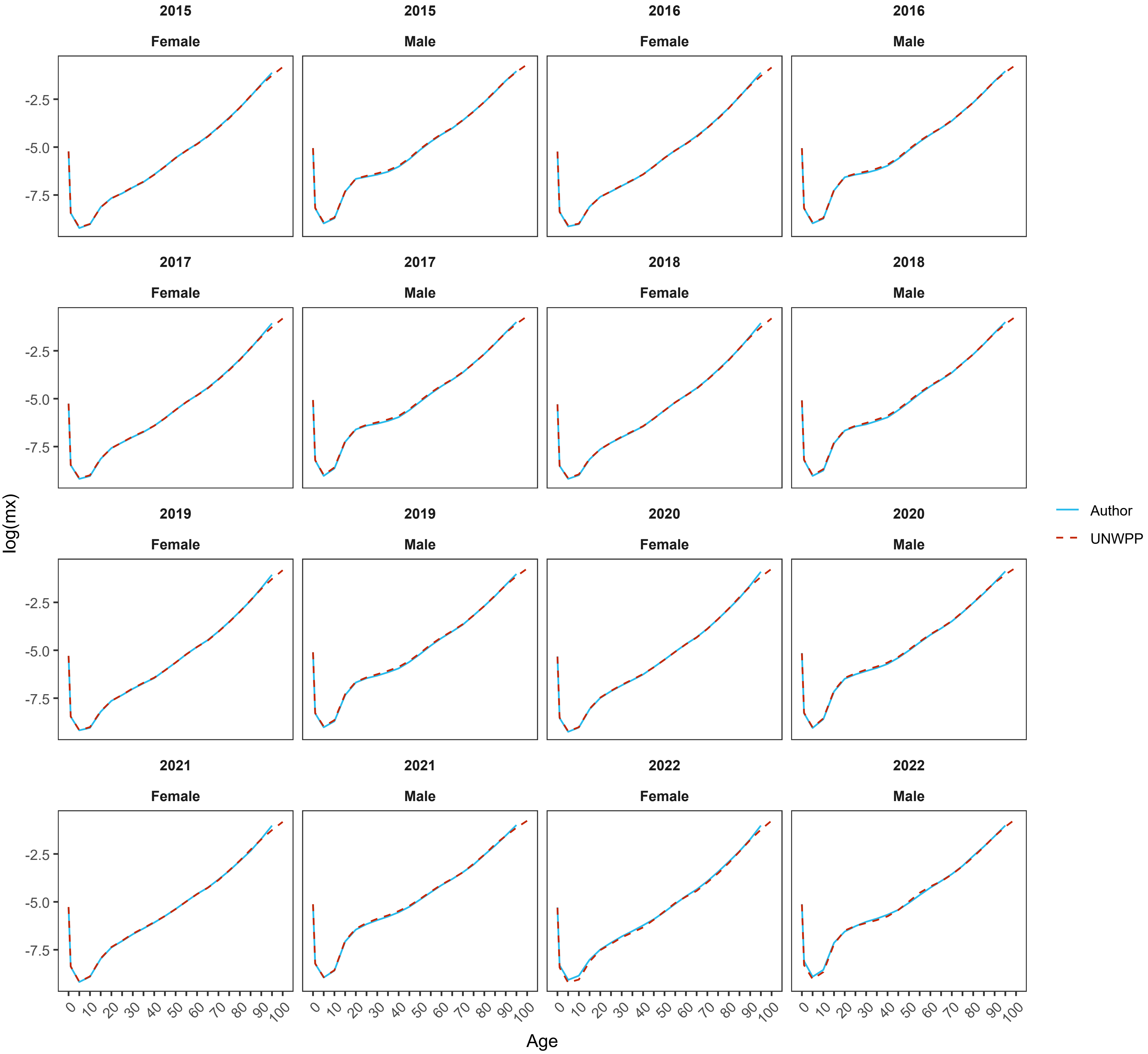

Figure S2a

Comparison of age-group-specific logged mortality rates (Australia),
author data set (solid line) vs. HLD (dashed line)

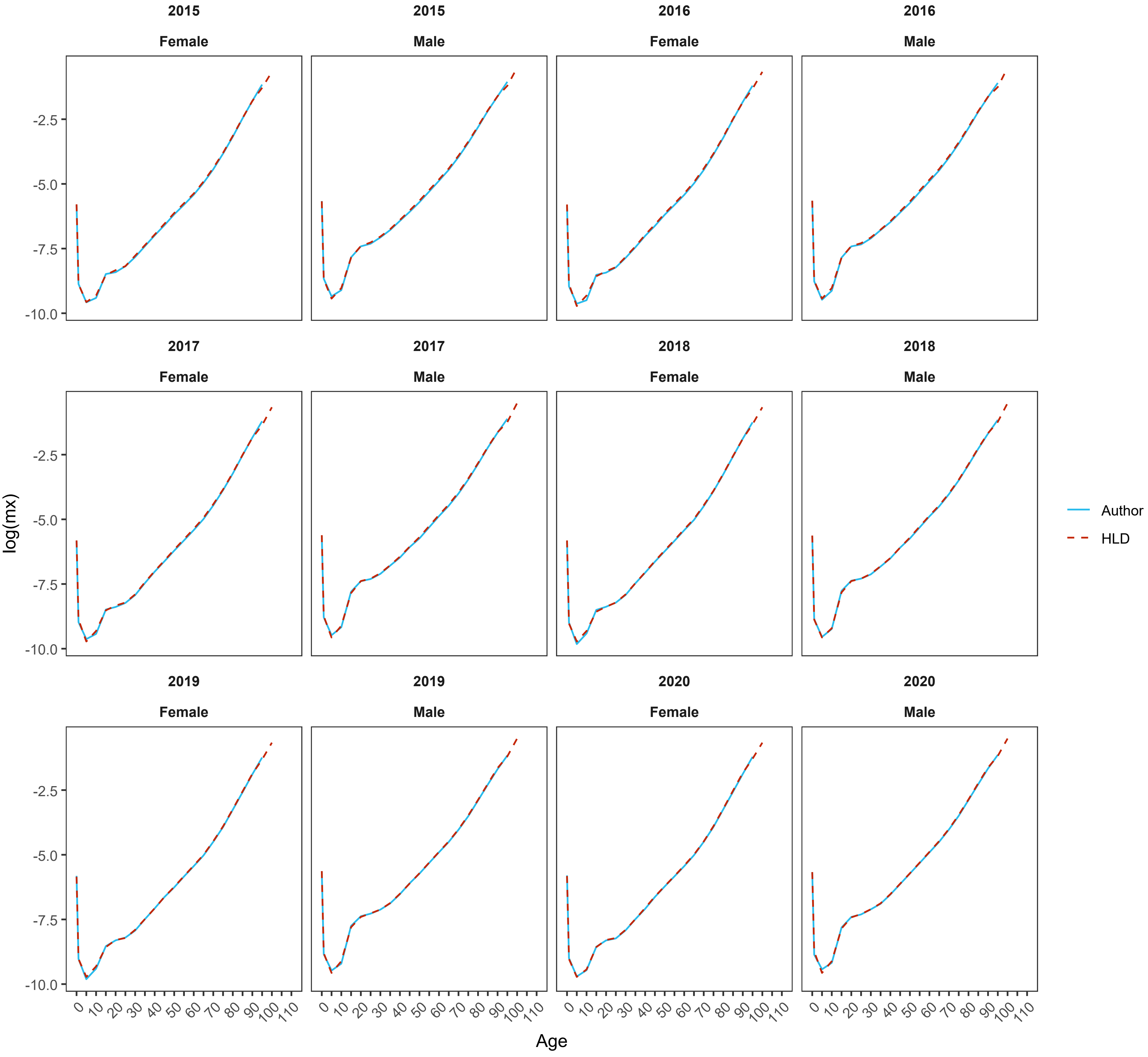

Figure S2b

Comparison of age-group-specific logged mortality rates (Austria), author data set (solid line) vs. HLD (dashed line)

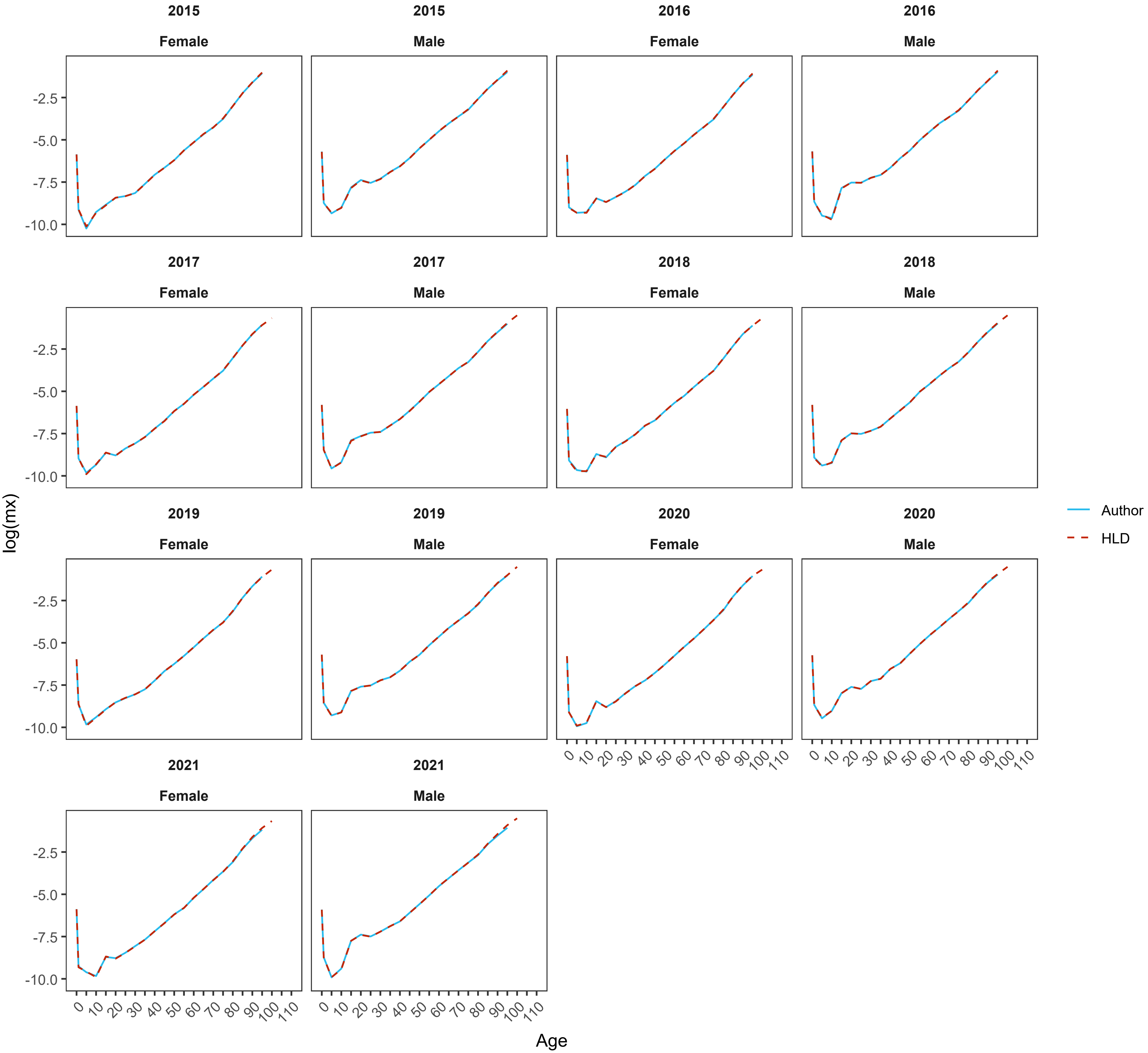

Figure S2c

Comparison of age-group-specific logged mortality rates (Bulgaria), author data set (solid line) vs. HLD (dashed line)

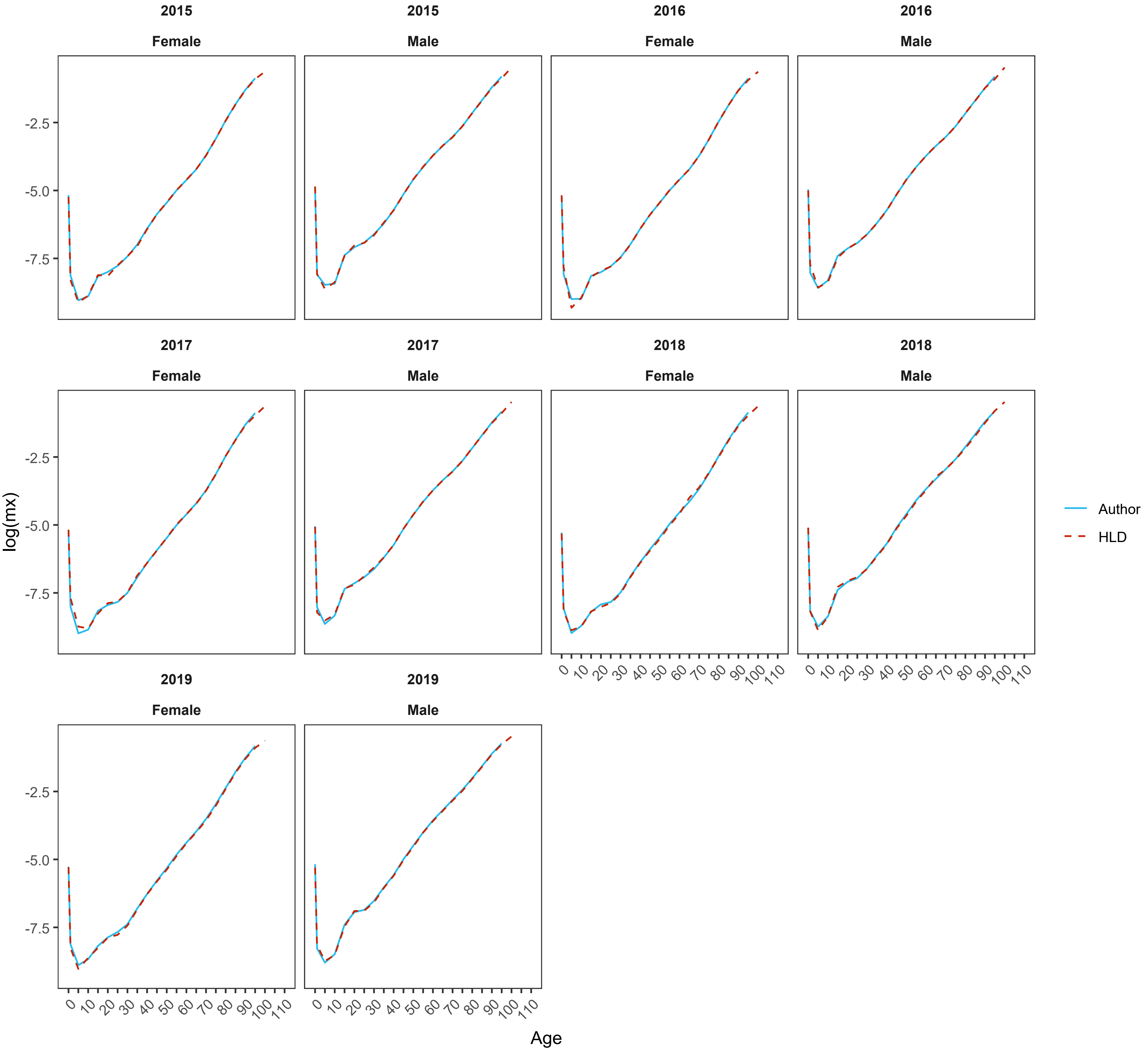

Figure S2d

Comparison of age-group-specific logged mortality rates (Brazil),
author data set (solid line) vs. HLD (dashed line)

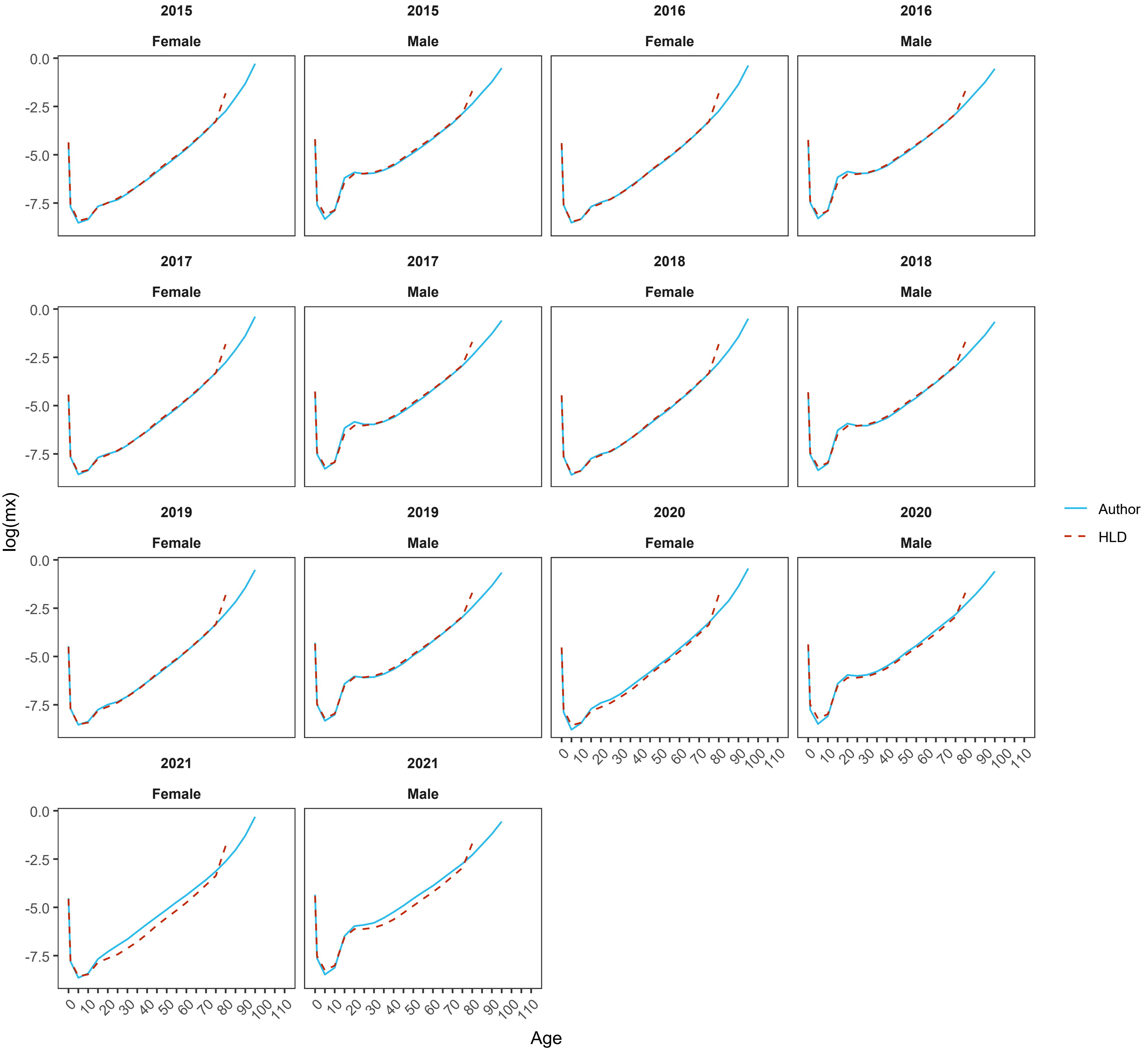

Figure S2e

Comparison of age-group-specific logged mortality rates (Canada),
author data set (solid line) vs. HLD (dashed line)

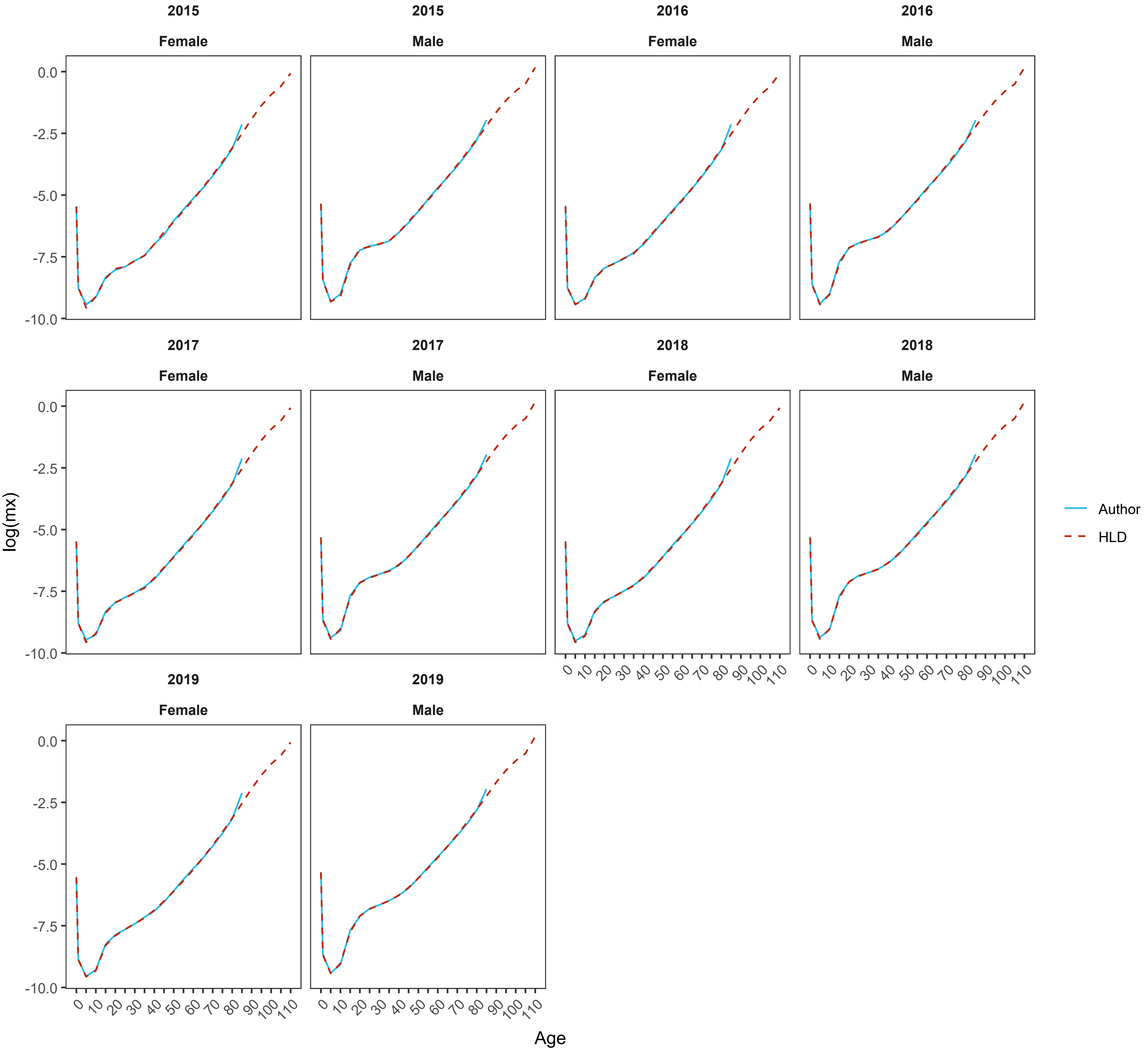

Figure S2f

Comparison of age-group-specific logged mortality rates (Switzerland),
author data set (solid line) vs. HLD (dashed line)

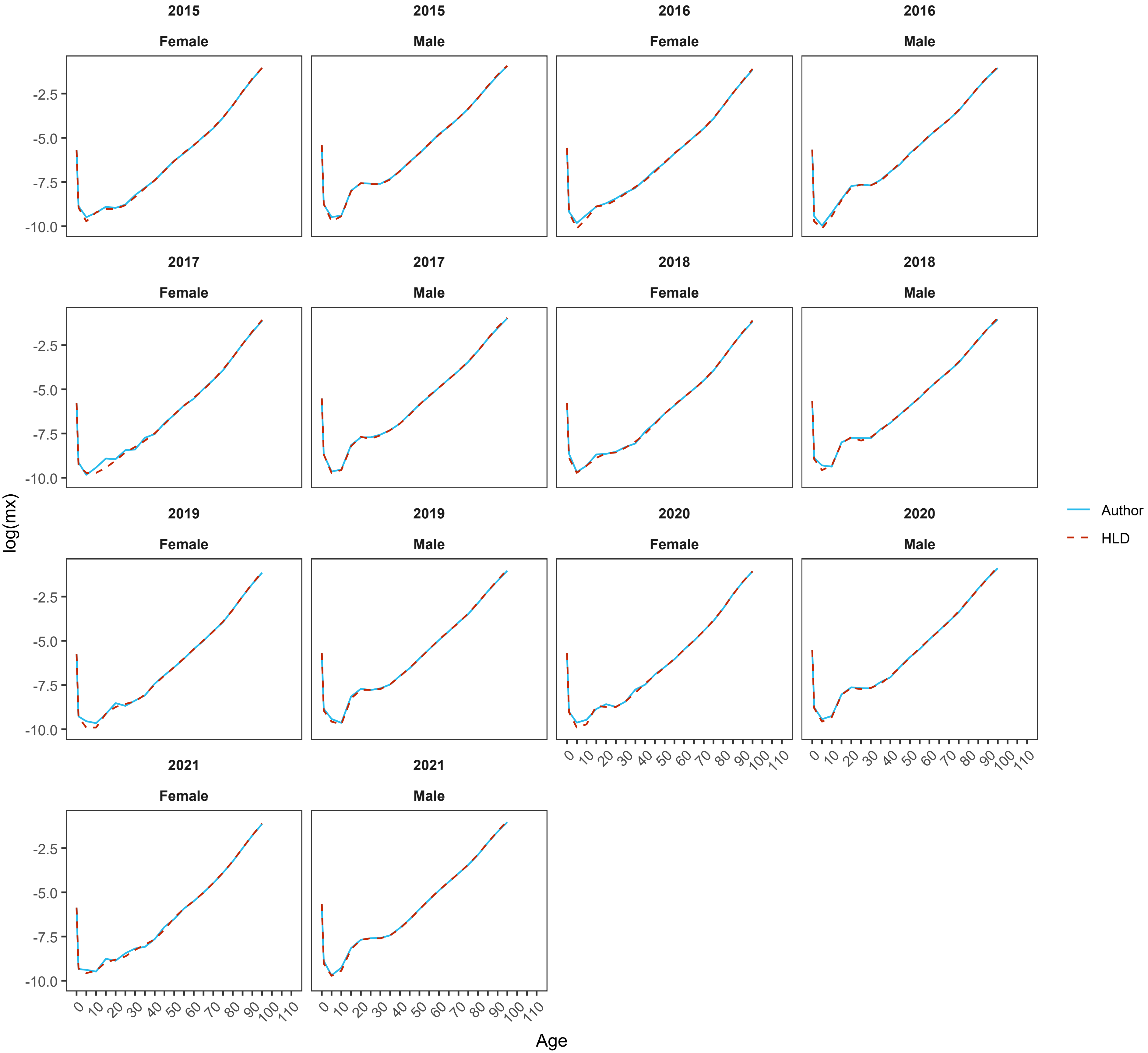

Figure S2g

Comparison of age-group-specific logged mortality rates (Czechia), author data set (solid line) vs. HLD (dashed line)

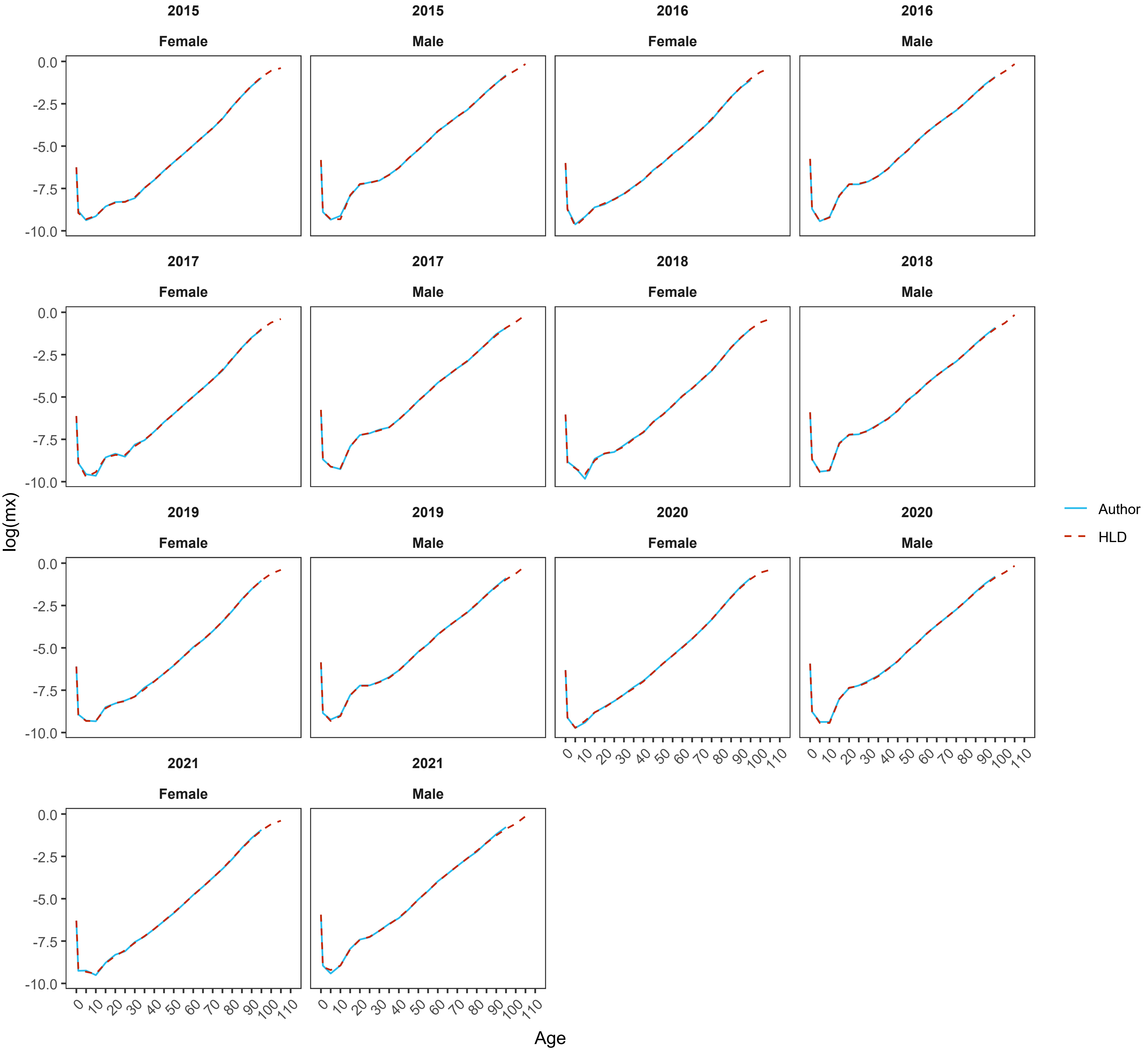

Figure S2h

Comparison of age-group-specific logged mortality rates (Denmark), author data set (solid line) vs. HLD (dashed line)

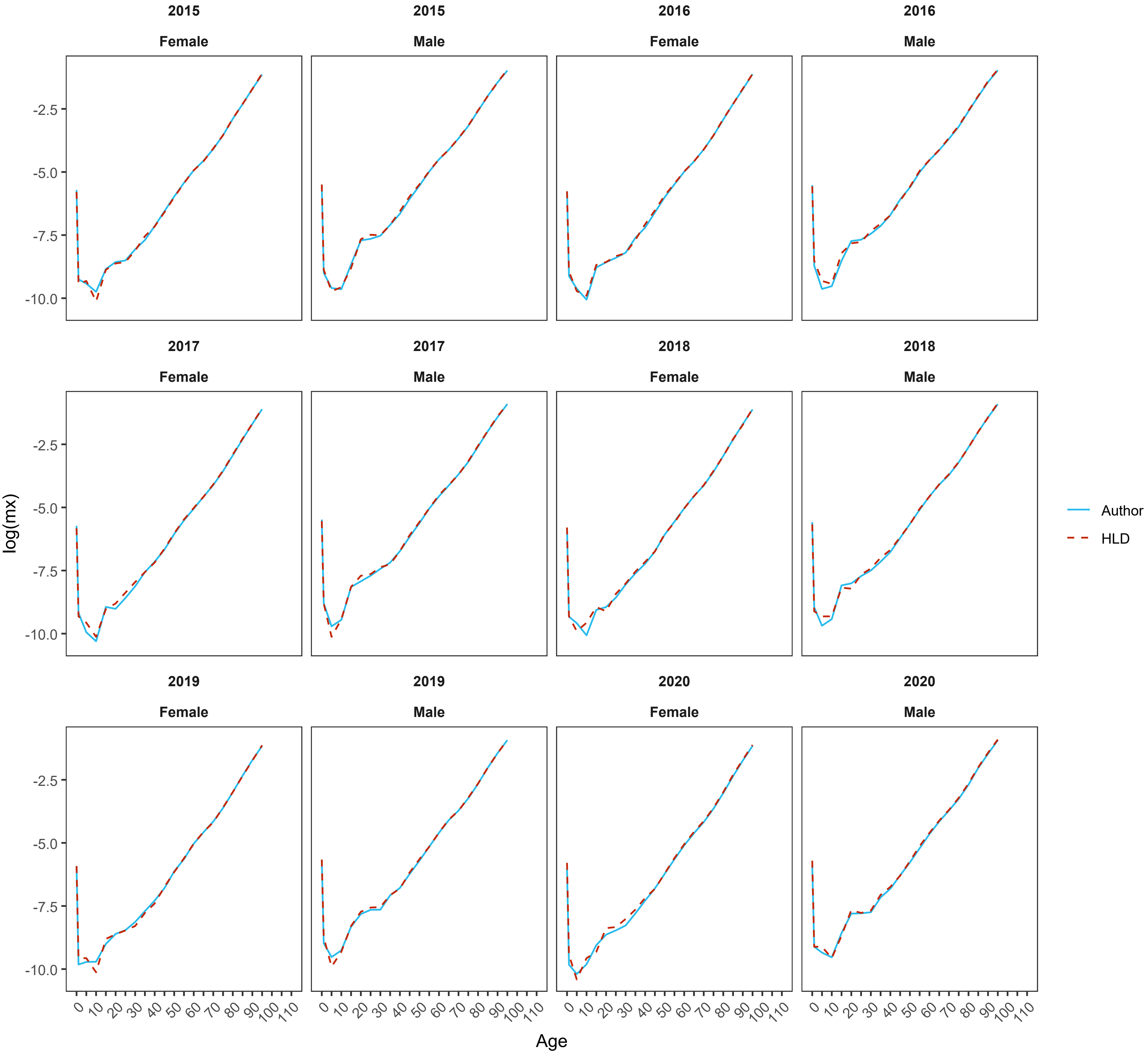

Figure S2i

Comparison of age-group-specific logged mortality rates (Spain), author data set (solid line) vs. HLD (dashed line)

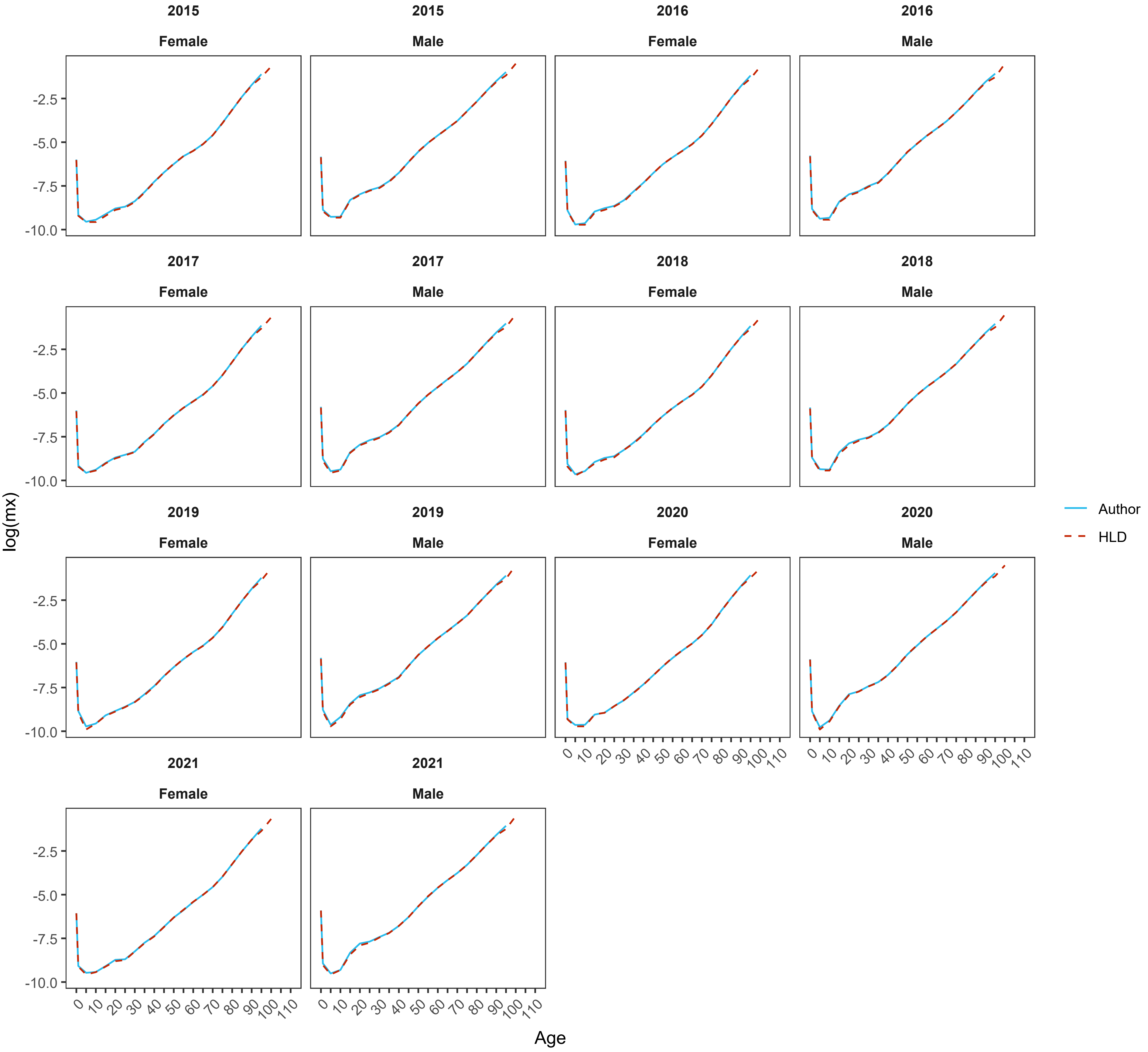

Figure S2j

Comparison of age-group-specific logged mortality rates (Hungary), author data set (solid line) vs. HLD (dashed line)

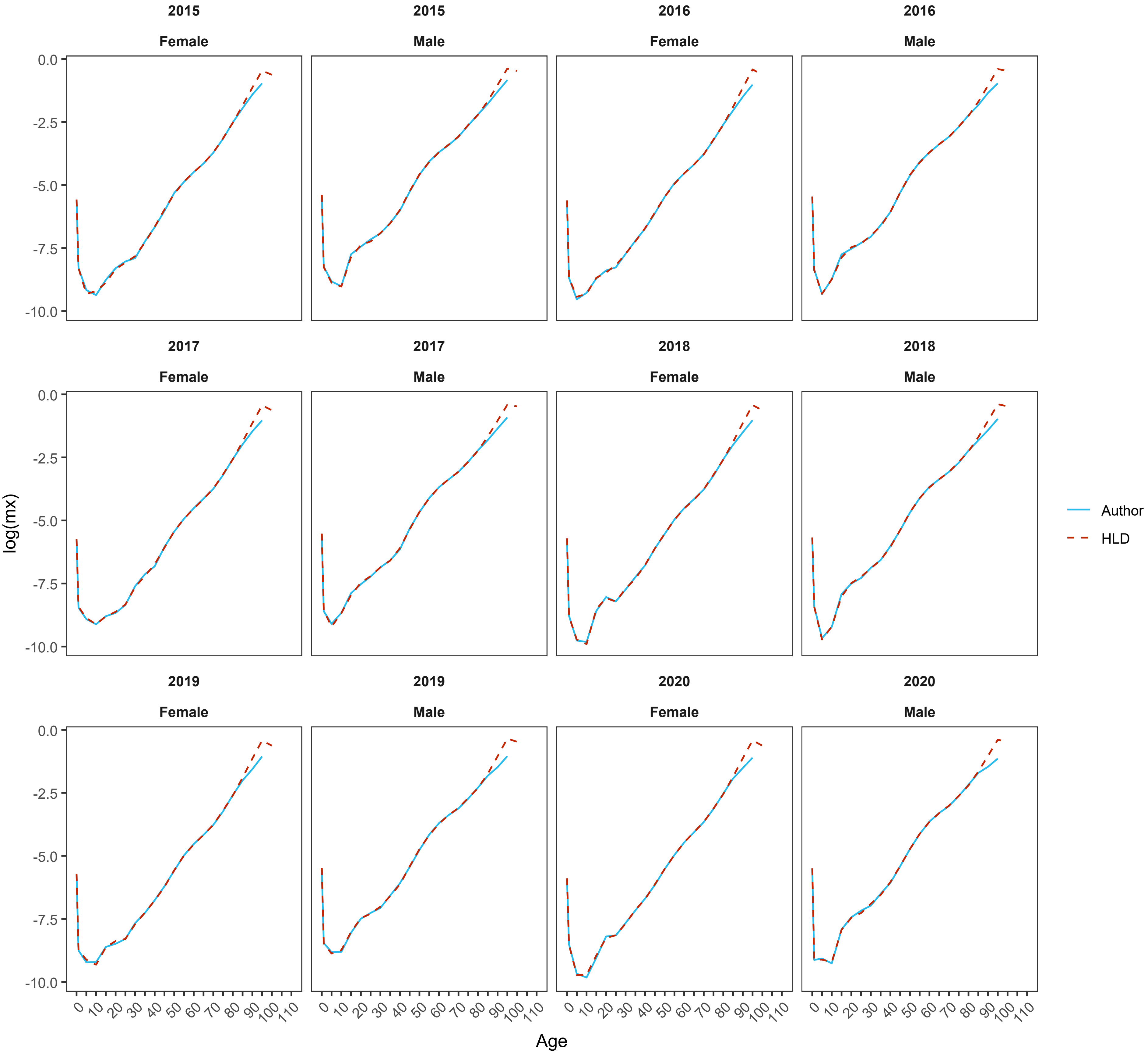

Figure S2k

Comparison of age-group-specific logged mortality rates (Japan),
author data set (solid line) vs. HLD (dashed line)

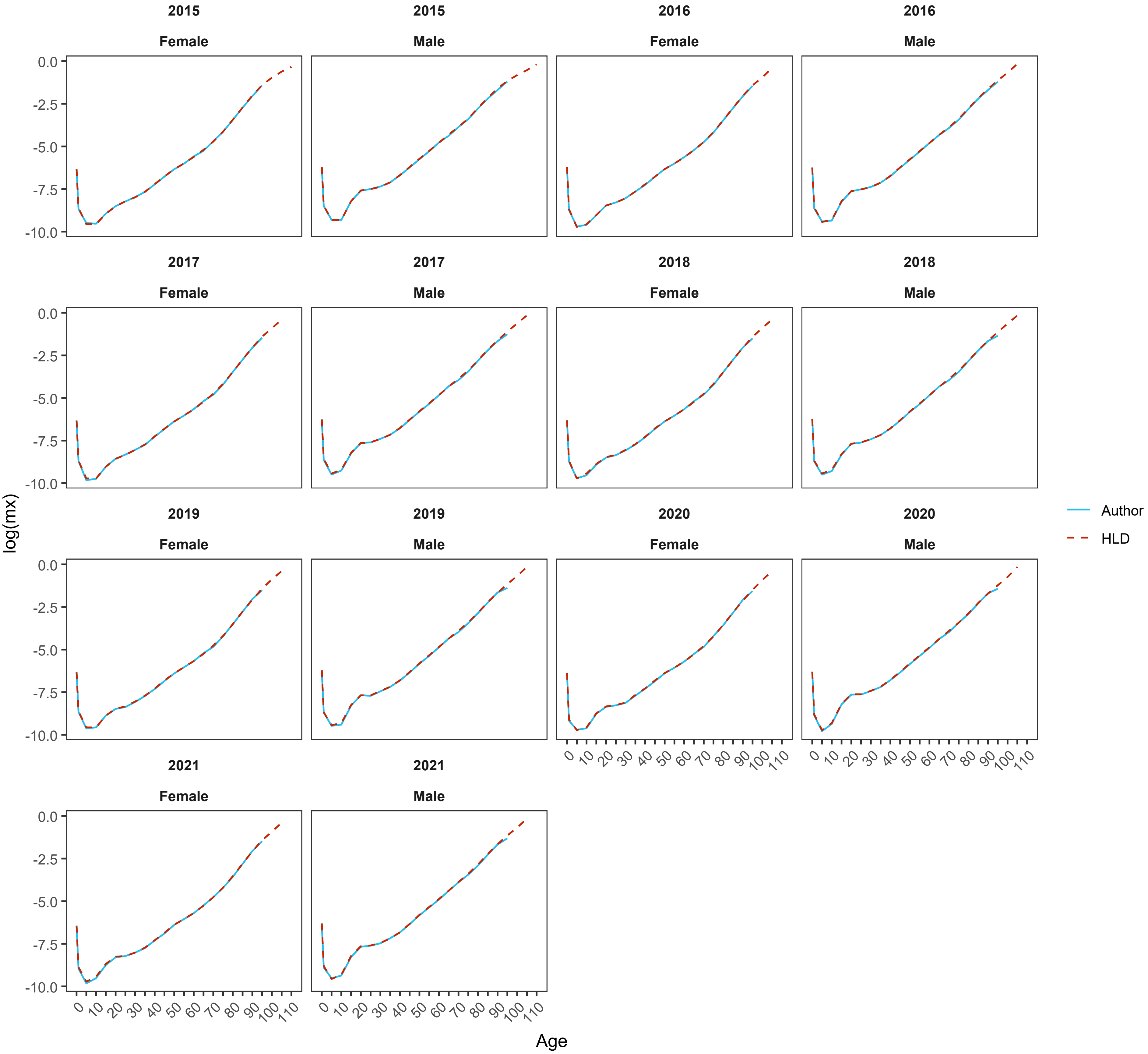

Figure S2l

Comparison of age-group-specific logged mortality rates (South Korea), author data set (solid line) vs. HLD (dashed line)

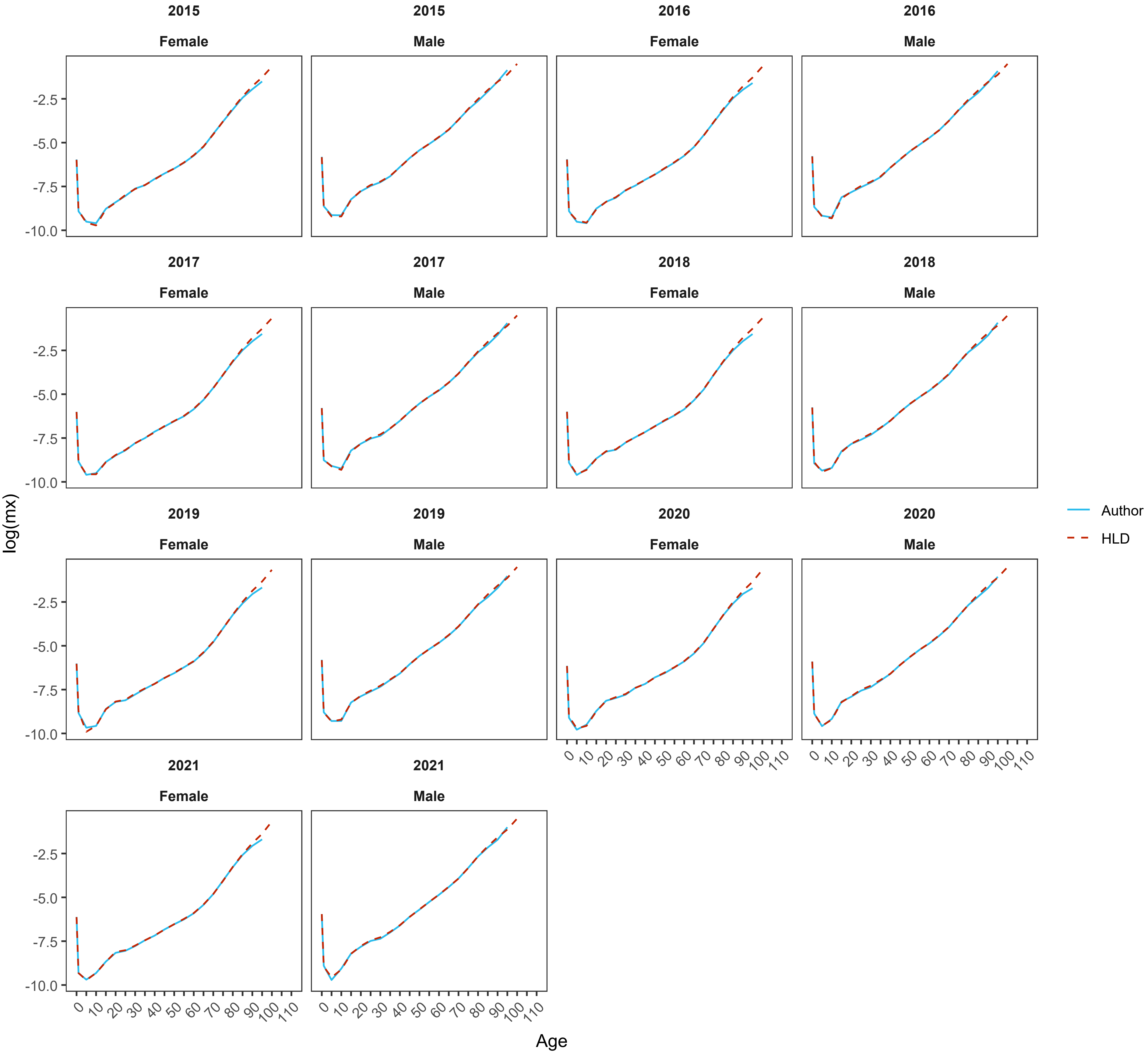

Figure S2m

Comparison of age-group-specific logged mortality rates (Latvia),
author data set (solid line) vs. HLD (dashed line)

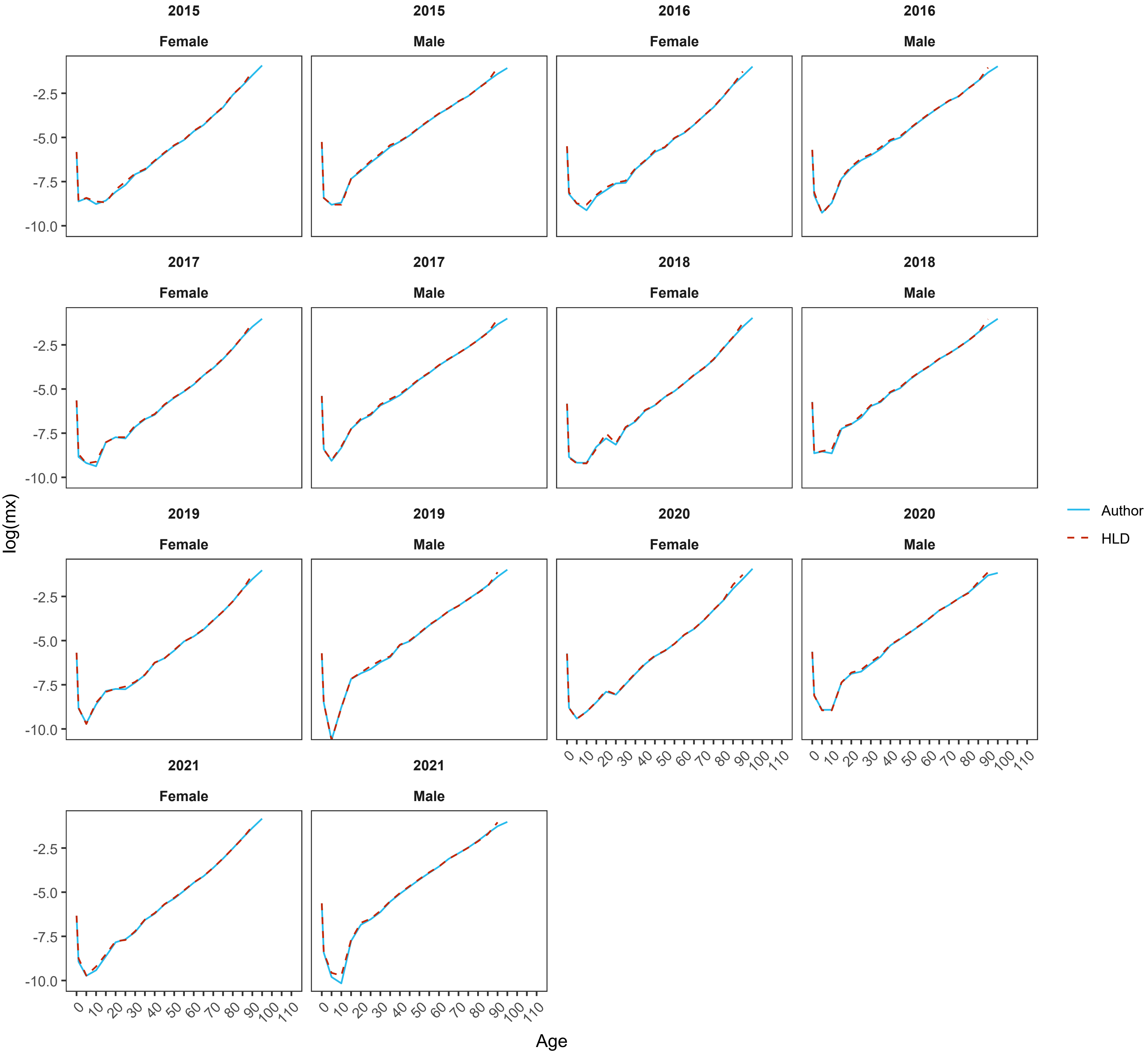

Figure S2n

Comparison of age-group-specific logged mortality rates (Netherlands),
author data set (solid line) vs. HLD (dashed line)

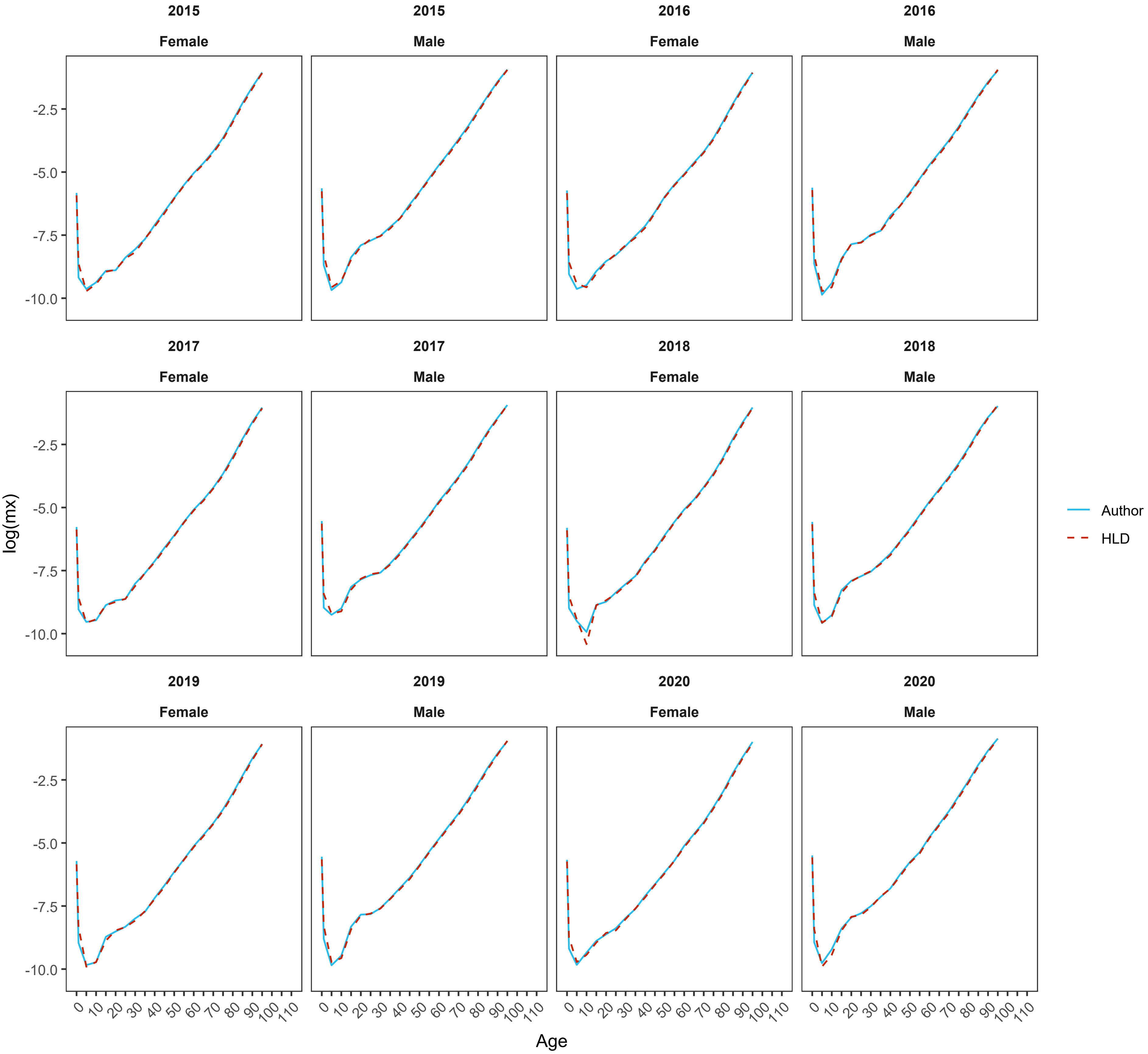

Figure S2o

Comparison of age-group-specific logged mortality rates (Poland),
author data set (solid line) vs. HLD (dashed line)

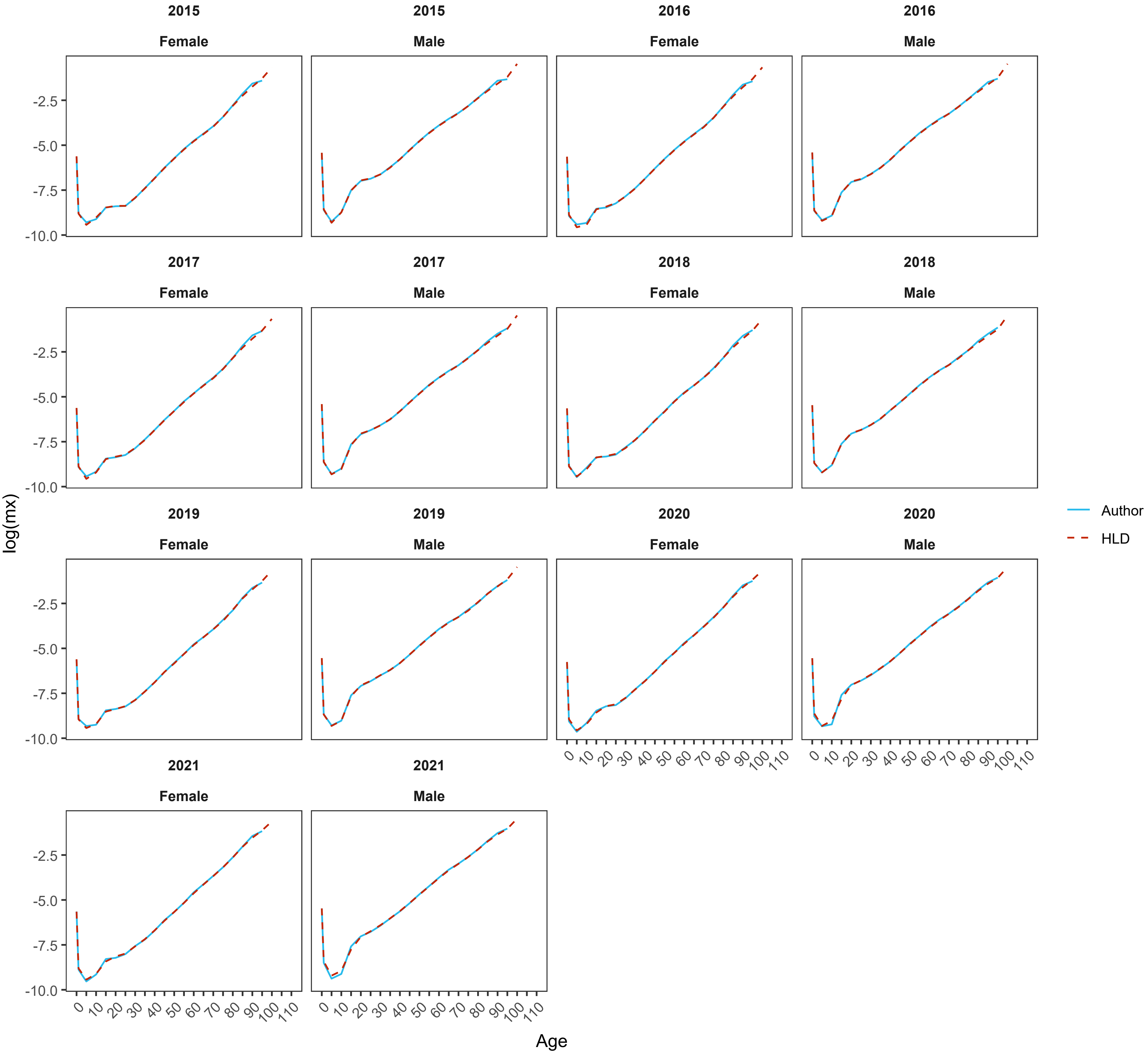

Figure S2p

Comparison of age-group-specific logged mortality rates (Russia),
author data set (solid line) vs. HLD (dashed line)

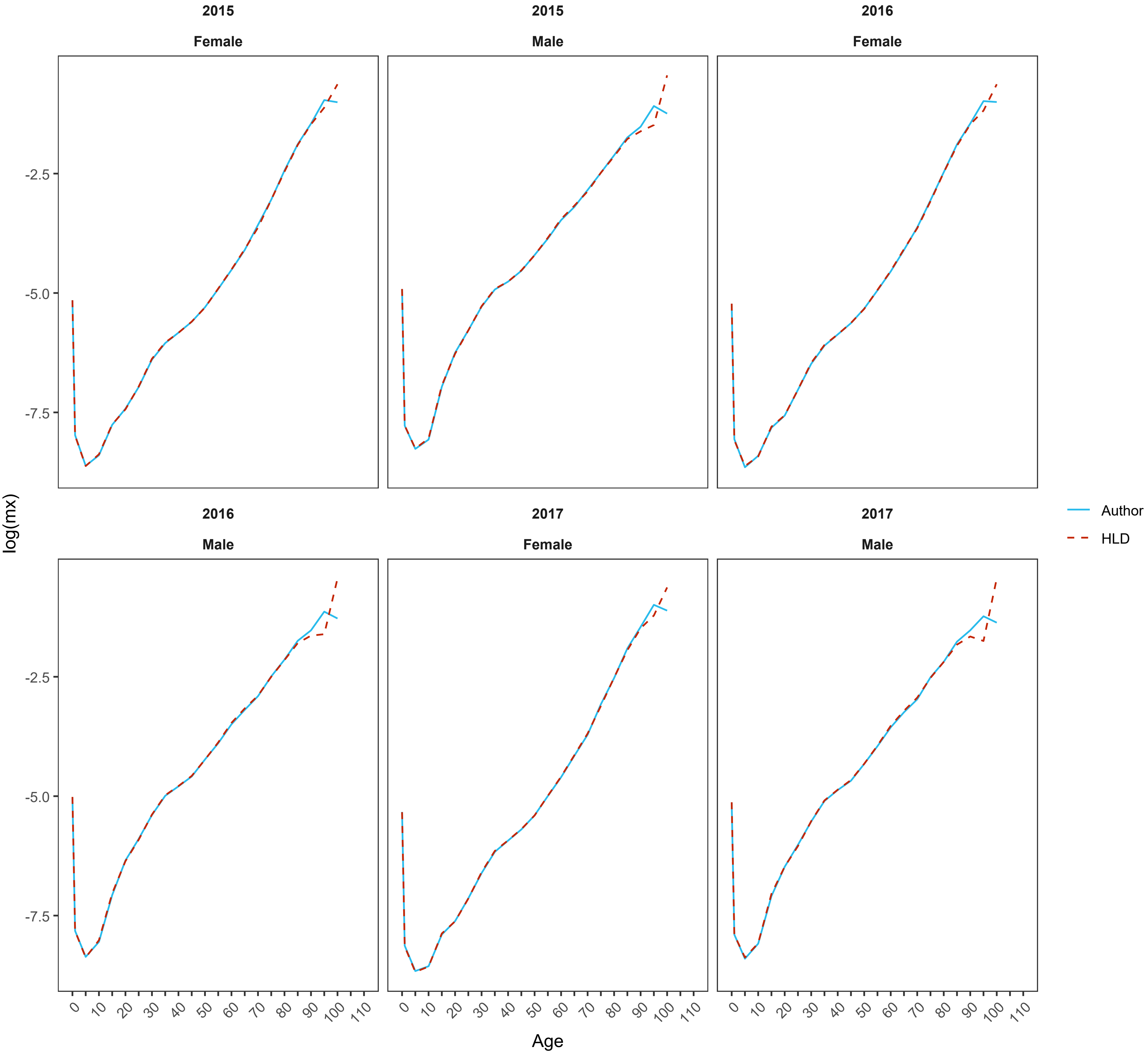

Figure S2q

Comparison of age-group-specific logged mortality rates (Sweden), author data set (solid line) vs. HLD (dashed line)

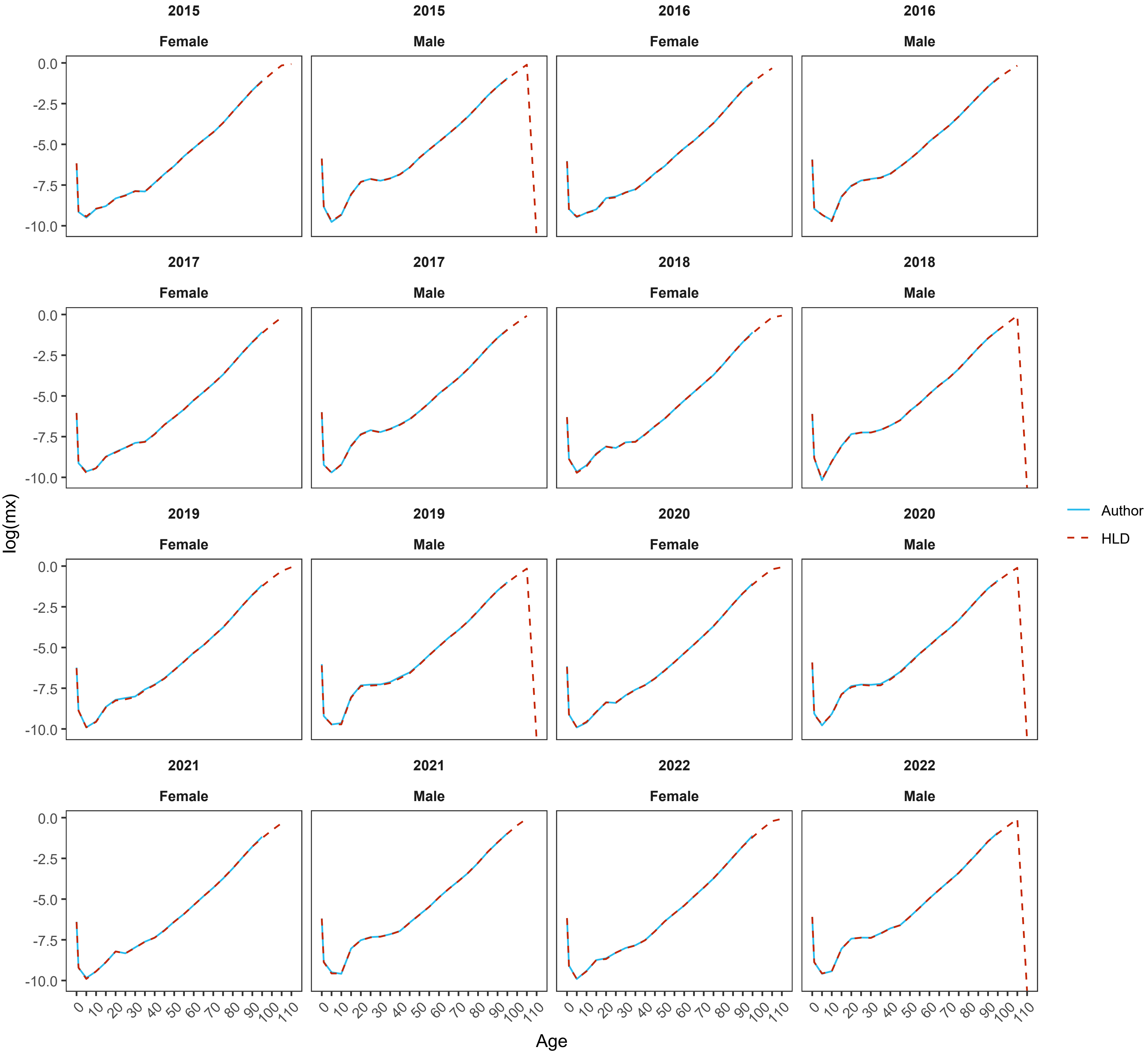

Figure S2r

Comparison of age-group-specific logged mortality rates (USA), author data set (solid line) vs. HLD (dashed line)

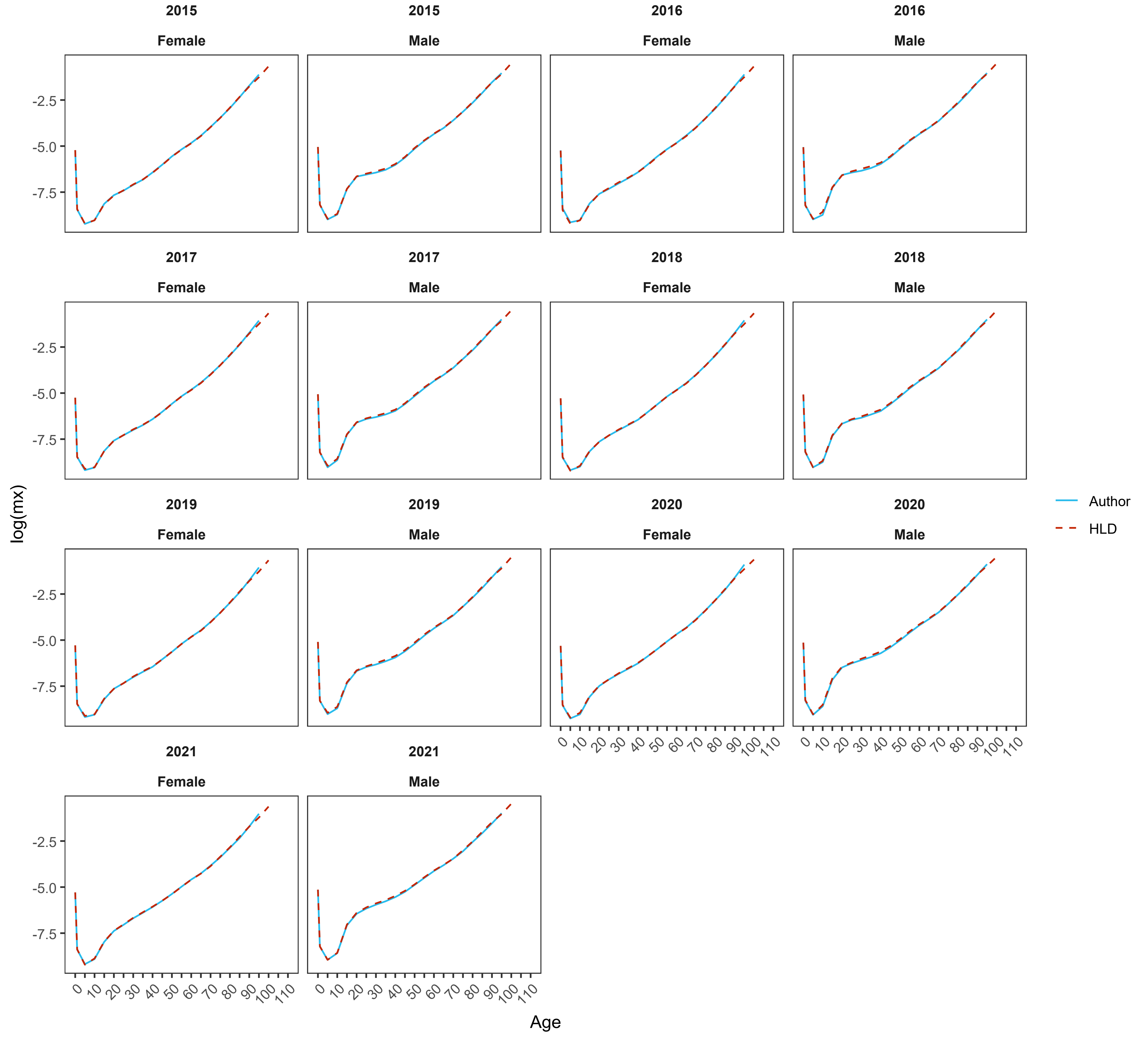

Figure S3a

Comparison of age-group-specific logged mortality rates (Australia),
author data set (solid line) vs. HMD (dashed line)

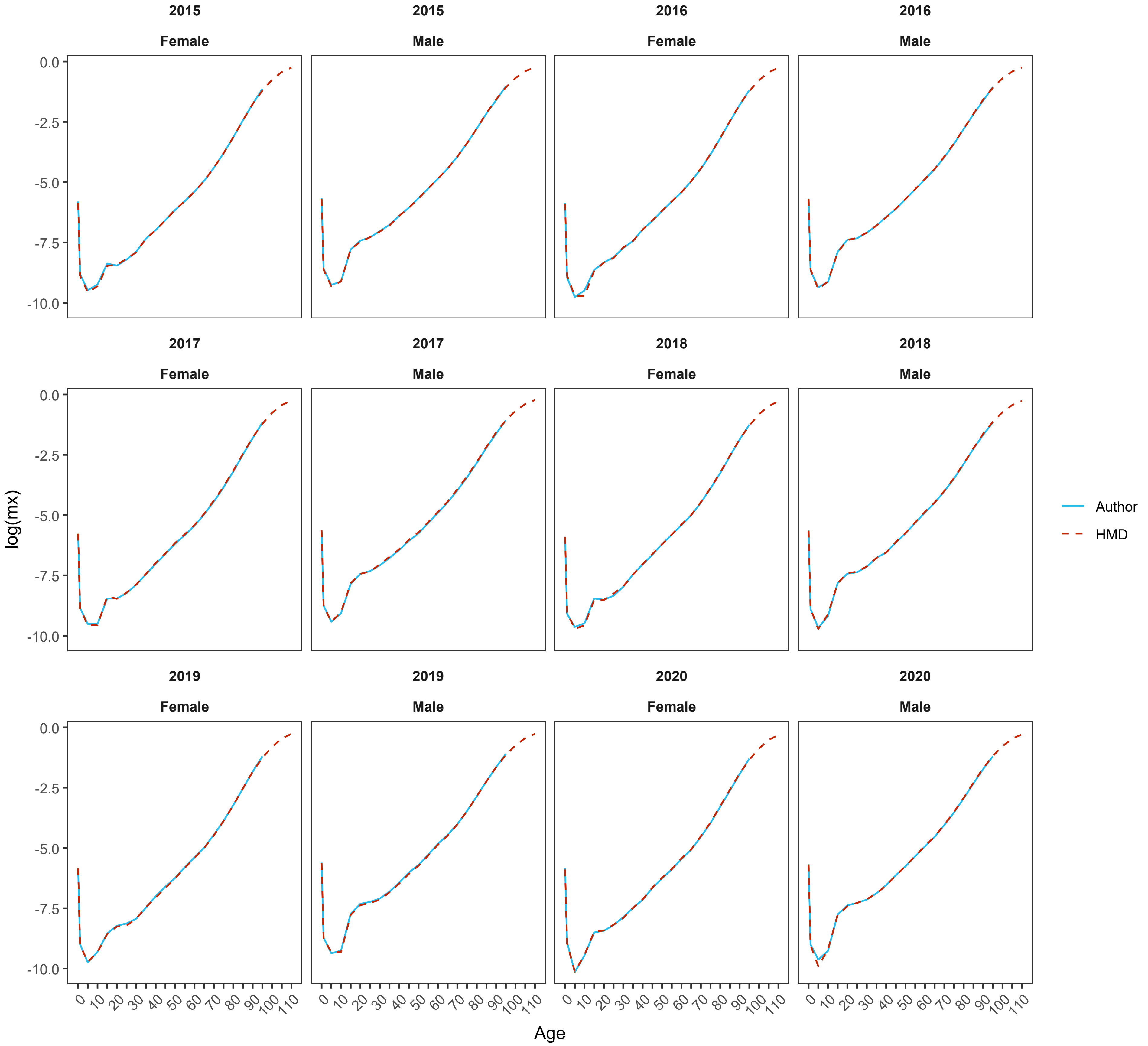

Figure S3b

Comparison of age-group-specific logged mortality rates (Austria), author data set (solid line) vs. HMD (dashed line)

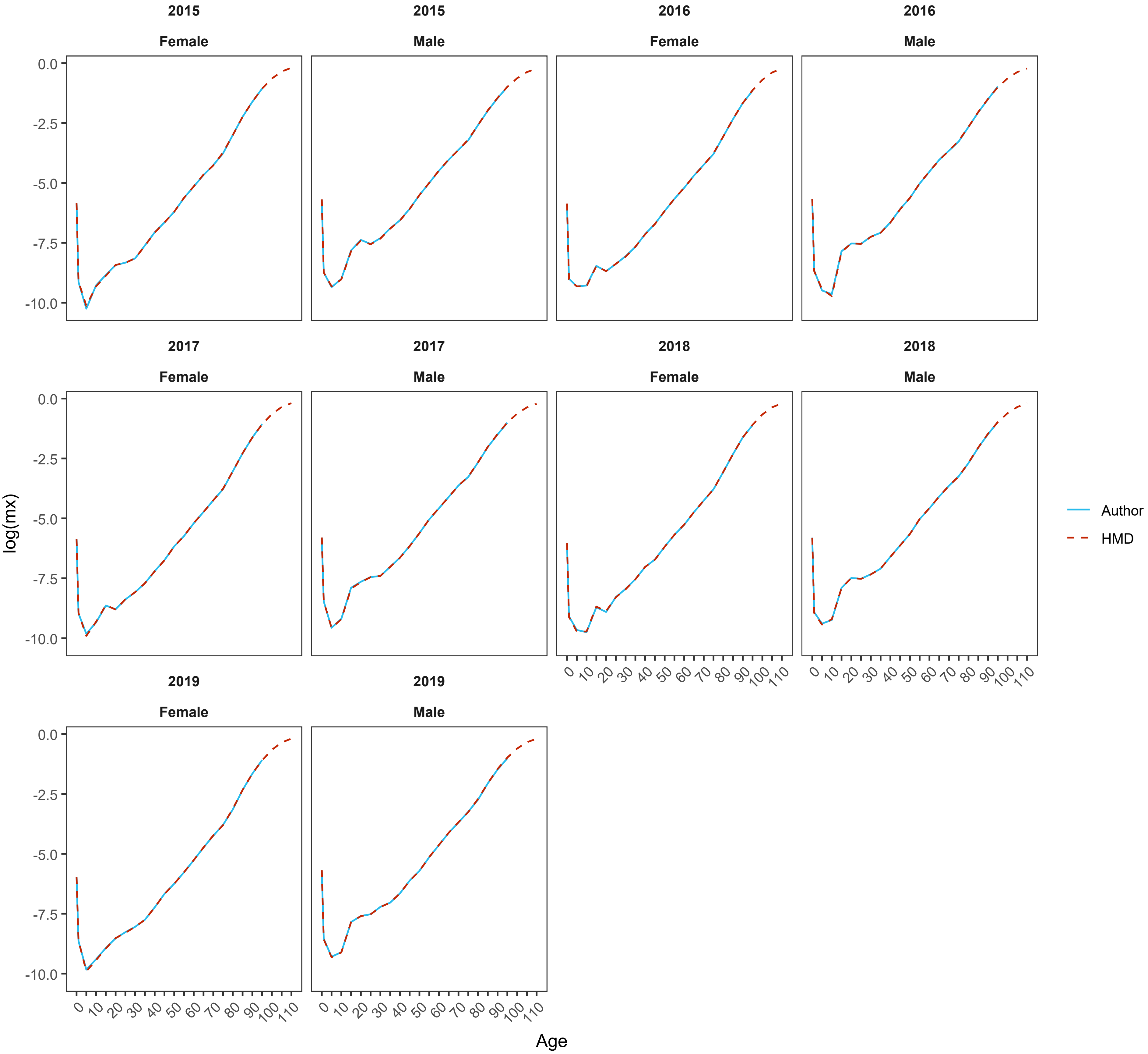

Figure S3c

Comparison of age-group-specific logged mortality rates (Bulgaria), author data set (solid line) vs. HMD (dashed line)

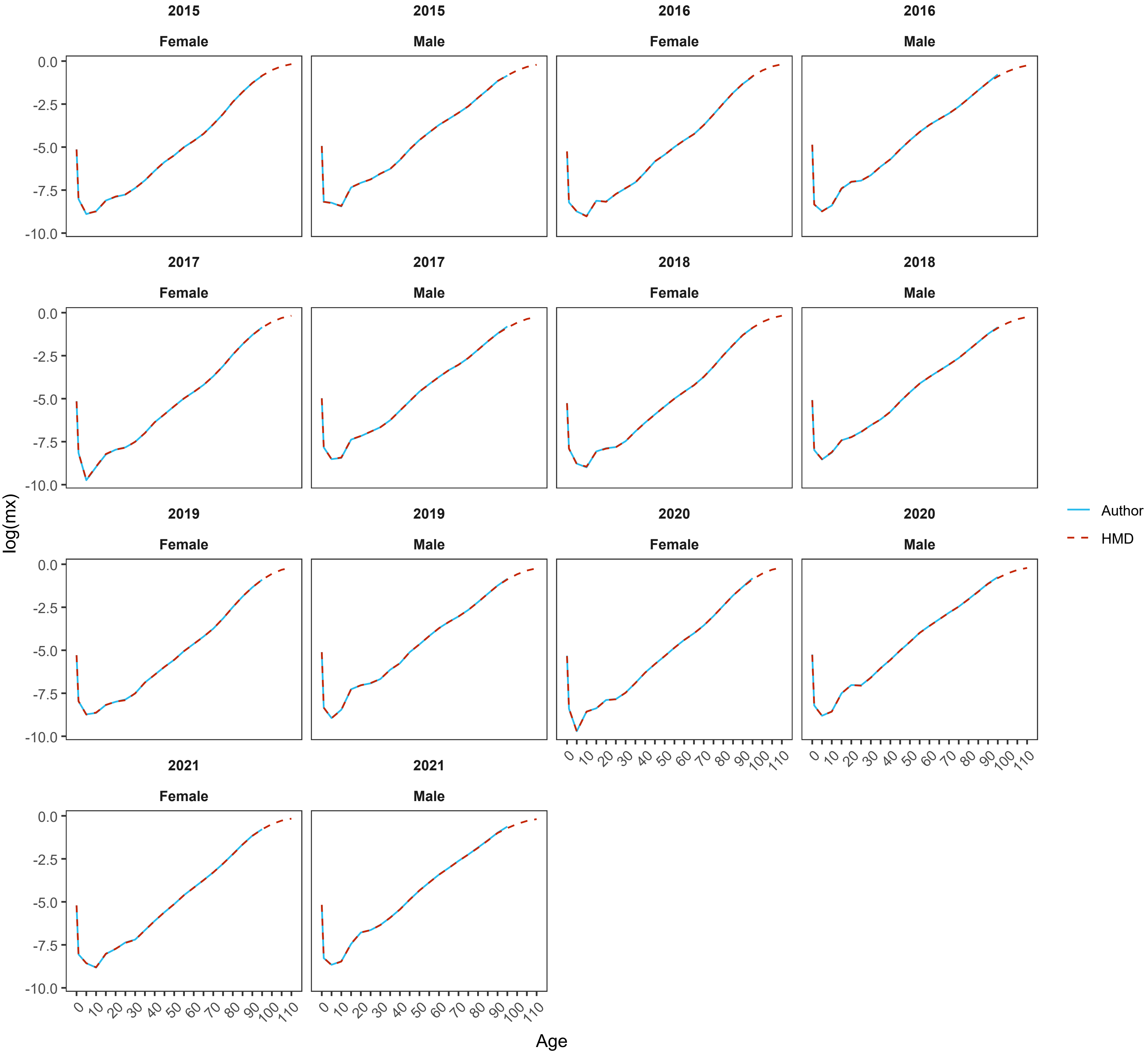

Figure S3d

Comparison of age-group-specific logged mortality rates (Canada),
author data set (solid line) vs. HMD (dashed line)

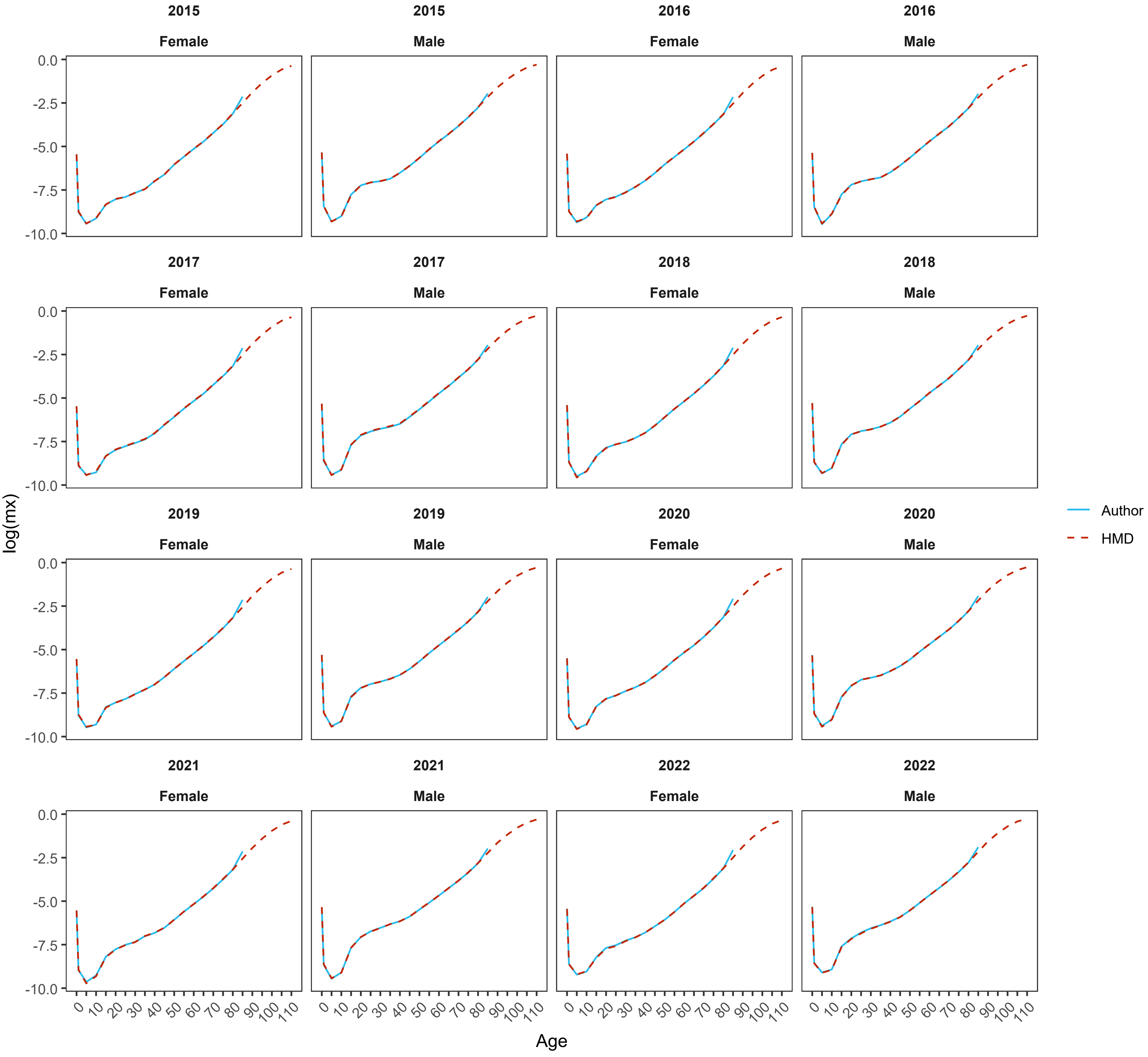

Figure S3e

Comparison of age-group-specific logged mortality rates (Chile),
author data set (solid line) vs. HMD (dashed line)

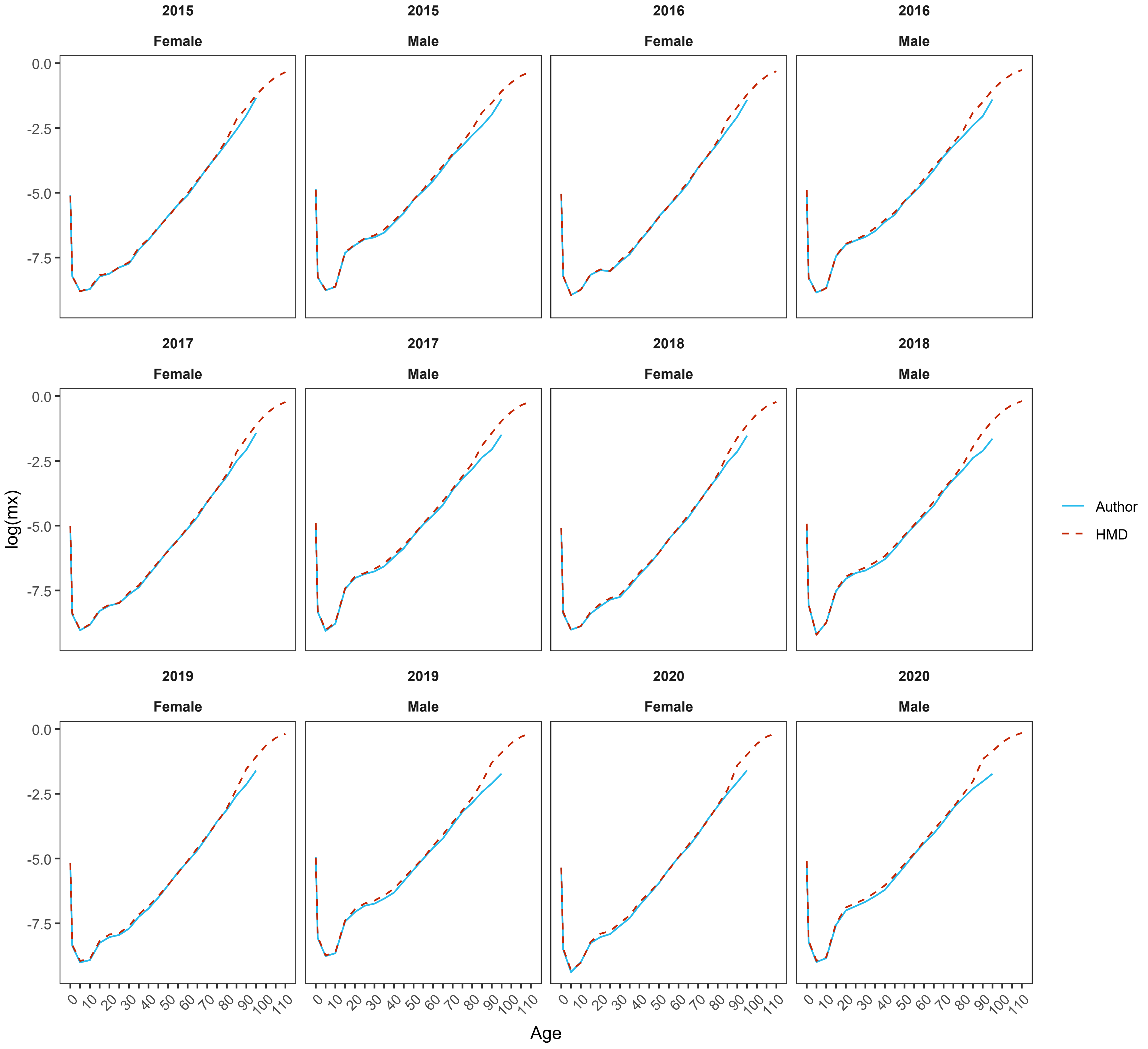

Figure S3f

Comparison of age-group-specific logged mortality rates (Croatia), author data set (solid line) vs. HMD (dashed line)

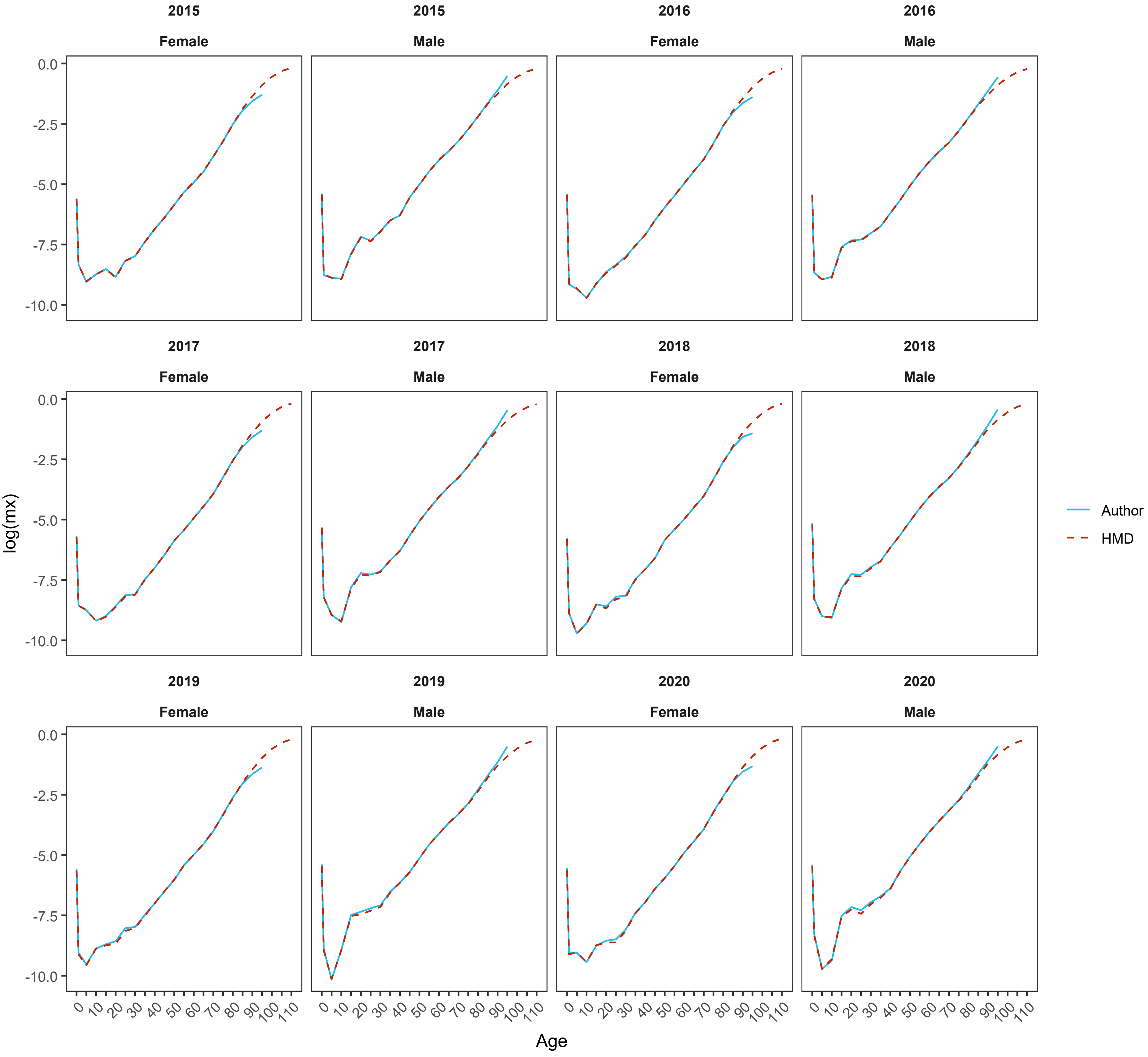

Figure S3g

Comparison of age-group-specific logged mortality rates (Czechia), author data set (solid line) vs. HMD (dashed line)

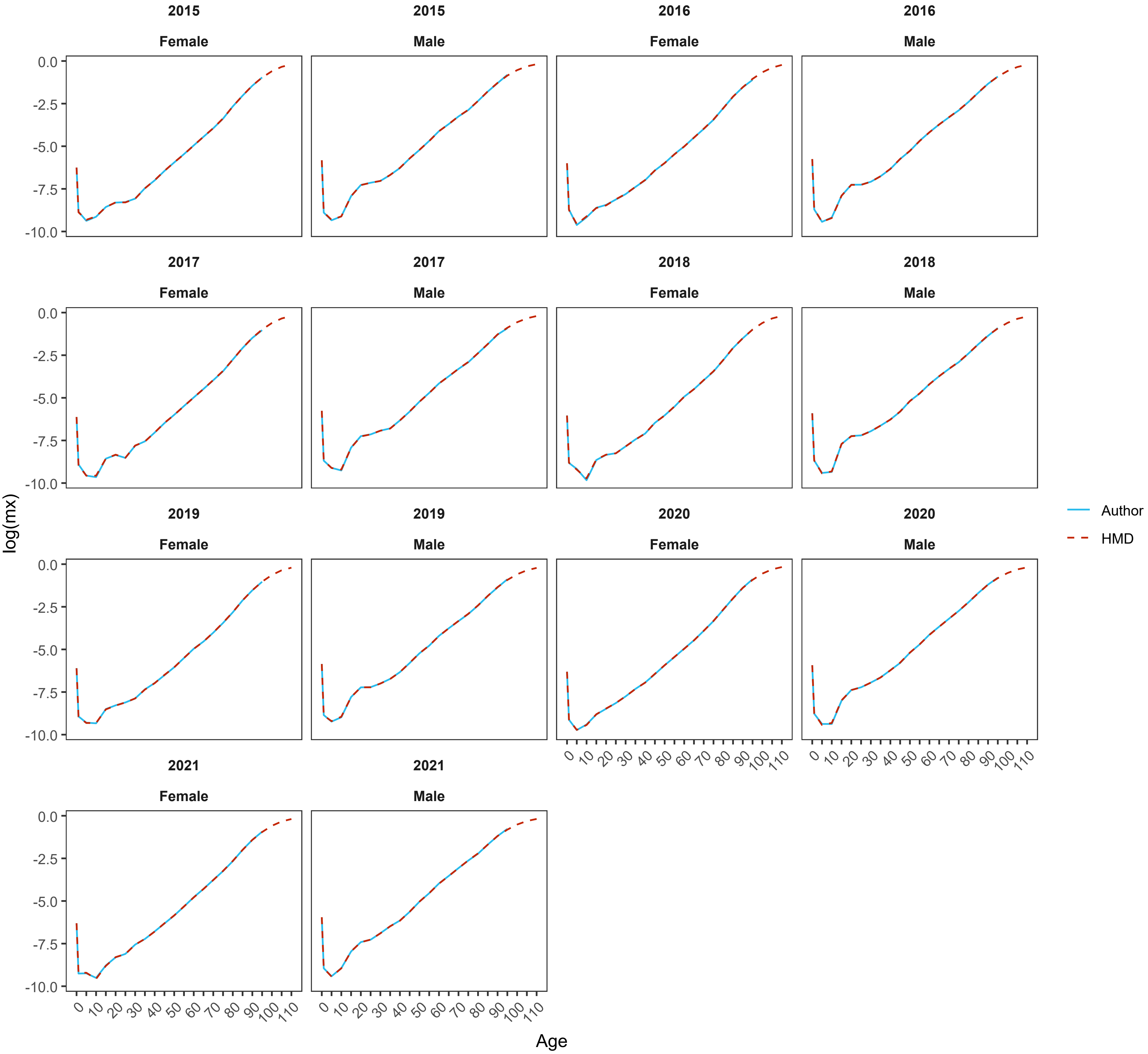

Figure S3h

Comparison of age-group-specific logged mortality rates (Denmark), author data set (solid line) vs. HMD (dashed line)

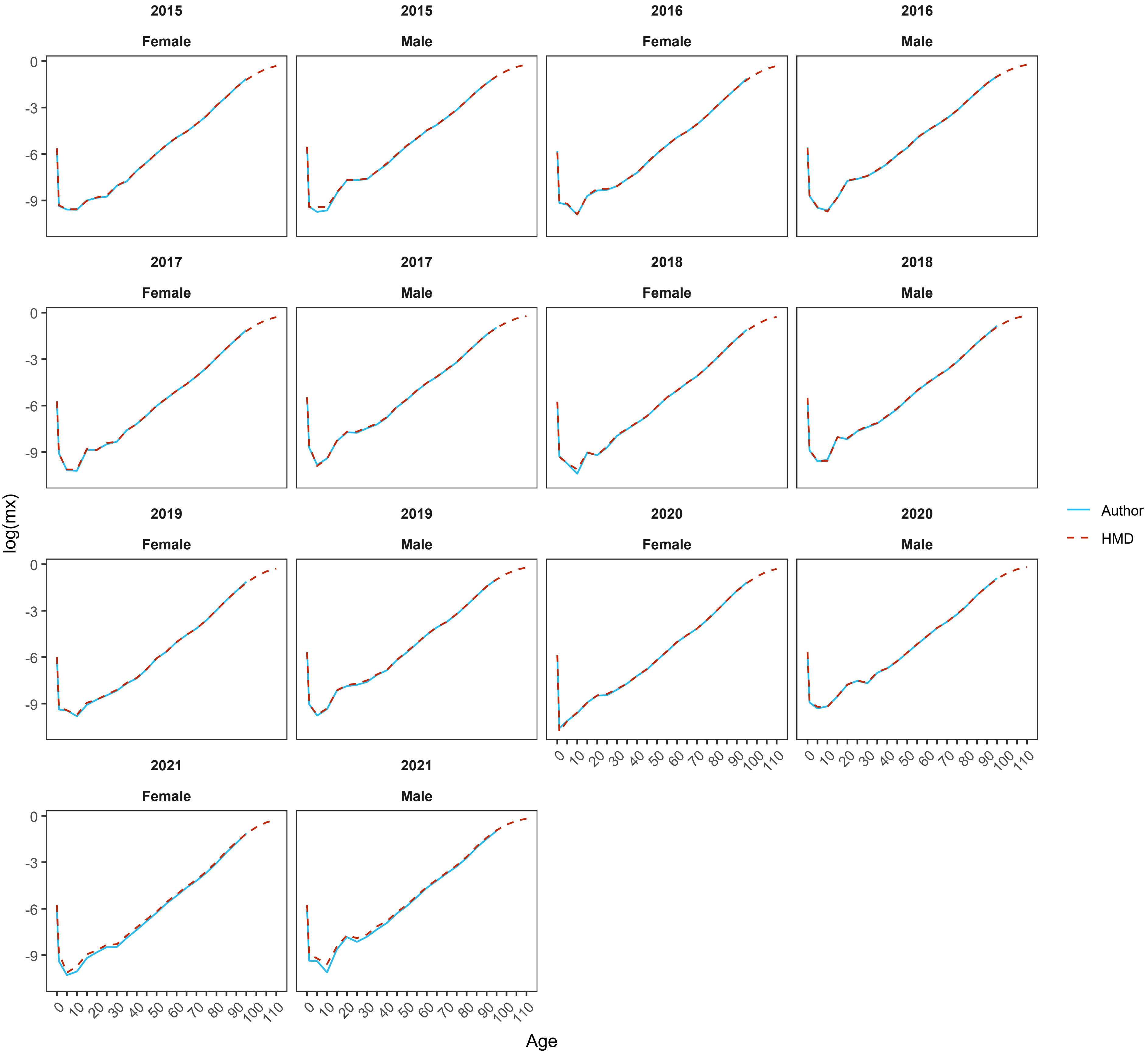

Figure S3i

Comparison of age-group-specific logged mortality rates (England and Wales),
author data set (solid line) vs. HMD (dashed line)

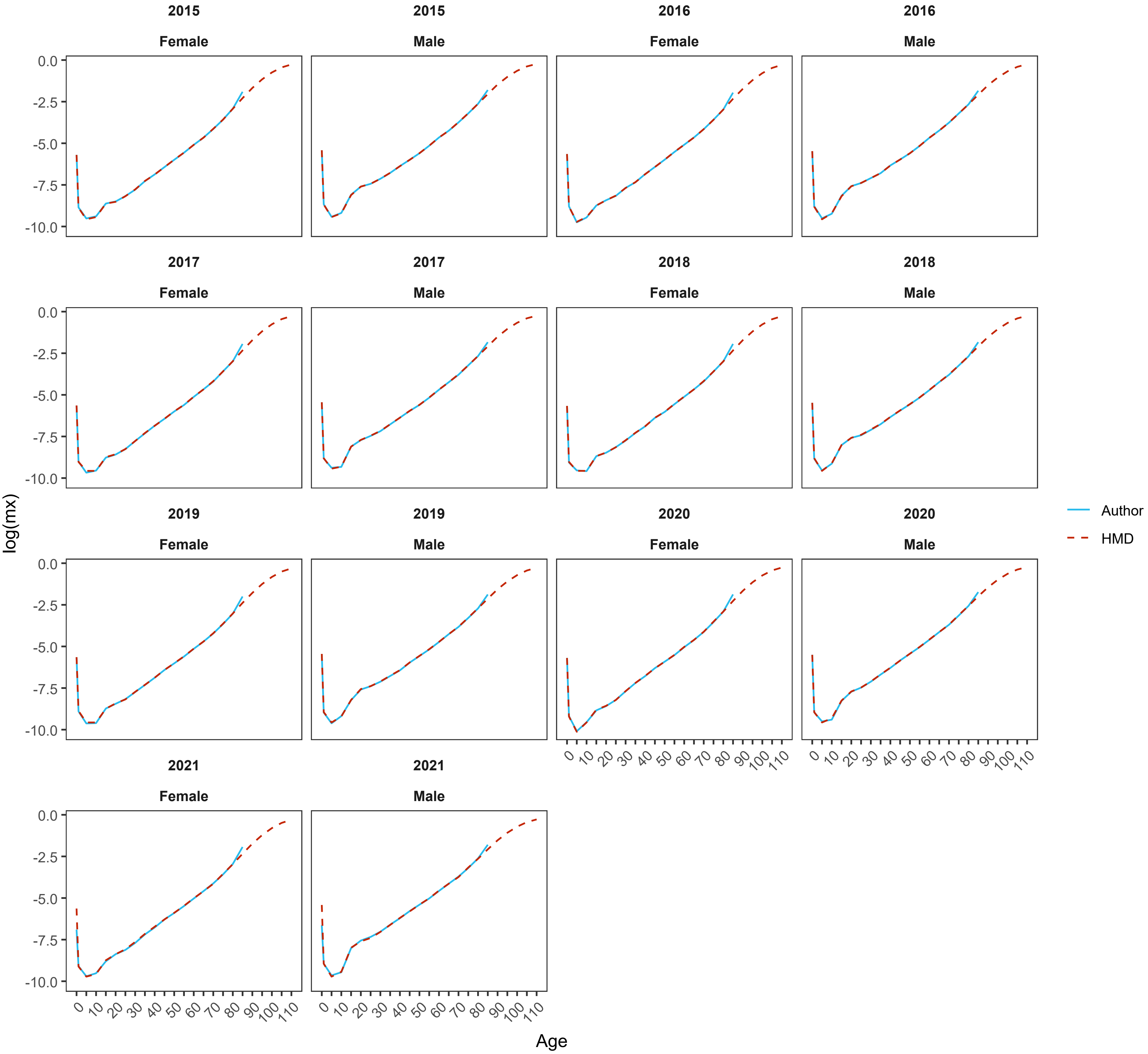

Figure S3j

Comparison of age-group-specific logged mortality rates (Hungary),
author data set (solid line) vs. HMD (dashed line)

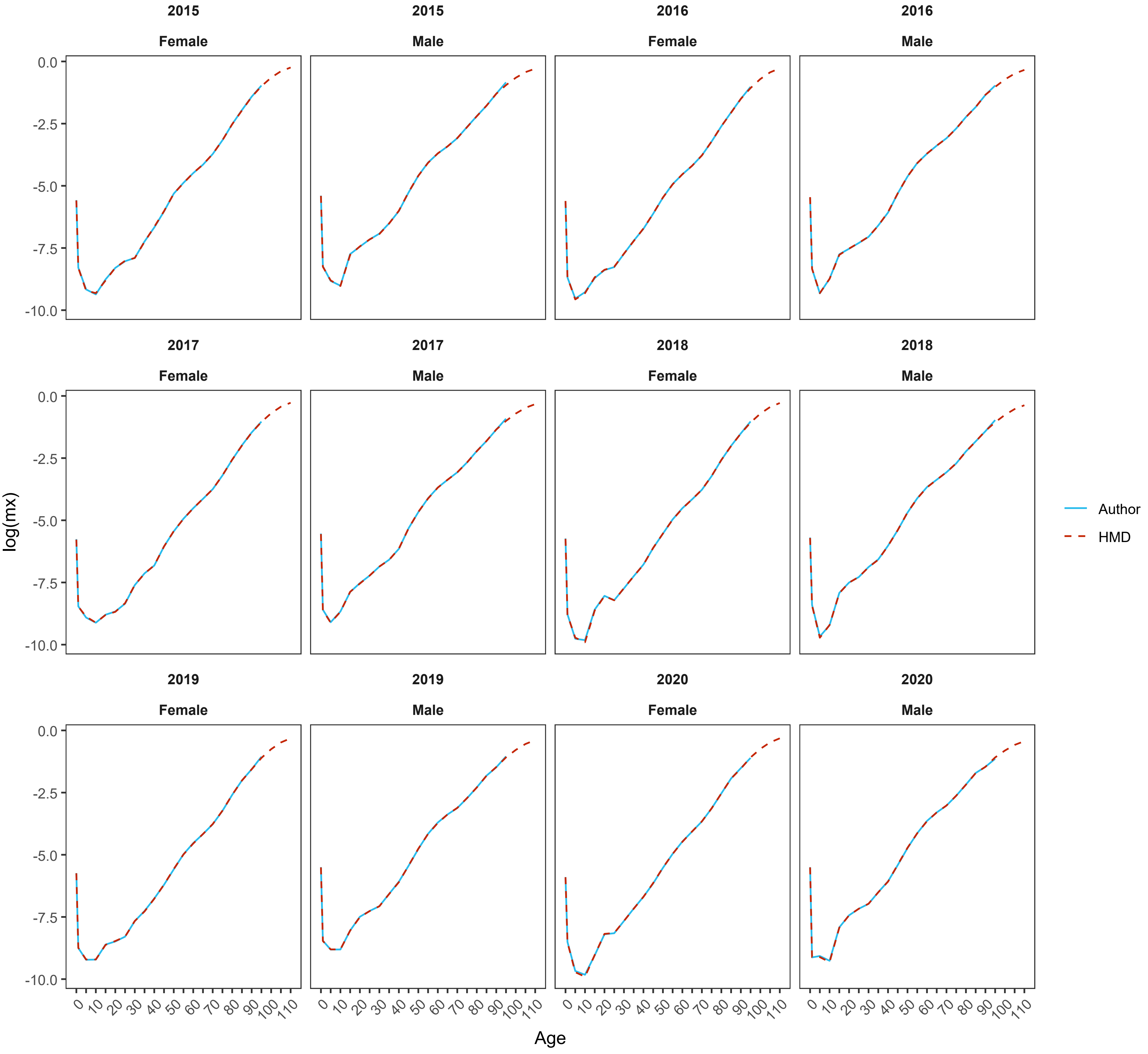

Figure S3k

Comparison of age-group-specific logged mortality rates (Japan), author data set (solid line) vs. HMD (dashed line)

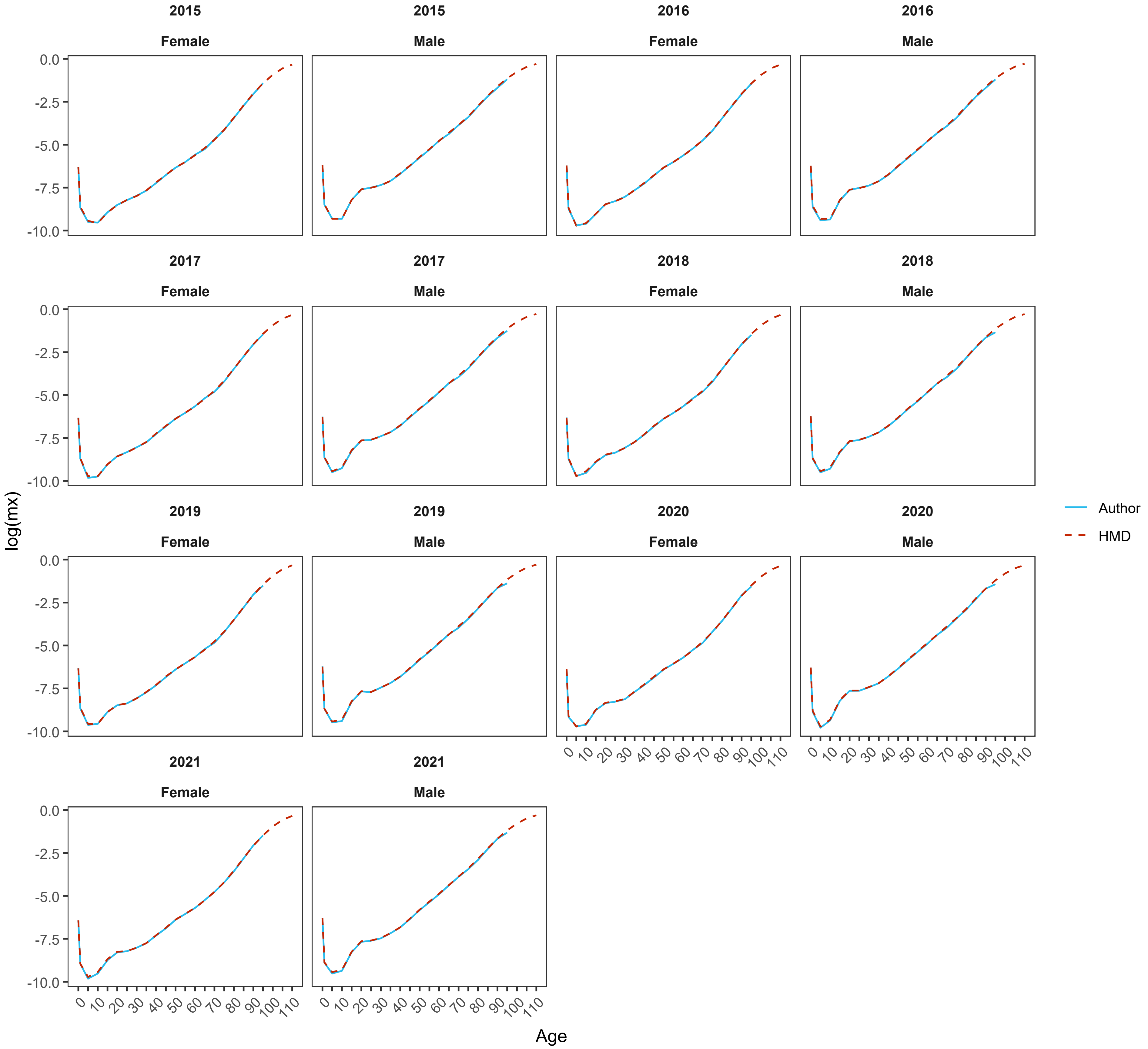

Figure S3l

Comparison of age-group-specific logged mortality rates (Latvia), author data set (solid line) vs. HMD (dashed line)

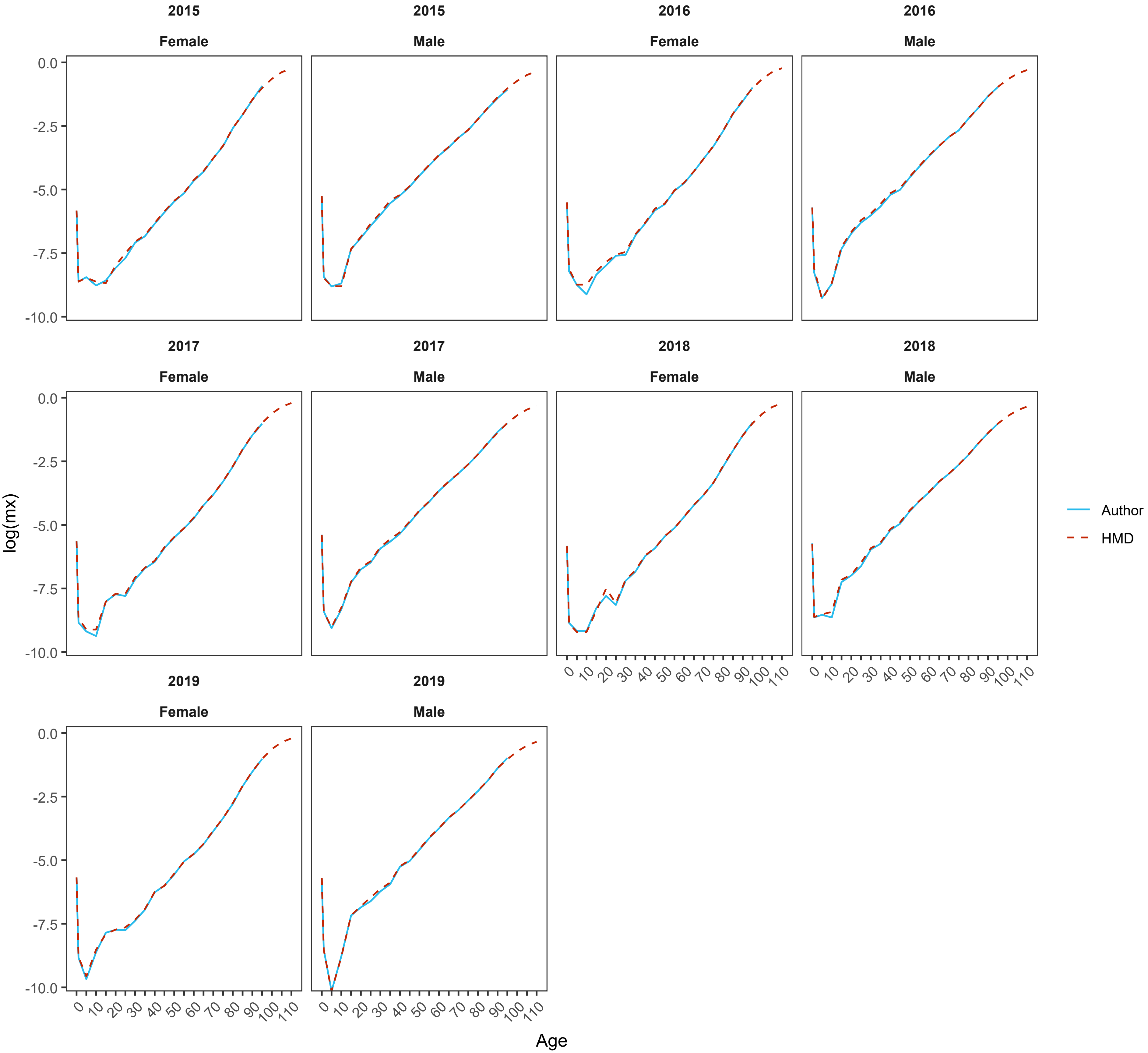

Figure S3m

Comparison of age-group-specific logged mortality rates (Lithuania), author data set (solid line) vs. HMD (dashed line)

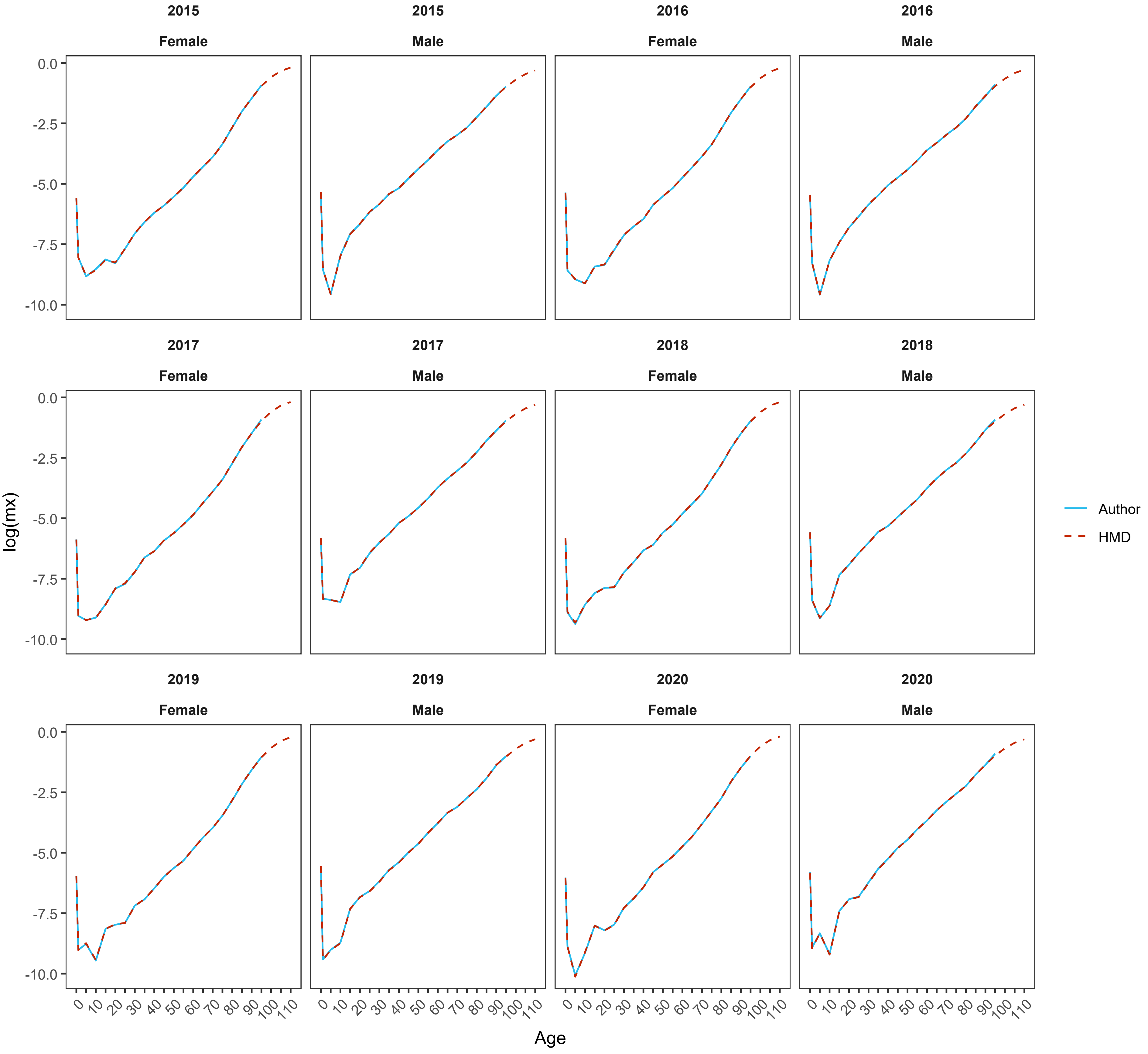

Figure S3n

Comparison of age-group-specific logged mortality rates (Netherlands), author data set (solid line) vs. HMD (dashed line)

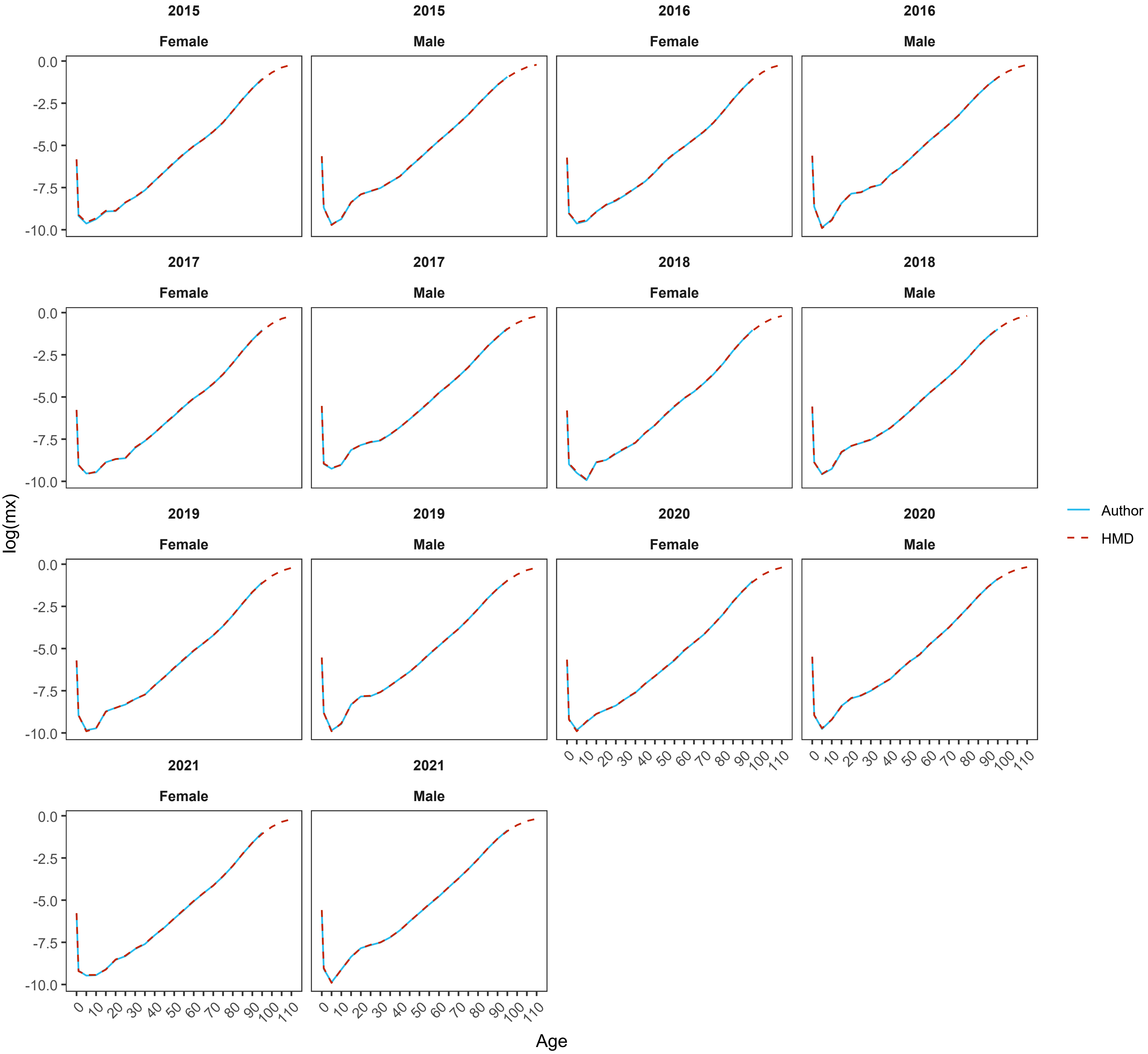

Figure S3o

Comparison of age-group-specific logged mortality rates (Northern Ireland), author data set (solid line) vs. HMD (dashed line)

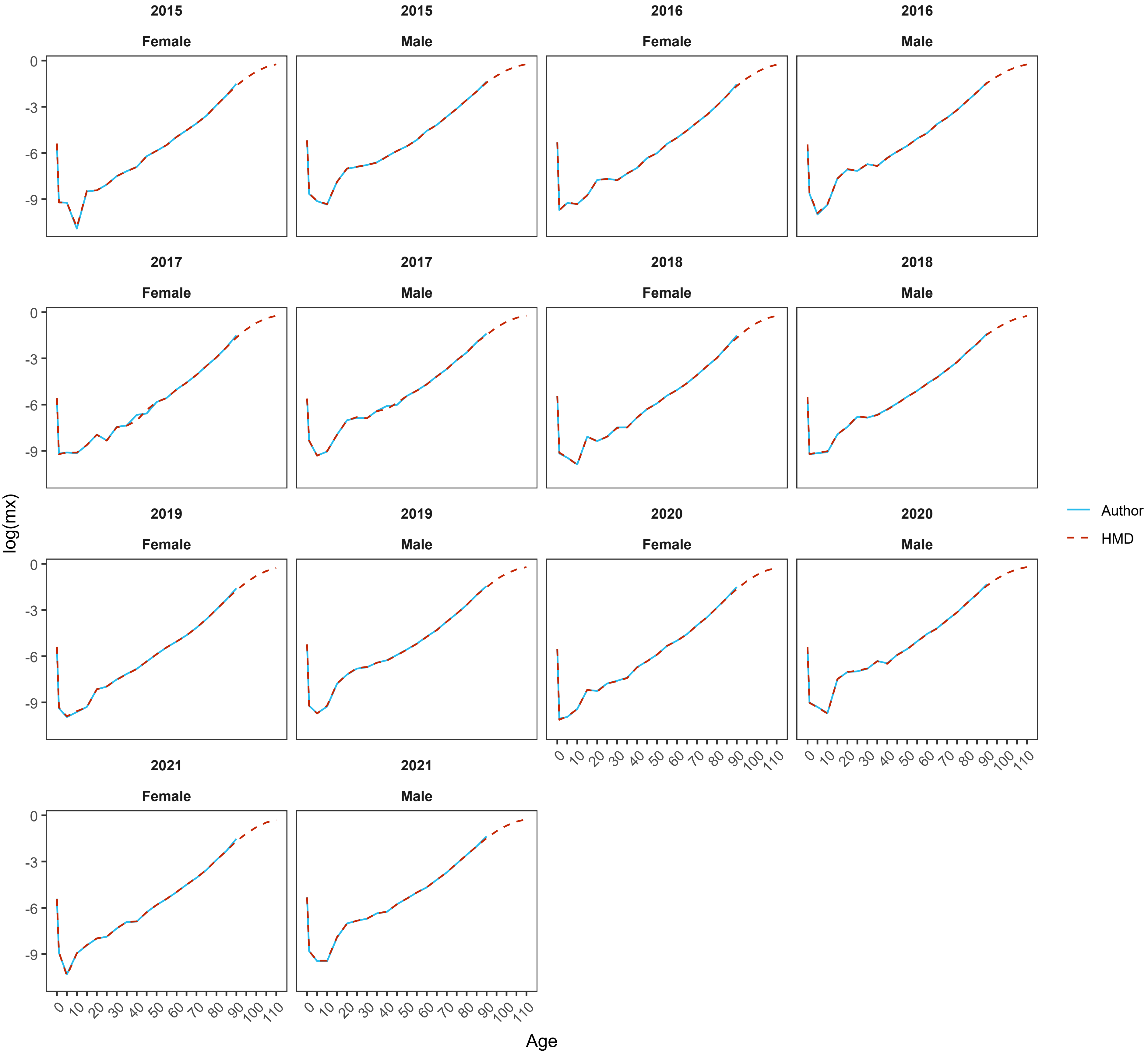

Figure S3p

Comparison of age-group-specific logged mortality rates (Poland),
author data set (solid line) vs. HMD (dashed line)

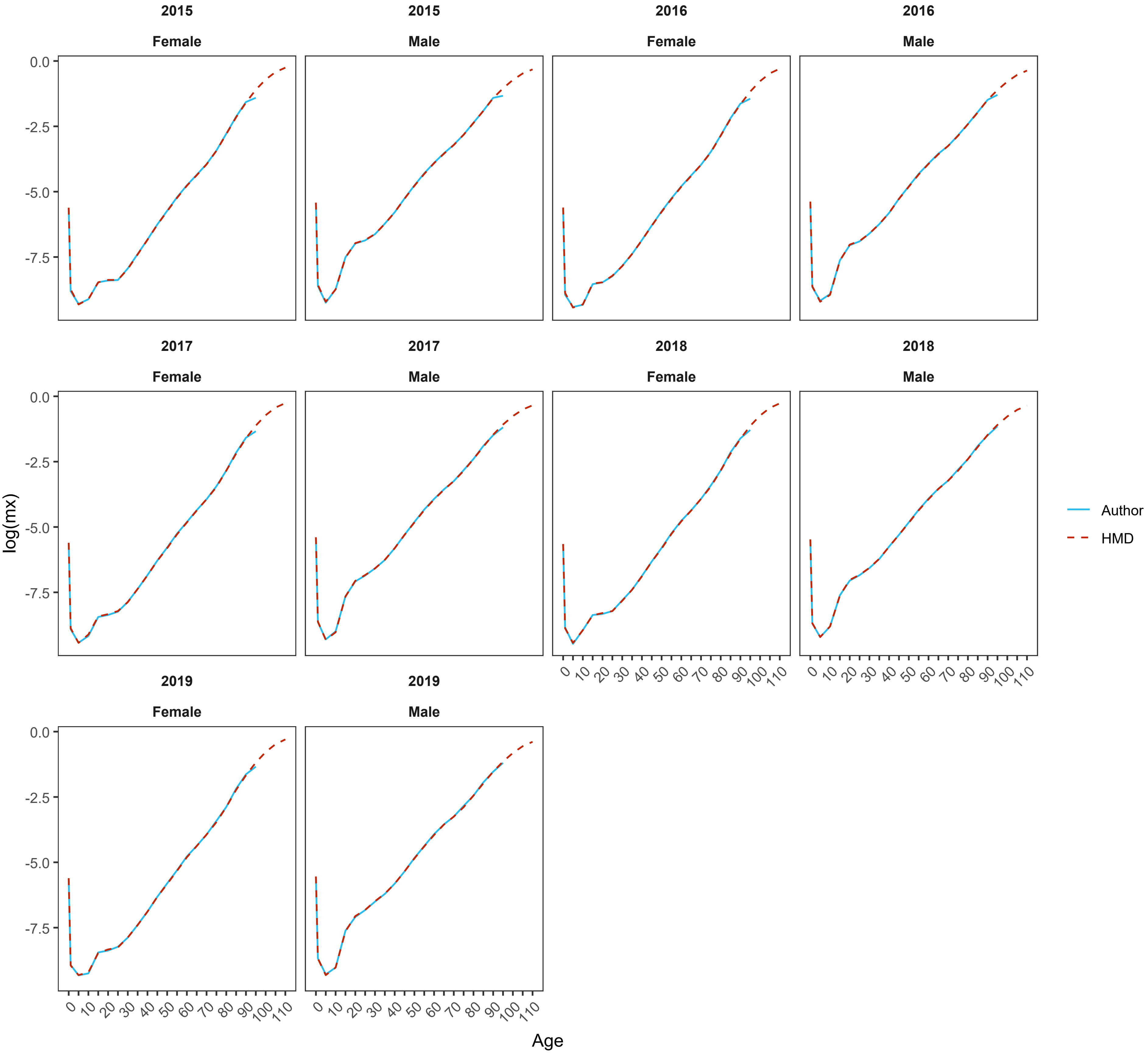

Figure S3q

Comparison of age-group-specific logged mortality rates (Scotland), author data set (solid line) vs. HMD (dashed line)

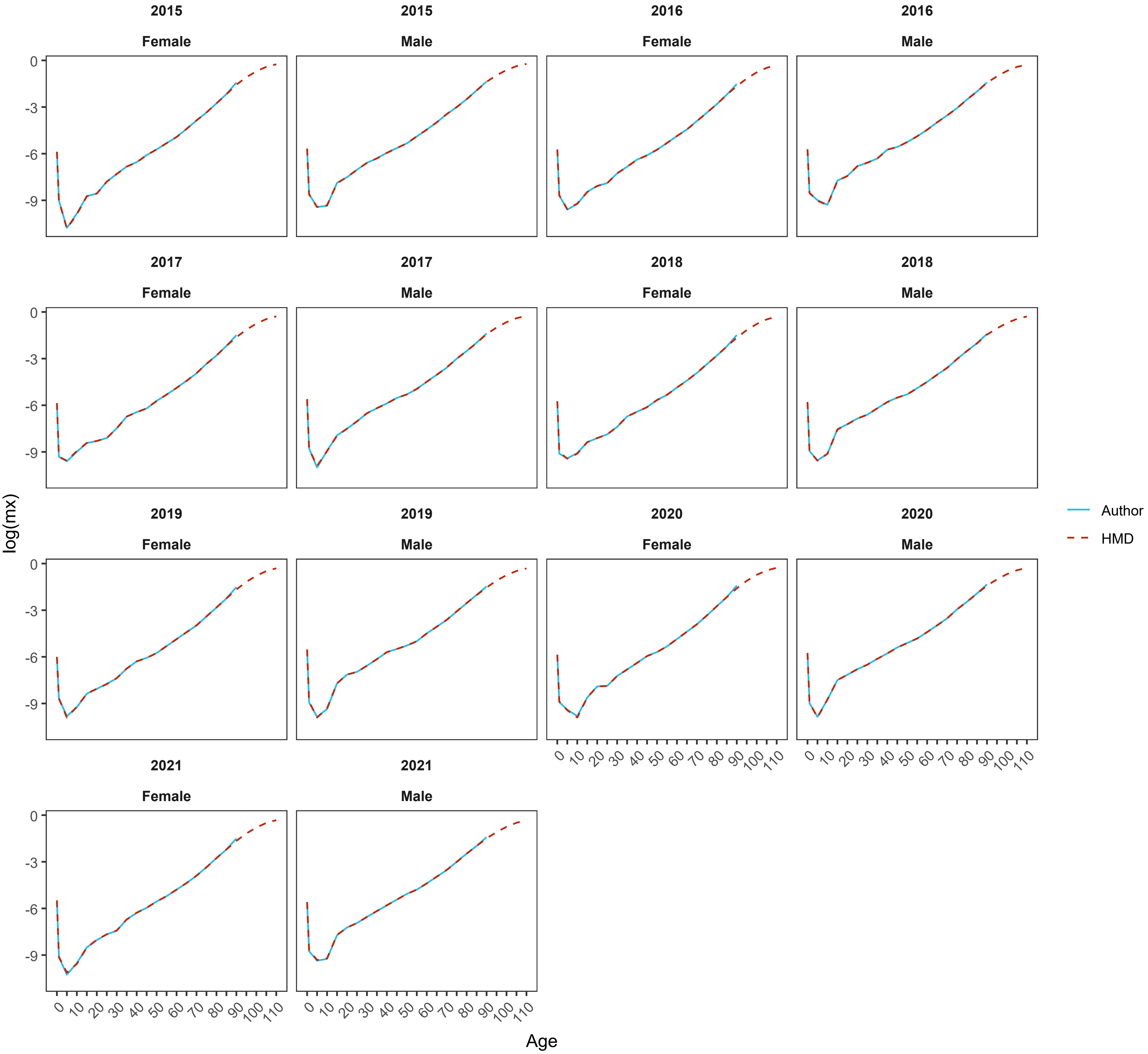

Figure S3r

Comparison of age-group-specific logged mortality rates (South Korea),
author data set (solid line) vs. HMD (dashed line)

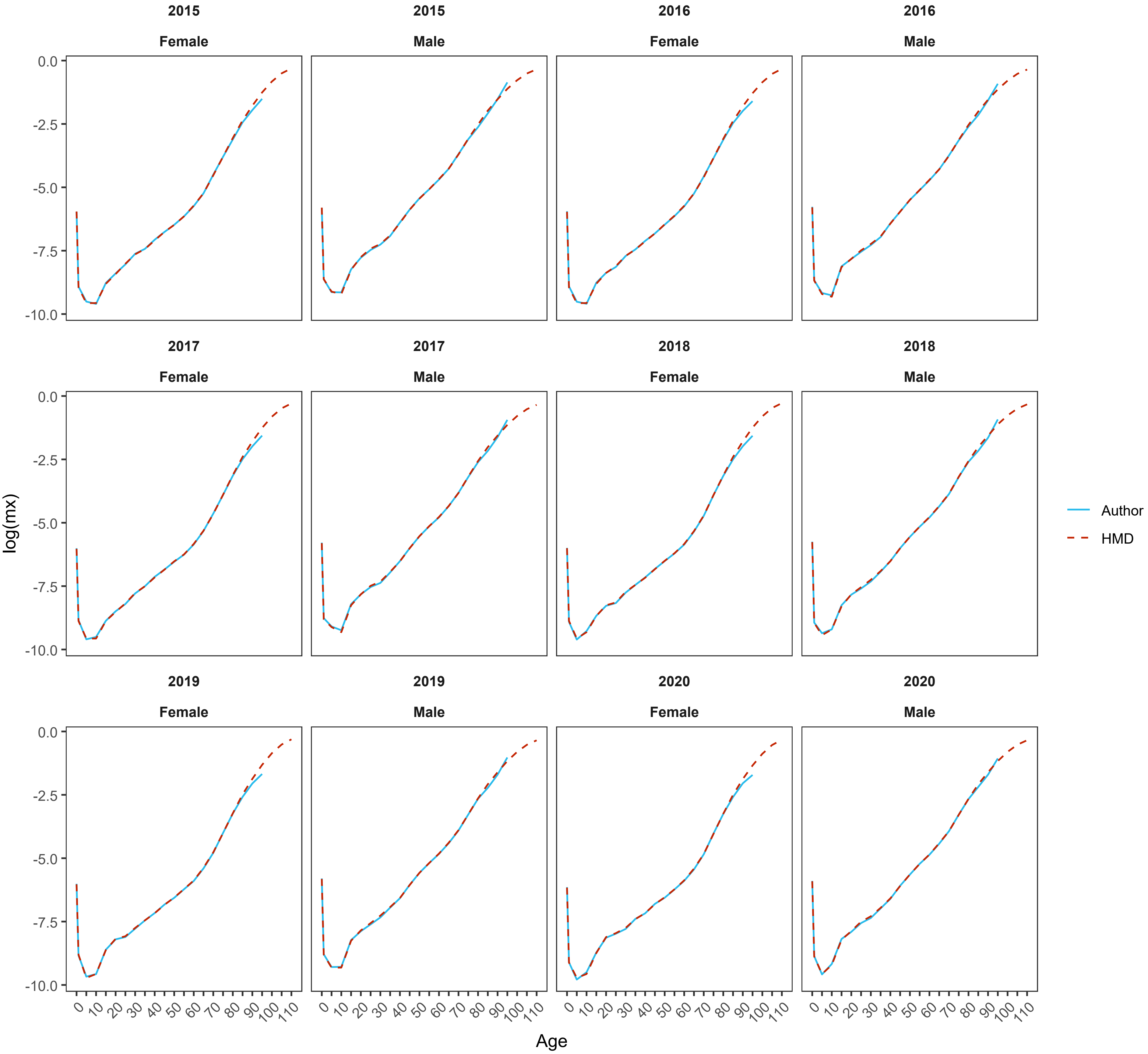

Figure S3s

Comparison of age-group-specific logged mortality rates (Spain), author data set (solid line) vs. HMD (dashed line)

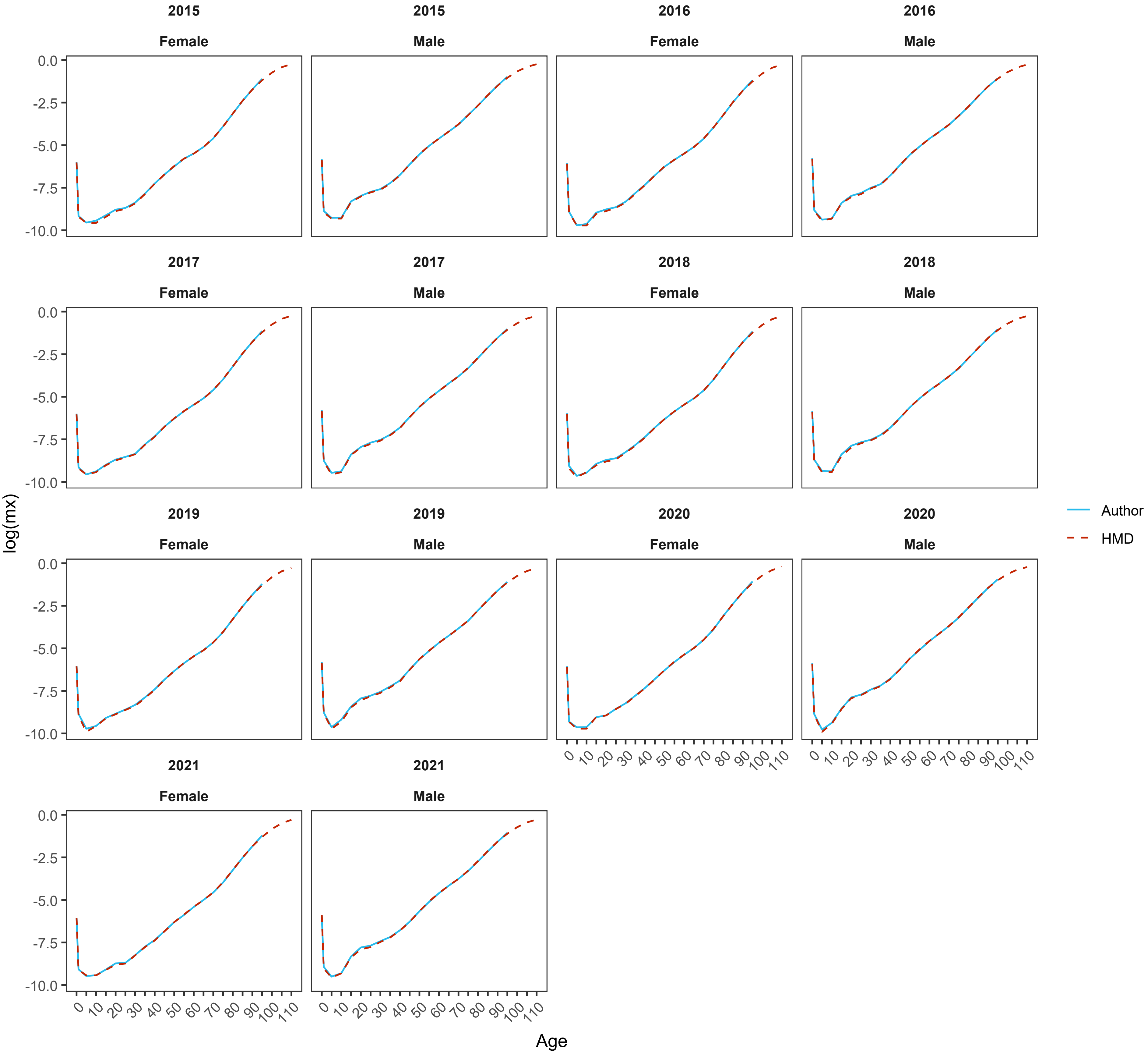

Figure S3t

Comparison of age-group-specific logged mortality rates (Sweden), author data set (solid line) vs. HMD (dashed line)

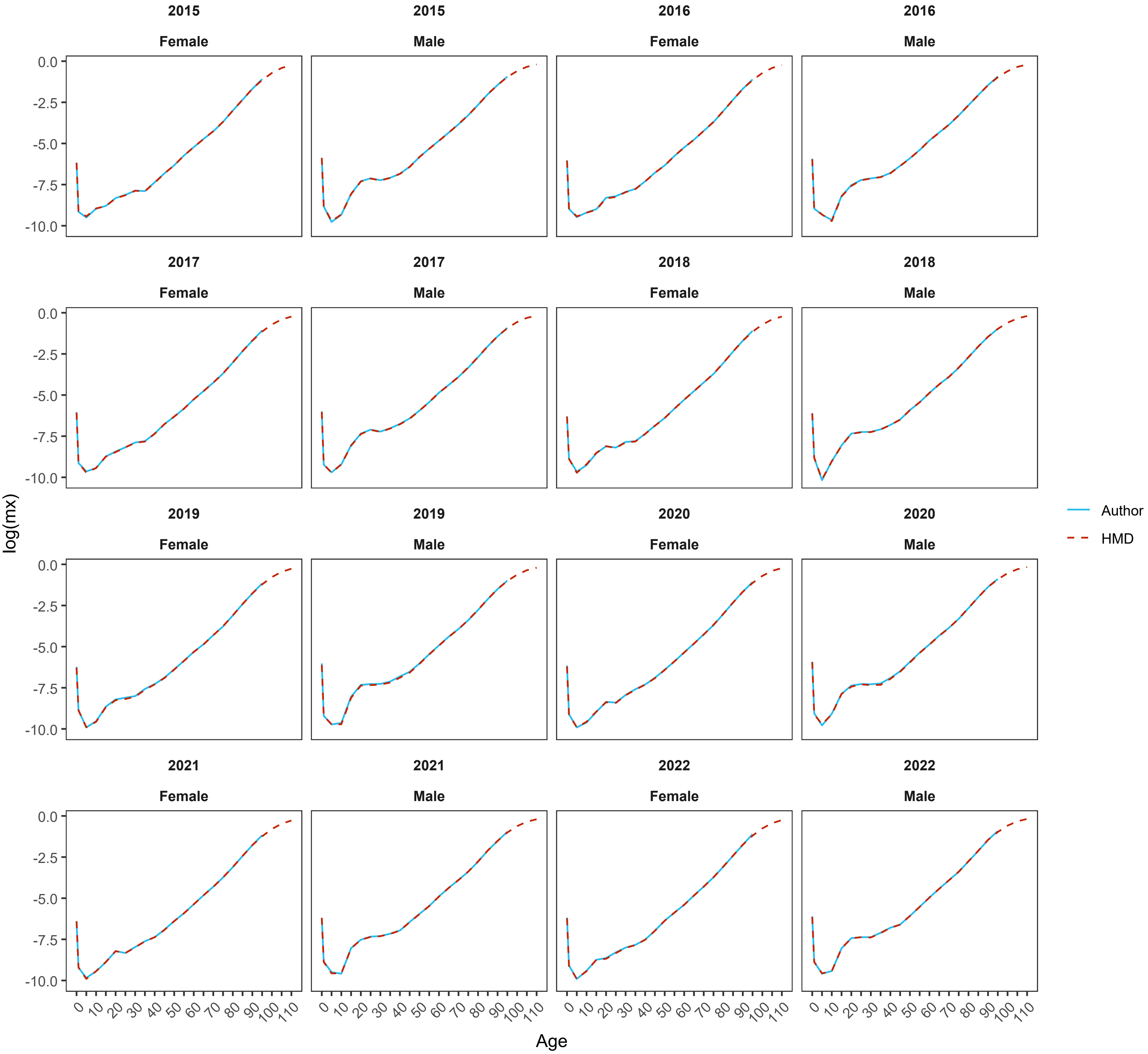

Figure S3u

Comparison of age-group-specific logged mortality rates (Switzerland), author data set (solid line) vs. HMD (dashed line)

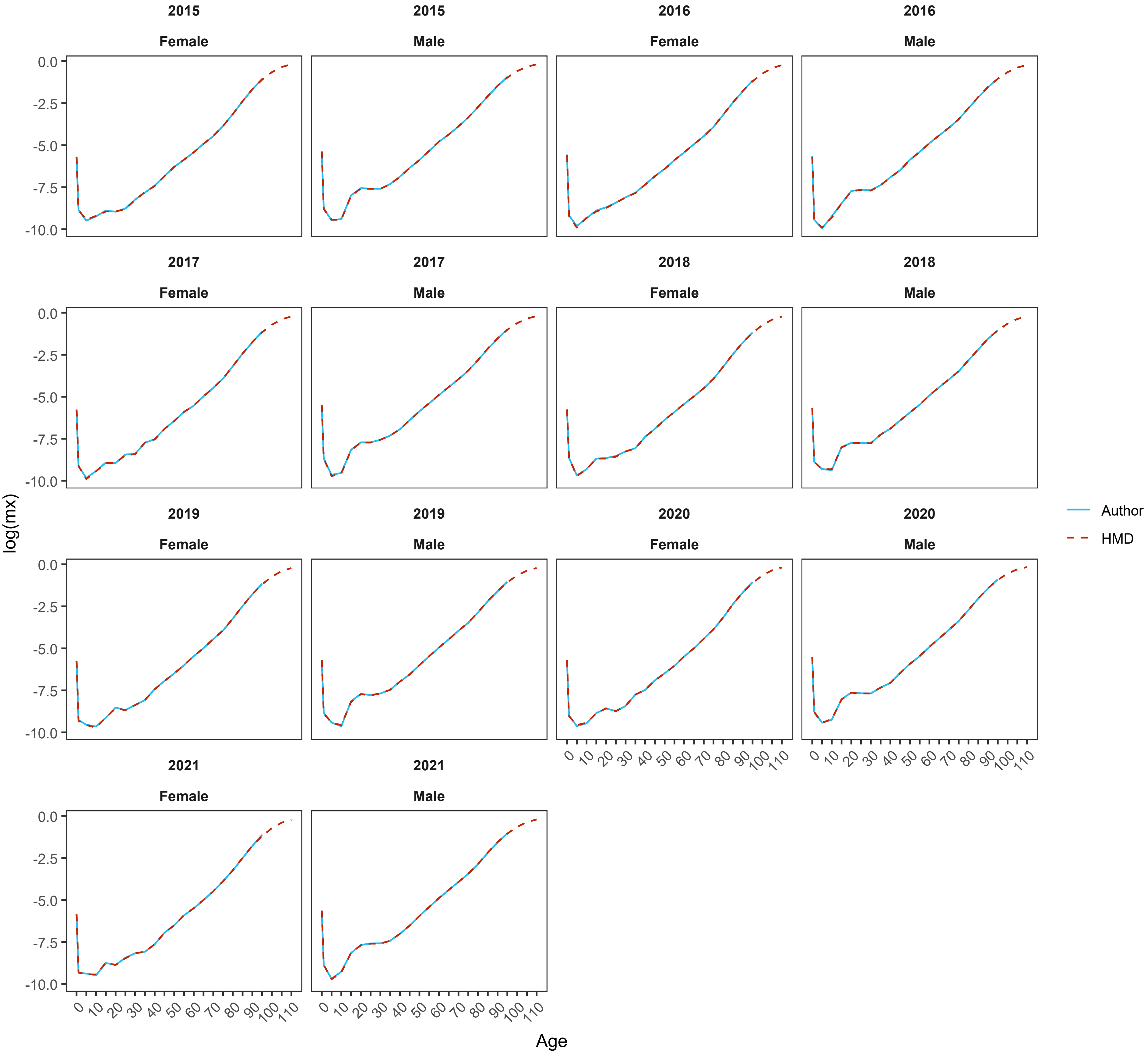

Figure S3v

Comparison of age-group-specific logged mortality rates (USA), author data set (solid line) vs. HMD (dashed line)

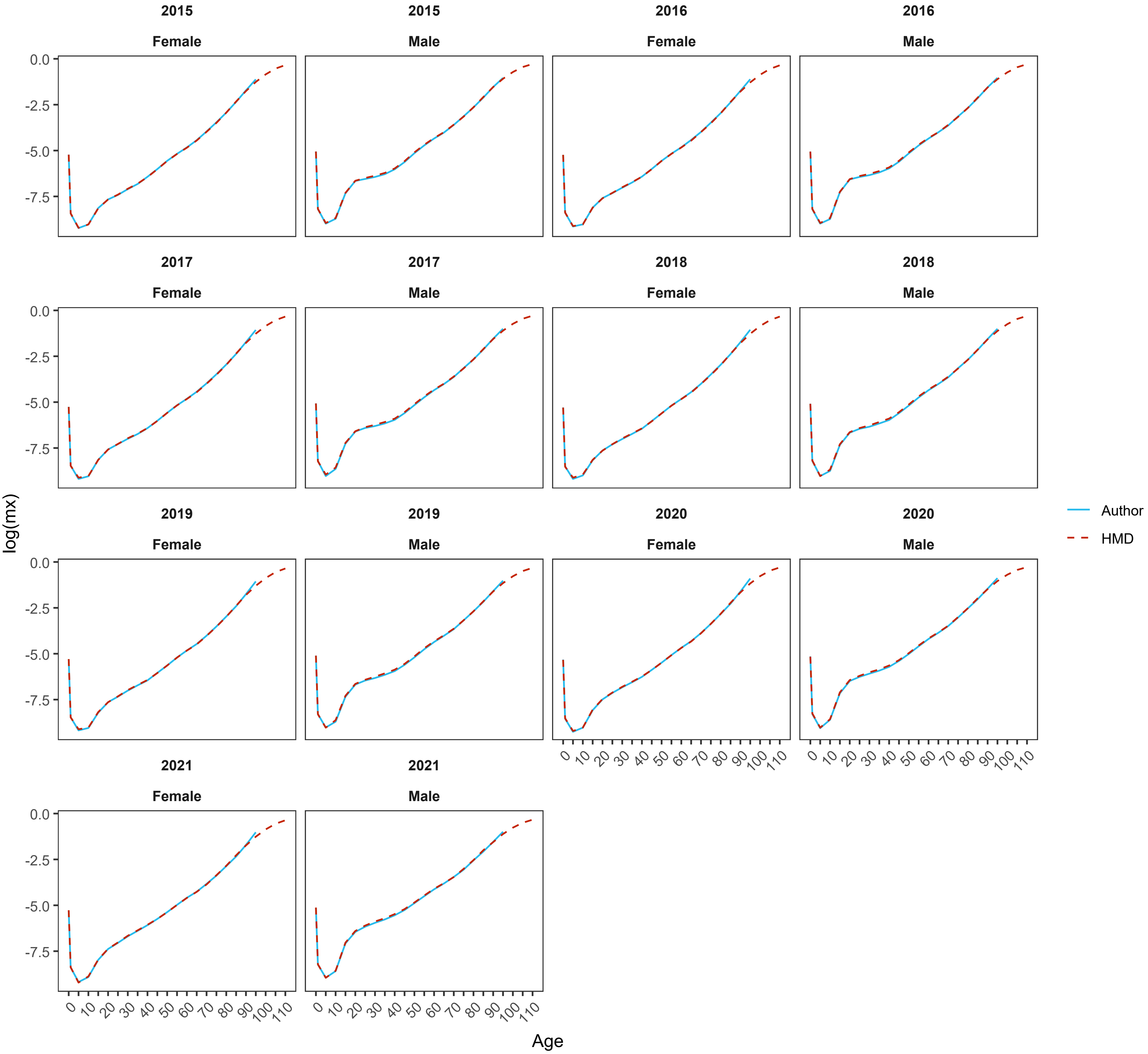

Figure S4a

Comparison of life expectancy at birth (Australia),
author data set (solid line) vs. UNWPP (dashed line)

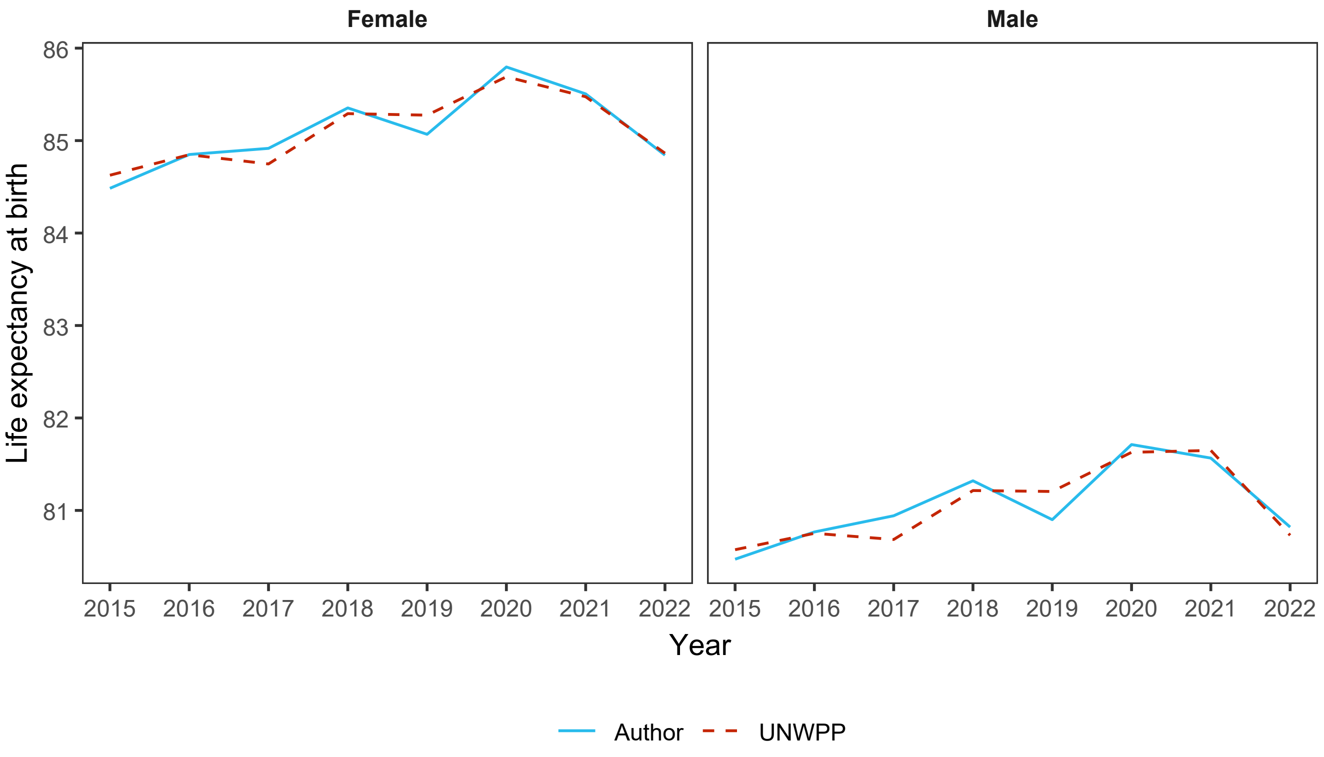

Figure S4b

Comparison of life expectancy at birth (Austria), author data set (solid line) vs. UNWPP (dashed line)

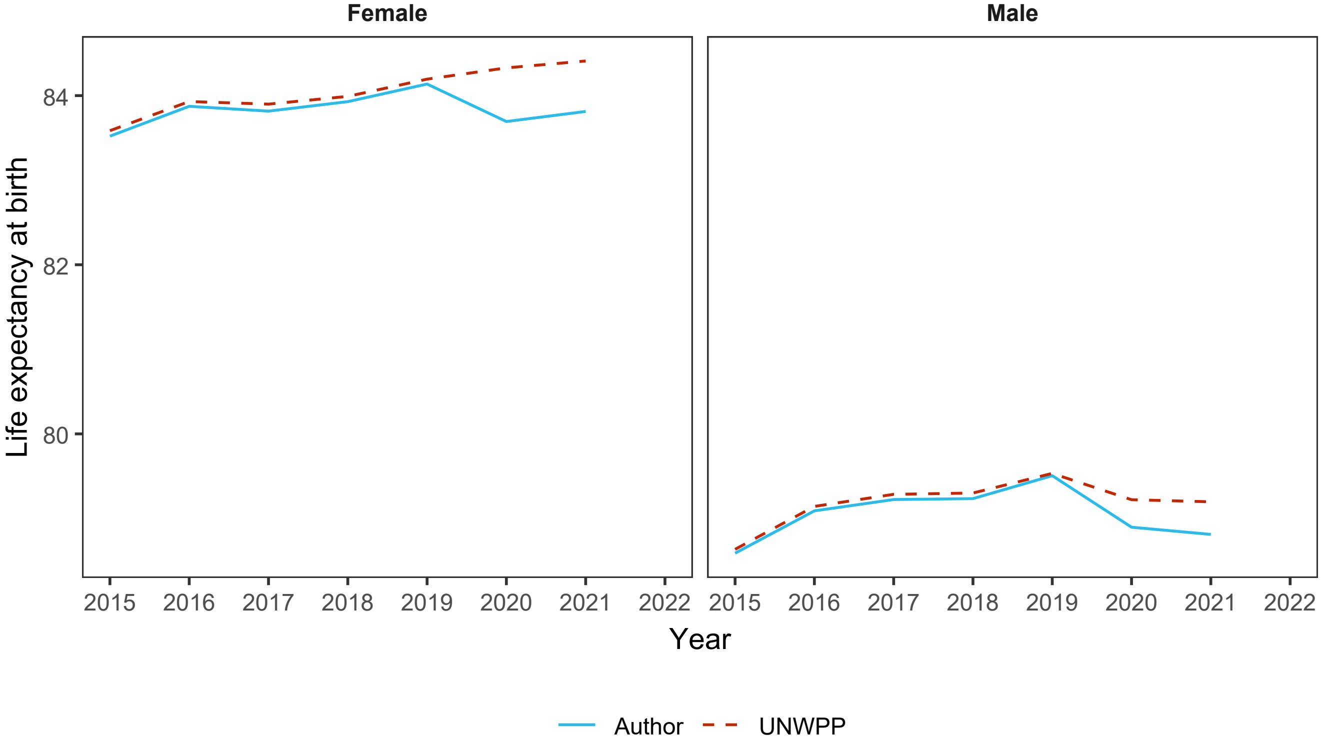

Figure S4c

Comparison of life expectancy at birth (Brazil),
author data set (solid line) vs. UNWPP (dashed line)

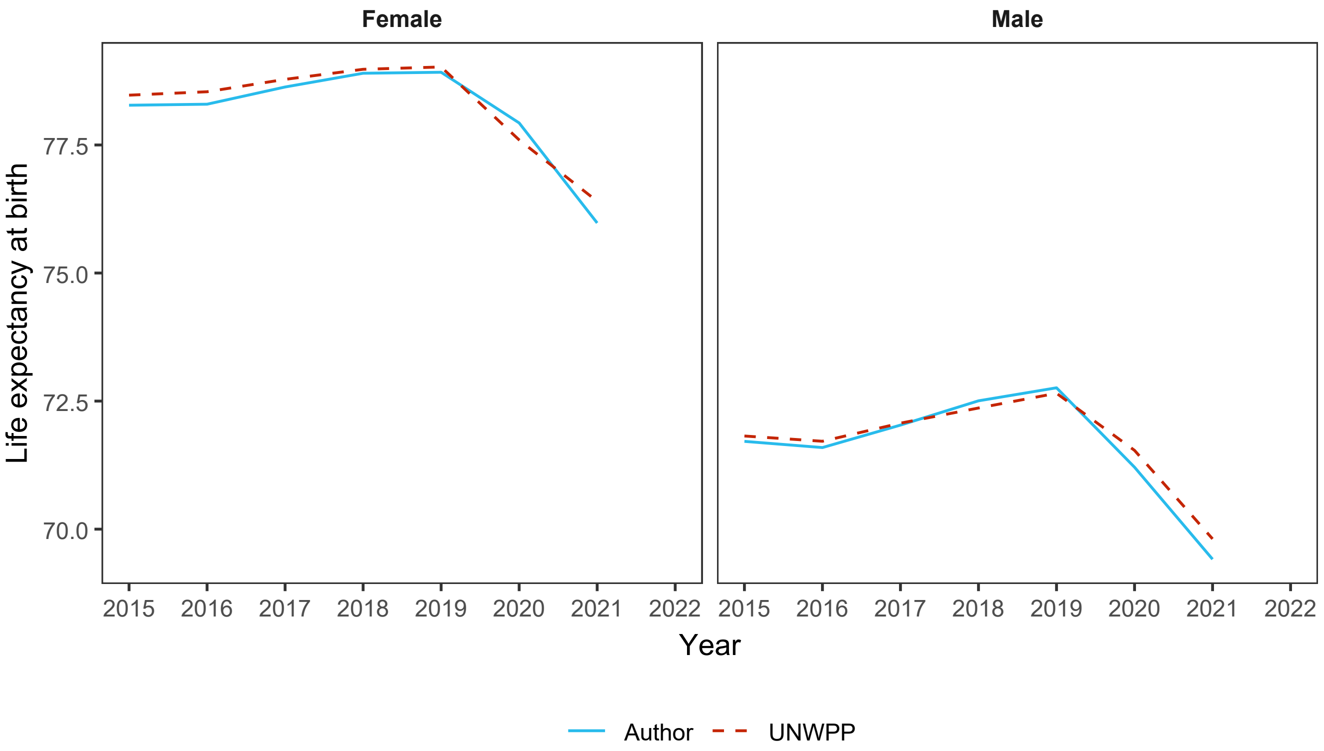

Figure S4d

Comparison of life expectancy at birth (Bulgaria),
author data set (solid line) vs. UNWPP (dashed line)

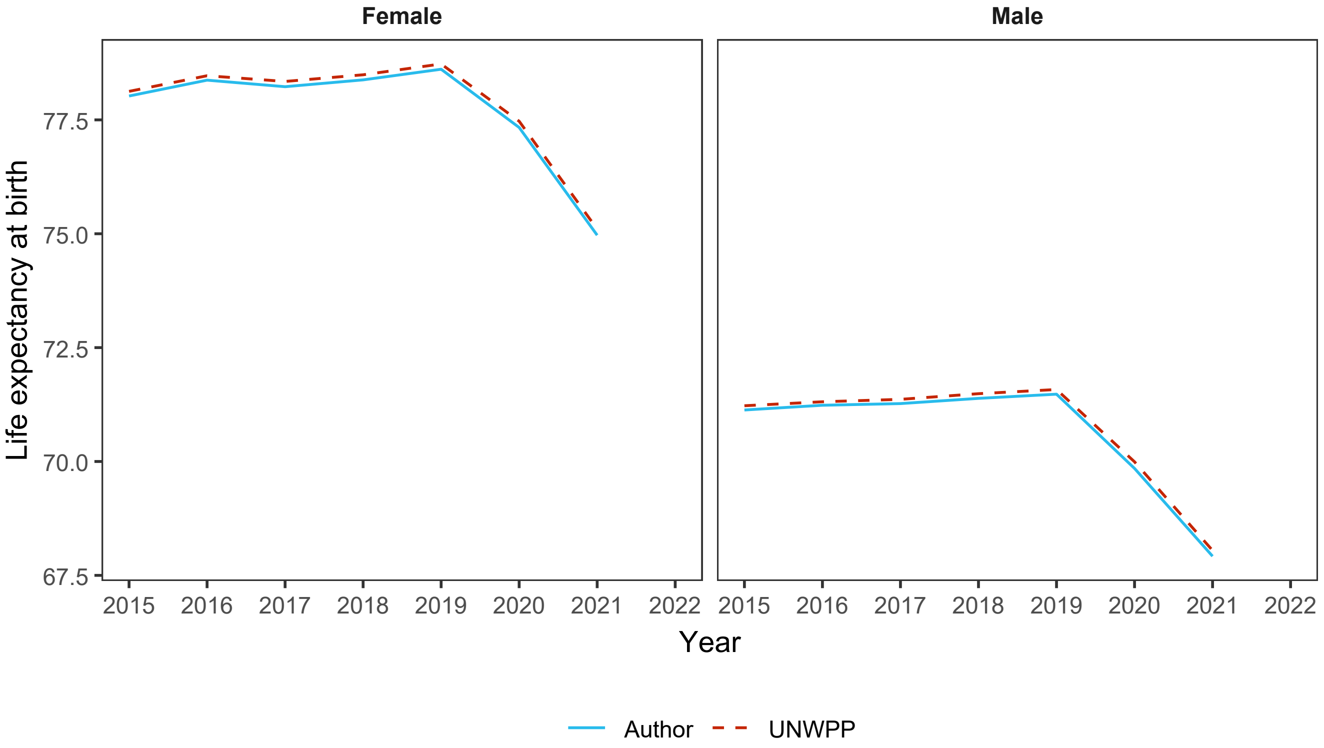

Figure S4e

Comparison of life expectancy at birth (Canada),
author data set (solid line) vs. UNWPP (dashed line)

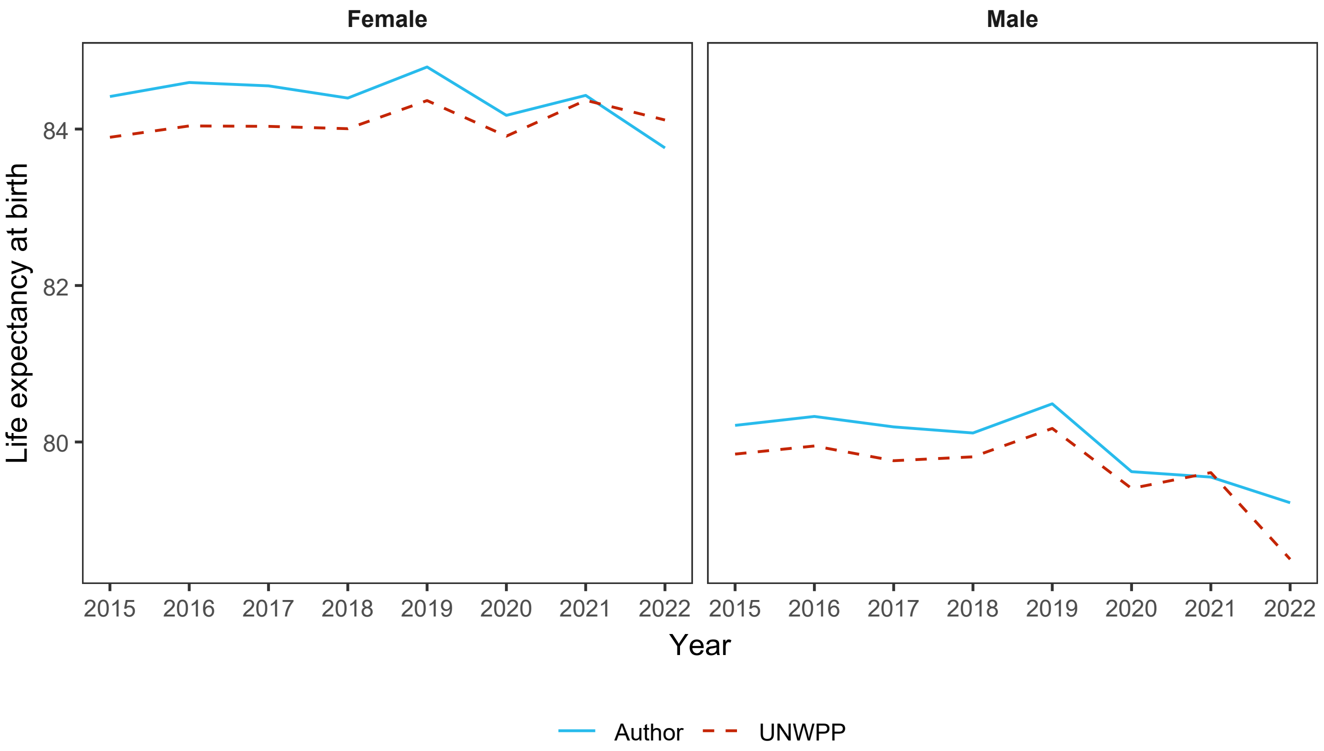

Figure S4f

Comparison of life expectancy at birth (Chile),
author data set (solid line) vs. UNWPP (dashed line)

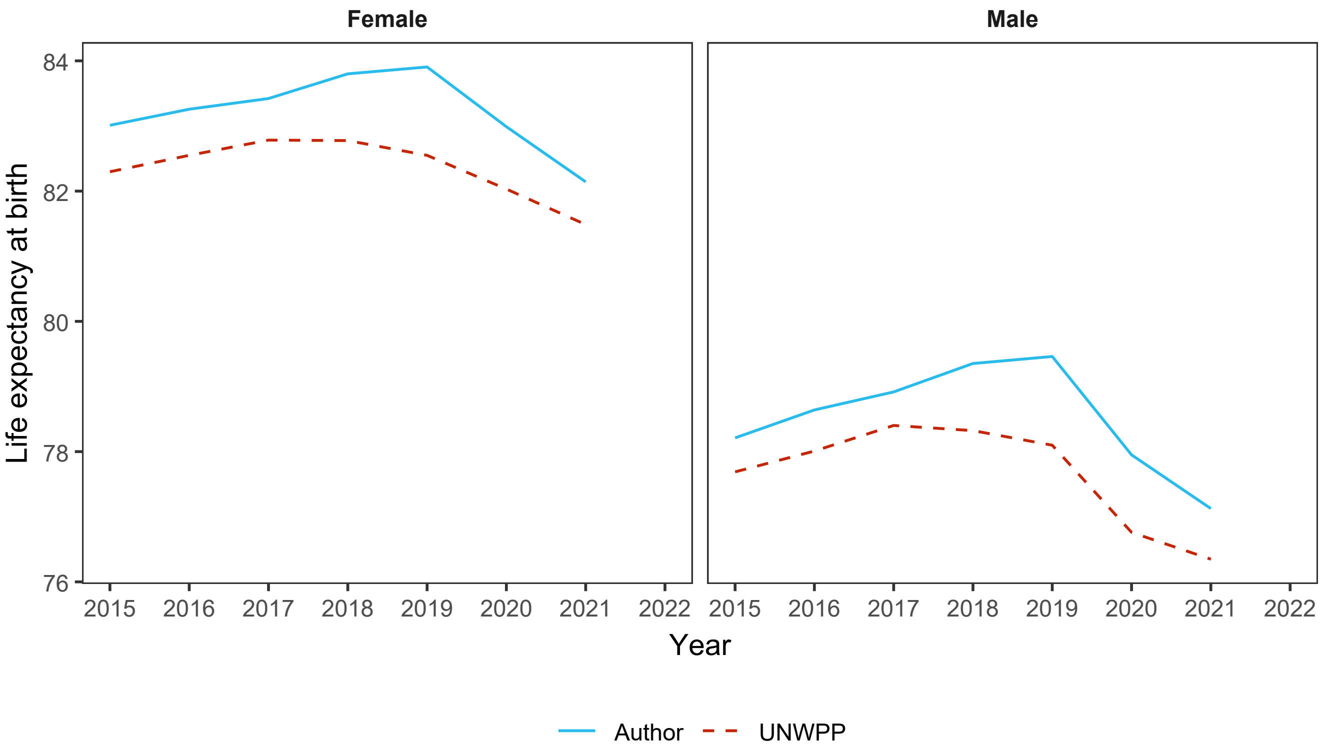

Figure S4g

Comparison of life expectancy at birth (Croatia), author data set (solid line) vs. UNWPP (dashed line)

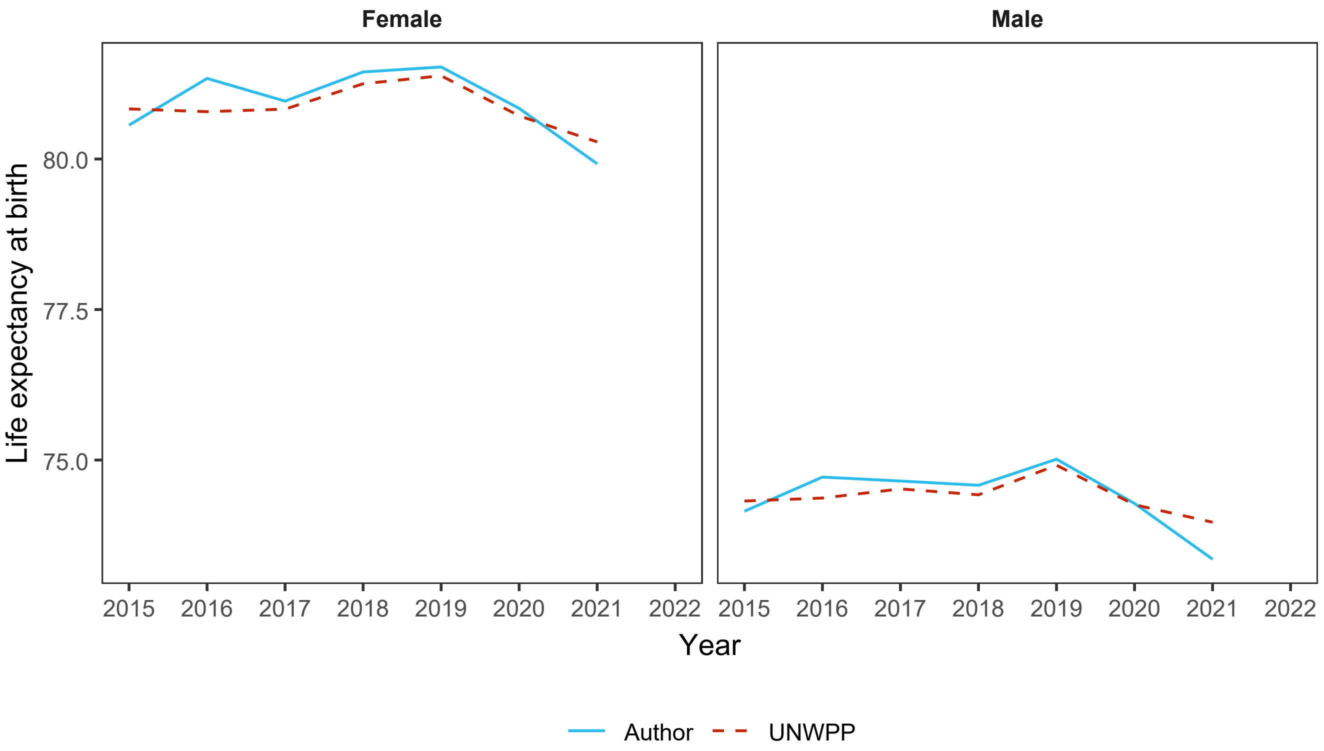

Figure S4h

Comparison of life expectancy at birth (Czechia), author data set (solid line) vs. UNWPP (dashed line)

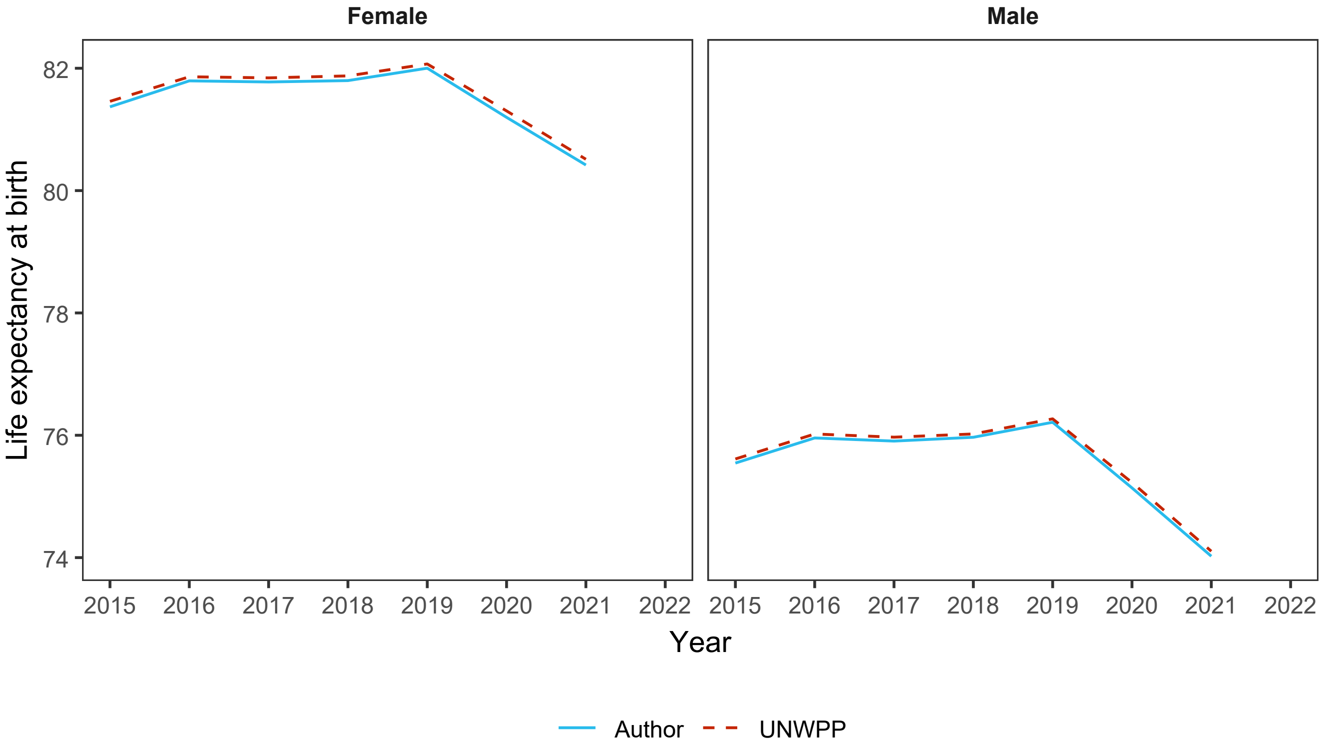

Figure S4i

Comparison of life expectancy at birth (Denmark), author data set (solid line) vs. UNWPP (dashed line)

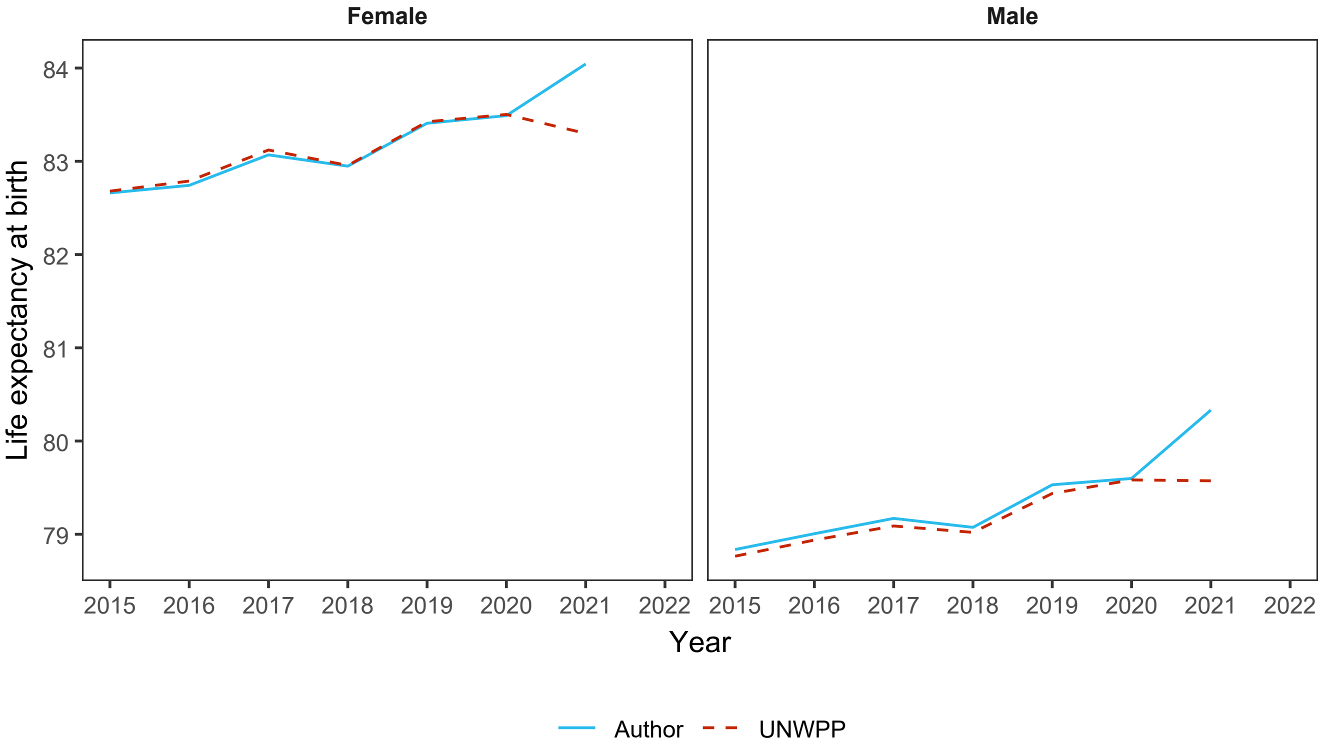

Figure S4j

Comparison of life expectancy at birth (Hungary),
author data set (solid line) vs. UNWPP (dashed line)

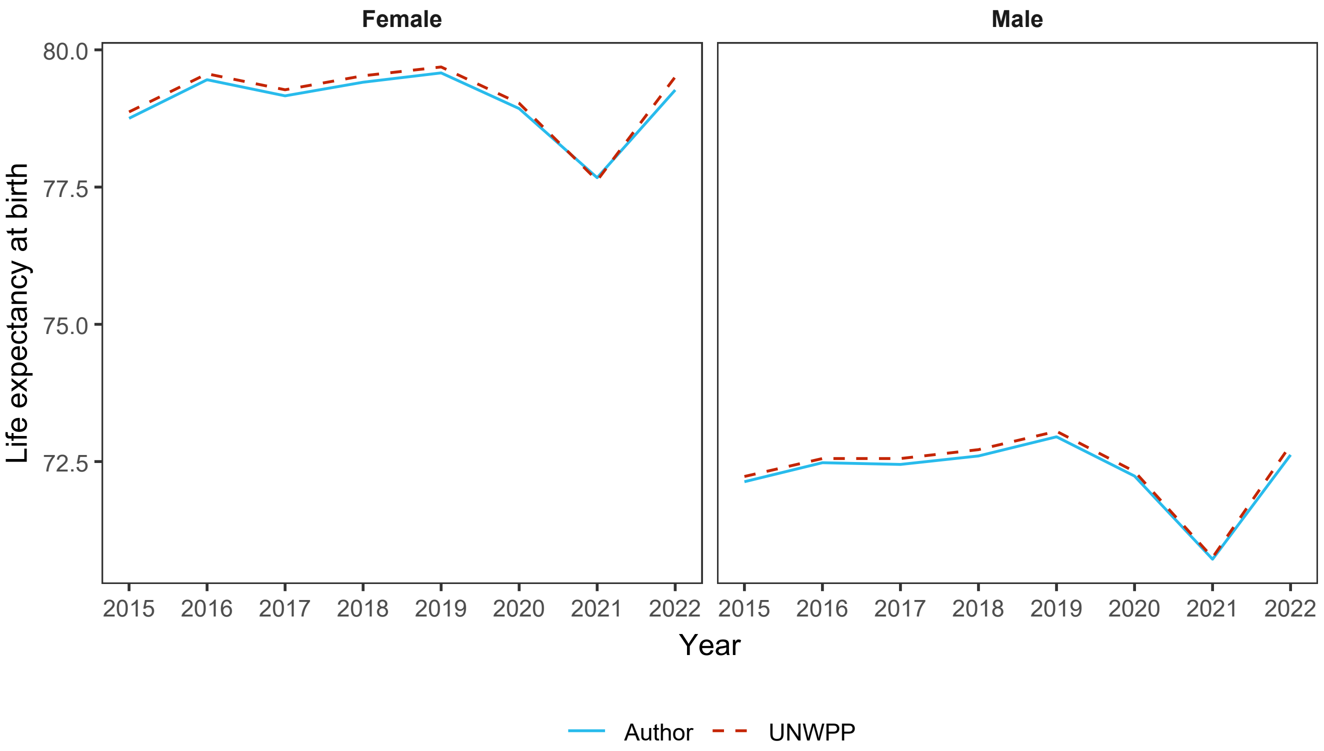

Figure S4k

Comparison of life expectancy at birth (Japan),
author data set (solid line) vs. UNWPP (dashed line)

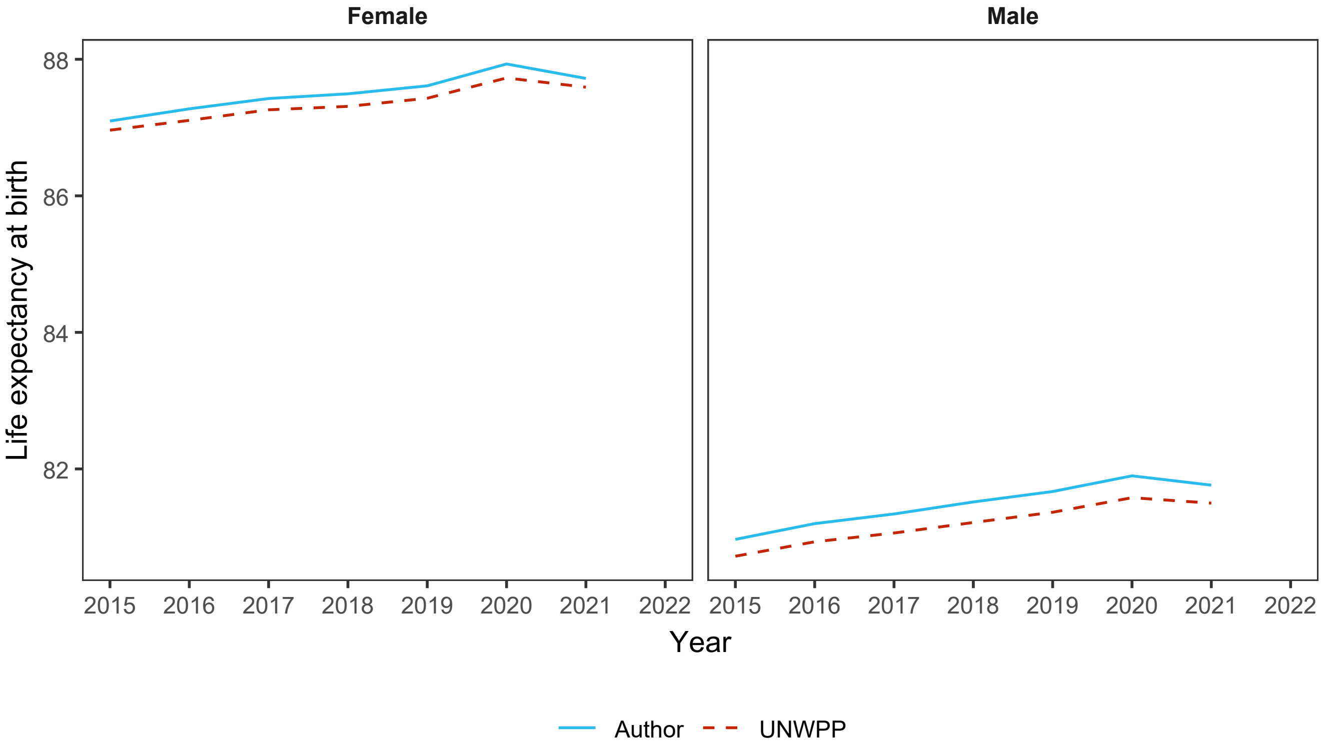

Figure S4I

Comparison of life expectancy at birth (Latvia),
author data set (solid line) vs. UNWPP (dashed line)

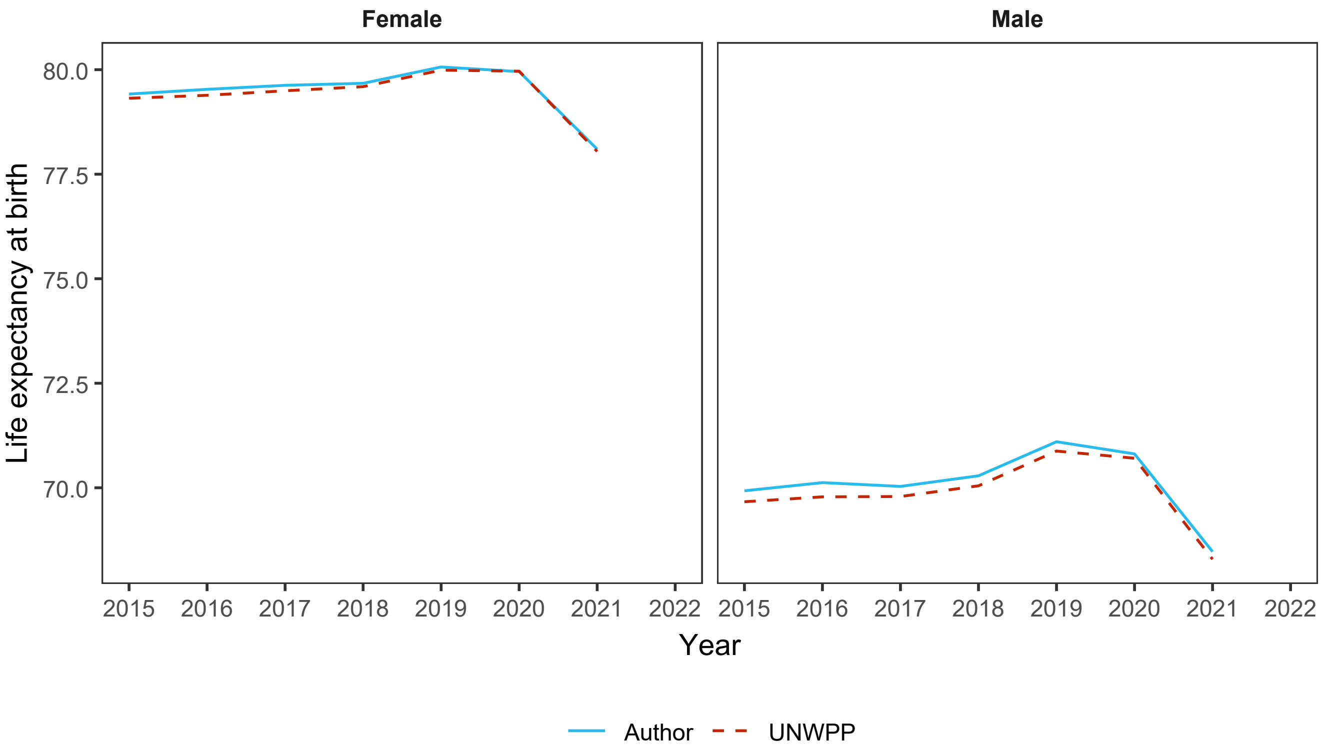

Figure S4m

Comparison of life expectancy at birth (Lithuania), author data set (solid line) vs. UNWPP (dashed line)

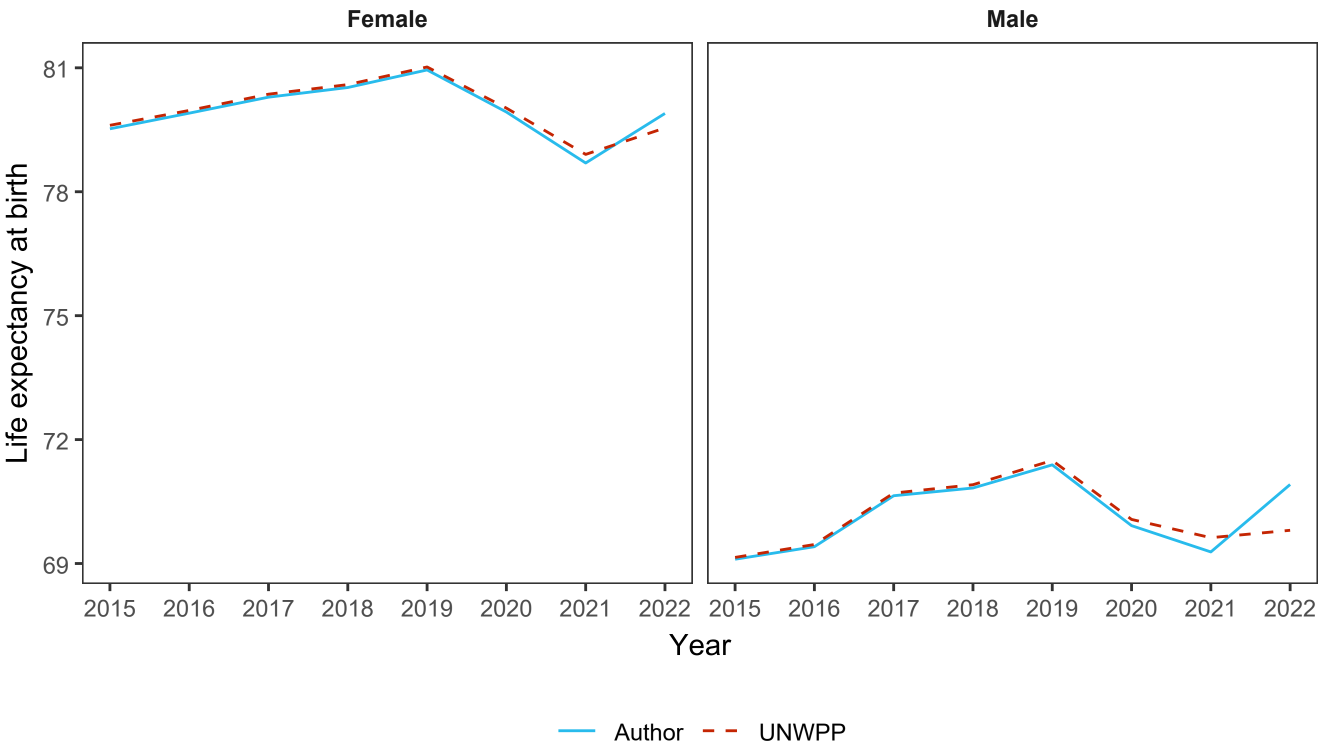

Figure S4n

Comparison of life expectancy at birth (Netherlands),
author data set (solid line) vs. UNWPP (dashed line)

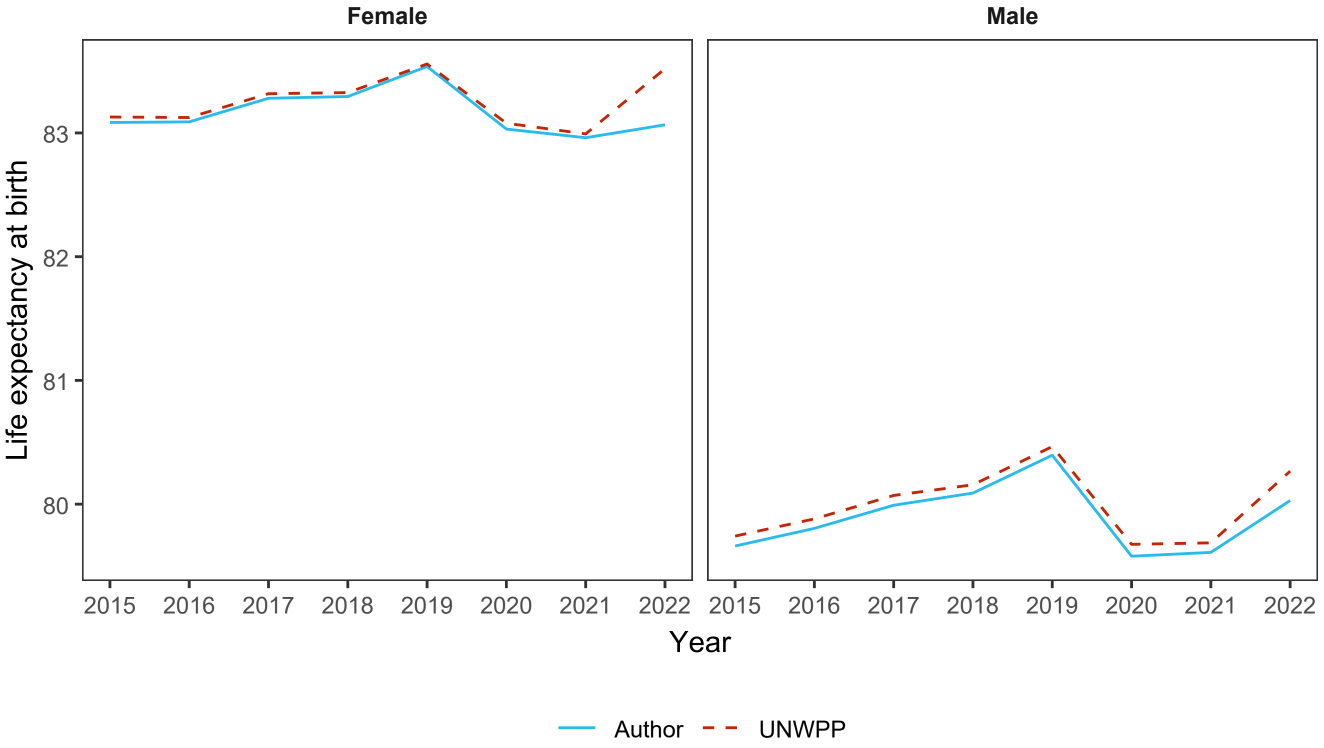

Figure S4o

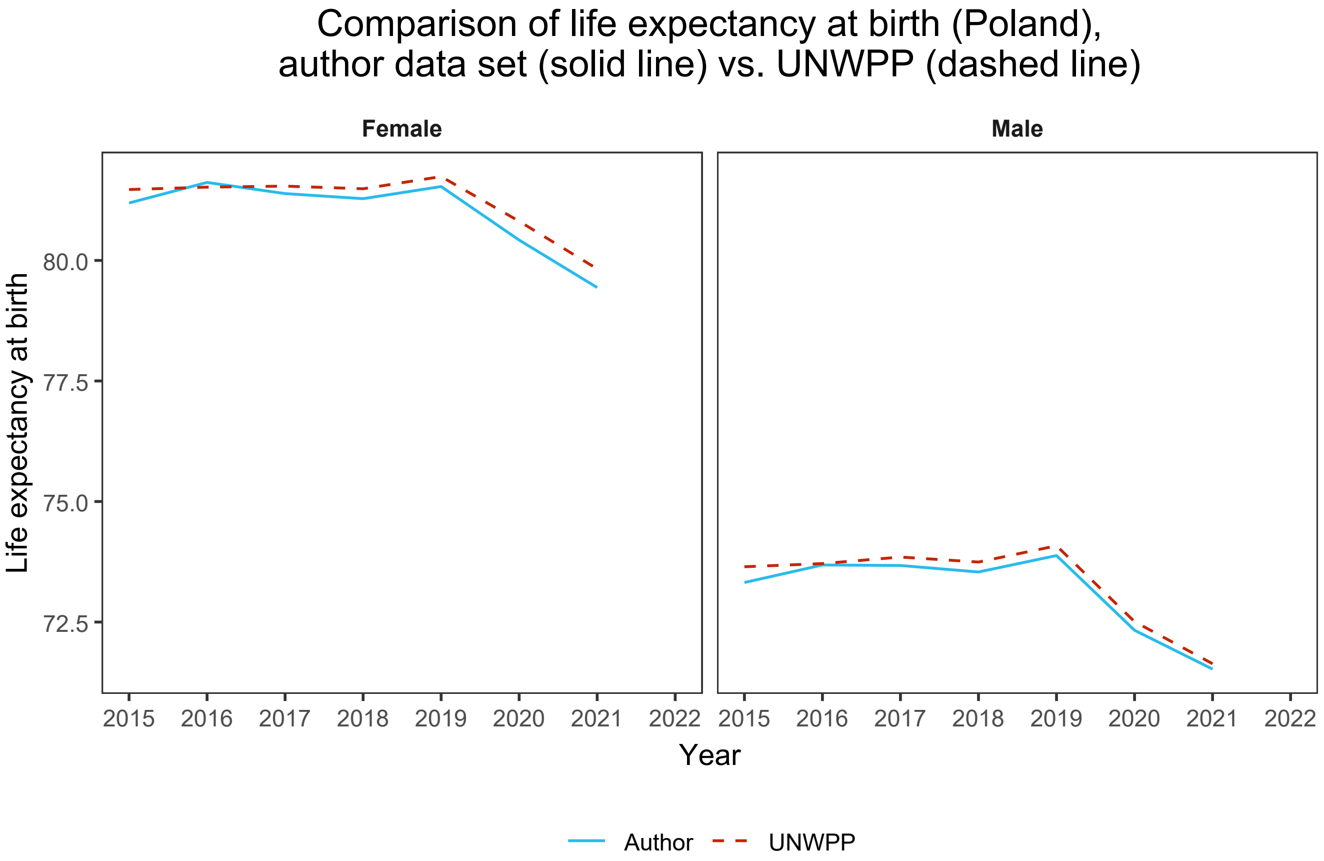
Comparison of life expectancy at birth (Poland),
author data set (solid line) vs. UNWPP (dashed line)
Female
Male
80.0
77.5
75.0
72.5
Life expectancy at birth
2015
2016
2017
2018
2019
2020
2021
2022
Year
Author
UNWPP

Figure S4p

Comparison of life expectancy at birth (Russia), author data set (solid line) vs. UNWPP (dashed line)

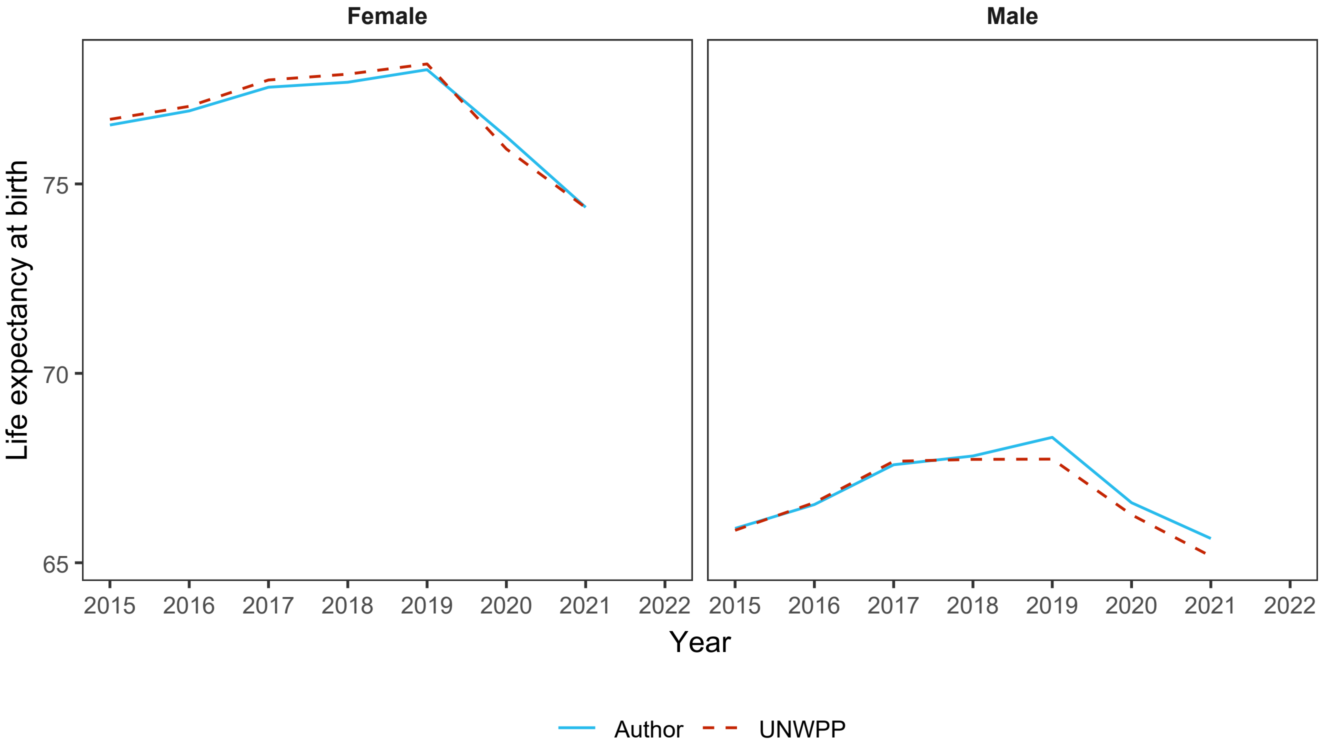

Figure S4q

Comparison of life expectancy at birth (South Korea), author data set (solid line) vs. UNWPP (dashed line)

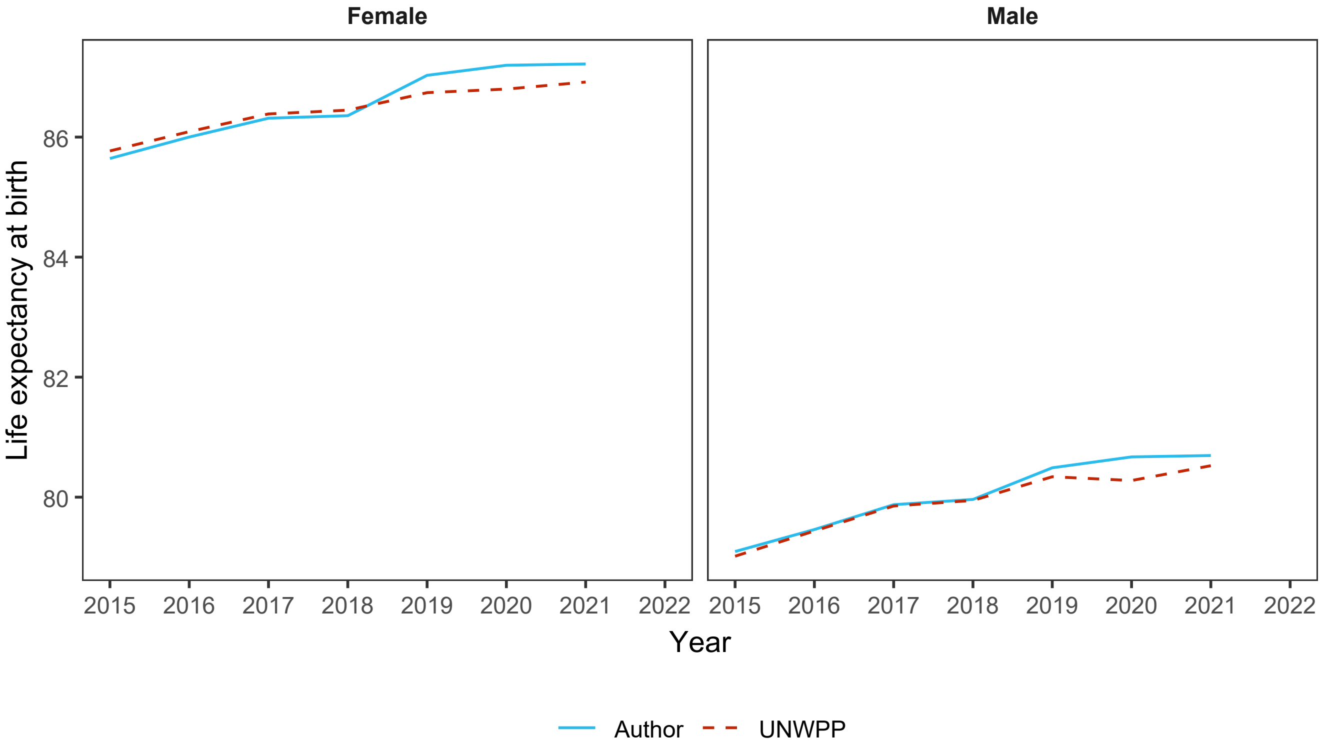

Figure S4r

Comparison of life expectancy at birth (Spain),
author data set (solid line) vs. UNWPP (dashed line)

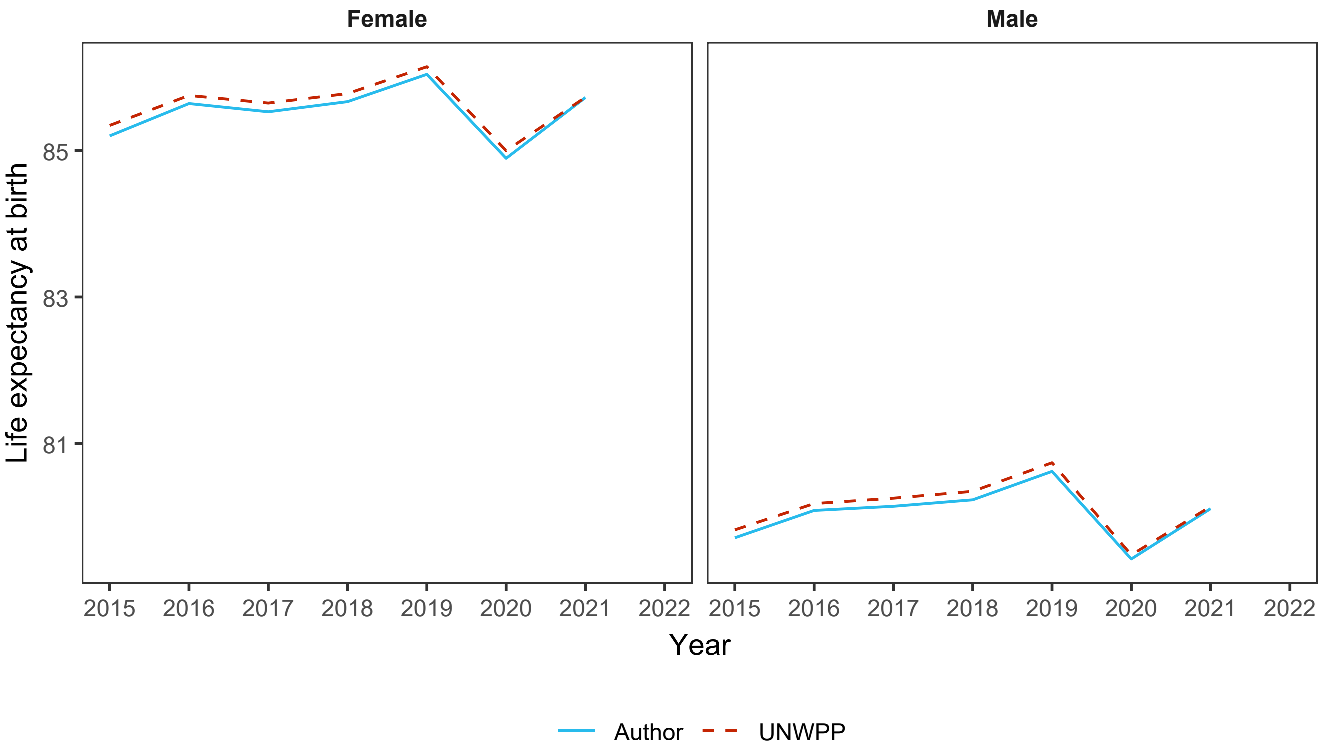

Figure S4s

Comparison of life expectancy at birth (Sweden),
author data set (solid line) vs. UNWPP (dashed line)

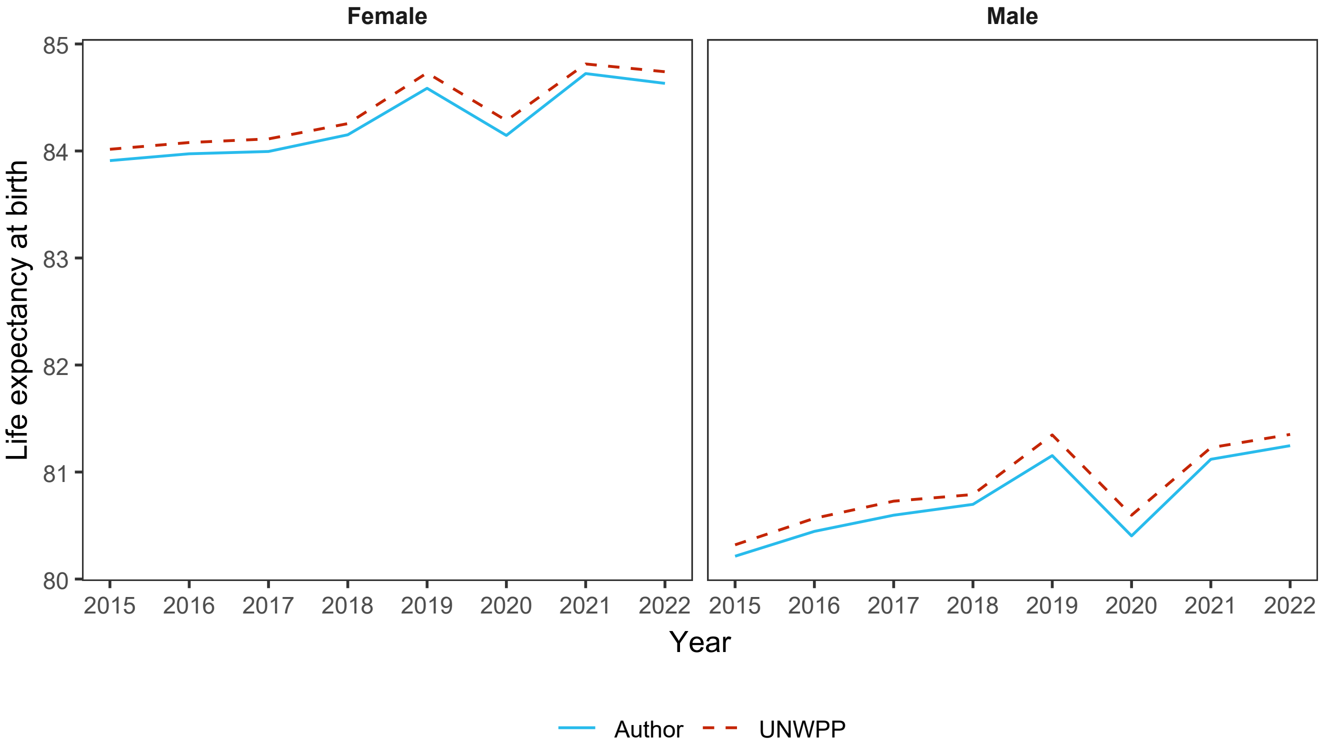

Figure S4t

Comparison of life expectancy at birth (Switzerland), author data set (solid line) vs. UNWPP (dashed line)

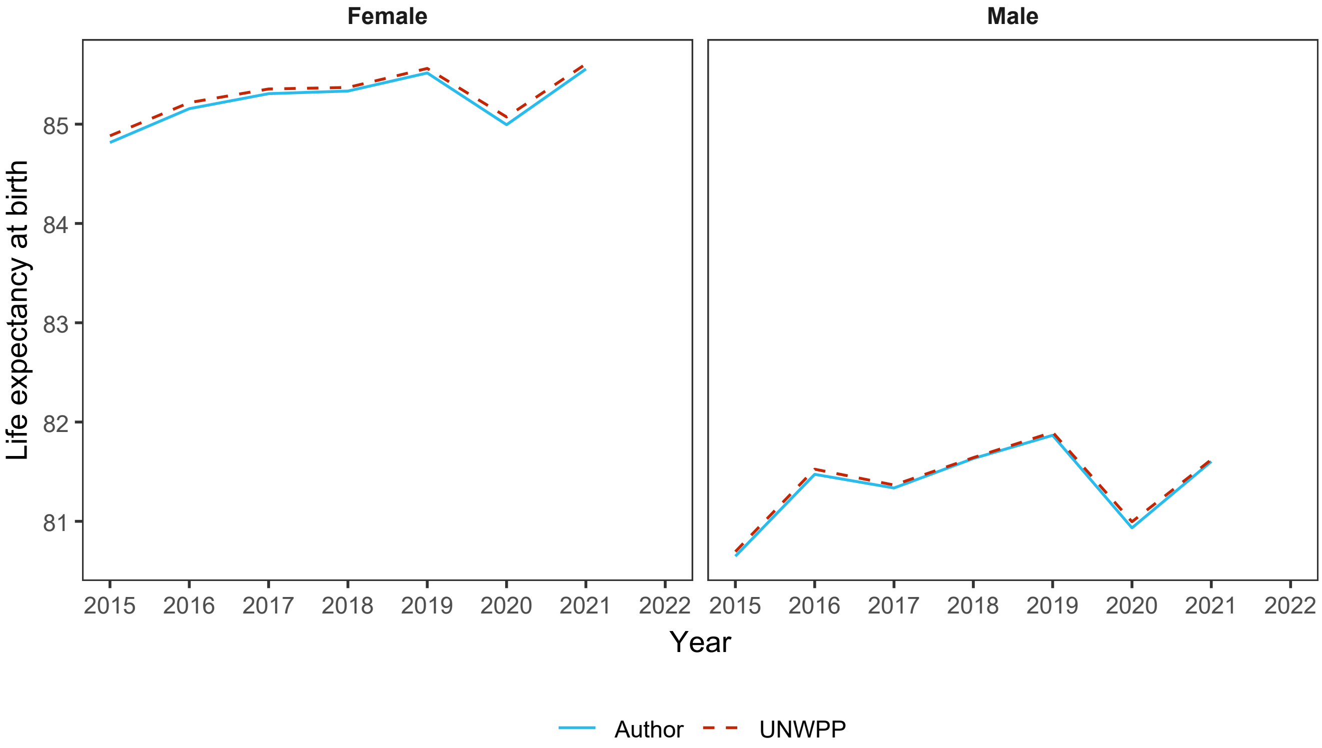

Figure S4u

Comparison of life expectancy at birth (USA),
author data set (solid line) vs. UNWPP (dashed line)

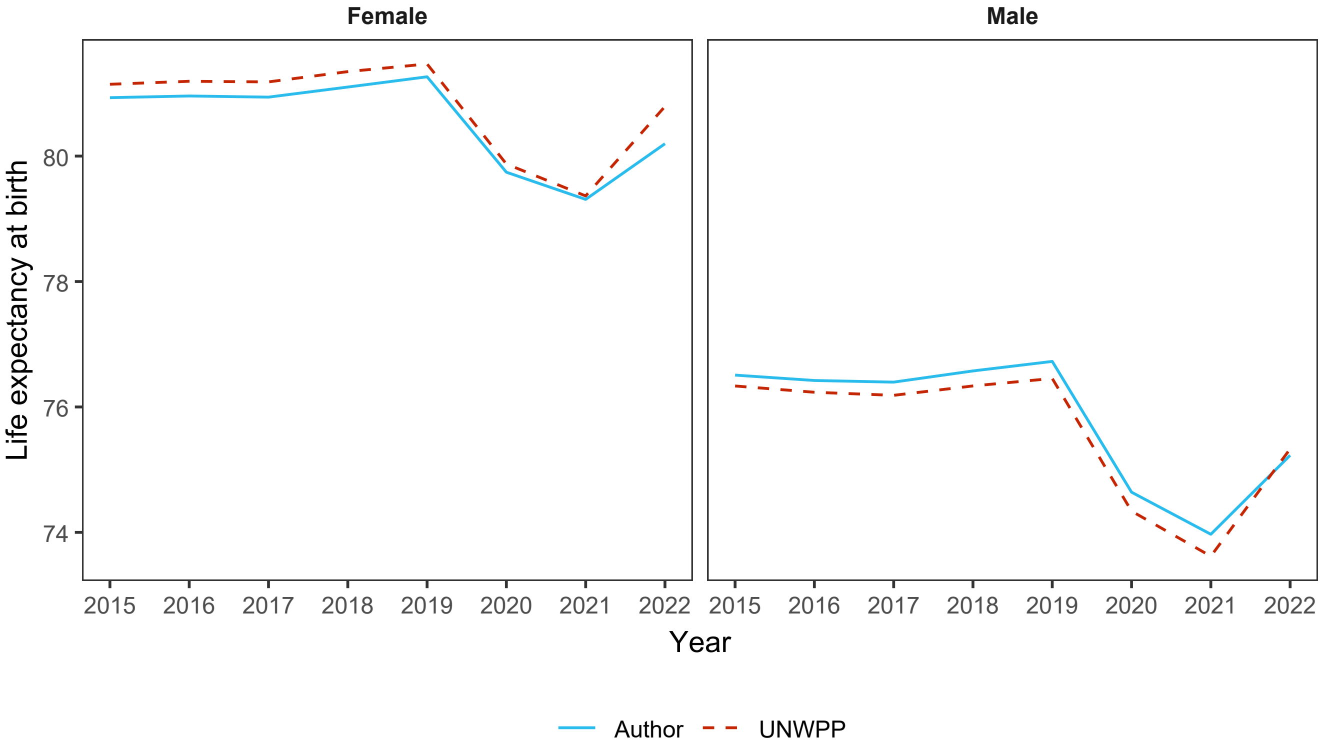

Figure S5

Contributions to changes in female life expectancy

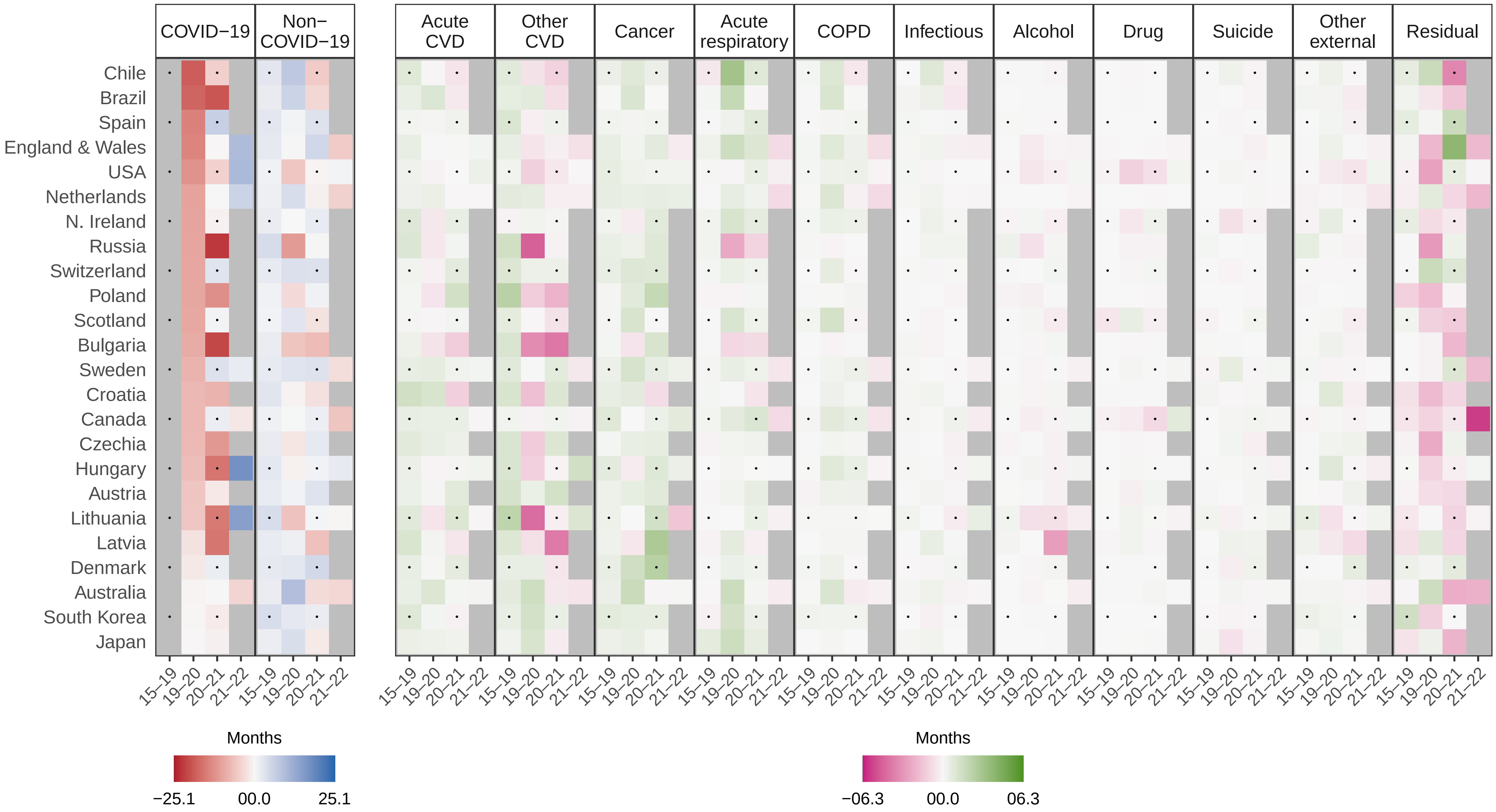

Figure S6

Contributions to changes in male life expectancy

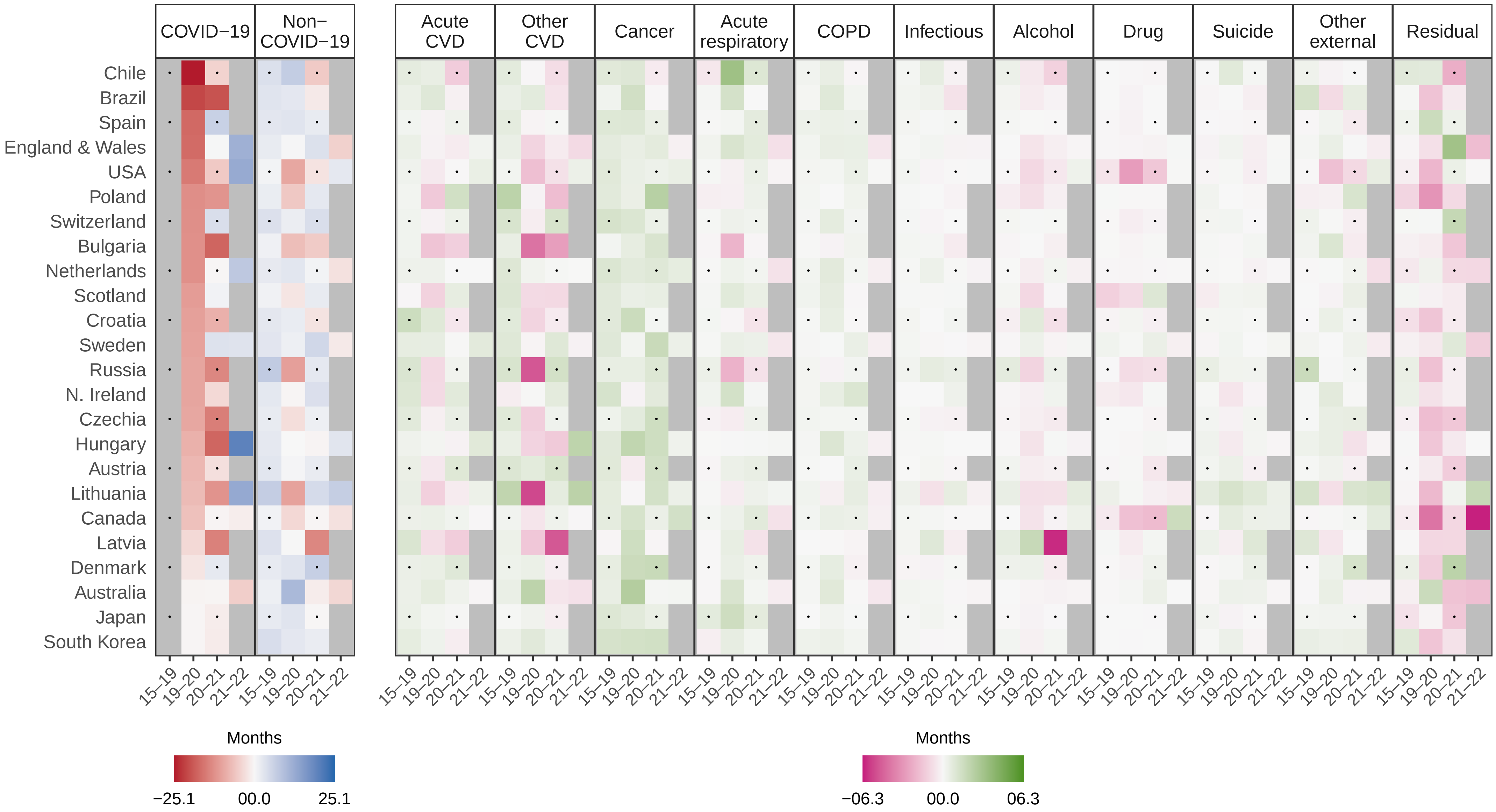

Figure S7a

Contributions to changes in female life expectancy
in Australia, Austria, Brazil

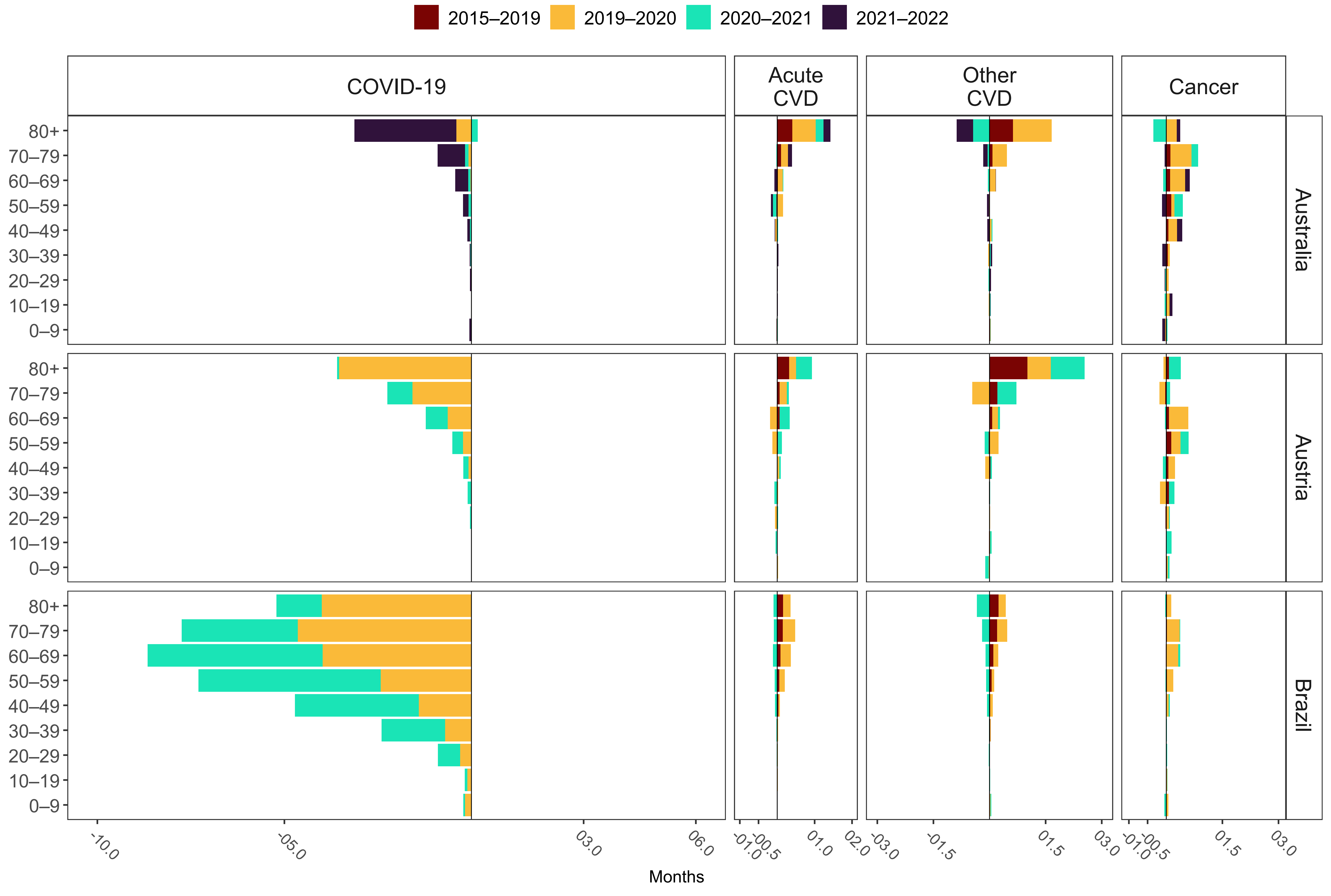

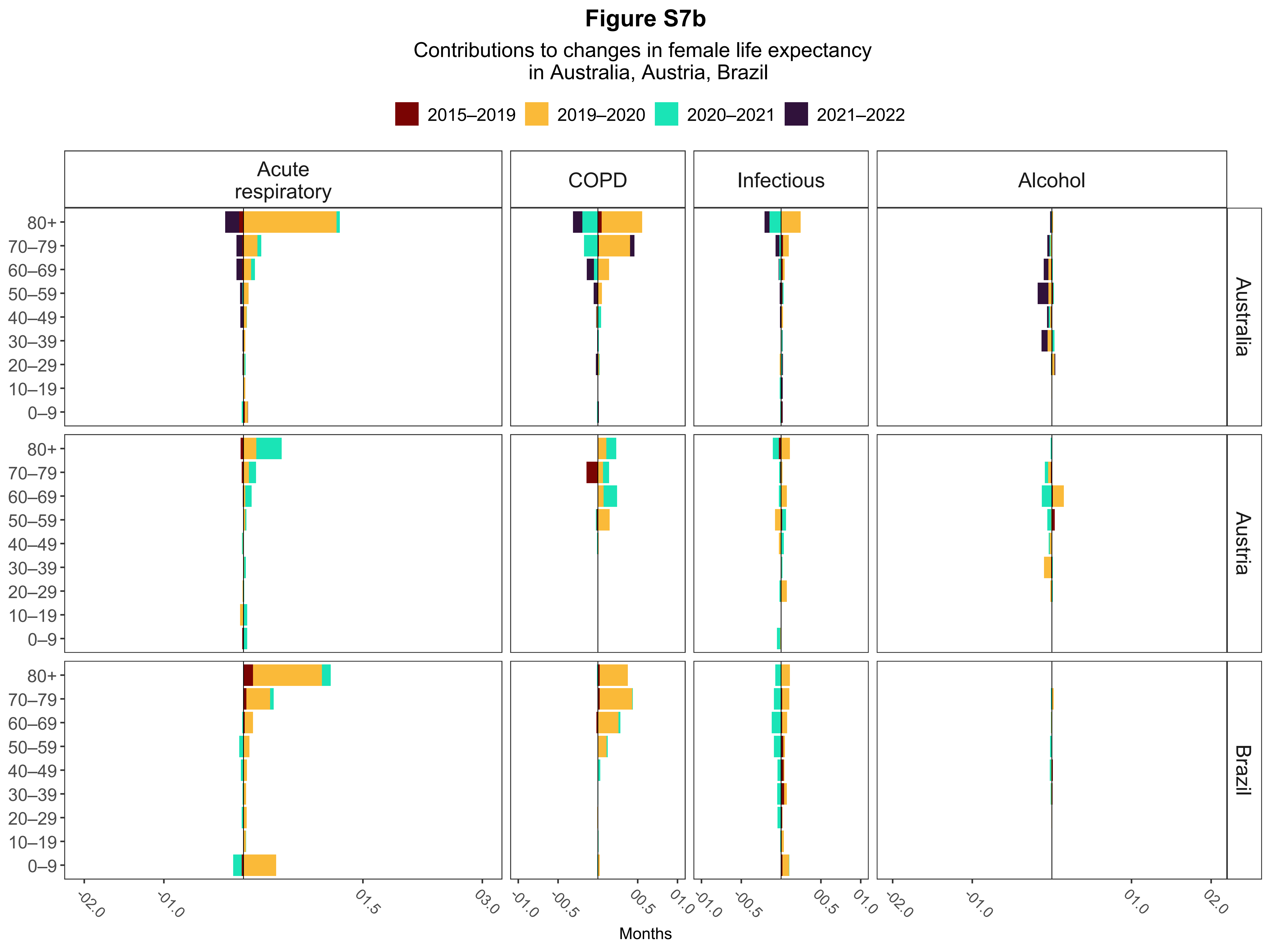

Figure S7b
Contributions to changes in female life expectancy in Australia, Austria, Brazil
2015–2019
2019–2020
2020–2021
2021–2022
Acute respiratory
COPD
Infectious
Alcohol
Australia
Austria
Brazil
80+
70–79
60–69
50–59
40–49
30–39
20–29
10–19
0–9
-02.0
-01.0
01.5
03.0
-01.0
-00.5
00.5
01.0
-01.0
-00.5
00.5
01.0
-02.0
-01.0
01.0
02.0
Months

Figure S7c

Contributions to changes in female life expectancy
in Australia, Austria, Brazil

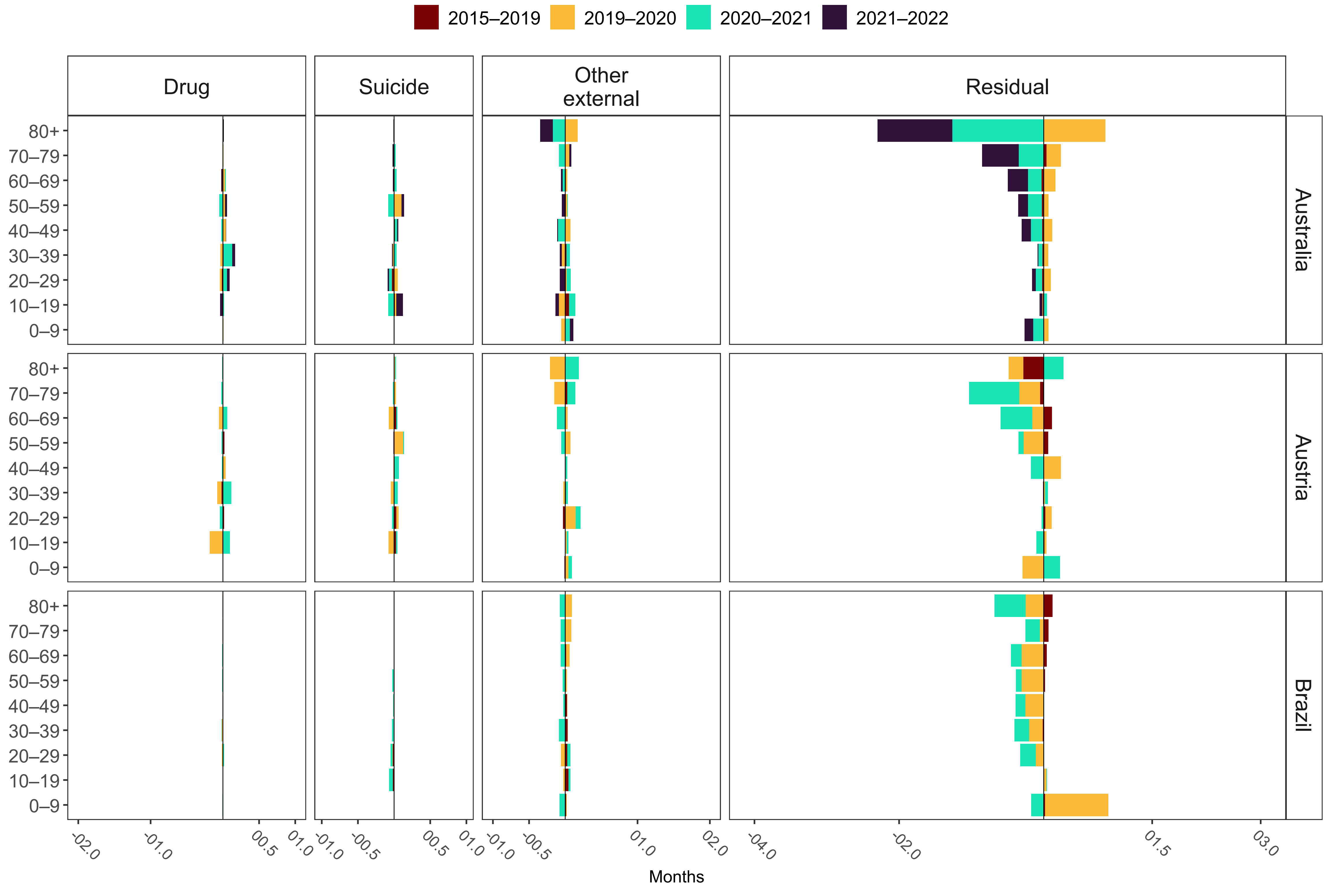

Figure S7d

Contributions to changes in female life expectancy in Bulgaria, Canada, Chile

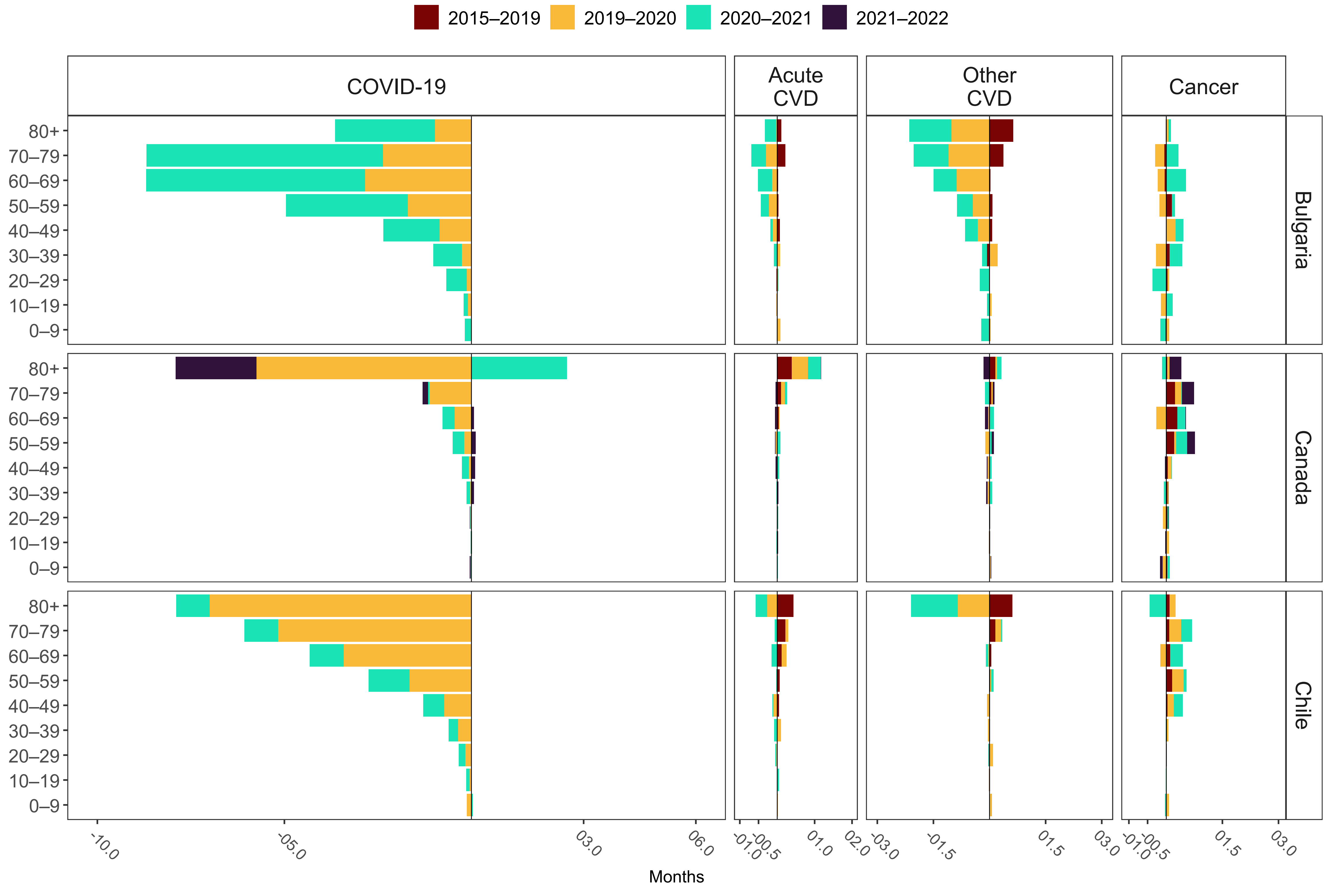

Figure S7e

Contributions to changes in female life expectancy
in Bulgaria, Canada, Chile

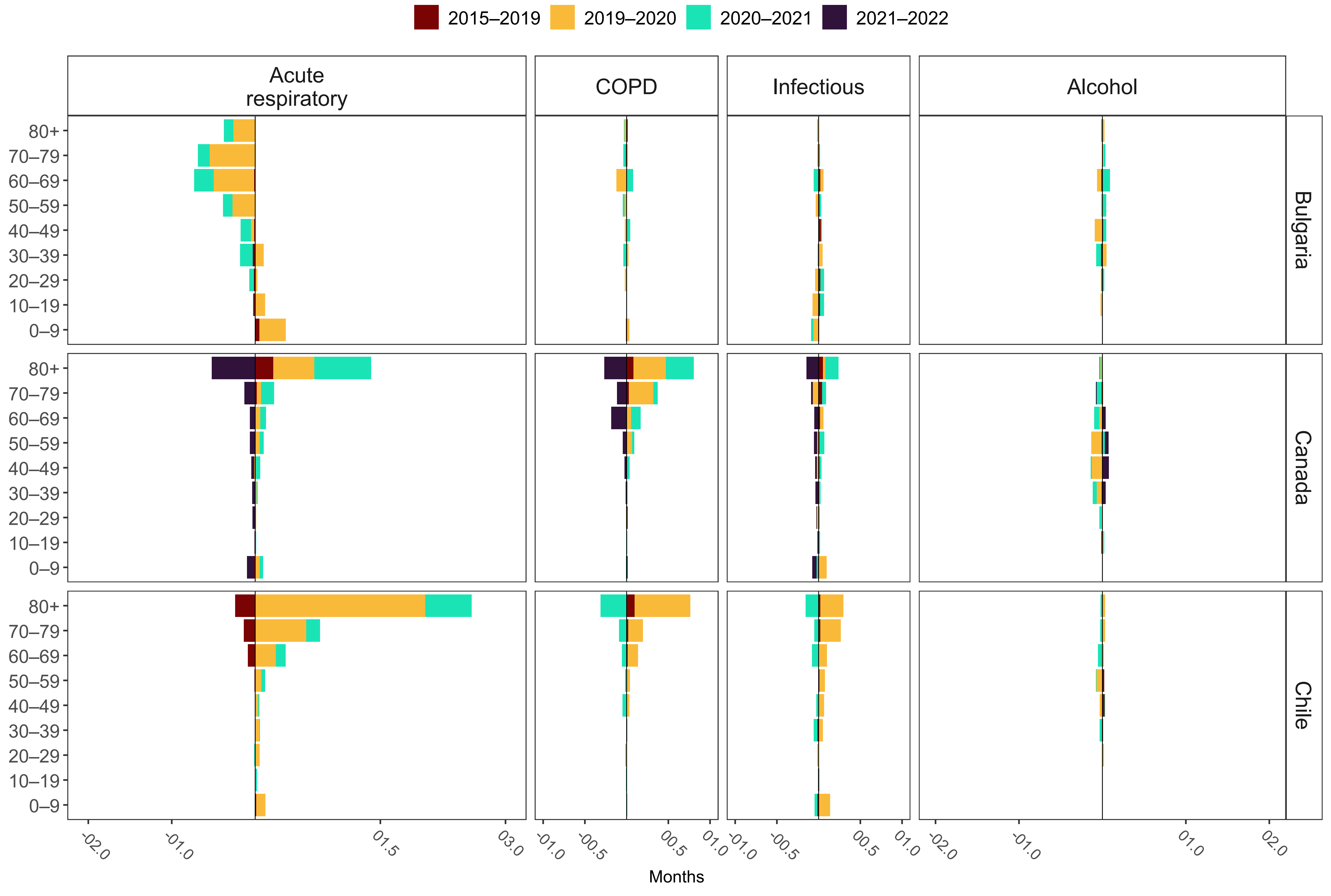

Figure S7f

Contributions to changes in female life expectancy
in Bulgaria, Canada, Chile

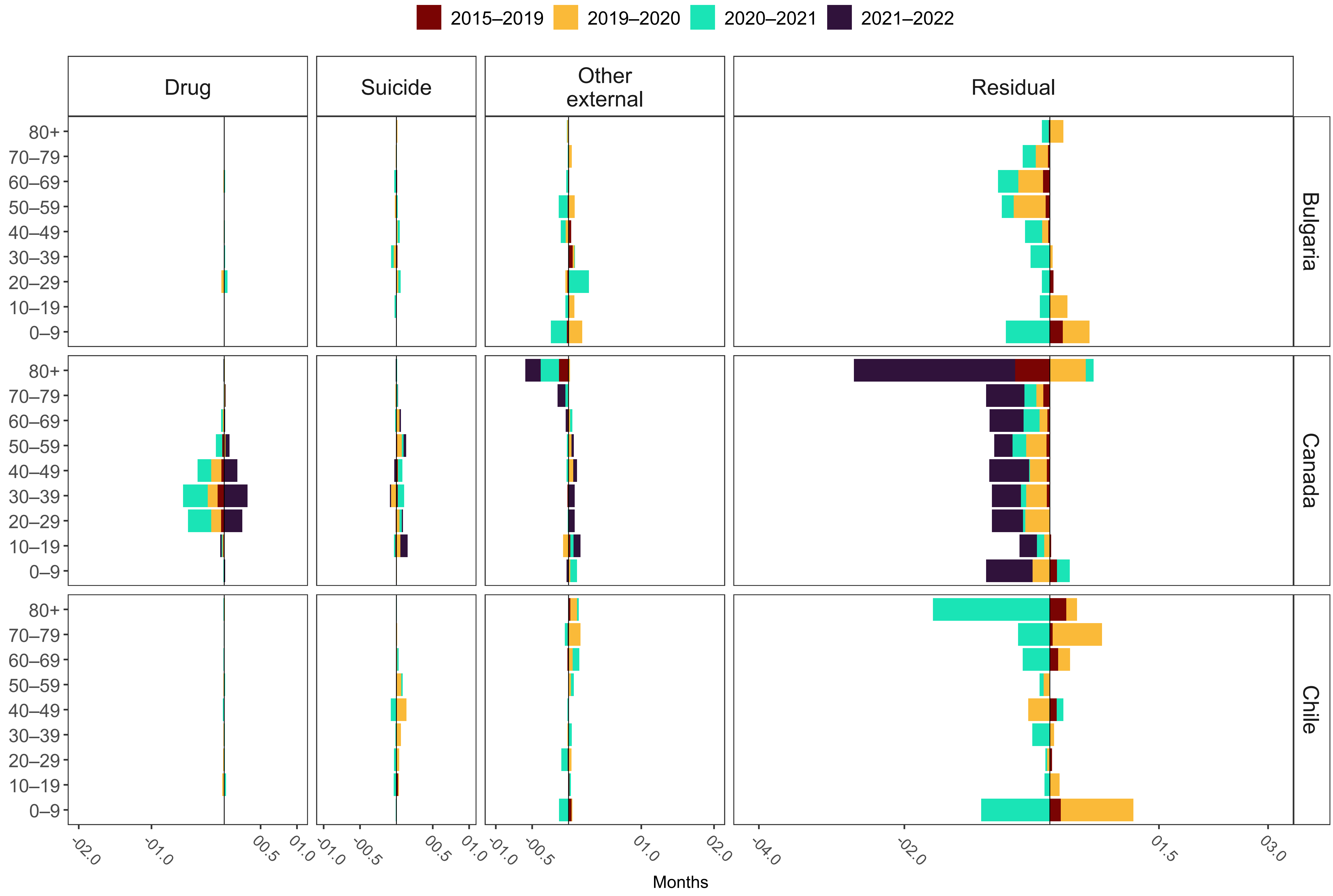

Figure S7g

Contributions to changes in female life expectancy
in Croatia, Czechia, Denmark

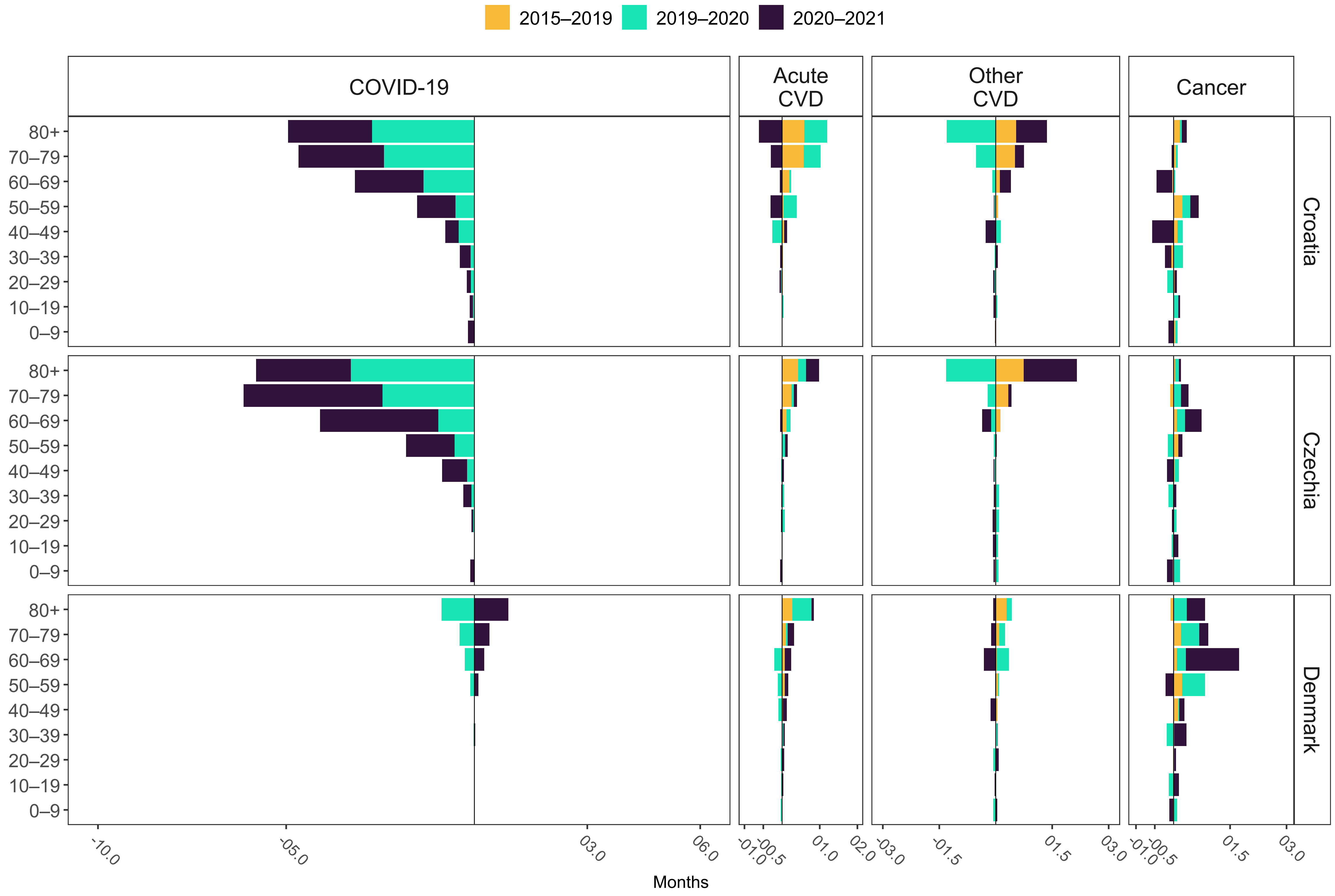

Figure S7h

Contributions to changes in female life expectancy
in Croatia, Czechia, Denmark

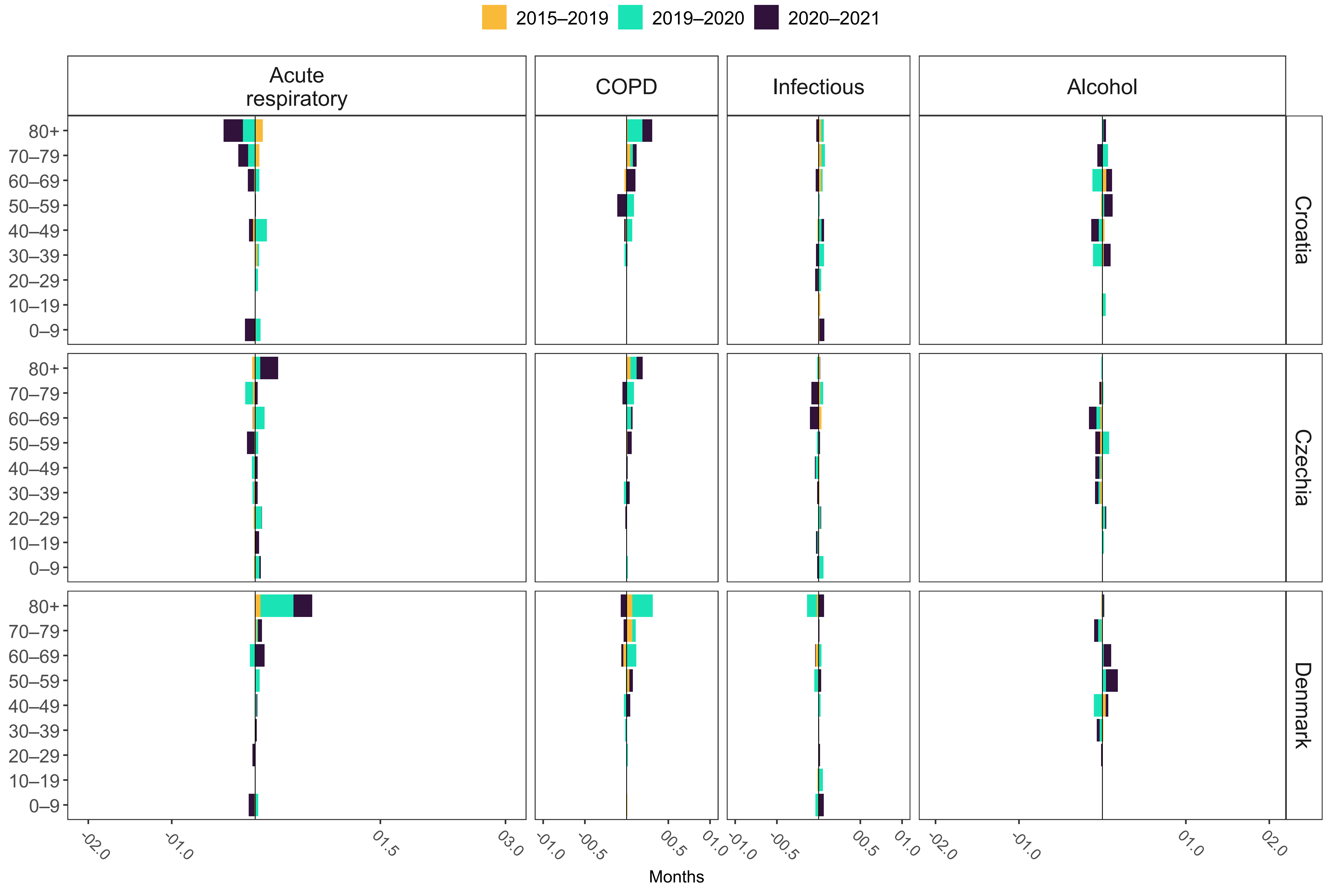

Figure S7i

Contributions to changes in female life expectancy in Croatia, Czechia, Denmark

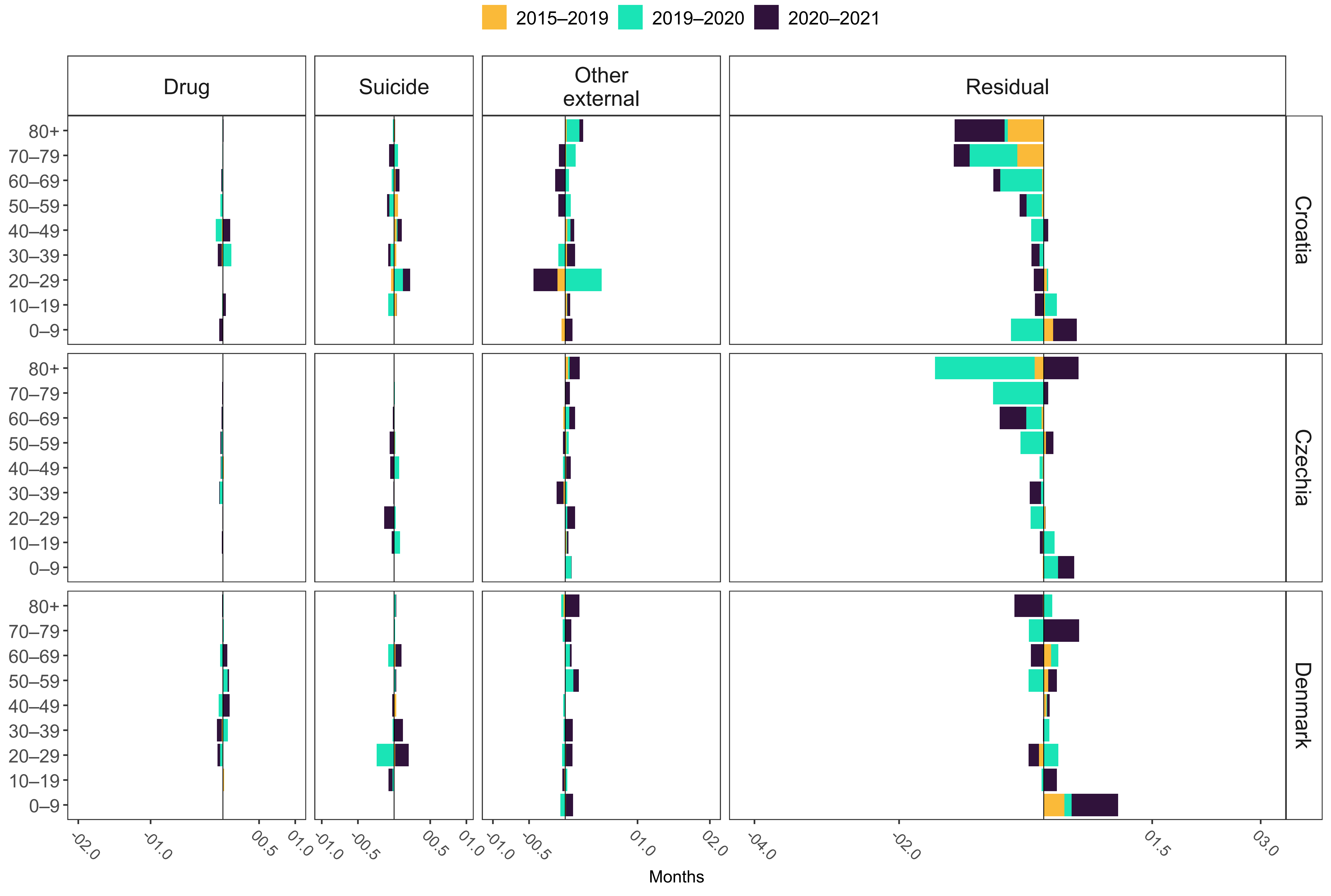

Figure S7j

Contributions to changes in female life expectancy
in England & Wales, Hungary, Japan

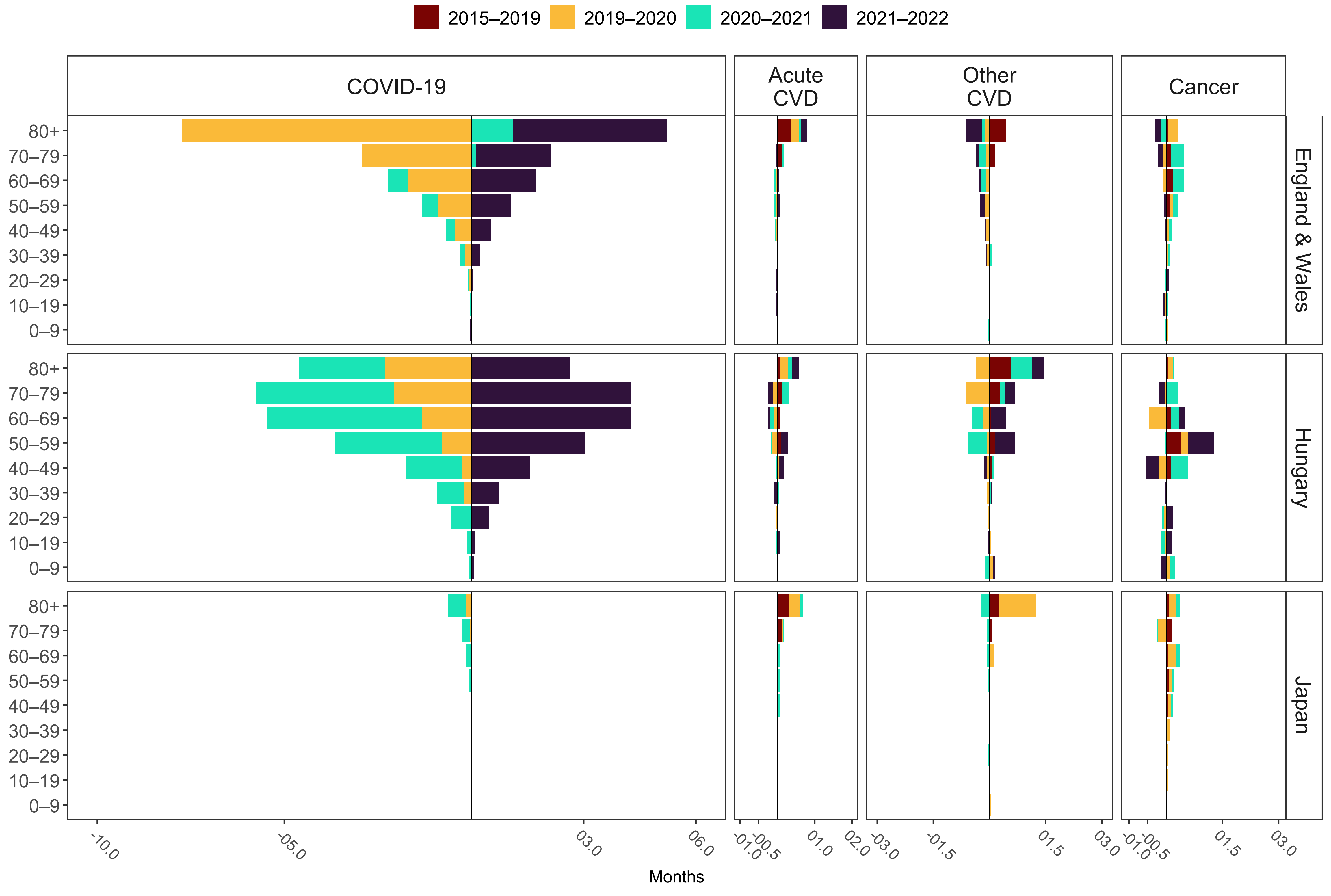

Figure S7k

Contributions to changes in female life expectancy
in England & Wales, Hungary, Japan

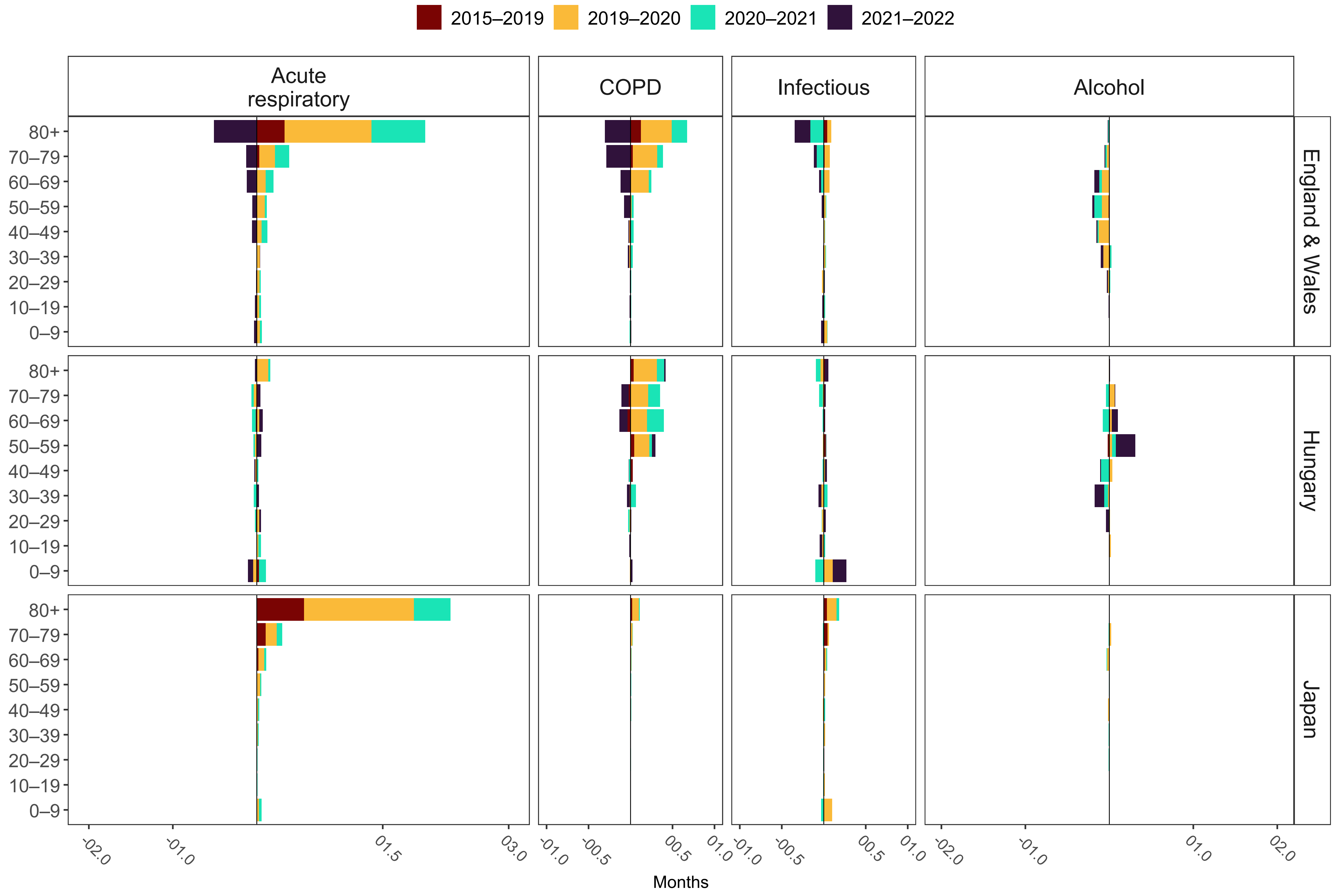

Figure S7I

Contributions to changes in female life expectancy
in England & Wales, Hungary, Japan

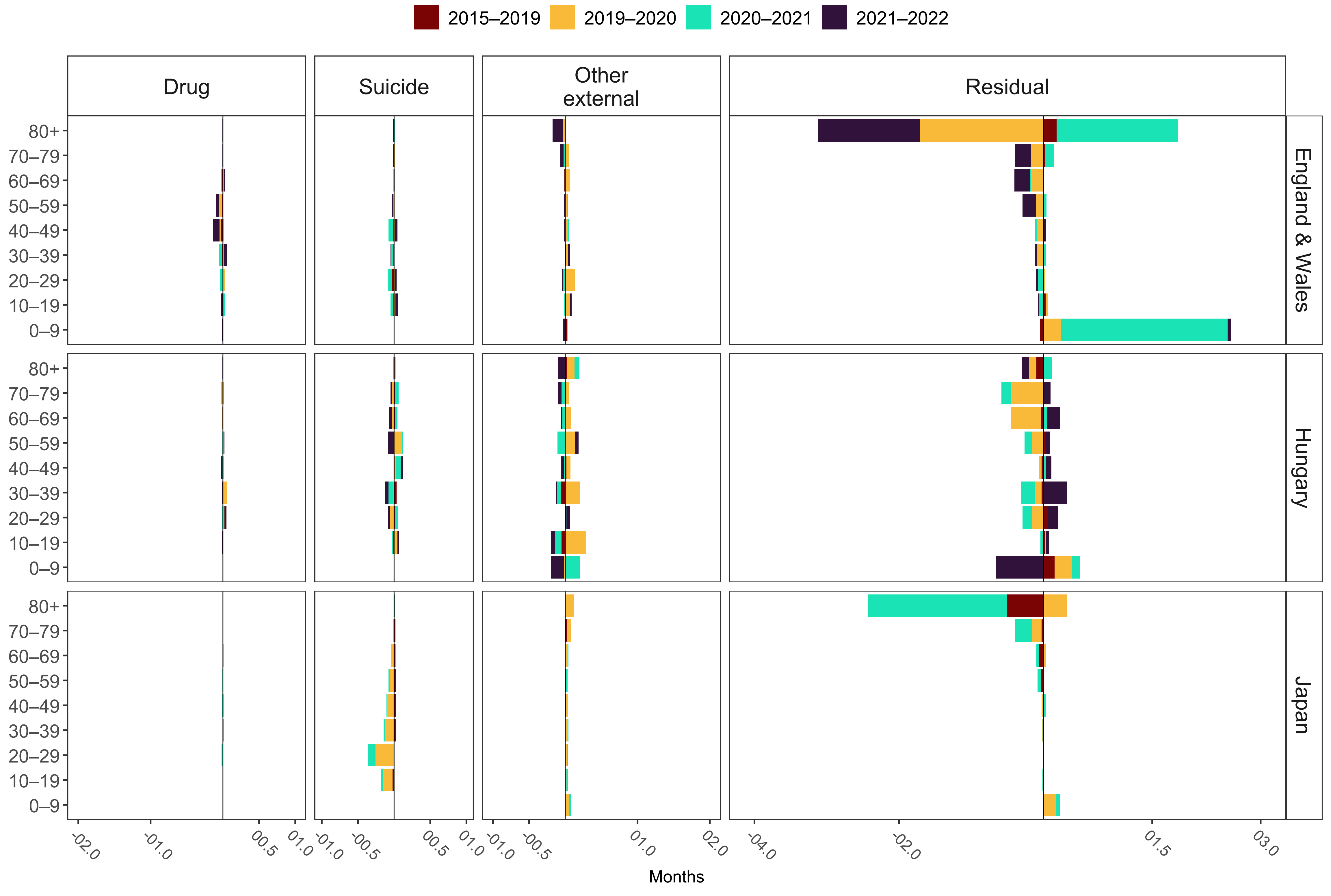

Figure S7m

Contributions to changes in female life expectancy
in Latvia, Lithuania, Netherlands

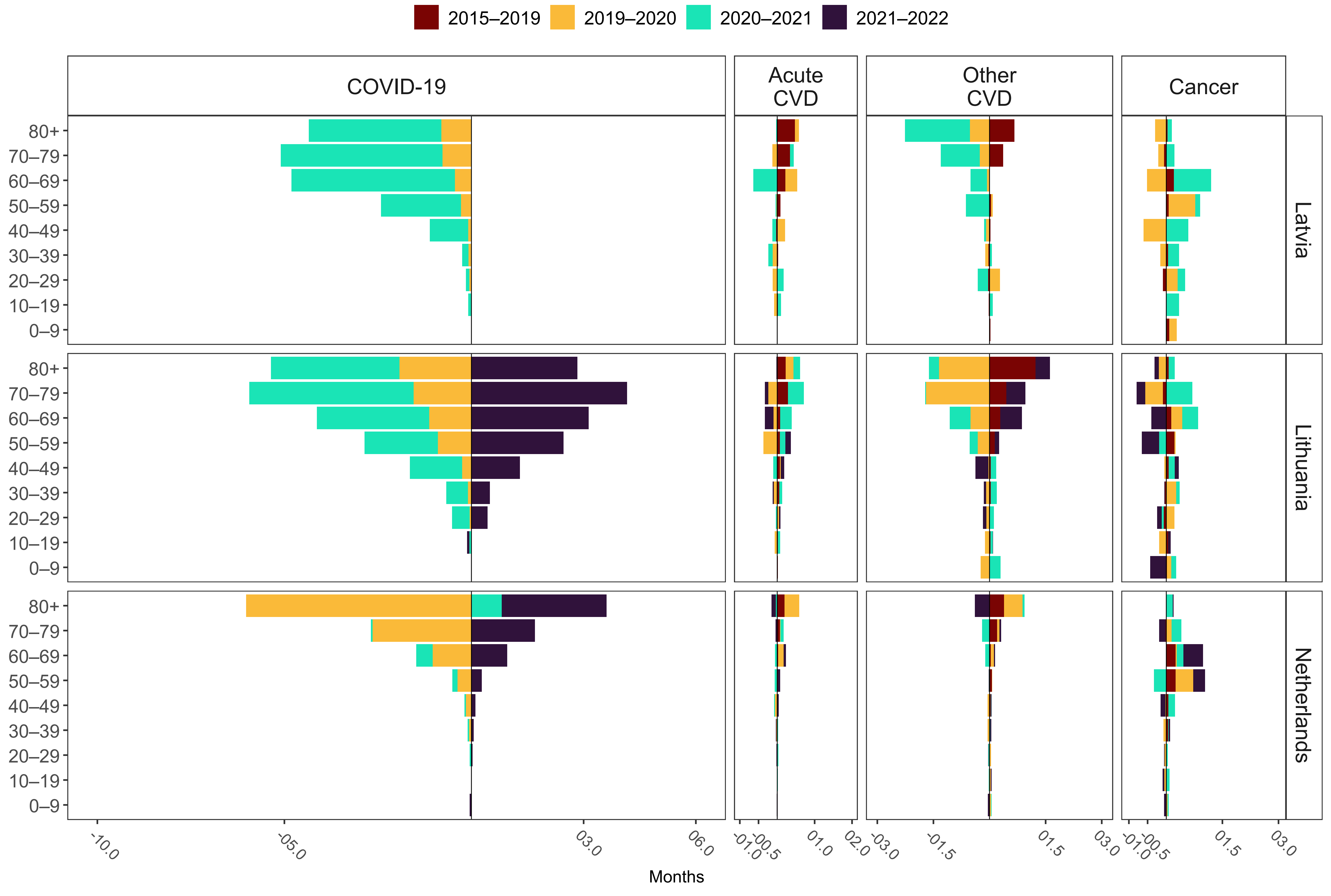

Figure S7n

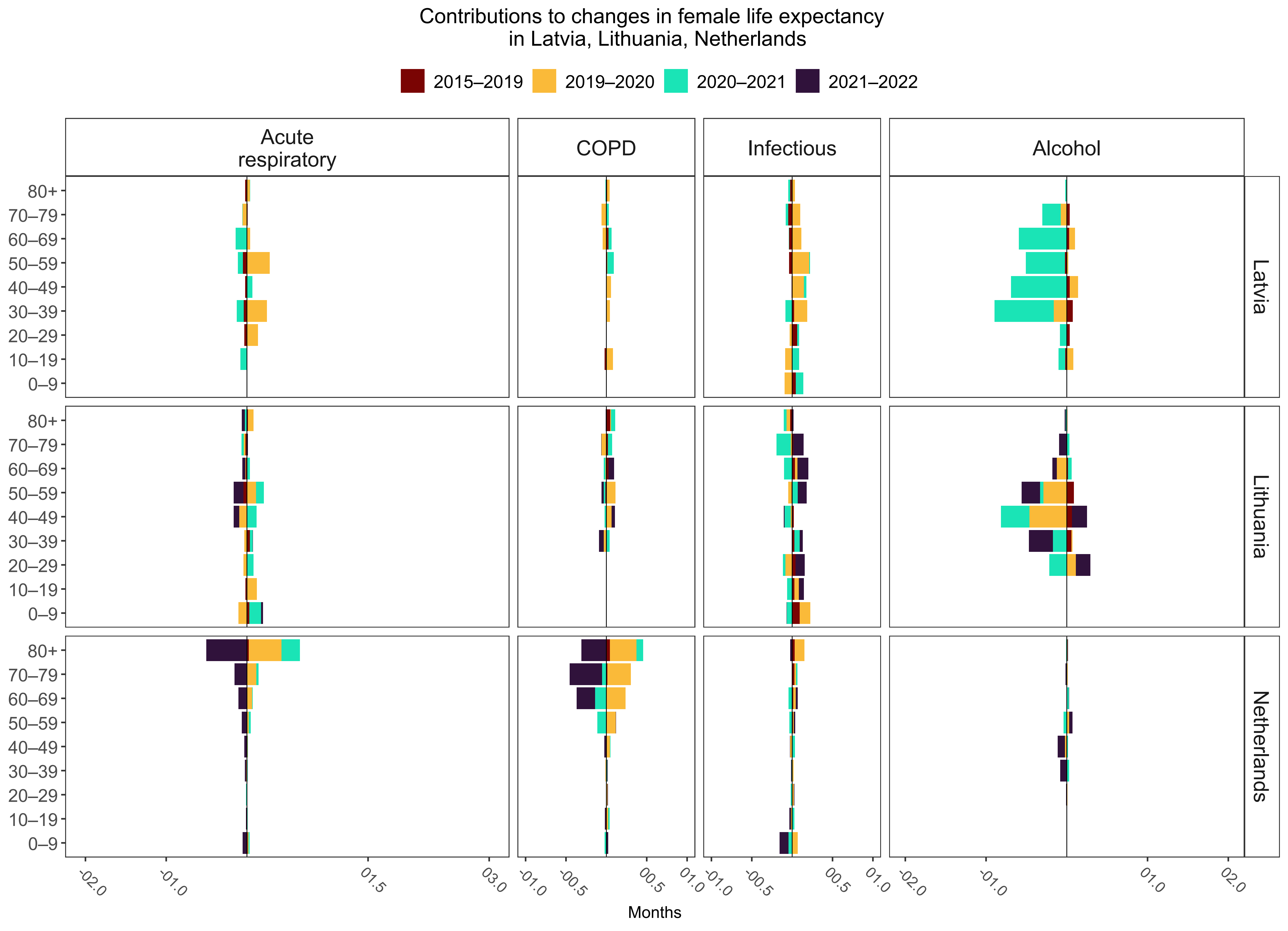

Contributions to changes in female life expectancy
in Latvia, Lithuania, Netherlands
2015–2019
2019–2020
2020–2021
2021–2022
Acute respiratory
COPD
Infectious
Alcohol
Latvia
Lithuania
Netherlands
80+
70–79
60–69
50–59
40–49
30–39
20–29
10–19
0–9
-02.0
-01.0
01.5
03.0
-01.0
-00.5
00.5
01.0
-01.0
-00.5
00.5
01.0
-02.0
-01.0
01.0
02.0
Months

Figure S7o

Contributions to changes in female life expectancy
in Latvia, Lithuania, Netherlands

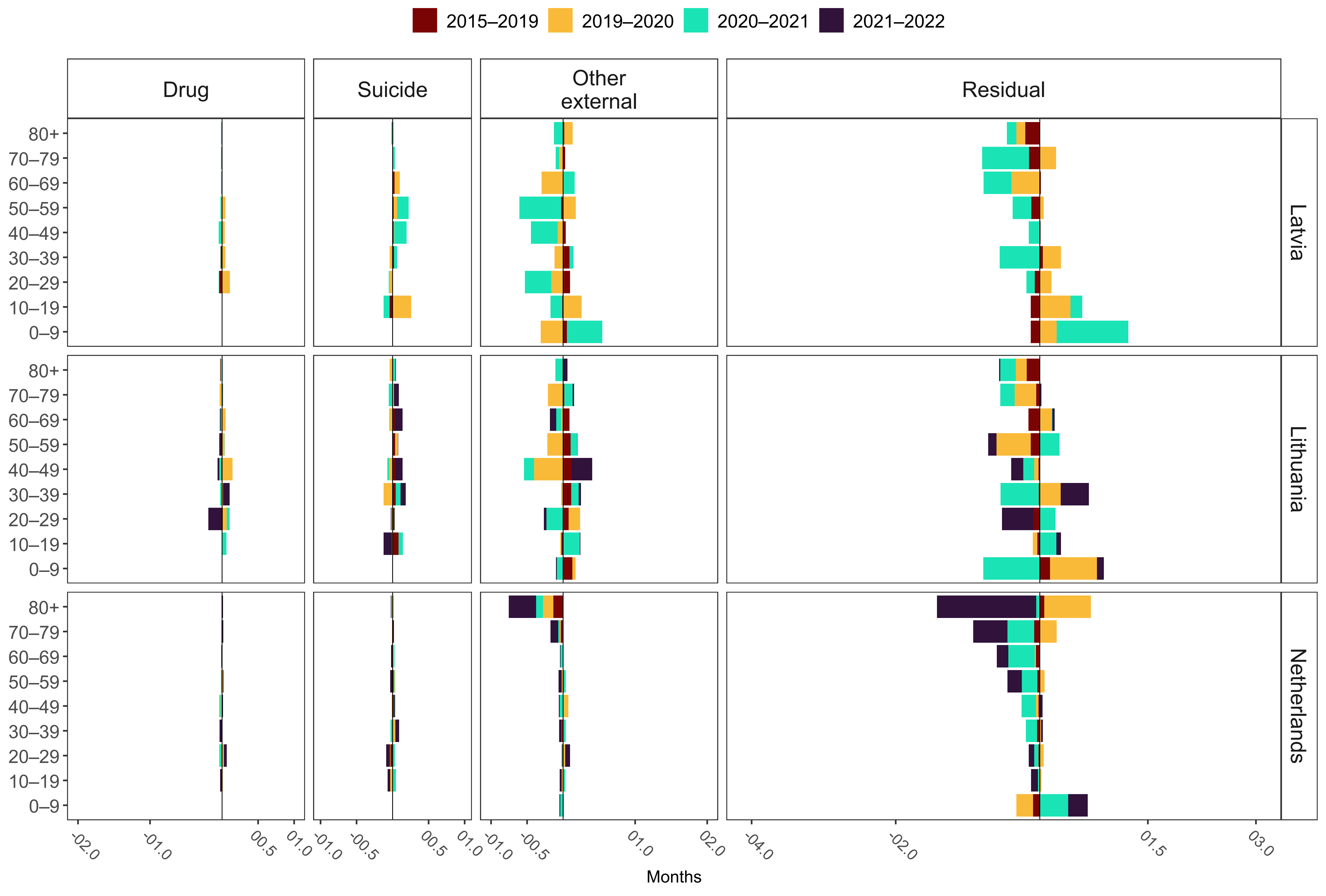

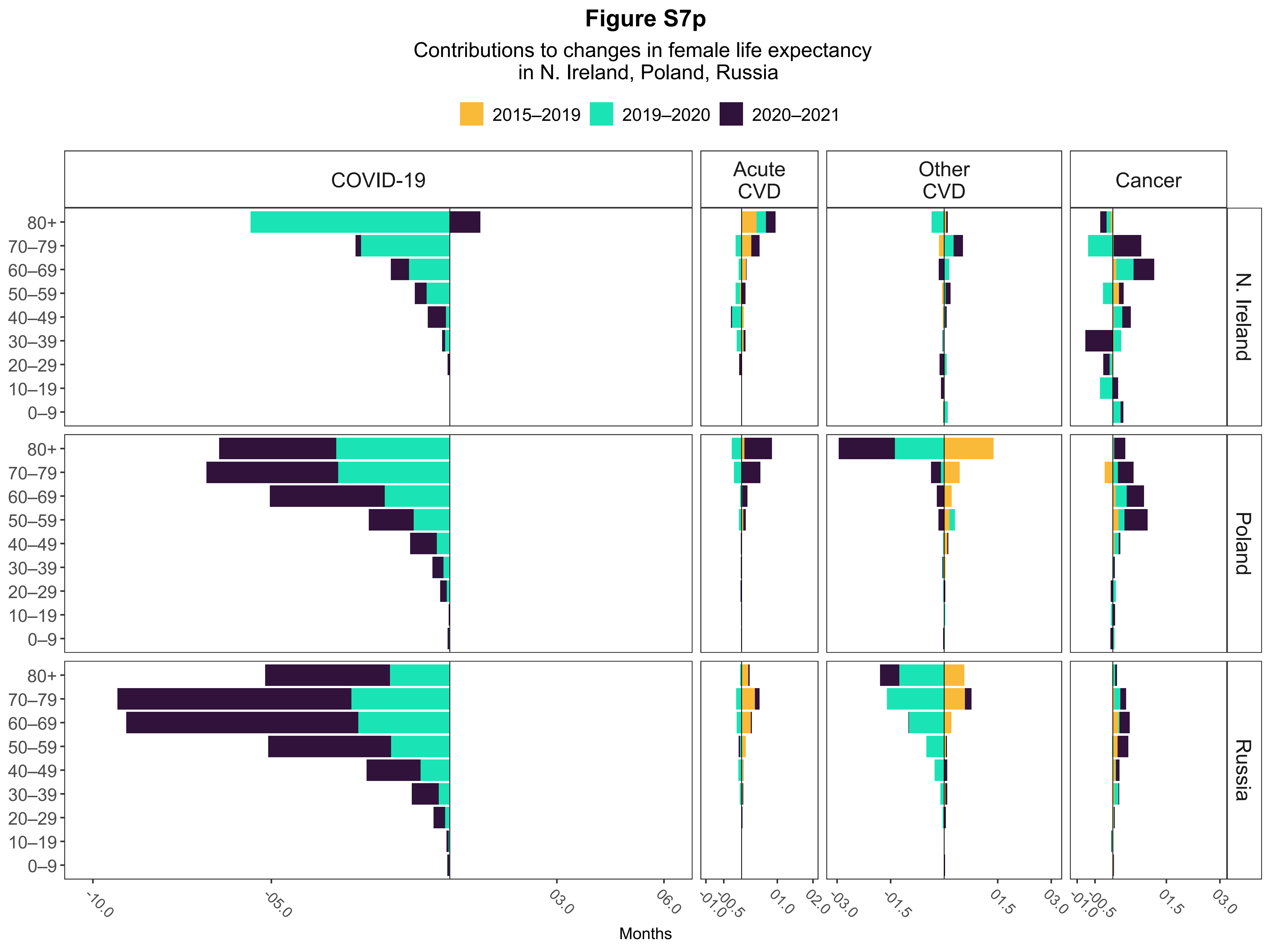
Figure S7p
Contributions to changes in female life expectancy
in N. Ireland, Poland, Russia
2015–2019
2019–2020
2020–2021
COVID-19
Acute CVD
Other CVD
Cancer
N. Ireland
Poland
Russia
80+
70–79
60–69
50–59
40–49
30–39
20–29
10–19
0–9
-10.0
-05.0
03.0
06.0
-01.0
-00.5
01.0
02.0
-03.0
-01.5
01.5
03.0
Months

Figure S7q

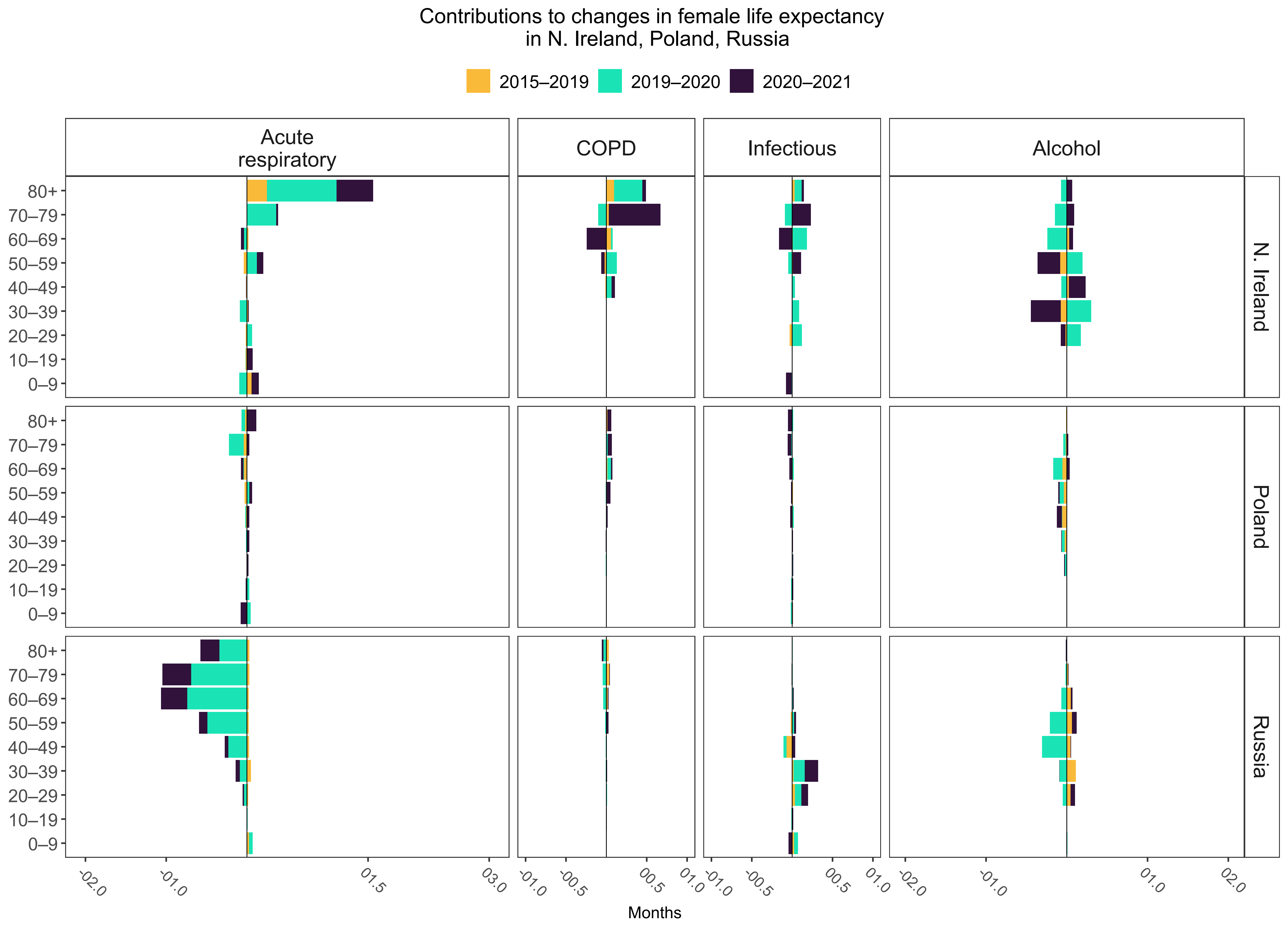

Contributions to changes in female life expectancy
in N. Ireland, Poland, Russia
2015–2019
2019–2020
2020–2021
Acute respiratory
COPD
Infectious
Alcohol
N. Ireland
Poland
Russia
80+
70–79
60–69
50–59
40–49
30–39
20–29
10–19
0–9
-02.0
-01.0
01.5
03.0
-01.0
-00.5
00.5
01.0
-01.0
-00.5
00.5
01.0
-02.0
-01.0
01.0
02.0
Months

Figure S7r

Contributions to changes in female life expectancy
in N. Ireland, Poland, Russia

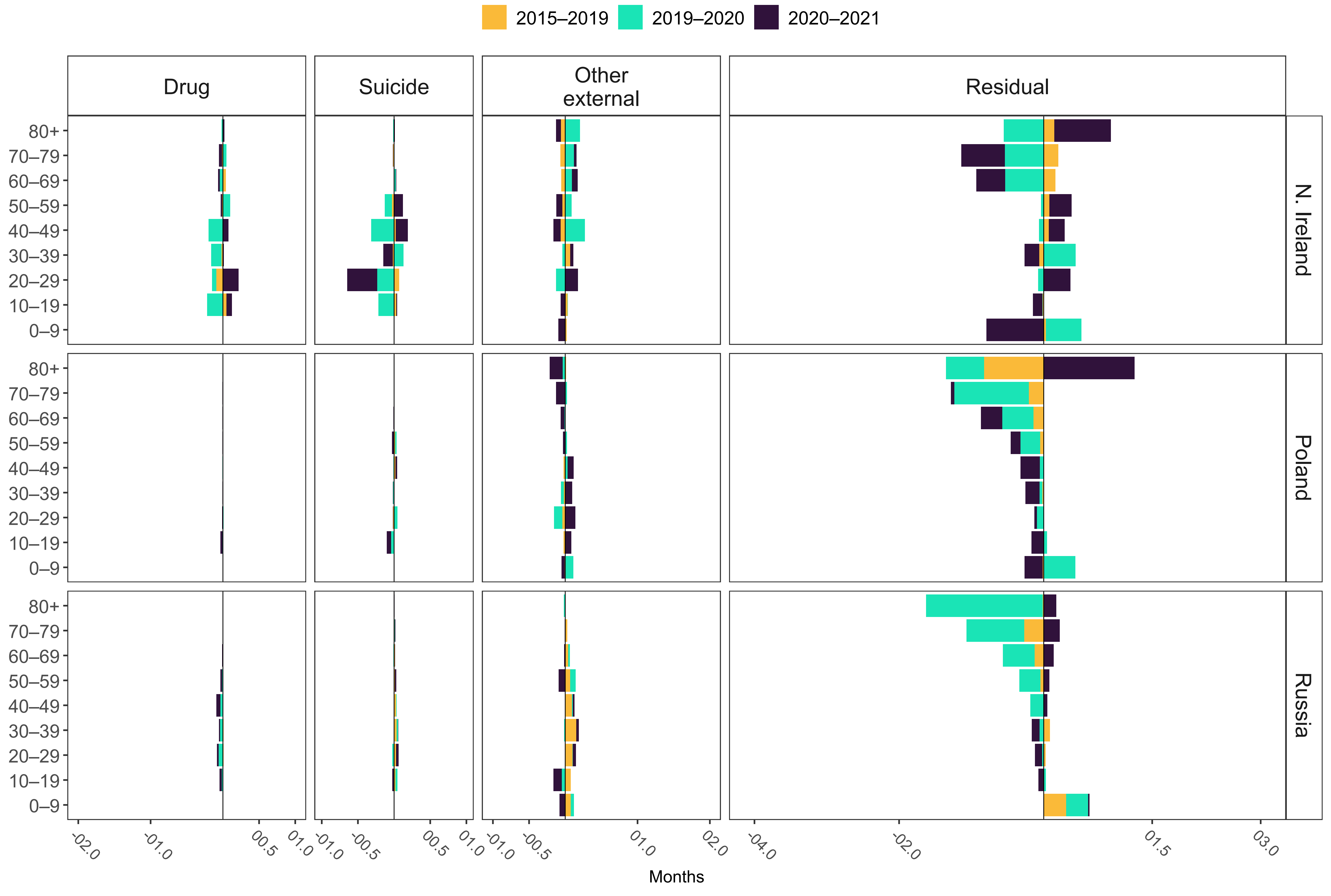

Figure S7s

Contributions to changes in female life expectancy
in Scotland, South Korea, Spain

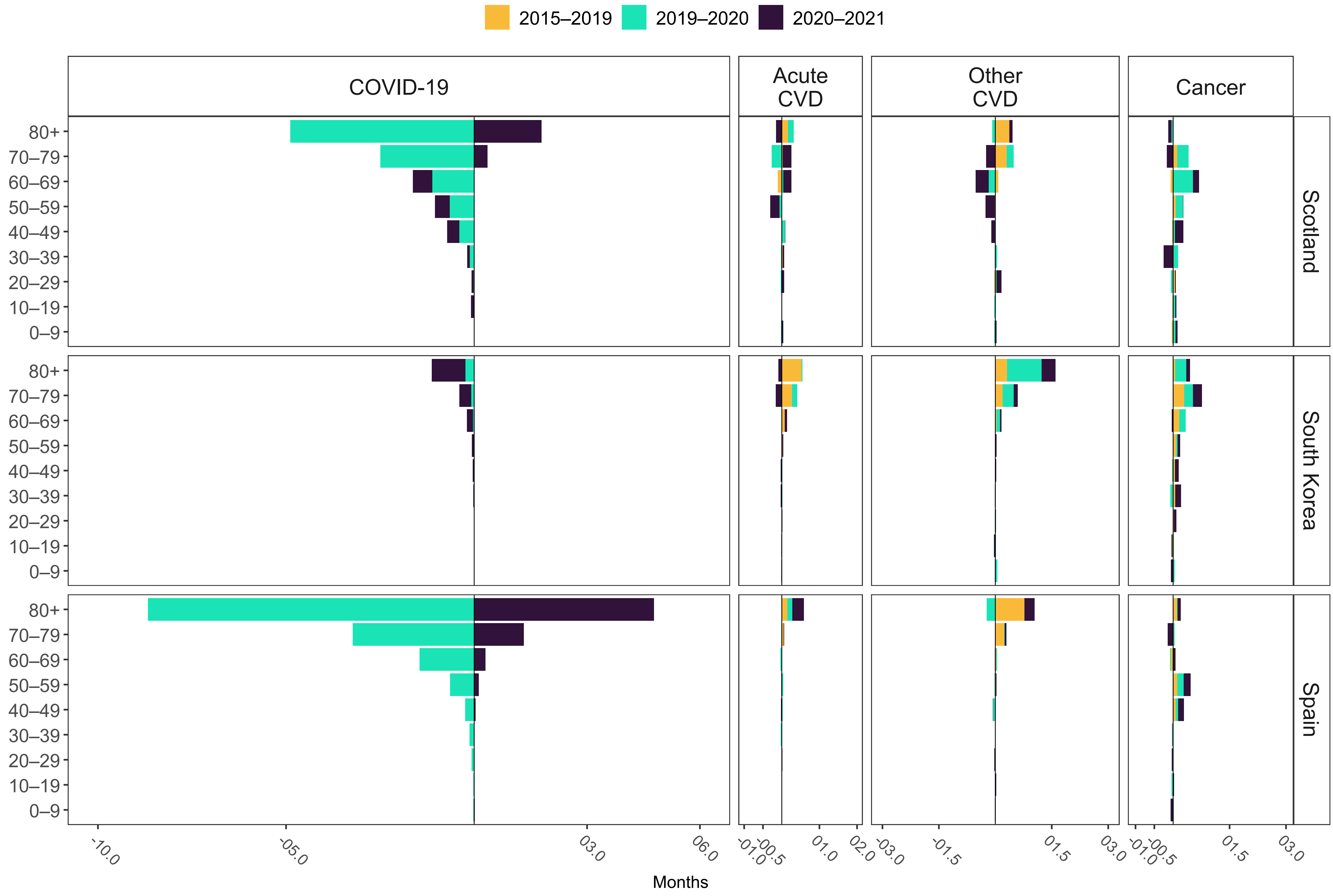

Figure S7t

Contributions to changes in female life expectancy
in Scotland, South Korea, Spain

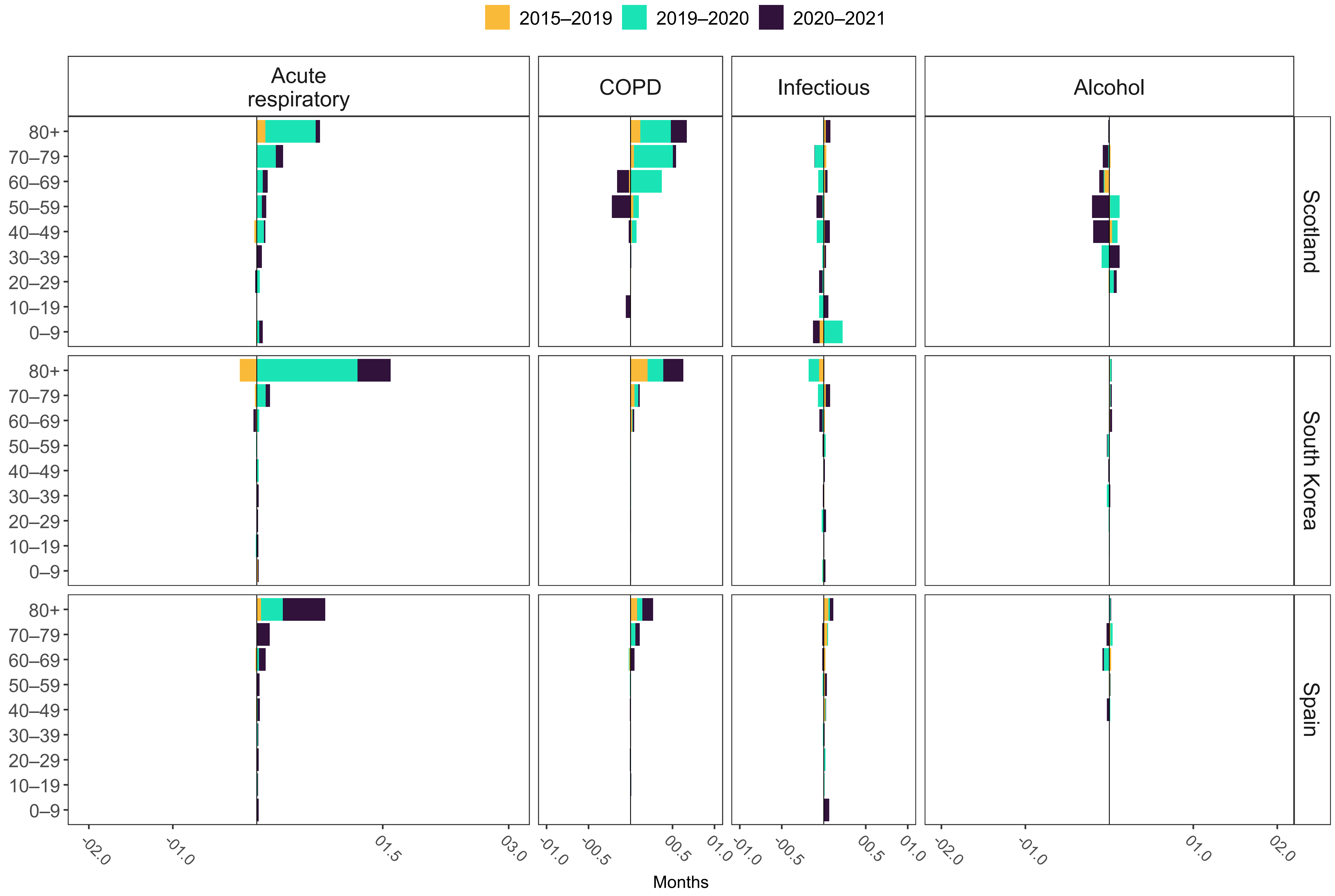

Figure S7u

Contributions to changes in female life expectancy
in Scotland, South Korea, Spain

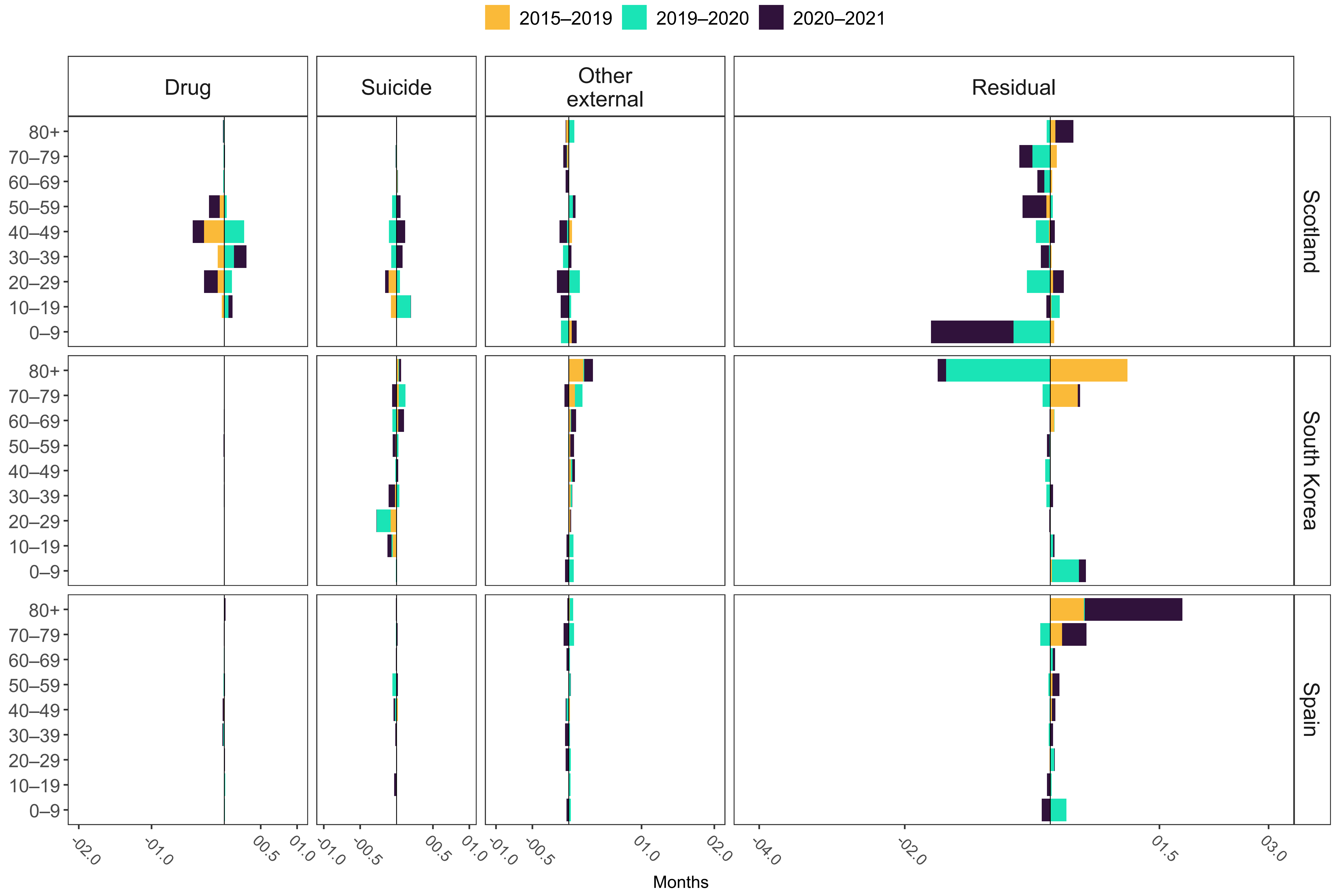

Figure S7v

Contributions to changes in female life expectancy
in Sweden, Switzerland, USA

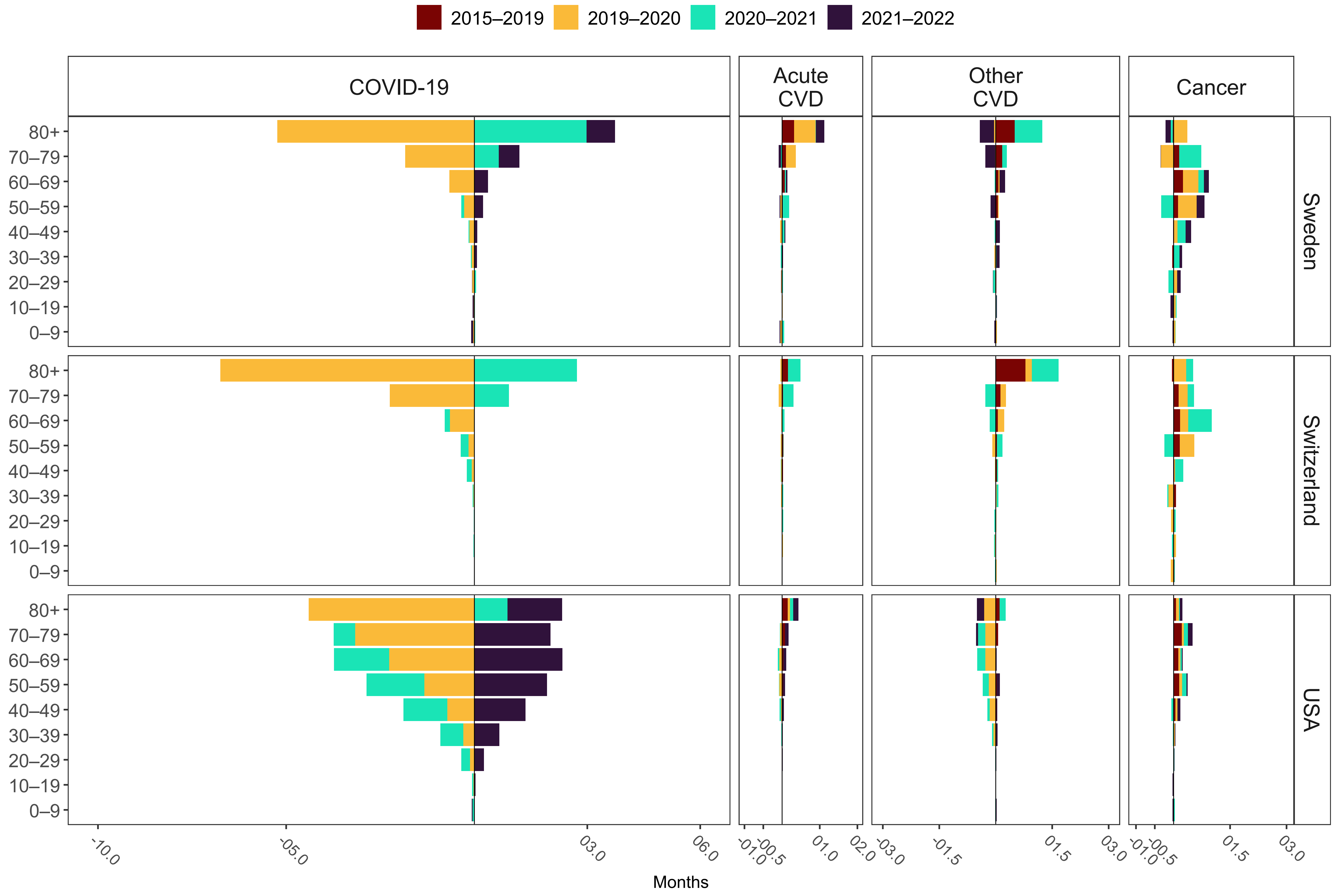

Figure S7w

Contributions to changes in female life expectancy
in Sweden, Switzerland, USA

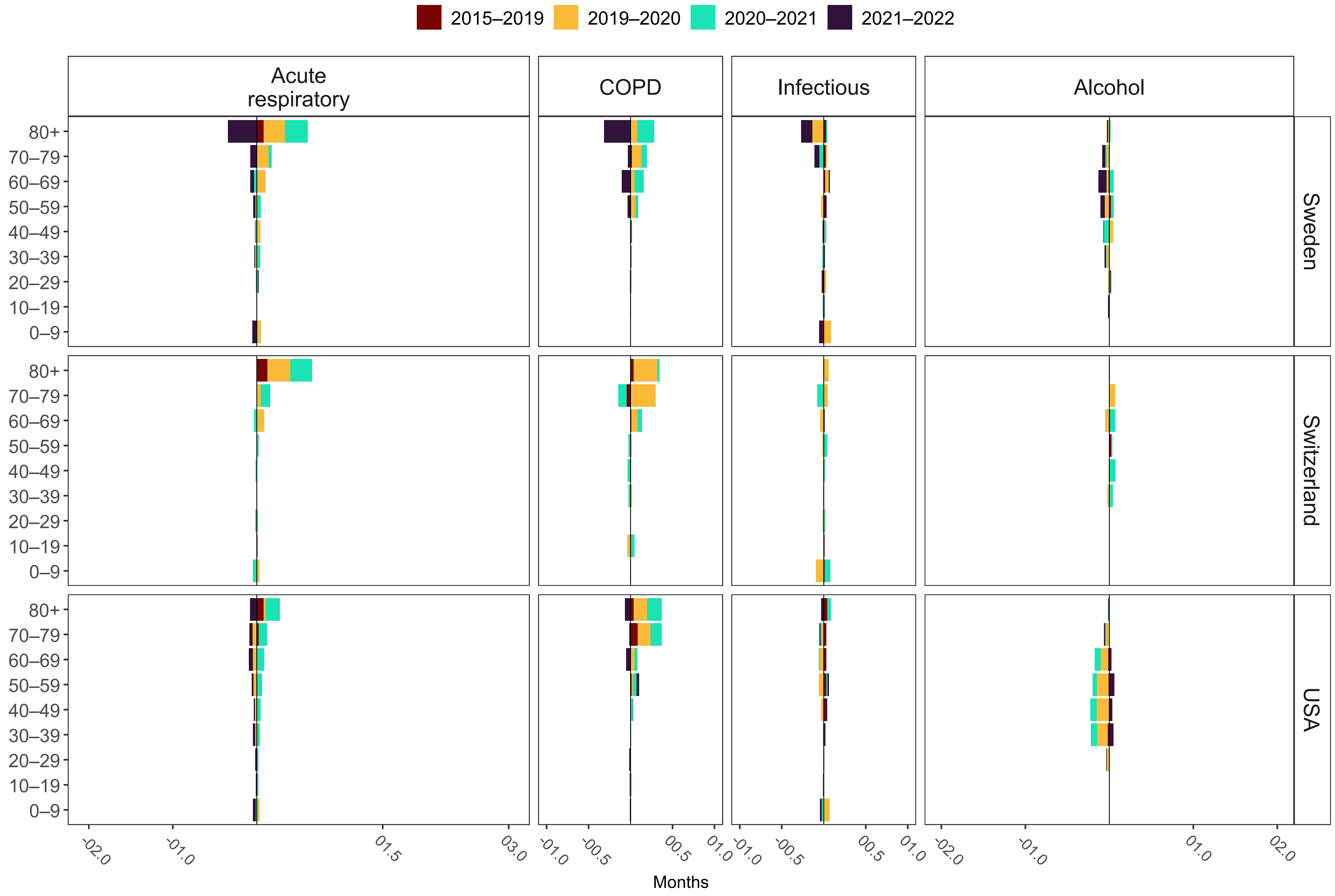

Figure S7x

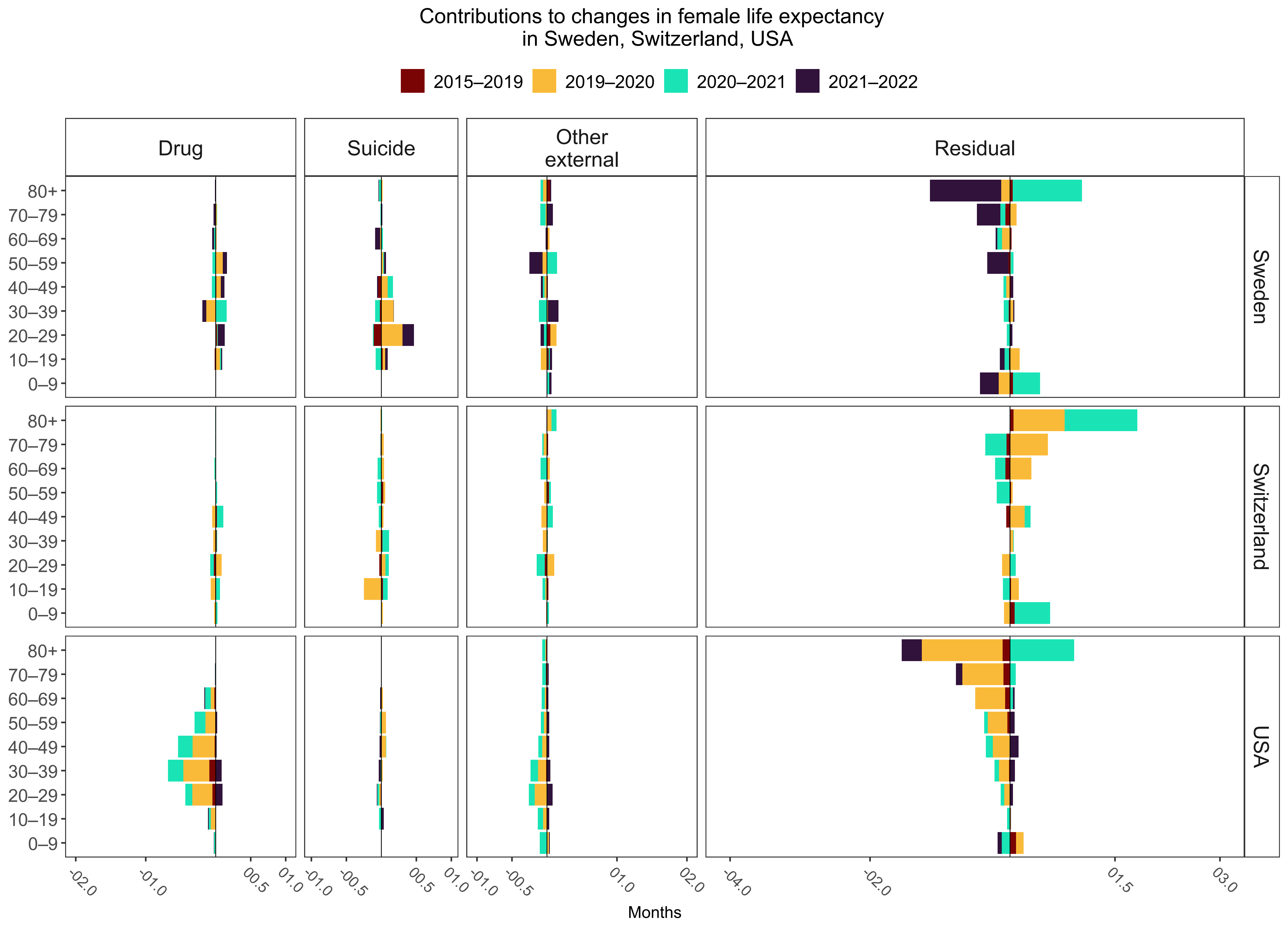
Contributions to changes in female life expectancy
in Sweden, Switzerland, USA
2015–2019
2019–2020
2020–2021
2021–2022
Drug
Suicide
Other external
Residual
Sweden
Switzerland
USA
80+
70–79
60–69
50–59
40–49
30–39
20–29
10–19
0–9
-02.0
-01.0
00.5
01.0
-01.0
-00.5
00.5
01.0
-01.0
-00.5
01.0
02.0
-04.0
-02.0
01.5
03.0
Months

Figure S8a

Contributions to changes in male life expectancy
in Australia, Austria, Brazil

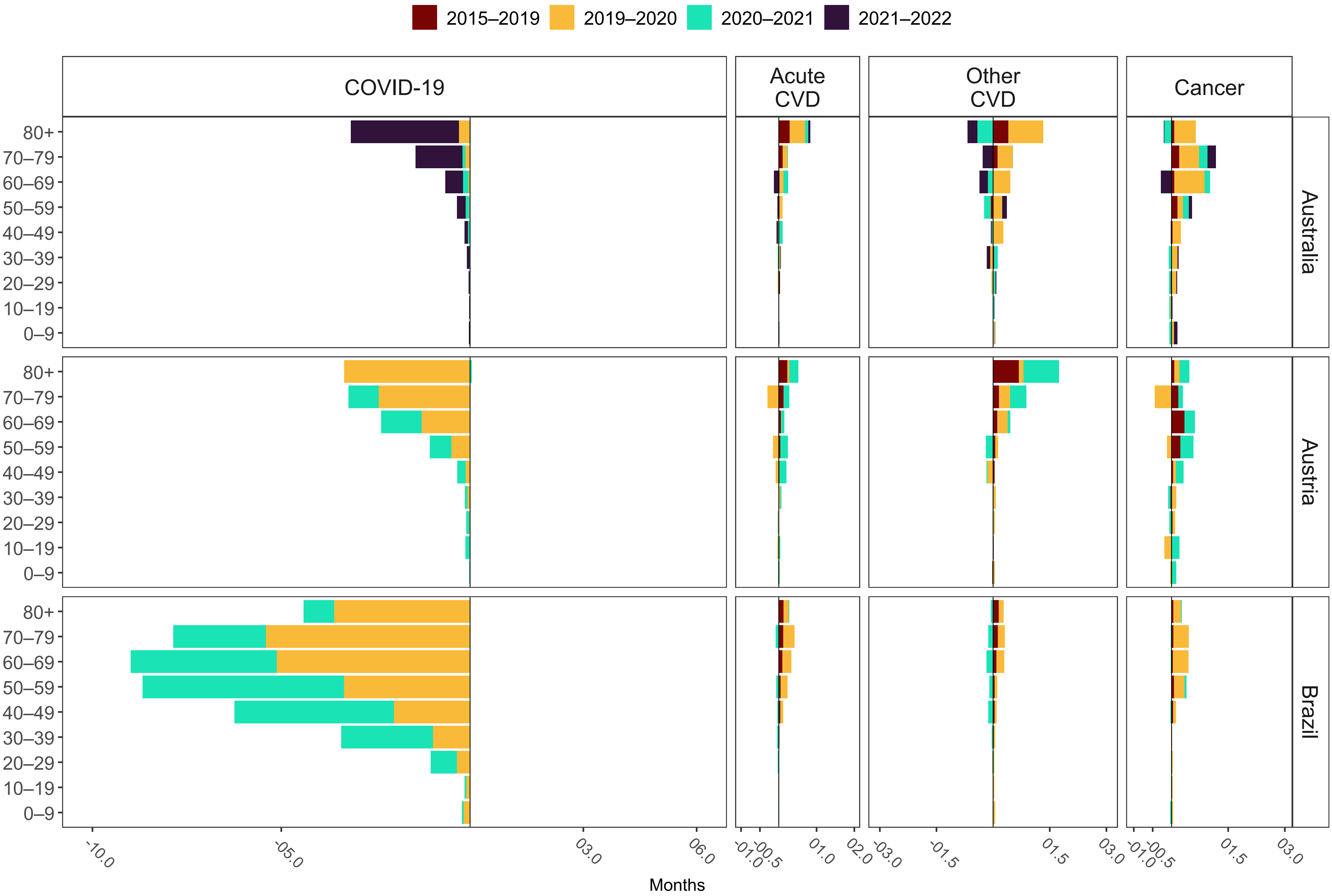

Figure S8b

Contributions to changes in male life expectancy
in Australia, Austria, Brazil

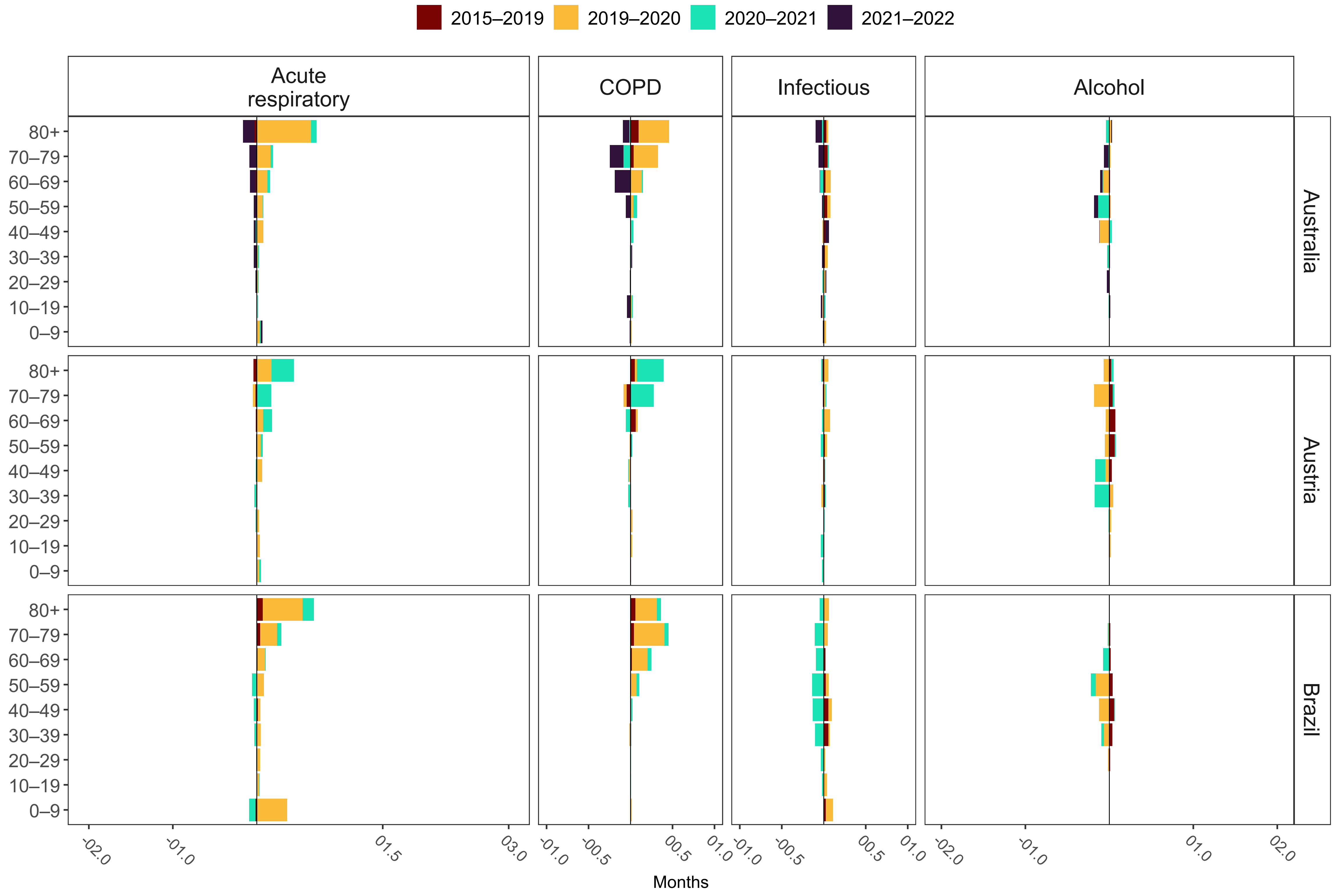

Figure S8c

Contributions to changes in male life expectancy
in Australia, Austria, Brazil

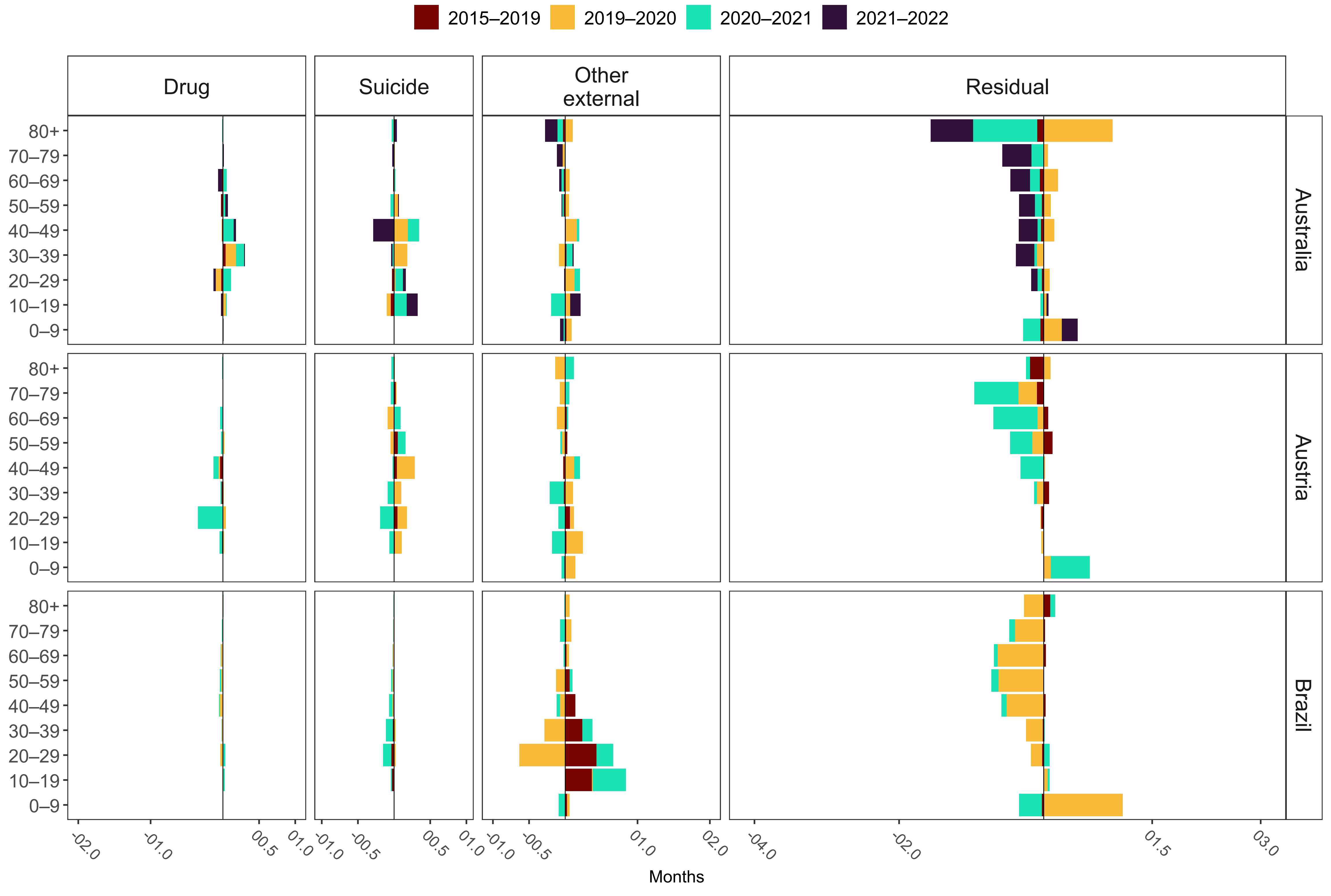

Figure S8d

Contributions to changes in male life expectancy
in Bulgaria, Canada, Chile

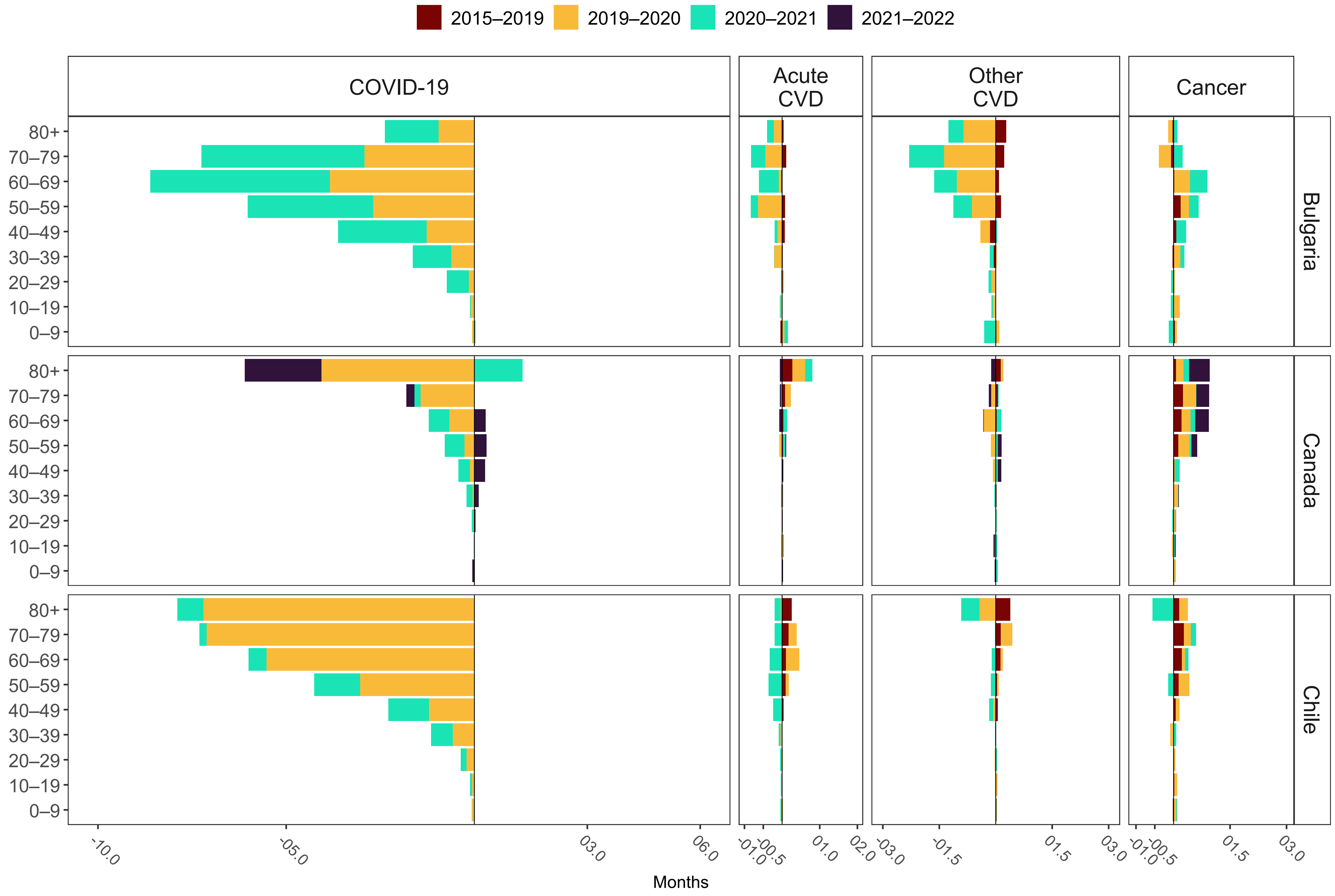

Figure S8e

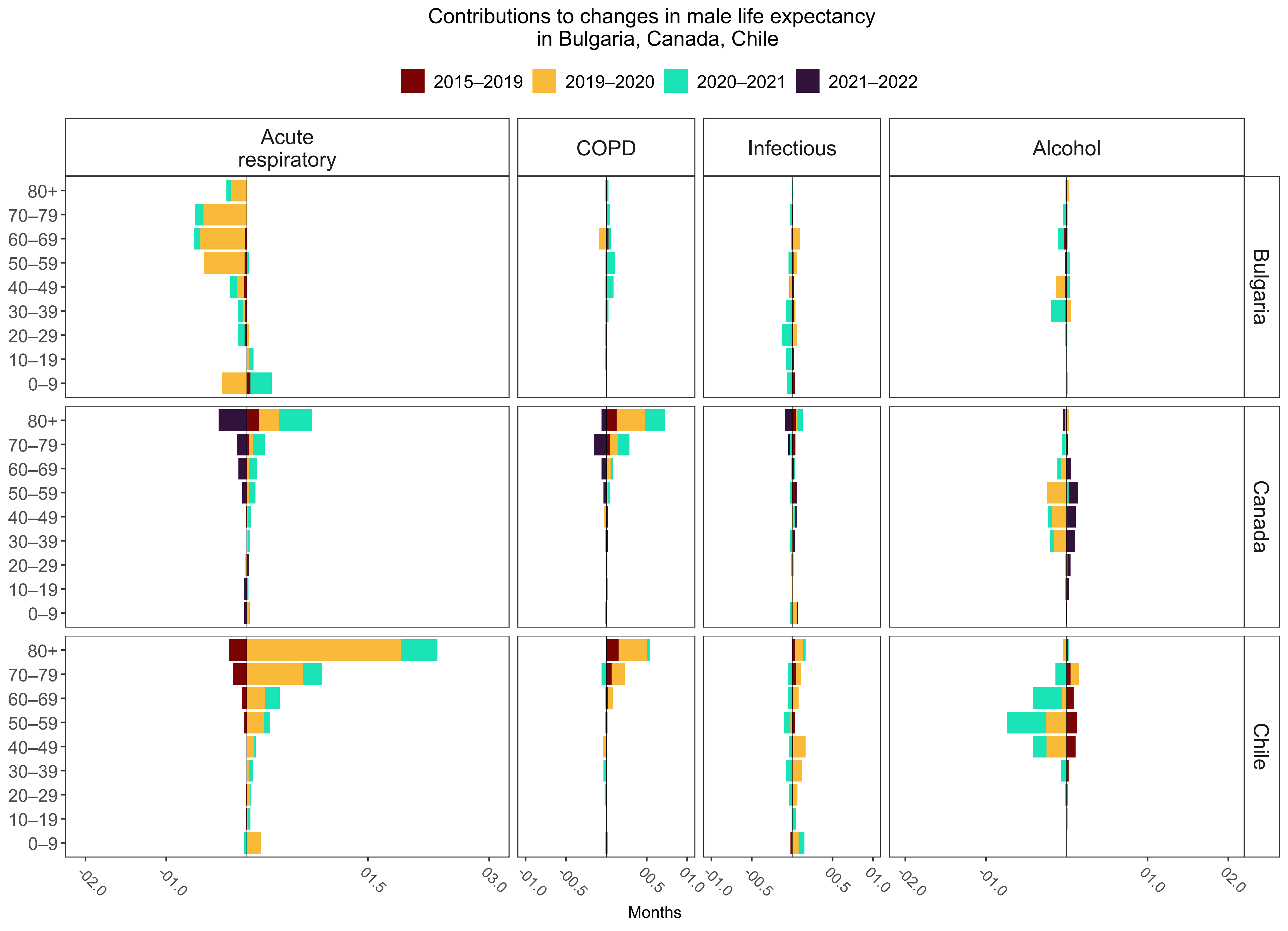
Contributions to changes in male life expectancy
in Bulgaria, Canada, Chile
2015–2019
2019–2020
2020–2021
2021–2022
Acute
respiratory
COPD
Infectious
Alcohol
Bulgaria
Canada
Chile
80+
70–79
60–69
50–59
40–49
30–39
20–29
10–19
0–9
-02.0
-01.0
01.5
03.0
-01.0
-00.5
00.5
01.0
-01.0
-00.5
00.5
01.0
-02.0
-01.0
01.0
02.0
Months

Figure S8f

Contributions to changes in male life expectancy in Bulgaria, Canada, Chile

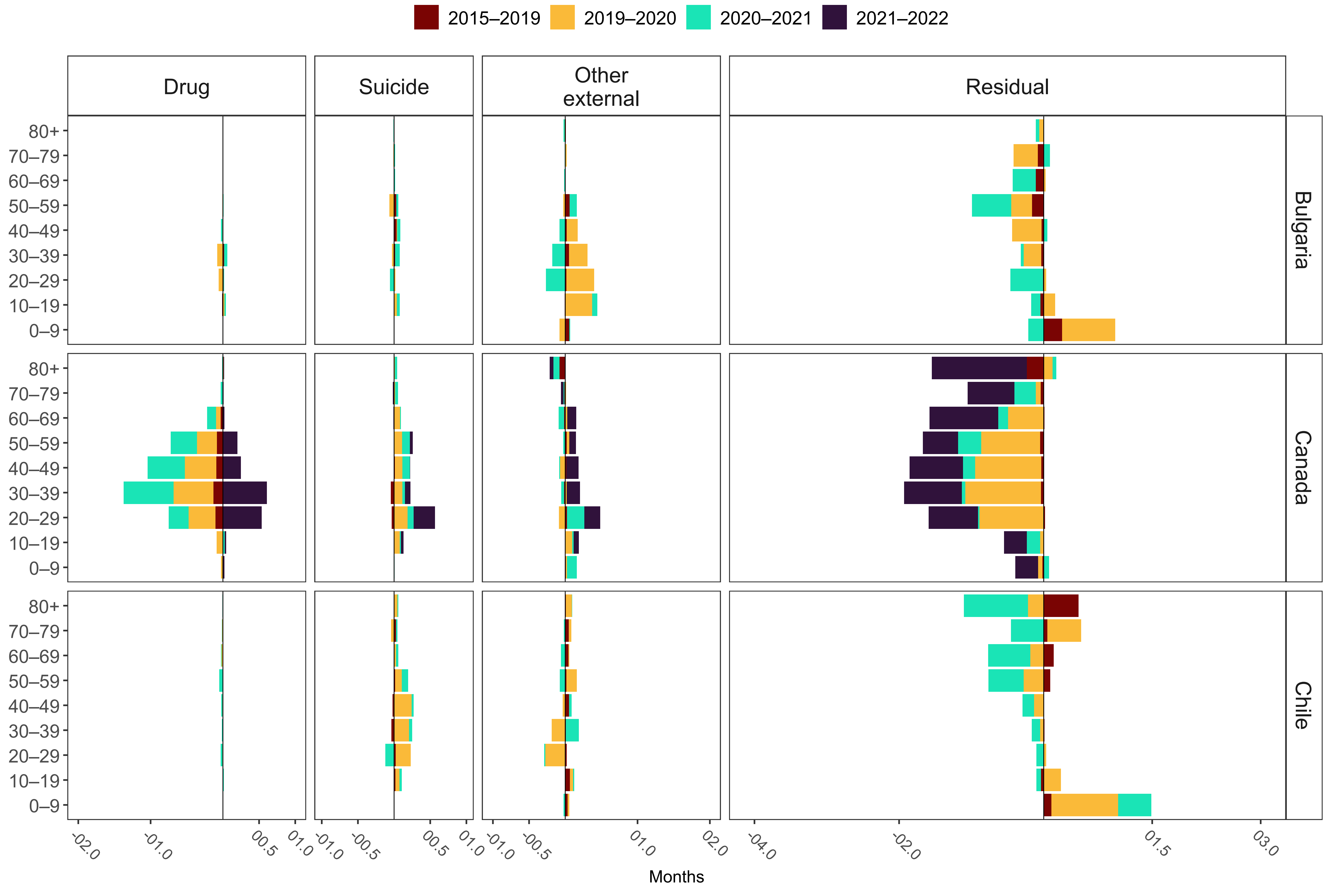

Figure S8g

Contributions to changes in male life expectancy in Croatia, Czechia, Denmark

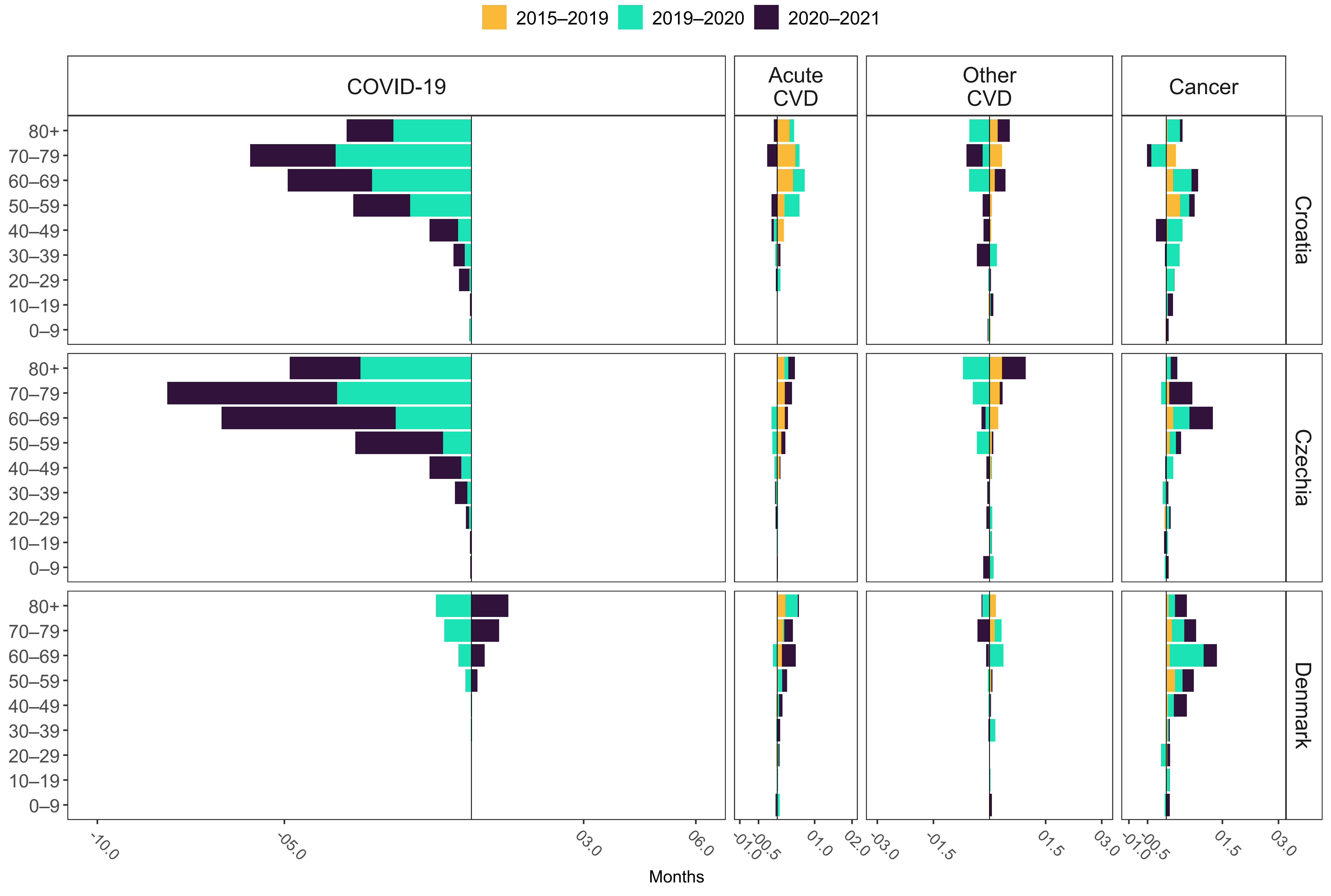

Figure S8h

Contributions to changes in male life expectancy
in Croatia, Czechia, Denmark

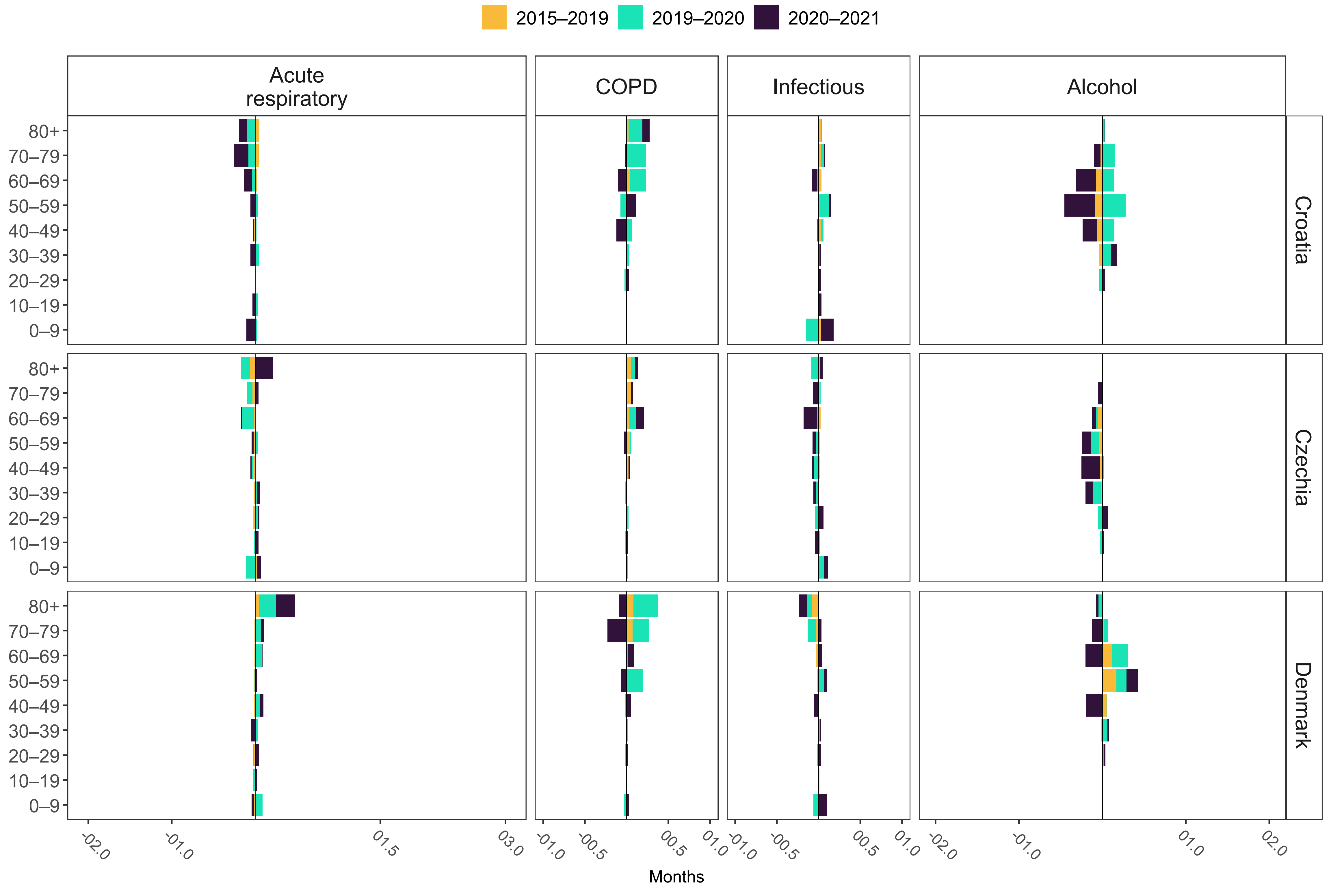

Figure S8i

Contributions to changes in male life expectancy
in Croatia, Czechia, Denmark

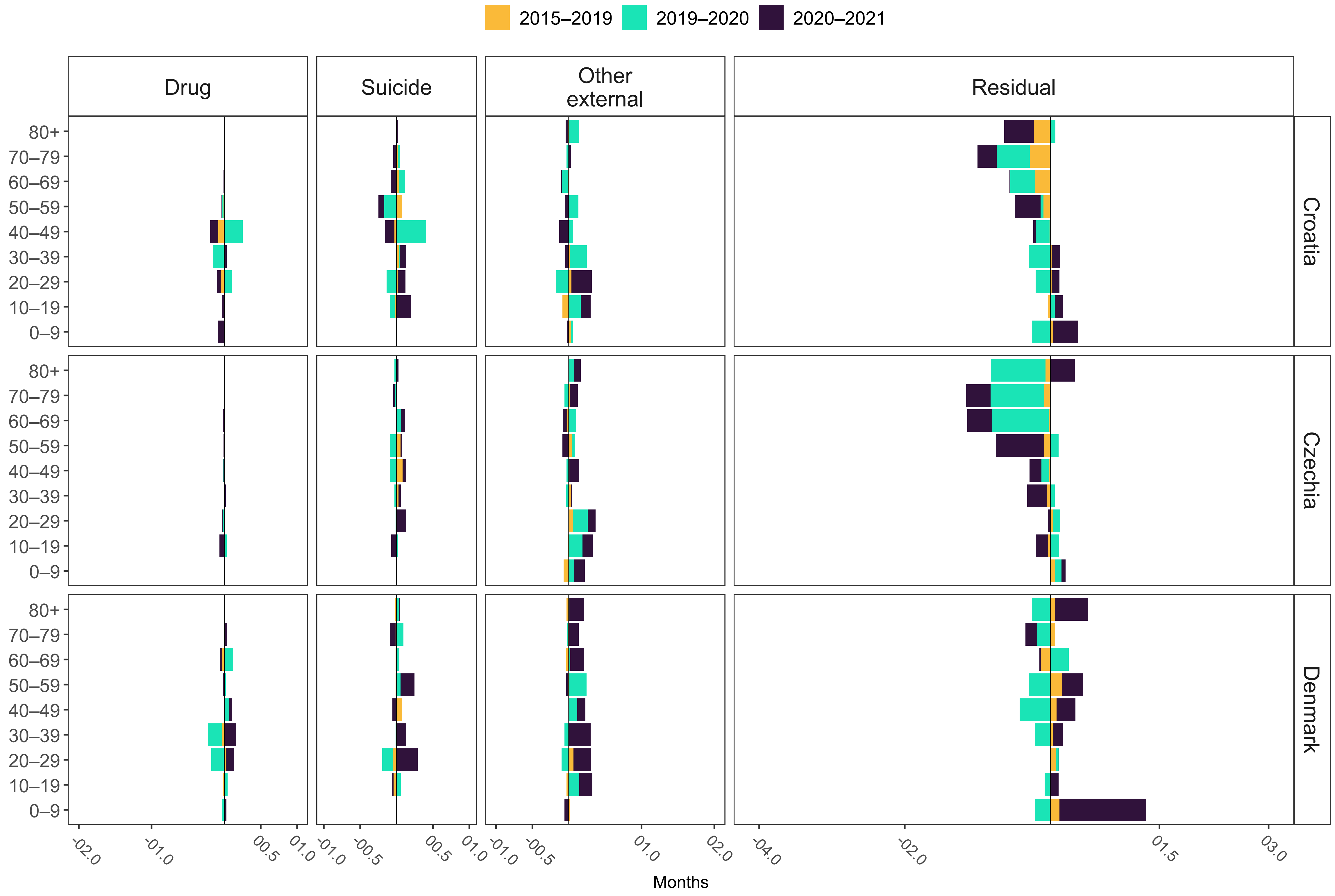

Figure S8j

Contributions to changes in male life expectancy in England & Wales, Hungary, Japan

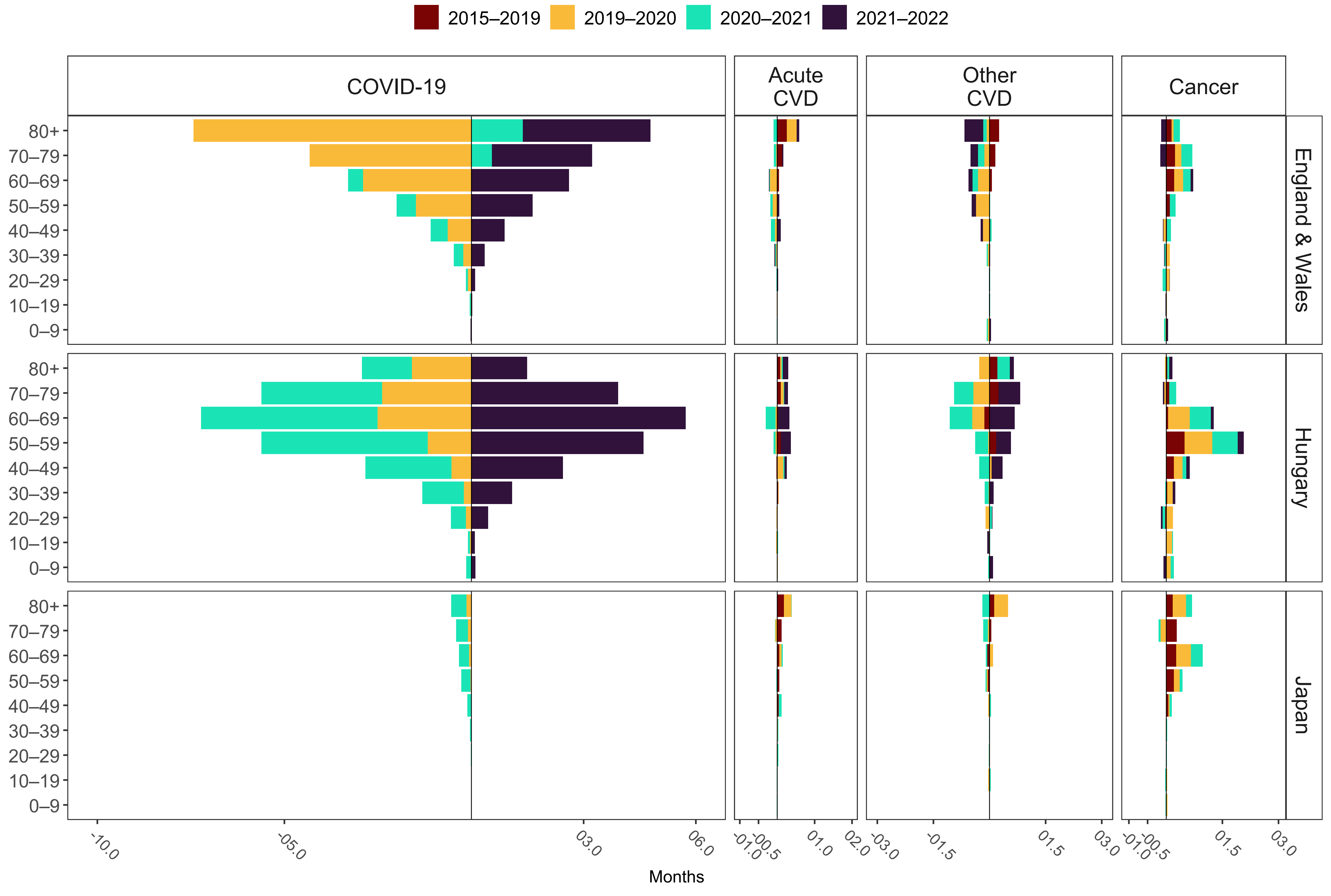

Figure S8k

Contributions to changes in male life expectancy in England & Wales, Hungary, Japan

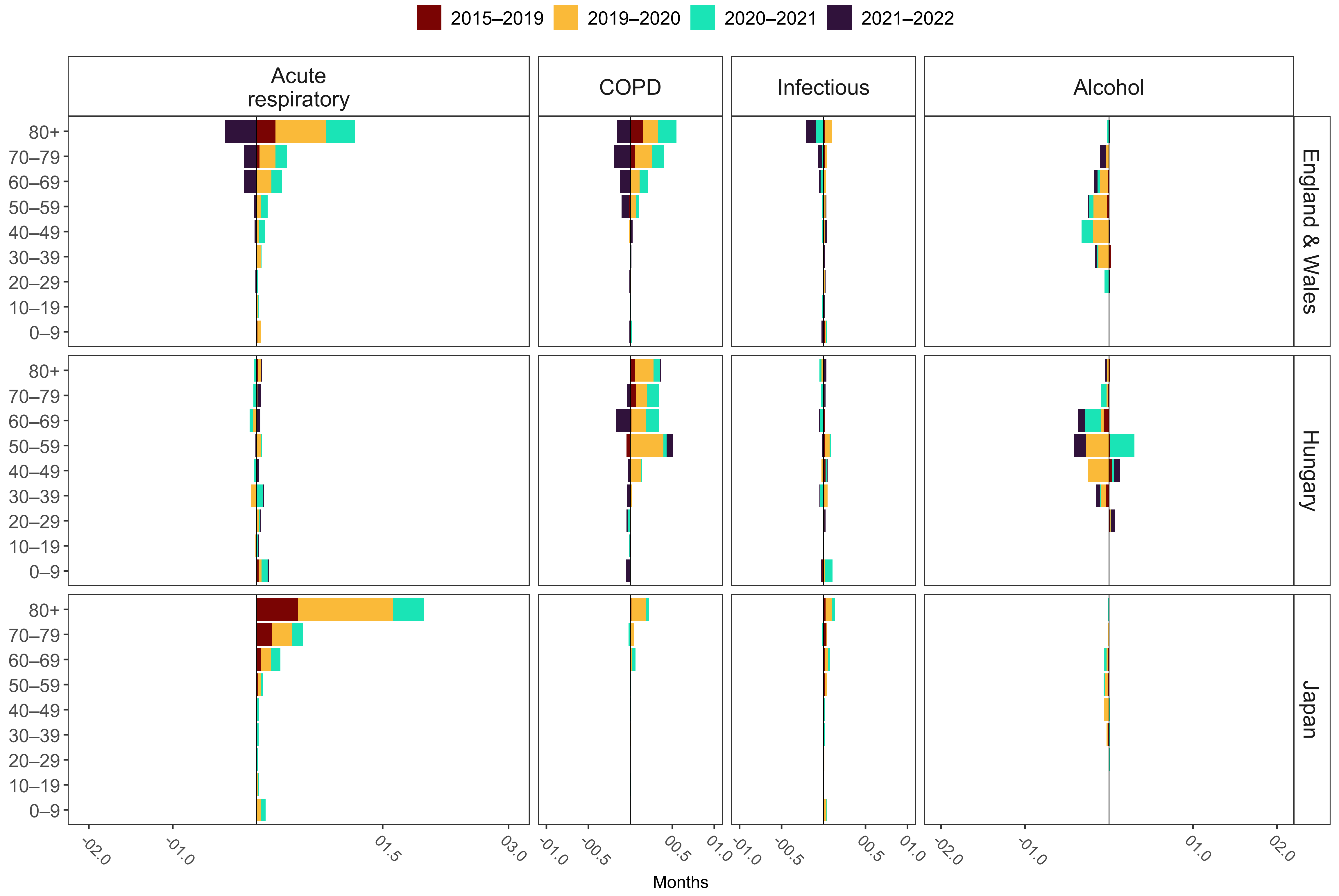

Figure S8I

Contributions to changes in male life expectancy
in England & Wales, Hungary, Japan

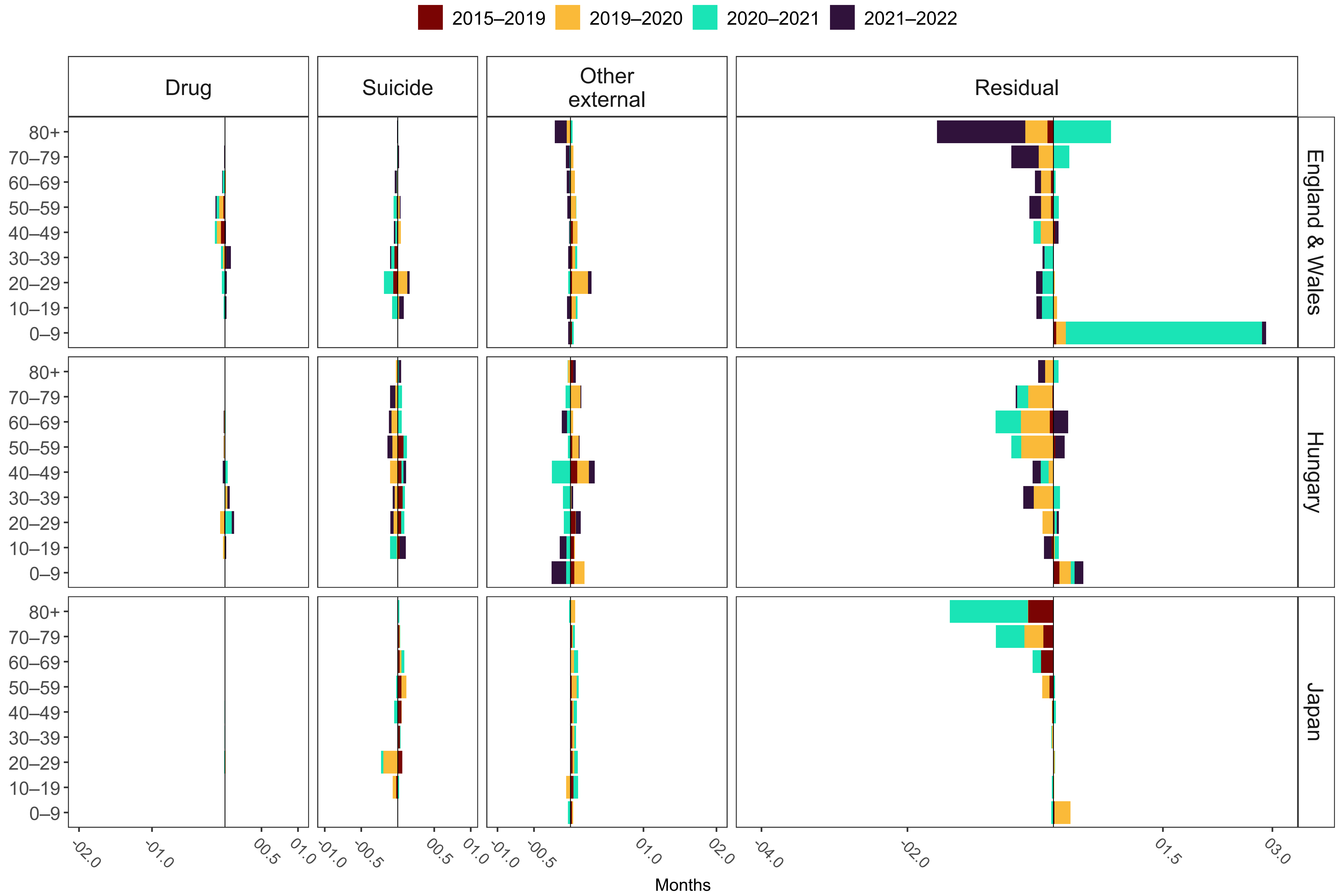

Figure S8m

Contributions to changes in male life expectancy
in Latvia, Lithuania, Netherlands

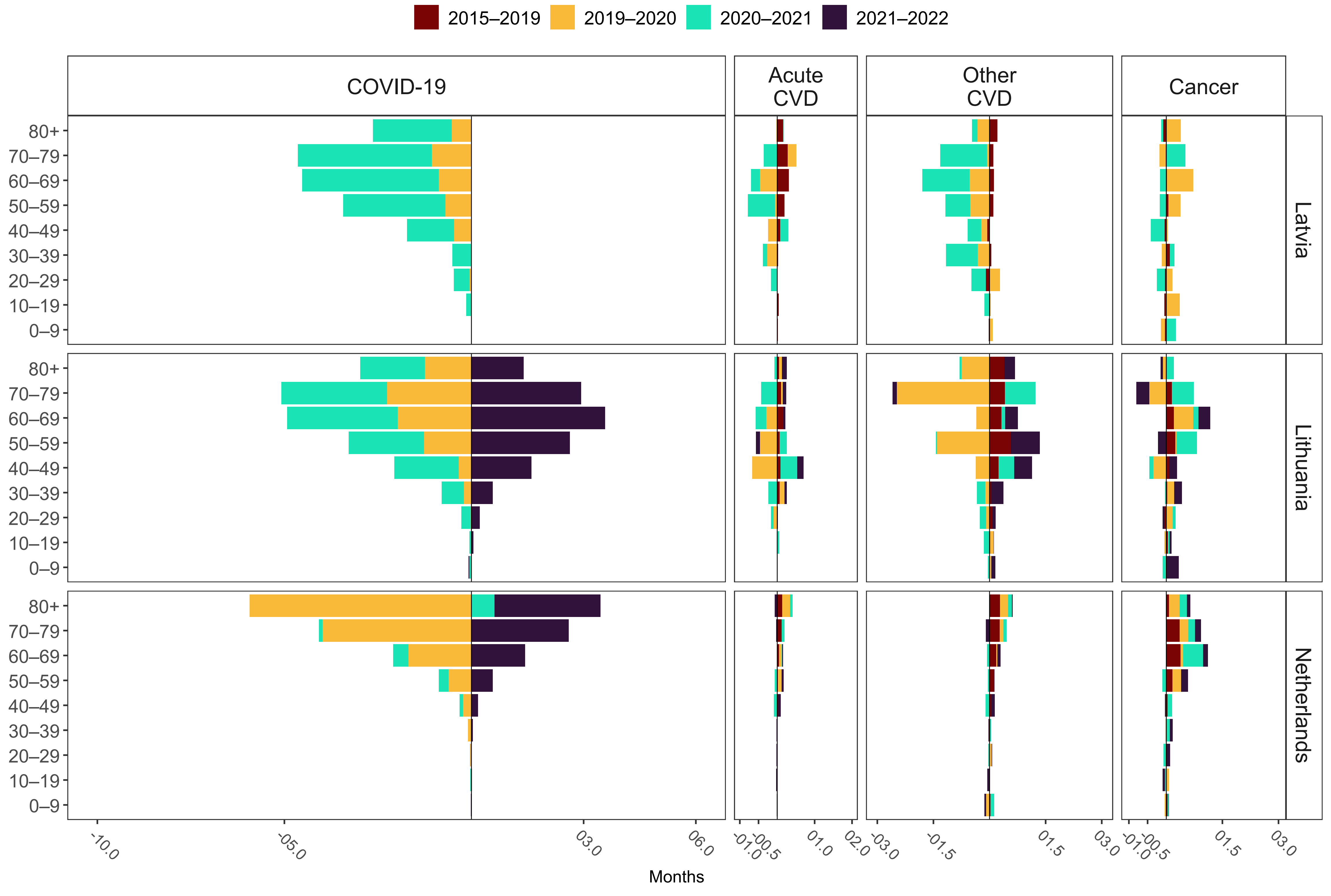

Figure S8n

Contributions to changes in male life expectancy
in Latvia, Lithuania, Netherlands

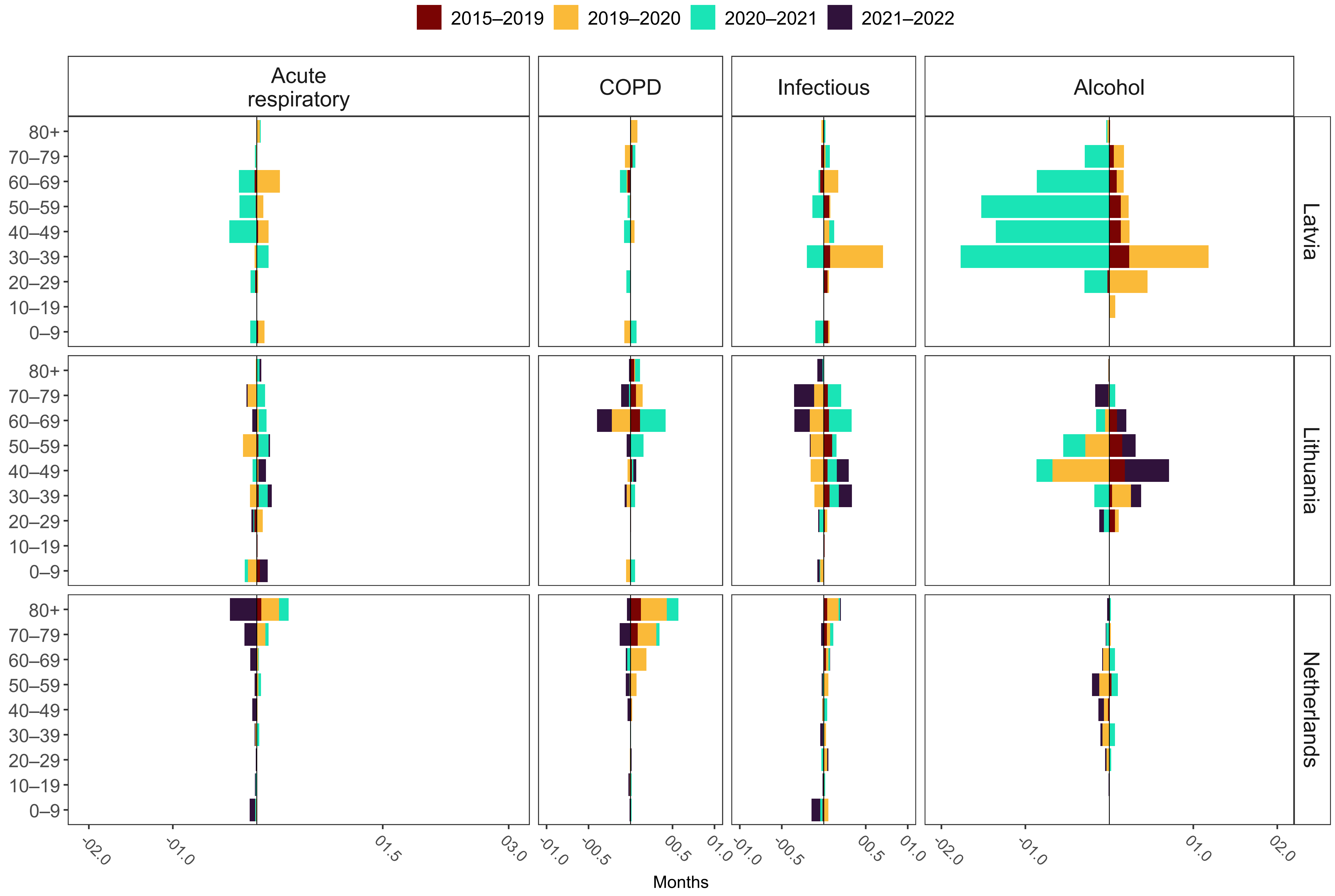

Figure S8o

Contributions to changes in male life expectancy
in Latvia, Lithuania, Netherlands

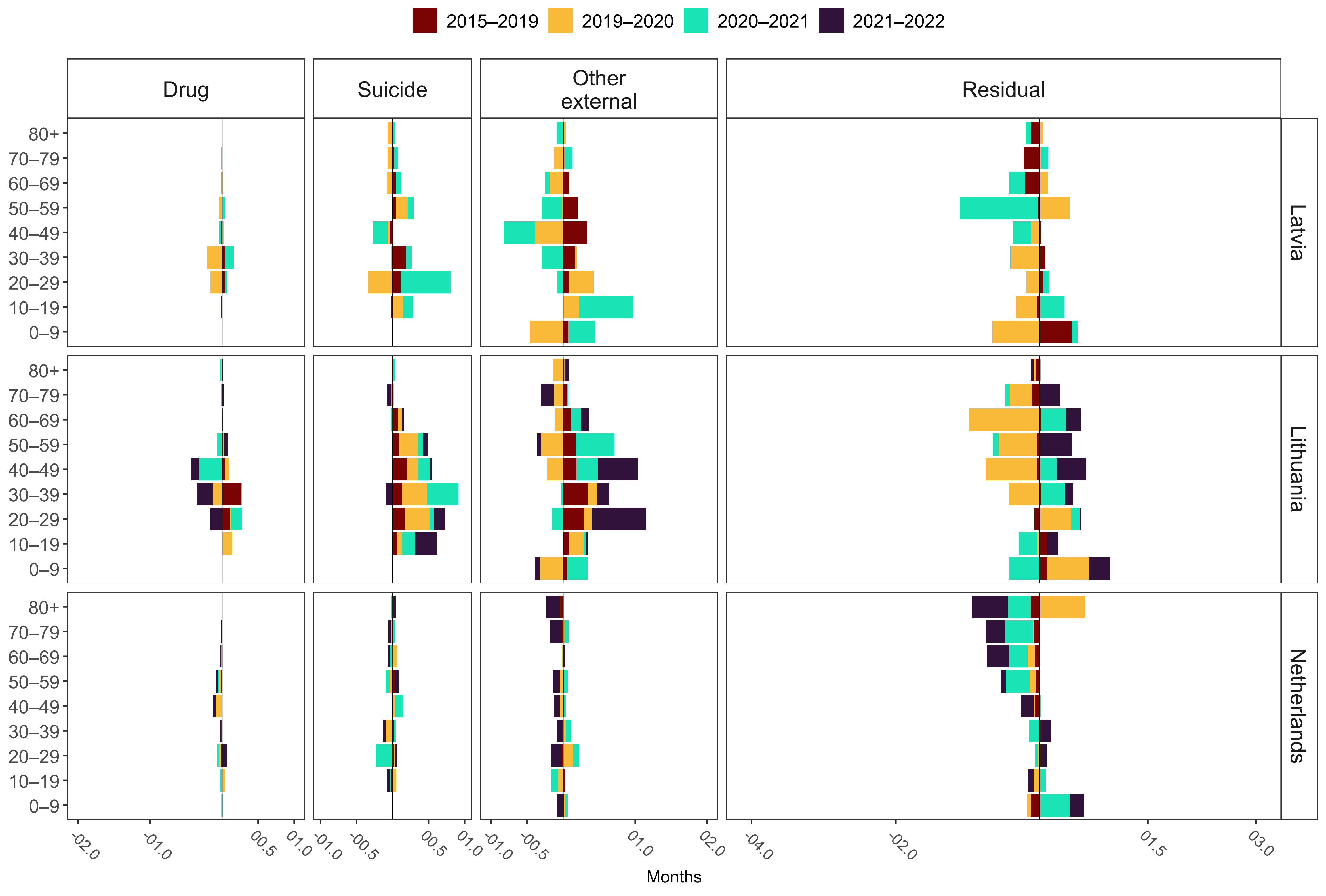

Figure S8p

Contributions to changes in male life expectancy in N. Ireland, Poland, Russia

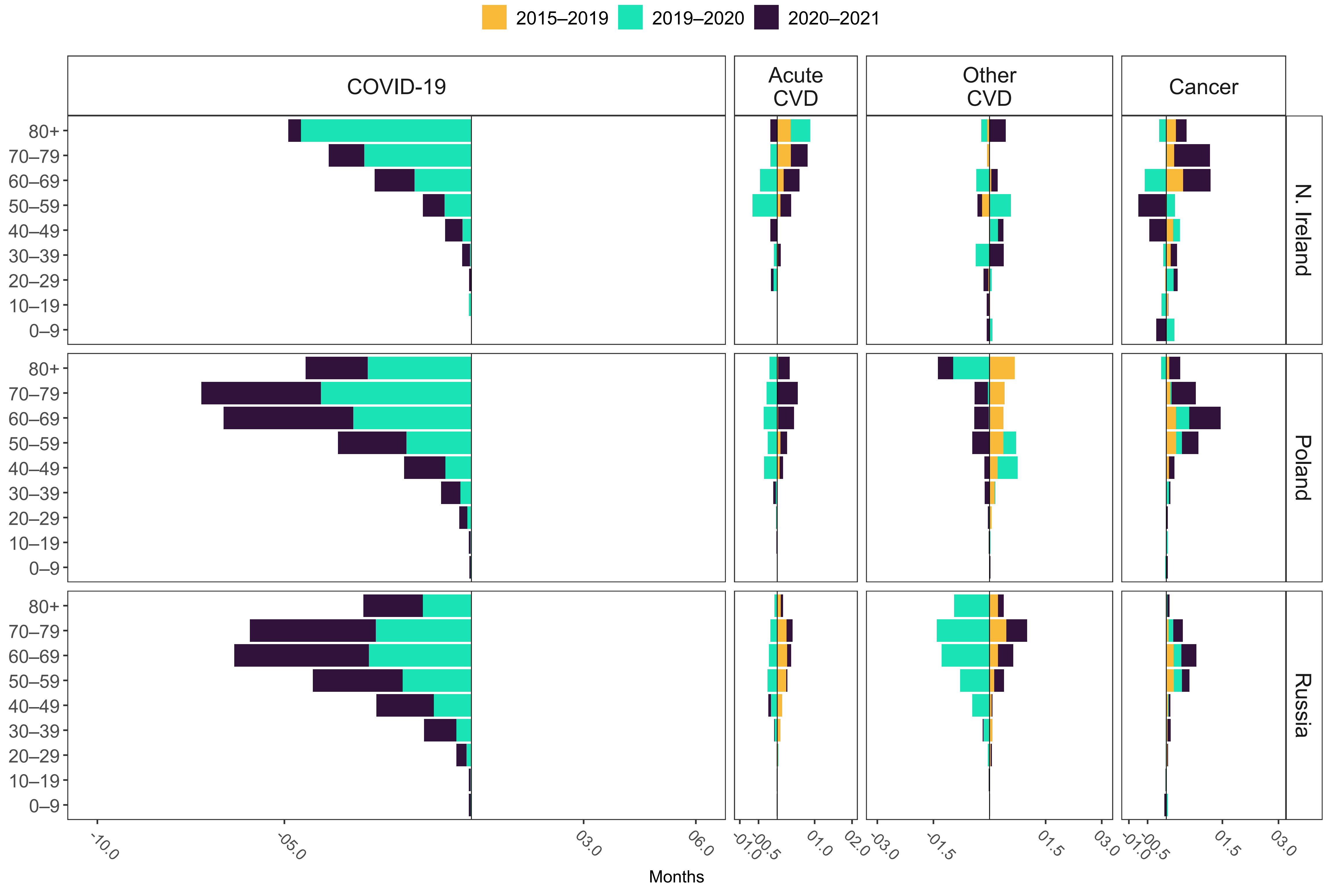

Figure S8q

Contributions to changes in male life expectancy
in N. Ireland, Poland, Russia

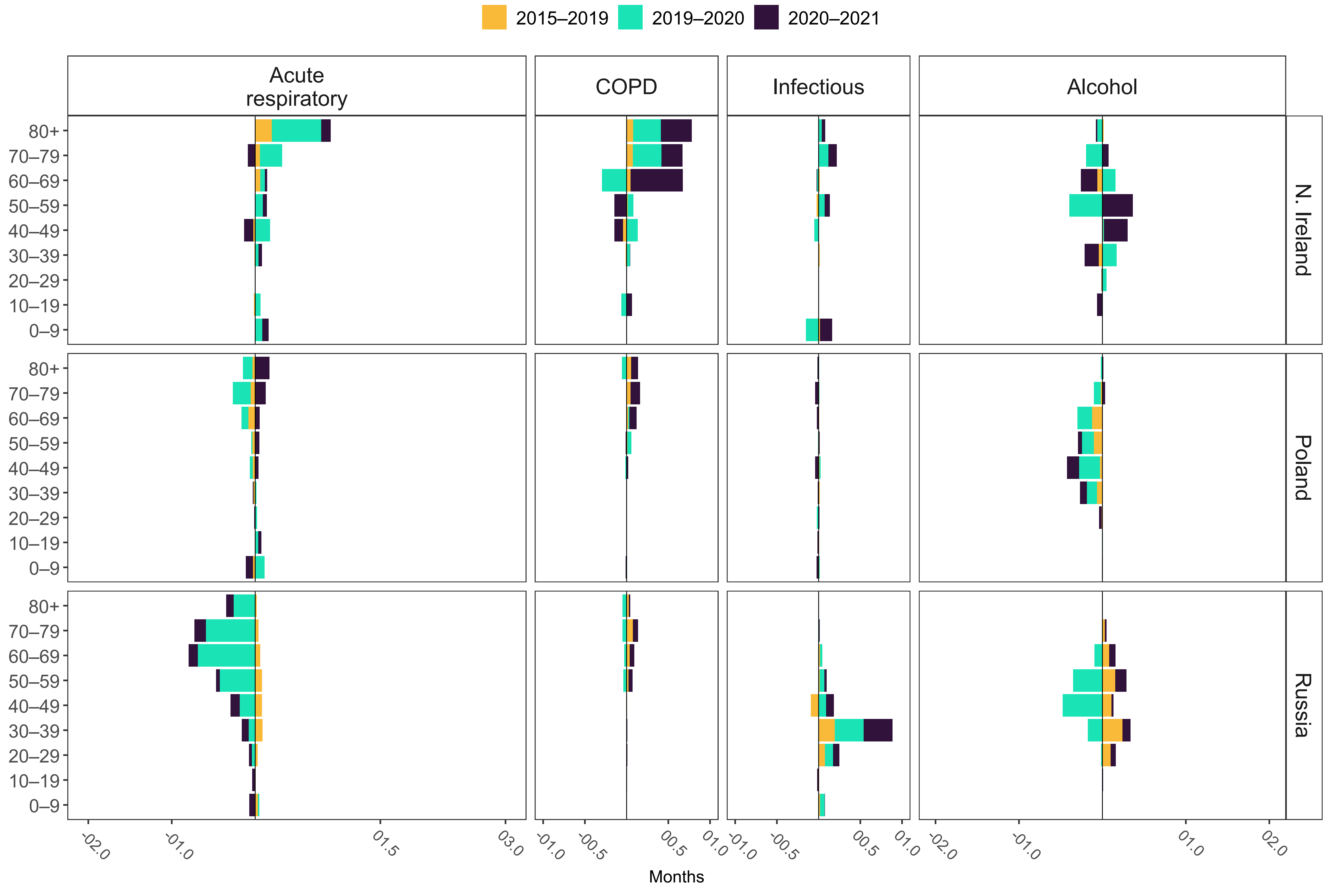

Figure S8r

Contributions to changes in male life expectancy
in N. Ireland, Poland, Russia

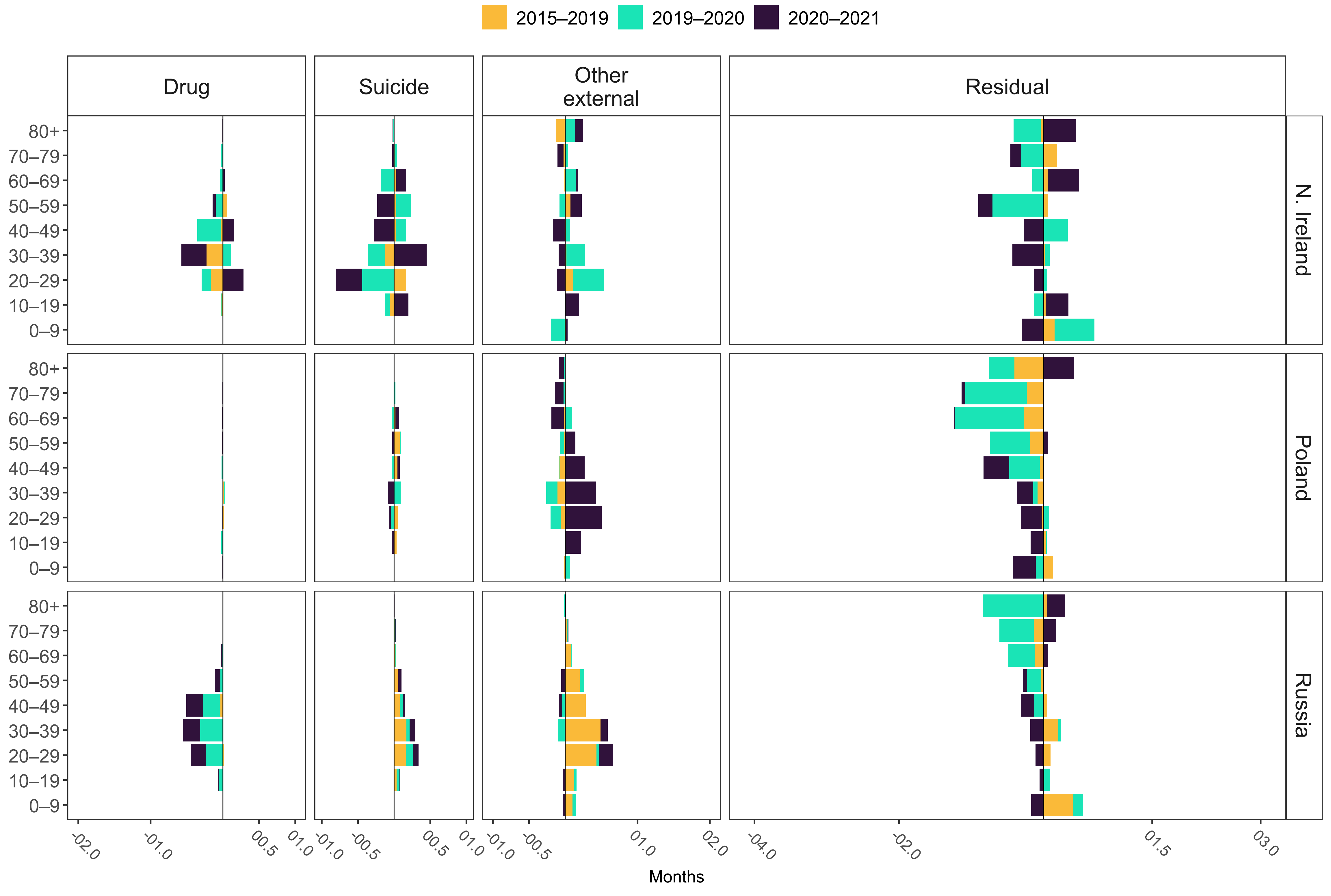

Figure S8s

Contributions to changes in male life expectancy
in Scotland, South Korea, Spain

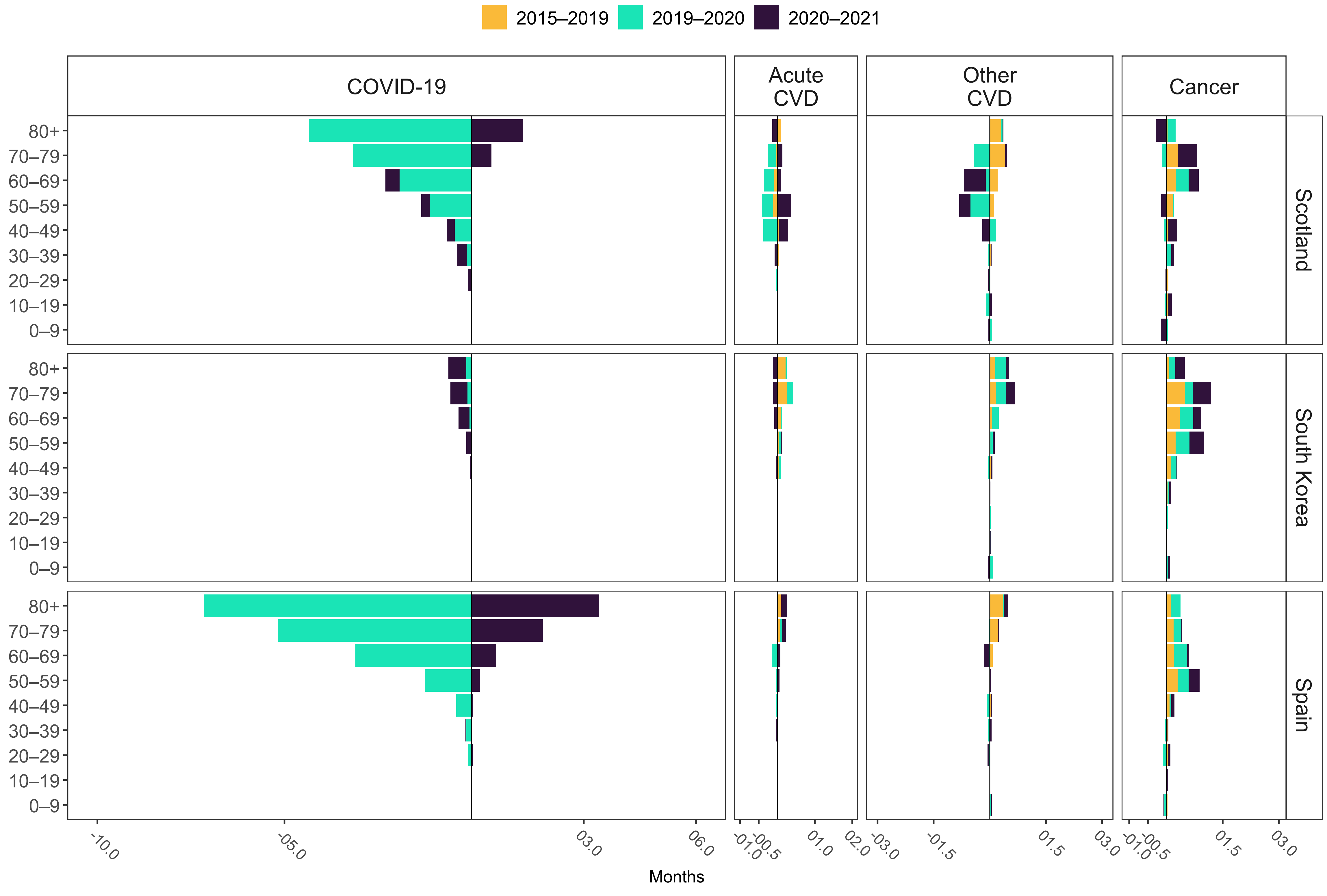

Figure S8t

Contributions to changes in male life expectancy
in Scotland, South Korea, Spain

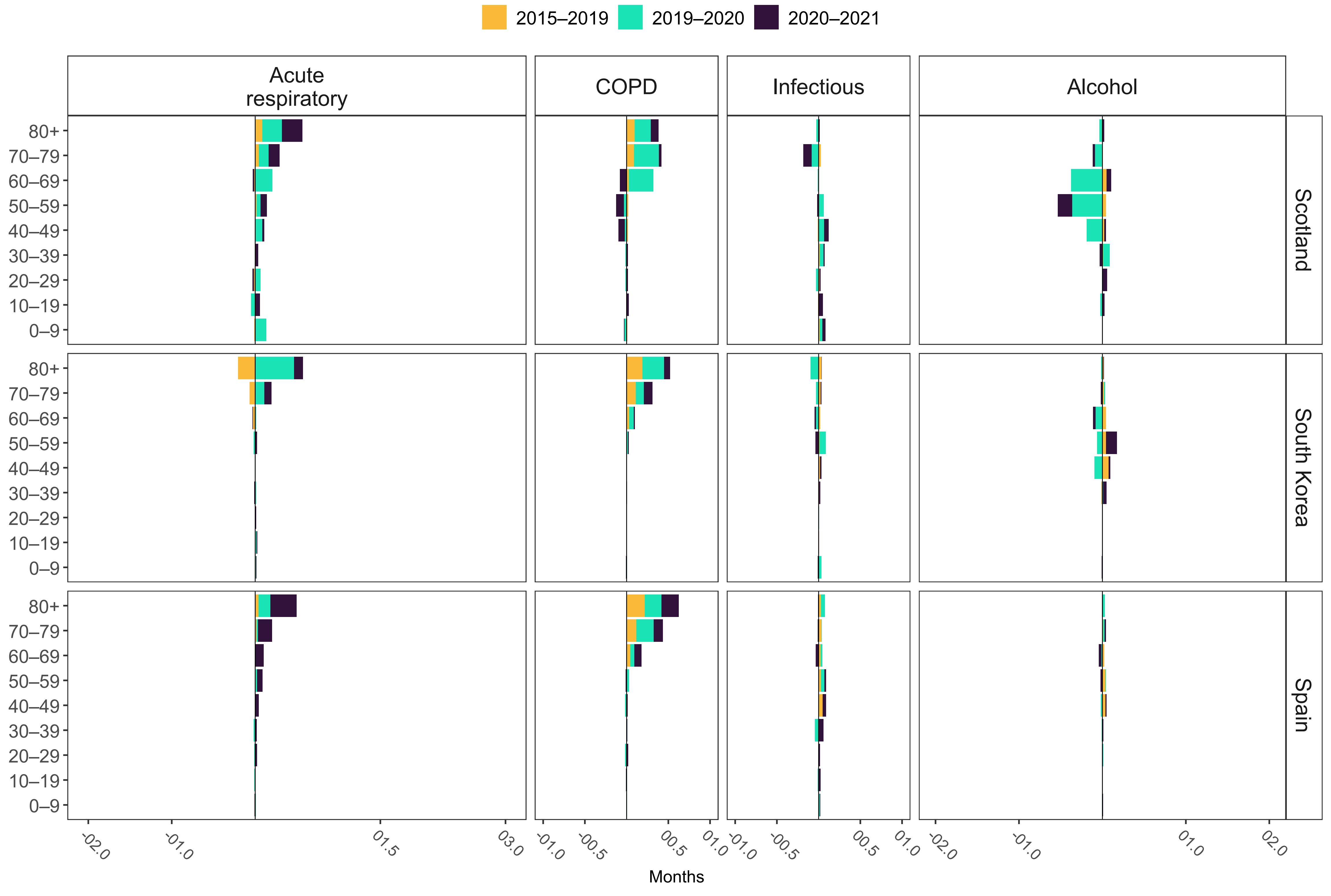

Figure S8u

Contributions to changes in male life expectancy
in Scotland, South Korea, Spain

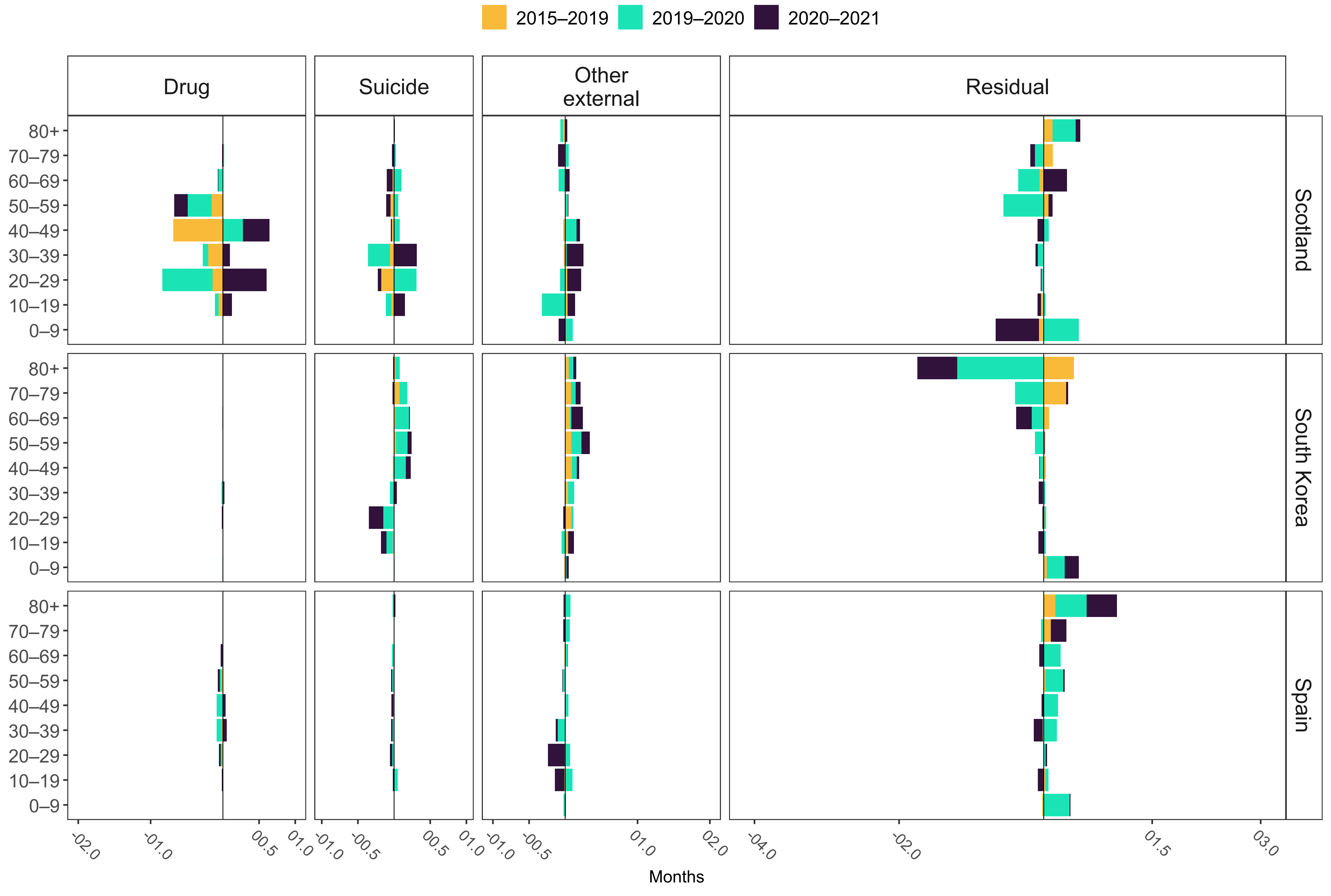

Figure S8v

Contributions to changes in male life expectancy
in Sweden, Switzerland, USA

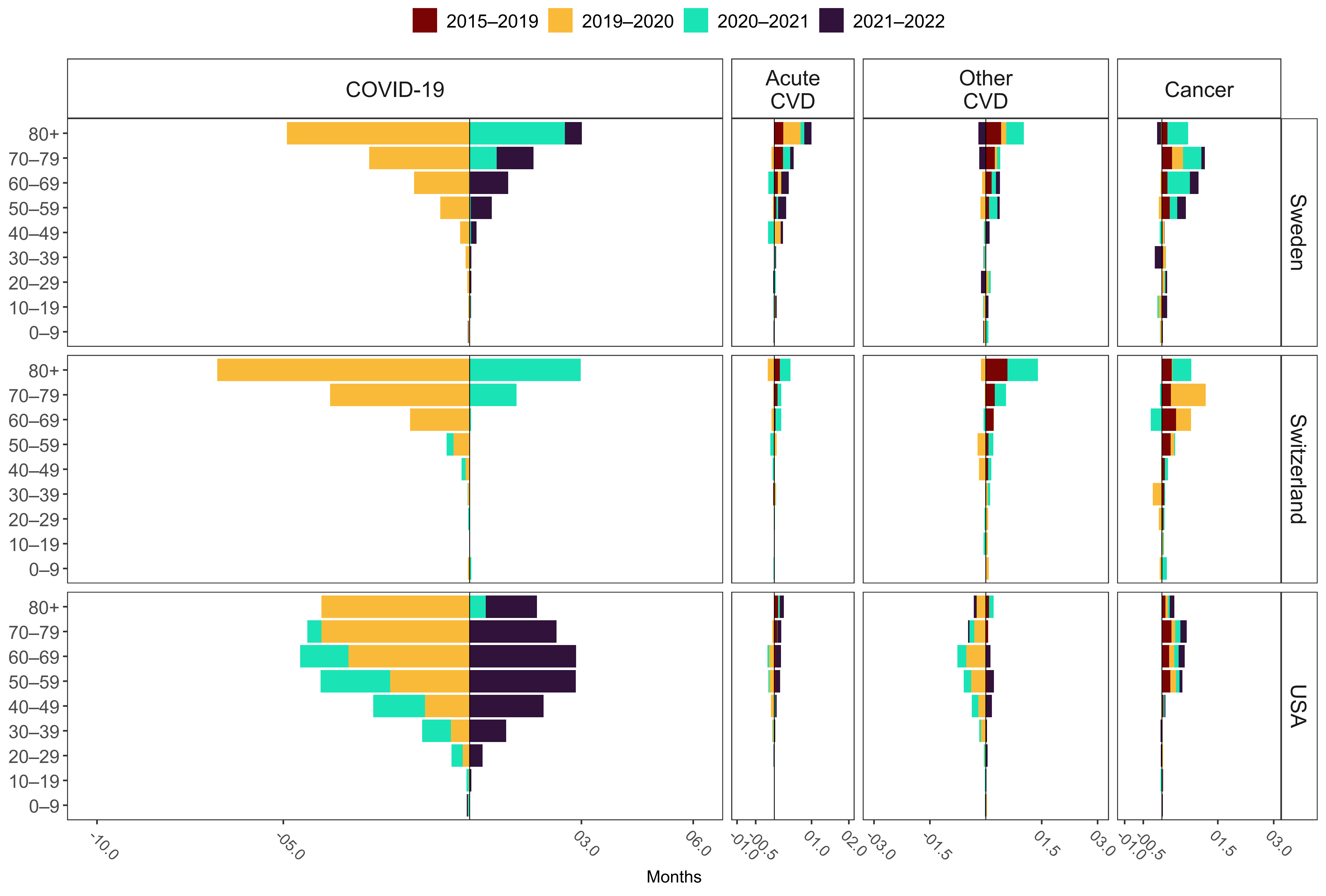

Figure S8w

Contributions to changes in male life expectancy in Sweden, Switzerland, USA

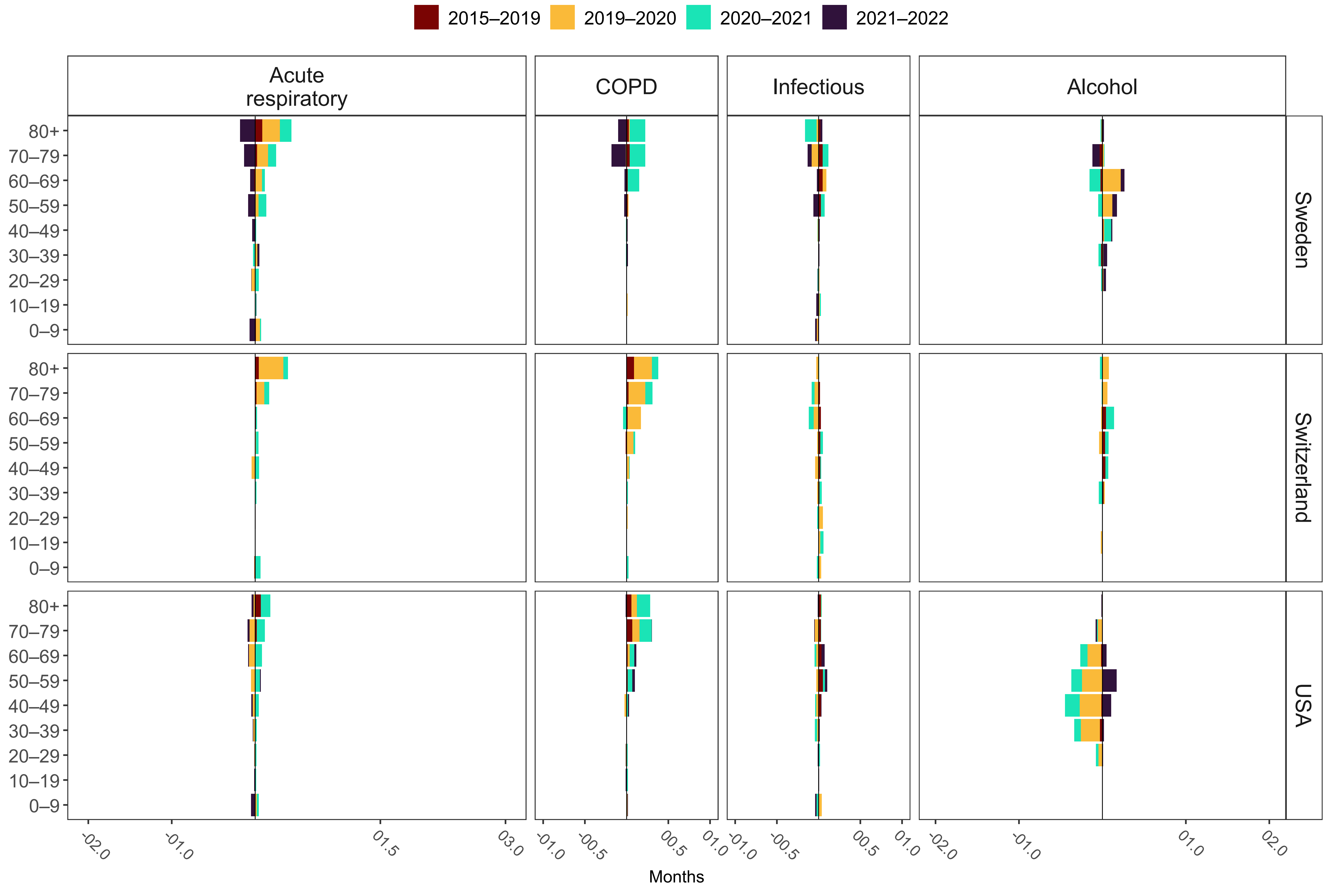

Figure S8x

Contributions to changes in male life expectancy in Sweden, Switzerland, USA

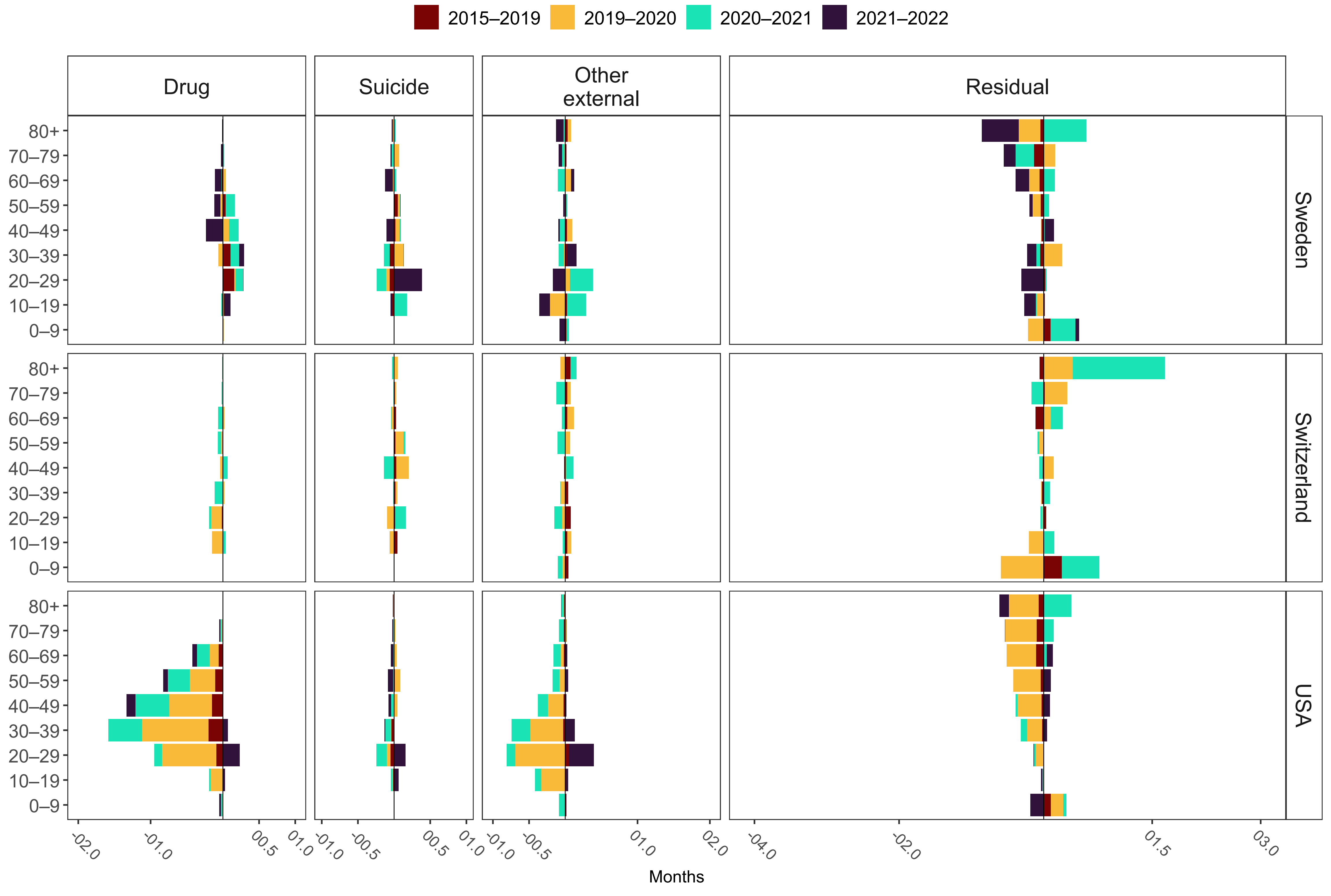

Supplement: pgae508_Supplementary_Data [file pgae508_supplementary_data.pdf]
